# Global, regional, and national burden and projections of severe heart failure attributable to hypertensive, ischemic, and rheumatic heart diseases: an analysis from the global burden of disease study 2021

**DOI:** 10.3389/fcvm.2026.1649064

**Published:** 2026-03-09

**Authors:** Gang Xue, Yuyan Hou, Shuhong Su, Weidong Jin, Xiao Wu, Zhitao Gao, Zhifang Wang

**Affiliations:** 1 Xinxiang Central Hospital, Xinxiang, Henan, China; 2 School of Medical Technology, Henan Medical University, Xinxiang, Henan, China

**Keywords:** disease burden, global burden of disease (GBD), health inequality, hypertensive heart disease, ischemic heart disease, rheumatic heart disease, severe heart failure

## Abstract

**Background:**

Severe heart failure (SHF) caused by hypertensive heart disease (HHD), ischemic heart disease (IHD), and rheumatic heart disease (RHD) remains a significant global public health concern. Accurate assessments of the global and regional burdens, trends, and health inequalities related to these diseases are critical for formulating targeted health policies and interventions.

**Methods:**

Data on the prevalence and years lived with disability (YLDs) due to severe heart failure resulting from HHD, IHD, and RHD from 1990 to 2021 were extracted from the Global Burden of Disease (GBD) Study 2021. Trends were analyzed according to age-standardized rates (ASRs) and sociodemographic index (SDI) stratifications. Predictions for 2022–2040 were generated using Nordpred modeling and validated with the Bayesian Age-Period-Cohort (BAPC) model.

**Results:**

In 2021, prevalent cases for severe heart failure attributable to HHD, IHD, and RHD reached 4.07 million, 6.24 million, and 0.74 million, respectively, with corresponding age-standardized prevalence rates (ASPR) of 48.3, 74.4, and 9.2 per 100,000. Compared with 1990, cases increased by 170.3% for HHD, 140.7% for IHD, and 39.0% for RHD. YLDs rose to 700,272 (HHD), 1,075,512 (IHD), and 129,155 (RHD), respectively. The greatest burdens occurred in Eastern Sub-Saharan Africa for HHD (ASPR: 95.0), North Africa and the Middle East for IHD (ASPR: 103.1), and South Asia for RHD (ASPR: 21.0). Projections indicate continued increases in prevalent cases and YLDs for all conditions, with stable or slightly rising standardized rates, highlighting persistent regional and gender inequalities.

**Conclusion:**

Severe heart failure burdens related to HHD, IHD, and RHD are increasing globally, influenced significantly by demographic shifts and socioeconomic disparities. Urgent implementation of context-specific preventive and healthcare strategies is essential to address health inequalities and reduce the future burden of severe heart failure worldwide.

## Introduction

Cardiovascular diseases remain the foremost contributor to global mortality, with heart failure serving as a critical endpoint for diverse etiologies shaped by biological, environmental, and socioeconomic interplay (1). Hypertensive heart disease (HHD), ischemic heart disease (IHD), and rheumatic heart disease (RHD) collectively drive severe heart failure (SHF) worldwide, yet their epidemiological trajectories diverge markedly across developmental contexts. While IHD dominates in aging, high-income populations amid caloric excess and sedentary lifestyles, HHD thrives in regions with fragmented primary care, and RHD persists as a marker of neglected infectious disease control (2–4). These disparities underscore a paradox of progress: socioeconomic advancement may alleviate historical burdens while incubating new risks, necessitating frameworks that capture nonlinear transitions.

Existing studies often isolate these etiologies or focus on short-term trends, neglecting the dynamic interaction between sociodemographic development and cardiovascular risk evolution. Conventional epidemiological transition models, which posit a linear shift from infectious to non-communicable diseases, fail to explain overlapping burdens in transitional economies where RHD legacies coexist with rising IHD rates (5, 6). Similarly, gender and age-related disparities are frequently oversimplified as biological inevitabilities rather than outcomes of structural inequities in healthcare access and social determinants (7, 8). This study addresses these gaps through a multidecadal, multilevel analysis of severe heart failure burdens, dissecting how uneven developmental trajectories, which marked by disparities in education, income, and healthcare, reshape risk portfolios.

The evolution of health inequities further reflects systemic biases in global health governance. Although targeted interventions have modestly reduced RHD disparities, widening gaps in HHD burden highlight the marginalization of chronic conditions in policy agendas. Conversely, the persistent prevalence of IHD in affluent societies illustrates how available preventive and therapeutic options often remain underutilized due to entrenched cultural norms and systemic barriers within health systems (9, 10). These patterns demand interventions that transcend etiology-specific silos, addressing shared structural determinants such as antibiotic accessibility and salt reduction policies while adapting to regional transition phases.

Ultimately, this study seeks to redefine cardiovascular disease management paradigms by advocating integrated and equity-oriented strategies. By systematically documenting how severe heart failure burdens from HHD, IHD, and RHD align with socioeconomic and health system factors, it underscores the urgency for multidimensional interventions that address fundamental societal determinants. Only through comprehensive, context-specific, and integrated public health approaches can global stakeholders effectively mitigate the disproportionate and evolving burden of severe heart failure worldwide.

## Methods

### Study design and data sources

This investigation employed a retrospective analytical framework utilizing publicly accessible data from the Global Burden of Disease (GBD) Study 2021. We assessed the global, regional, and national burdens and temporal trends of severe heart failure (SHF) attributable to hypertensive heart disease (HHD), ischemic heart disease (IHD), and rheumatic heart disease (RHD) across 204 countries and territories from 1990 to 2021. Future burdens through 2040 were projected and validated using the Nordpred model and the Bayesian Age-Period-Cohort (BAPC) model with integrated nested Laplace approximations. The standardized methodology of GBD synthesizes diverse epidemiological data, ensuring comparability across regions and periods. Comprehensive descriptions of data collection, quality assurance, statistical modeling, and uncertainty estimation are detailed in prior GBD publications (11).

### Key metrics and temporal trend analysis

Primary outcome measures included annual prevalent cases, years lived with disability (YLDs), age-standardized prevalence rates (ASPR), and age-standardized YLD rates (ASYR) related to severe heart failure resulting from HHD, IHD, and RHD. Temporal trends were quantified using the estimated annual percentage change (EAPC), calculated through log-linear regression models applied to age-standardized rates (12):

$$\mathrm{EAPC}=(e^{\beta }-1)\times 100$$

where *β* is the slope of the natural logarithm of the ASR over time, derived from the linear regression model:

ln(ASR)=β×Year+α

Here, *α* is the intercept. A positive EAPC indicates an increasing trend, while a negative EAPC suggests a decline. Statistical significance was defined as 95% CIs not crossing zero.

### Disease definition

In Global Burden Disease, HHD is classified as a distinct cause, defined as a group of conditions leading to mortality or disability. In GBD 2021, HHD was mapped to ICD-10 codes I11-I11.9 and ICD-9 codes 402-402.91.

IHD is heart damage caused by narrowed heart arteries. IHD includes acute myocardial infarction and chronic IHD. In GBD 2021, IHD was coded according to the International Classification of Diseases, 10th Revision (as ICD-10).

RHD is defined as valvular damage resulting from rheumatic fever. In GBD 2021, RHD was coded ICD-10 codes I01-I01.9, I02.0, and I05-109.9, and in ICD-9 codes 391-391.9, 392.0, and 393-398.99.

### Socioeconomic and geographic stratification

Countries were stratified by the Sociodemographic Index (SDI)—a composite measure integrating per capita income, educational attainment, and fertility rates—to evaluate the relationship between socioeconomic development and severe heart failure burden from HHD, IHD, and RHD. Additionally, regional variations were analyzed according to 21 GBD-defined regions and five SDI quintiles (high, high-middle, middle, low-middle, low) to identify disparities in epidemiological trends and future burdens across developmental contexts (13).

### Correlation between age-standardized rates and sociodemographic development

To further elucidate the relationship between sociodemographic development and severe heart failure burdens, Spearman's rank correlation analyses were performed between age-standardized rates (ASPR and ASYR) and SDI at the national level for HHD, IHD, and RHD. This analysis allowed for identification of non-linear or complex relationships between disease burdens and socioeconomic progress, highlighting epidemiological transition stages in different regions (14, 15).

### Cross-country and gender inequality analysis

Cross-country inequalities were quantified using the concentration index (CI), a measure commonly employed to assess the extent of socioeconomic inequalities in health outcomes. Countries were ranked by SDI, and CI values were calculated for ASPR and ASYR of severe heart failure from HHD, IHD, and RHD. A positive CI indicated a concentration of the disease burden among higher-SDI nations, whereas a negative CI reflected a higher burden in lower-SDI settings. Gender disparities were evaluated by comparing standardized prevalence and YLD rates between males and females globally and regionally, identifying significant differences indicative of underlying gender-specific health inequalities (16).

### Projection analysis

Projections of severe heart failure burden from 2022 to 2040 were initially generated using the Nordpred modeling framework, accounting for age-period-cohort interactions. To enhance projection robustness and ensure accuracy, the Bayesian Age-Period-Cohort (BAPC) model with integrated nested Laplace approximations was utilized for validation. Consistency of results from both modeling approaches confirmed projection stability. Projected metrics included prevalent cases, YLDs, ASPR, and ASYR for each condition stratified by region and sex, thus providing a comprehensive view of future epidemiological trajectories (17).

All statistical analyses and geospatial visualizations were conducted using R statistical software (version 4.4.2).

## Results

### Global burden and trends of severe heart failure due to HHD, IHD, and RHD

#### Severe heart failure due to HHD

**Figure 1:**
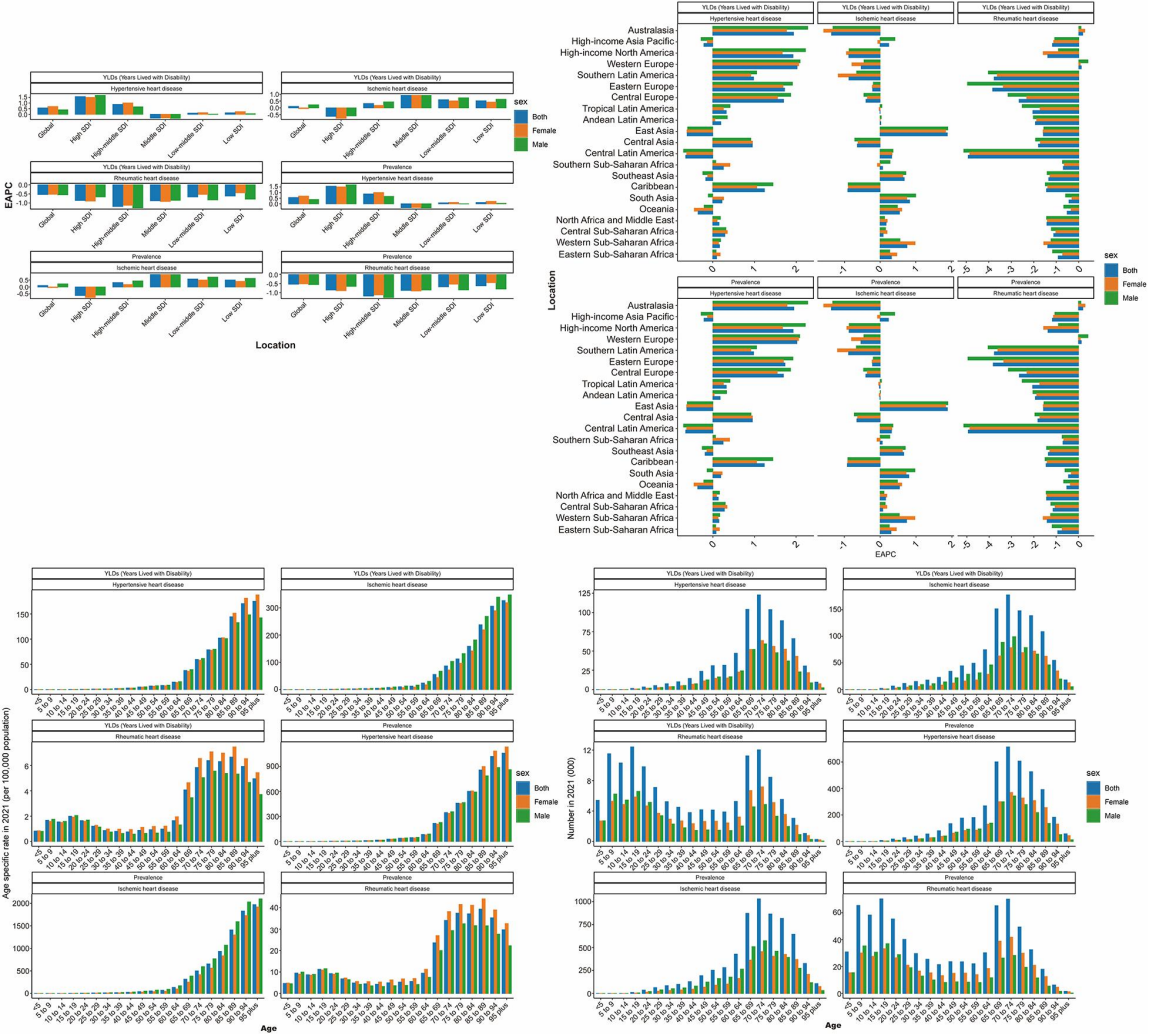
EAPC in ASPR and ASYR by global, SDI, and GBD regions from 1990 to 2021 and age-specific ASPR, ASYR, and number in 2021 for HHD, IHD, and RHD. ASPR, age-standardized prevalence rate; ASYR, age-standardized YLD rate; EAPC, estimated annual percentage change; IHD, ischemic heart disease; HHD, hypertensive heart disease; RHD, rheumatic heart disease; SDI, sociodemographic index; GBD, Global Burden of Disease.

Globally, severe heart failure (SHF) due to HHD accounted for 4,073,302 prevalent cases in 2021 (ASPR: 48.312 per 100,000; EAPC = 0.56), an increase of 170.3% from 1990. Females had a higher absolute burden (2,216,111 cases; ASPR: 47.767; EAPC = 0.68) than males (1,857,191 cases; ASPR: 48.487; EAPC = 0.38). SHF-related YLDs reached 700,272 globally (ASYR: 8.299; EAPC = 0.57), slightly higher among males (ASYR: 8.346; 320,450 YLDs; EAPC = 0.40) compared to females (ASYR: 8.190; 379,822 YLDs; EAPC = 0.69). Trends from 1990 to 2021 in ASRs were consistent across sexes (Tables 1, 2, Supplementary Tables S1, S2 and Figure 1).

**Table 1:** The prevalence of SHF due to HHD in 1990 and 2021.

Locations	Cause	Sex	1990 prevalence cases (95% UI)	2021 prevalence cases (95% UI)	1990 ASPR (95% UI)	2021 ASPR (95% UI)	1990–2021 EAPC (95%CI)	1990–2021 prevalence cases changes (%)
Global	Hypertensive heart disease	Both	1,506,979 (1,161,737–1,933,553)	4,073,302 (3,078,489–5,324,403)	40.859 (31.301–52.529)	48.312 (36.774–62.917)	0.56 (0.52–0.59)	170.296
High SDI	Hypertensive heart disease	Both	275,424 (208,999–358,970)	793,747 (607,525–1,034,136)	25.133 (19.43–32.284)	36.93 (28.819–47.203)	1.57 (1.46–1.68)	188.191
High-middle SDI	Hypertensive heart disease	Both	323,219 (240,938–417,762)	892,801 (662,261–1,174,875)	35.081 (26.276–45.415)	45.554 (34.187–59.712)	0.87 (0.83–0.92)	176.222
Low SDI	Hypertensive heart disease	Both	122,235 (92,366–157,951)	298,258 (226,400–382,208)	65.885 (49.281–85.465)	67.884 (50.825–89.191)	0.12 (0.09–0.14)	144.004
Low-middle SDI	Hypertensive heart disease	Both	241,675 (188,511–305,778)	632,843 (479,532–812,356)	47.399 (37.051–60.286)	49.334 (37.576–63.682)	0.08 (0.07–0.1)	161.857
Middle SDI	Hypertensive heart disease	Both	542,837 (424,289–691,330)	1,451,596 (1,078,299–1,912,457)	61.844 (47.752–79.376)	57.998 (43.505–76.028)	−0.35 (−0.47 to −0.23)	167.409
Andean Latin America	Hypertensive heart disease	Both	9,607 (7,521–12,223)	27,997 (21,092–36,725)	48.937 (37.783–63.285)	48.335 (36.353–63.816)	0.17 (0.07–0.27)	191.423
Australasia	Hypertensive heart disease	Both	2,374 (1,805–3,068)	9,790 (7,649–12,310)	10.221 (7.811–13.143)	17.073 (13.402–21.296)	1.95 (1.83–2.06)	312.384
Caribbean	Hypertensive heart disease	Both	11,731 (9,053–15,284)	34,276 (26,011–45,140)	46.089 (35.643–60.327)	63.657 (48.306–83.933)	1.24 (1.17–1.3)	192.183
Central Asia	Hypertensive heart disease	Both	11,437 (8,393–15,531)	22,586 (15,244–30,831)	26.089 (18.889–35.855)	31.118 (20.735–43.166)	0.95 (0.74–1.16)	97.482
Central Europe	Hypertensive heart disease	Both	46,482 (33,953–62,654)	110,467 (79,889–146,524)	32.659 (24.017–43.471)	47.551 (35.01–62.344)	1.7 (1.55–1.84)	137.655
Central Latin America	Hypertensive heart disease	Both	33,590 (25,977–42,770)	92,673 (69,294–121,504)	44.567 (34.24–57.373)	38.754 (28.922–51.164)	−0.65 (−0.72 to −0.59)	175.895
Central Sub-Saharan Africa	Hypertensive heart disease	Both	12,626 (8,966–16,712)	34,634 (25,197–46,102)	72.175 (50.752–97.706)	77.883 (55.504–105.375)	0.28 (0.22–0.33)	174.307
East Asia	Hypertensive heart disease	Both	504,991 (377,968–654,323)	1,324,469 (959,524–1,752,486)	70.579 (53.094–90.49)	62.913 (46.746–82.941)	−0.63 (−0.85 to −0.4)	162.276
Eastern Europe	Hypertensive heart disease	Both	25,346 (17,951–34,740)	50,472 (34,371–72,036)	9.338 (6.654–12.677)	13.939 (9.575–19.736)	1.73 (1.56–1.91)	99.132
Eastern Sub-Saharan Africa	Hypertensive heart disease	Both	57,014 (41,773–74,691)	138,614 (103,620–176,601)	91.912 (68.061–121.952)	95.038 (70.589–125.487)	0.08 (0.06–0.1)	143.123
High-income Asia Pacific	Hypertensive heart disease	Both	34,240 (24,997–45,717)	96,838 (70,317–129,926)	18.702 (13.496–24.989)	18.753 (14.34–24.422)	−0.21 (−0.4 to −0.02)	182.821
High-income North America	Hypertensive heart disease	Both	103,076 (77,776–134,676)	309,288 (234,952–391,869)	29.891 (22.761–38.6)	49.089 (38.263–61.358)	1.93 (1.79–2.07)	200.058
North Africa and Middle East	Hypertensive heart disease	Both	118,908 (94,277–147,636)	334,721 (259,665–422,722)	78.004 (60.621–99.313)	79.243 (60.292–101.327)	0.13 (0.08–0.17)	181.496
Oceania	Hypertensive heart disease	Both	901 (696–1,154)	2,194 (1,698–2,797)	37.694 (28.605–49.137)	34.815 (26.246–44.726)	−0.36 (−0.42 to −0.31)	143.507
South Asia	Hypertensive heart disease	Both	153,342 (118,770–192,645)	466,689 (346,487–613,529)	33.92 (26.111–43.46)	36.202 (26.884–47.618)	0.19 (0.17–0.21)	204.345
Southeast Asia	Hypertensive heart disease	Both	120,672 (95,719–152,755)	312,751 (242,388–396,699)	54.752 (43.048–69.89)	53.198 (41.106–68.691)	−0.19 (−0.23 to −0.14)	159.174
Southern Latin America	Hypertensive heart disease	Both	12,711 (9,344–17,112)	32,189 (22,432–43,374)	28.695 (21.155–38.29)	35.865 (25.334–47.929)	0.98 (0.92–1.03)	153.237
Southern Sub-Saharan Africa	Hypertensive heart disease	Both	15,635 (11,316–20,350)	35,176 (26,033–45,785)	64.103 (47.337–85.173)	68.6 (51.068–90.444)	0.24 (0.19–0.3)	124.982
Tropical Latin America	Hypertensive heart disease	Both	39,985 (31,018–50,768)	133,972 (100,547–177,427)	48.838 (37.747–62.345)	54.078 (40.721–71.847)	0.32 (0.29–0.36)	235.056
Western Europe	Hypertensive heart disease	Both	128,443 (94,797–172,695)	356,698 (264,801–471,838)	21.431 (15.921–28.33)	32.851 (24.752–42.65)	2.02 (1.71–2.33)	177.709
Western Sub-Saharan Africa	Hypertensive heart disease	Both	63,866 (48,160–82,425)	146,806 (112,460–187,911)	83.844 (64.012–107.663)	86.717 (65.99–112.424)	0.14 (0.1–0.19)	129.866

**Table 2:** The YLDs of SHF due to HHD in 1990 and 2021.

Locations	Cause	Sex	1990 YLDs cases(95% UI)	2021 YLDs cases(95% UI)	1990 ASYR(95% UI)	2021 ASYR(95% UI)	1990−2021 EAPC (95%CI)	1990–2021 YLDs cases changes
Global	Hypertensive heart disease	Both	259,063 (164,169–376,322)	700,272 (440,202–1,028,642)	6.998 (4.42–10.171)	8.299 (5.205–12.121)	0.57 (0.53–0.61)	170.31
High SDI	Hypertensive heart disease	Both	47,887 (29,448–70,046)	137,579 (84,087–203,139)	4.37 (2.706–6.367)	6.425 (4.014–9.342)	1.57 (1.46–1.68)	187.299
High-middle SDI	Hypertensive heart disease	Both	55,736 (33,935–82,708)	153,745 (94,369–226,853)	6.031 (3.706–8.872)	7.843 (4.818–11.524)	0.88 (0.84–0.92)	175.845
Low SDI	Hypertensive heart disease	Both	20,861 (13,261–30,428)	51,110 (33,181–74,639)	11.121 (7.089–16.207)	11.524 (7.409–16.914)	0.13 (0.11–0.16)	145.003
Low-middle SDI	Hypertensive heart disease	Both	41,104 (26,229–60,167)	108,037 (68,843–158,662)	7.984 (5.107–11.492)	8.371 (5.277–12.184)	0.11 (0.09–0.12)	162.838
Middle SDI	Hypertensive heart disease	Both	93,199 (58,090–136,290)	249,101 (155,822–368,407)	10.543 (6.577–15.364)	9.925 (6.197–14.562)	−0.34 (−0.46 to −0.22)	167.279
Andean Latin America	Hypertensive heart disease	Both	1,658 (1,058–2,407)	4,838 (2,989–7,258)	8.405 (5.296–12.416)	8.341 (5.09–12.535)	0.19 (0.09–0.29)	191.797
Australasia	Hypertensive heart disease	Both	413 (247–602)	1,695 (1,032–2,462)	1.775 (1.084–2.573)	2.965 (1.803–4.342)	1.93 (1.82–2.05)	310.412
Caribbean	Hypertensive heart disease	Both	2,028 (1,291–3,005)	5,914 (3,681–8,915)	7.942 (5.045–11.703)	10.99 (6.843–16.563)	1.24 (1.18–1.3)	191.617
Central Asia	Hypertensive heart disease	Both	1,971 (1,189–2,966)	3,905 (2,294–5,981)	4.482 (2.66–6.786)	5.36 (3.055–8.227)	0.95 (0.74–1.16)	98.123
Central Europe	Hypertensive heart disease	Both	8,053 (4,706–12,061)	19,103 (11,181–28,383)	5.649 (3.351–8.33)	8.235 (4.878–12.124)	1.7 (1.55–1.85)	137.216
Central Latin America	Hypertensive heart disease	Both	5,790 (3,654–8,412)	15,946 (9,911–23,588)	7.637 (4.732–11.073)	6.66 (4.123–9.95)	−0.65 (−0.71 to −0.58)	175.406
Central Sub-Saharan Africa	Hypertensive heart disease	Both	2,177 (1,297–3,310)	5,991 (3,721–9,195)	12.316 (7.334–18.55)	13.349 (8.242–20.478)	0.29 (0.23–0.35)	175.195
East Asia	Hypertensive heart disease	Both	86,852 (52,382–128,067)	227,600 (141,048–343,854)	12.057 (7.307–17.669)	10.792 (6.678–16.119)	−0.61 (−0.83 to −0.39)	162.055
Eastern Europe	Hypertensive heart disease	Both	4,377 (2,605–6,741)	8,694 (4,914–14,059)	1.611 (0.967–2.44)	2.402 (1.369–3.87)	1.72 (1.54–1.9)	98.629
Eastern Sub-Saharan Africa	Hypertensive heart disease	Both	9,756 (6,124–14,472)	23,808 (15,363–34,959)	15.563 (9.777–22.697)	16.174 (10.426–23.744)	0.09 (0.07–0.11)	144.034
High-income Asia Pacific	Hypertensive heart disease	Both	5,971 (3,584–8,915)	16,711 (10,070–25,305)	3.251 (1.982–4.889)	3.262 (1.983–4.796)	−0.21 (−0.4 to −0.02)	179.869
High-income North America	Hypertensive heart disease	Both	17,981 (10,690–26,473)	53,804 (32,784–78,613)	5.22 (3.168–7.637)	8.563 (5.262–12.383)	1.92 (1.78–2.06)	199.227
North Africa and Middle East	Hypertensive heart disease	Both	20,467 (13,163–29,767)	57,870 (37,031–84,216)	13.308 (8.501–19.389)	13.619 (8.557–19.832)	0.15 (0.11–0.2)	182.748
Oceania	Hypertensive heart disease	Both	155 (98–231)	380 (231–565)	6.419 (4.052–9.555)	5.969 (3.558–9.179)	−0.35 (−0.41 to −0.29)	145.161
South Asia	Hypertensive heart disease	Both	25,772 (16,260–37,626)	78,804 (48,891–117,445)	5.63 (3.541–8.185)	6.07 (3.786–8.964)	0.22 (0.2–0.24)	205.774
Southeast Asia	Hypertensive heart disease	Both	20,658 (13,388–30,232)	53,686 (34,131–77,637)	9.303 (5.94–13.481)	9.088 (5.764–13.096)	−0.17 (−0.21 to −0.12)	159.88
Southern Latin America	Hypertensive heart disease	Both	2,216 (1,328–3,376)	5,578 (3,220–8,388)	4.988 (3.009–7.597)	6.221 (3.618–9.353)	0.98 (0.92–1.04)	151.715
Southern Sub-Saharan Africa	Hypertensive heart disease	Both	2,690 (1,691–4,020)	6,076 (3,785–9,075)	10.98 (6.806–16.376)	11.794 (7.3–17.603)	0.25 (0.2–0.31)	125.874
Tropical Latin America	Hypertensive heart disease	Both	6,872 (4,354–9,947)	22,973 (13,977–34,164)	8.335 (5.199–12.052)	9.263 (5.614–13.719)	0.32 (0.29–0.36)	234.299
Western Europe	Hypertensive heart disease	Both	22,279 (13,196–33,322)	61,628 (36,880–90,733)	3.717 (2.239–5.53)	5.701 (3.413–8.3)	2.02 (1.71–2.33)	176.619
Western Sub-Saharan Africa	Hypertensive heart disease	Both	10,925 (6,898–15,826)	25,271 (16,349–37,005)	14.231 (9.219–20.502)	14.807 (9.579–21.567)	0.16 (0.12–0.2)	131.314

**Figure 2:**
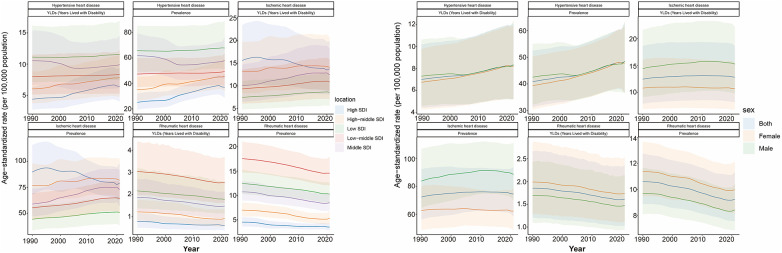
Trends in ASPR and ASYR of HHD, IHD, and RHD by global, sex, and SDI regions, 1990–2021. ASPR, age-standardized prevalence rate; ASYR, age-standardized YLD rate; SDI, sociodemographic index.

SHF burden due to HHD peaked in the 70–74 age group. ASRs increased progressively with age, reaching maximum levels in individuals aged ≥90 years, especially among females. Low SDI regions had the highest standardized SHF burden (ASPR: 67.884; EAPC = 0.12; ASYR: 11.524; EAPC = 0.13), while Middle SDI regions had the highest absolute burden (1,451,596 cases; 249,101 YLDs). Notably, ASPR and ASYR showed rising trends predominantly in High and High-middle SDI regions, with declines only observed in Middle SDI regions (Tables 1, 2, Figure 1 and Figure 2).

**Figure 3:**
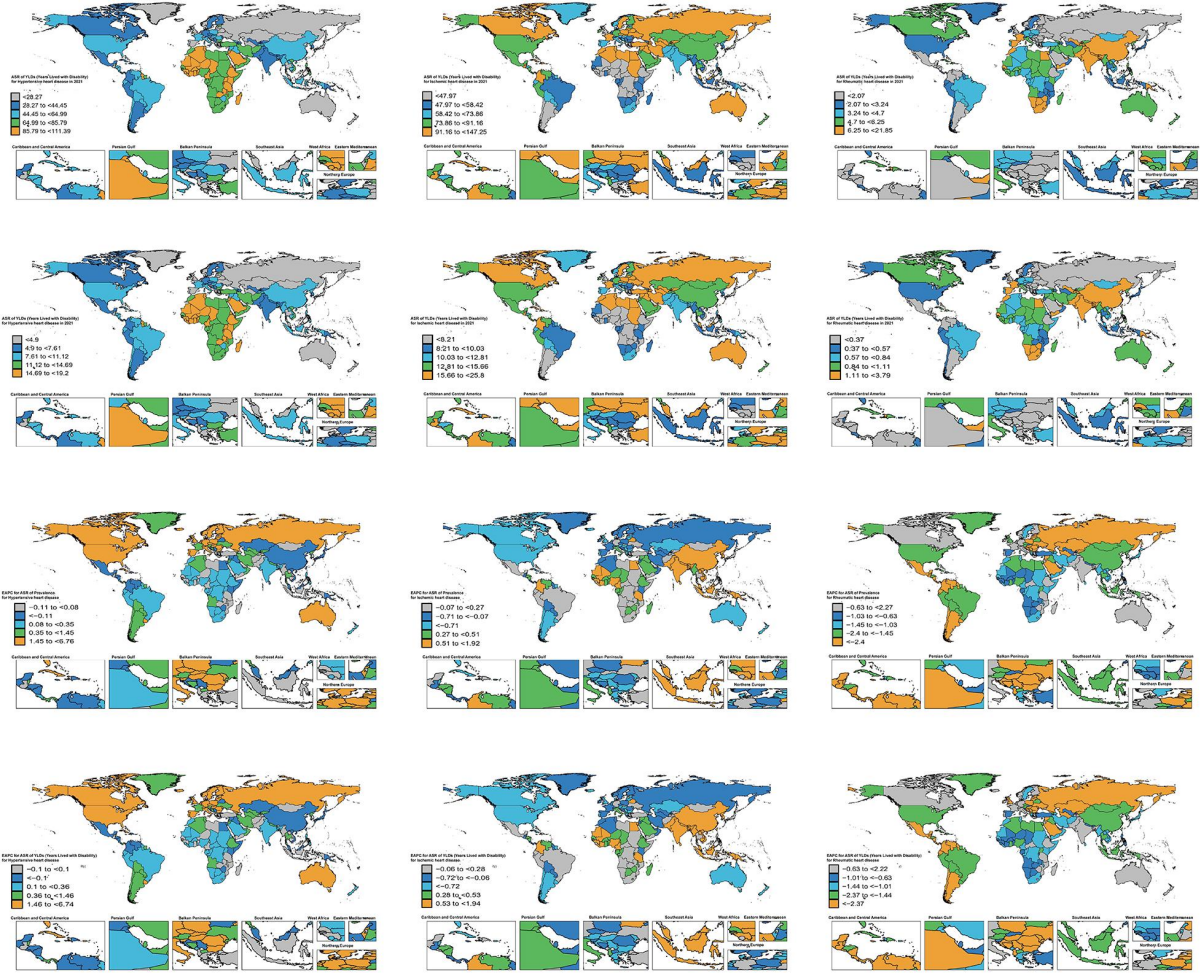
Global distribution of ASPR, ASYR, and EAPC for HHD, IHD, and RHD in 204 countries and territories, 1990–2021. ASPR, age-standardized prevalence rate; ASYR, age-standardized YLD rate; EAPC, estimated annual percentage change.

Eastern Sub-Saharan Africa had the highest standardized SHF burden due to HHD (ASPR: 95.038; ASYR: 16.174), whereas East Asia had the largest absolute burden (1,324,469 cases; 227,600 YLDs), despite lower standardized rates (ASPR: 44.308; EAPC = −0.63; ASYR: 7.573; EAPC = −0.61). At the national level, China reported the greatest absolute SHF burden (1,274,212 cases; 218,890 YLDs), followed by India and the United States of America. Jordan had the highest national ASPR (111.388) and ASYR (19.201). Latvia showed the steepest rise (ASPR EAPC = 6.76; ASYR EAPC = 6.74), while Belarus experienced the sharpest decline (ASPR EAPC = −3.77; ASYR EAPC = −3.74) (Tables 1, 2, Supplementary Tables S3, S4, Figures 1, 2 and Figure 3).

#### Severe heart failure due to IHD

In 2021, SHF resulting from IHD caused 6,242,655 prevalent cases worldwide (ASPR: 74.384 per 100,000; EAPC = 0.10), increasing by 140.7% since 1990. The burden was greater in males (3,351,396 cases; ASPR: 88.469; EAPC = 0.22) than females (2,891,259 cases; ASPR: 62.290; EAPC = −0.04). Global SHF-related YLDs due to IHD were 1,075,512 (ASYR: 12.803; EAPC = 0.10), higher among males (ASYR: 15.246; 579,142 YLDs; EAPC = 0.22) compared to females (ASYR: 10.699; 496,370 YLDs; EAPC = −0.05), with consistent ASR trends across sexes over the study period (Tables 3, 4, Supplementary Tables S1, S2 and Figure 1).

**Table 3:** The prevalence of SHF due–IHD in 1990 and 2021.

Locations	Cause	Sex	1990 prevalence cases(95% UI)	2021 prevalence cases(95% UI)	1990 ASPR(95% UI)	2021 ASPR(95% UI)	1990–2021 EAPC (95%CI)	1990–2021 prevalence cases changes(%)
Global	Ischemic heart disease	Both	2,593,437 (2,014,066–3,290,834)	6,242,655 (4,915,567–7,878,649)	72.303 (56.299–92.296)	74.384 (58.582–93.602)	0.1 (0.07–0.14)	140.71
High SDI	Ischemic heart disease	Both	995,638 (746,571–1,288,470)	1,758,646 (1,375,180–2,215,851)	89.604 (67.644–115.61)	78.99 (62.727–98.248)	−0.62 (−0.7 to −0.53)	76.635
High-middle SDI	Ischemic heart disease	Both	690,009 (532,479–878,149)	1,594,270 (1,254,418–2,010,107)	76.767 (59.742–97.816)	81.651 (64.212–102.377)	0.31 (0.26–0.37)	131.051
Low SDI	Ischemic heart disease	Both	83,741 (65,317–106,164)	226,042 (177,077–285,212)	44.228 (33.561–57.049)	50.7 (38.537–65.656)	0.5 (0.48–0.53)	169.93
Low-middle SDI	Ischemic heart disease	Both	299,874 (244,407–366,079)	845,700 (669,223–1,054,400)	55.213 (43.888–69.18)	63.875 (50.267–80.518)	0.57 (0.54–0.61)	182.018
Middle SDI	Ischemic heart disease	Both	520,363 (417,646–642,850)	1,811,640 (1,414,550–2,270,582)	58.809 (46.768–73.574)	72.816 (56.589–91.51)	0.9 (0.83–0.97)	248.149
Andean Latin America	Ischemic heart disease	Both	14,563 (11,517–18,390)	45,796 (35,593–58,975)	74.263 (57.814–96.136)	79.003 (60.568–102.692)	−0.01 (−0.14–0.13)	214.468
Australasia	Ischemic heart disease	Both	29,586 (22,588–38,530)	57,763 (45,201–73,066)	125.113 (95.911–163.056)	100.069 (79.246–125.703)	−1.36 (−1.58 to −1.14)	95.238
Caribbean	Ischemic heart disease	Both	26,360 (20,329–34,589)	45,117 (34,582–59,742)	106.075 (81.185–139.721)	83.37 (63.898–109.781)	−0.91 (−0.97 to −0.86)	71.157
Central Asia	Ischemic heart disease	Both	45,609 (33,065–61,458)	64,662 (46,539–86,450)	107.439 (76.449–146.041)	93.277 (65.834–127.075)	−0.65 (−0.75 to −0.55)	41.775
Central Europe	Ischemic heart disease	Both	134,701 (101,530–175,089)	205,971 (160,176–260,835)	97.442 (74.555–126.954)	87.903 (69.141–110.901)	−0.4 (−0.47 to −0.33)	52.91
Central Latin America	Ischemic heart disease	Both	62,986 (50,962–77,582)	212,632 (167,079–265,563)	81.046 (64.29–101.615)	88.053 (69.027–110.686)	0.31 (0.28–0.35)	237.586
Central Sub-Saharan Africa	Ischemic heart disease	Both	7,359 (5,248–10,003)	19,852 (14,516–26,285)	44.638 (31.175–61.938)	46.618 (33.785–63.698)	0.07 (0.05–0.09)	169.765
East Asia	Ischemic heart disease	Both	344,281 (265,344–441,136)	1,538,293 (1,171,146–1,973,700)	47.903 (37.166–61.622)	74.422 (56.939–94.8)	1.87 (1.68–2.06)	346.813
Eastern Europe	Ischemic heart disease	Both	284,752 (220,716–361,262)	373,085 (289,689–478,798)	110.438 (85.632–140.409)	102.904 (80.061–131.81)	−0.21 (−0.28 to −0.14)	31.021
Eastern Sub-Saharan Africa	Ischemic heart disease	Both	23,974 (17,615–32,275)	63,142 (46,771–84,137)	40.709 (29.269–56.547)	45.016 (32.745–61.157)	0.3 (0.26–0.34)	163.377
High-income Asia Pacific	Ischemic heart disease	Both	73,129 (52,323–95,730)	202,625 (152,245–258,790)	39.084 (27.955–51.517)	42.722 (33.72–53.524)	0.23 (0.11–0.35)	177.079
High-income North America	Ischemic heart disease	Both	388,394 (292,040–497,290)	630,057 (488,452–789,134)	107.601 (81.923–136.078)	91.942 (72.052–114.772)	−0.87 (−0.97 to −0.76)	62.221
North Africa and Middle East	Ischemic heart disease	Both	157,852 (127,782–194,866)	450,387 (361,175–561,053)	97.209 (77.061–122.203)	103.137 (81.493–129.339)	0.15 (0.1–0.2)	185.322
Oceania	Ischemic heart disease	Both	1,335 (1,045–1,697)	4,045 (3,208–5,156)	59.284 (45.873–76.694)	69.215 (53.704–90.454)	0.54 (0.5–0.57)	202.996
South Asia	Ischemic heart disease	Both	291,010 (236,771–351,108)	945,521 (749,832–1,165,943)	56.69 (45.878–69.885)	69.179 (54.752–85.642)	0.8 (0.75–0.85)	224.91
Southeast Asia	Ischemic heart disease	Both	116,413 (93,623–146,374)	355,497 (280,008–449,660)	52.682 (41.366–67.07)	62.234 (48.921–78.939)	0.66 (0.62–0.69)	205.376
Southern Latin America	Ischemic heart disease	Both	21,496 (15,875–28,290)	35,284 (25,050–47,276)	49.436 (36.442–65.942)	39.946 (28.604–53.039)	−0.88 (−0.97 to −0.79)	64.142
Southern Sub-Saharan Africa	Ischemic heart disease	Both	13,896 (10,481–17,956)	29,504 (22,541–38,353)	58.337 (43.707–76.016)	59.035 (44.535–77.846)	0.06 (−0.02–0.14)	112.32
Tropical Latin America	Ischemic heart disease	Both	47,598 (38,029–58,695)	140,828 (109,235–178,857)	58.507 (45.836–73.262)	56.305 (43.761–71.734)	−0.02 (−0.07–0.04)	195.87
Western Europe	Ischemic heart disease	Both	484,688 (358,116–635,335)	758,308 (578,046–979,903)	82.257 (62.035–107.713)	73.69 (57.546–95.007)	−0.53 (−0.7 to −0.37)	56.453
Western Sub-Saharan Africa	Ischemic heart disease	Both	23,455 (17,163–31,141)	64,287 (47,650–84,397)	31.459 (22.601–42.764)	38.876 (28.22–52.462)	0.74 (0.71–0.76)	174.087

**Table 4:** The YLDs of SHF due–IHD in 1990 and 2021.

Locations	cause	sex	1990 YLDs cases(95% UI)	2021 YLDs cases(95% UI)	1990 ASYR(95% UI)	2021 ASYR(95% UI)	1990–2021 EAPC (95%CI)	1990–2021 YLDs cases changes
Global	Ischemic heart disease	Both	448,419 (283,192–662,990)	1,075,512 (683,301–1,576,580)	12.449 (7.865–18.59)	12.803 (8.179–18.638)	0.1 (0.07–0.14)	139.845
High SDI	Ischemic heart disease	Both	173,387 (109,050–261,732)	304,914 (195,771–442,525)	15.602 (9.872–23.391)	13.739 (8.864–19.843)	−0.62 (−0.7 to −0.53)	75.857
High-middle SDI	Ischemic heart disease	Both	119,283 (74,658–176,817)	274,875 (174,175–406,314)	13.216 (8.362–19.722)	14.077 (8.964–20.7)	0.32 (0.26–0.37)	130.439
Low SDI	Ischemic heart disease	Both	14,294 (9,128–21,077)	38,643 (25,560–57,384)	7.449 (4.756–11.076)	8.575 (5.573–12.608)	0.52 (0.49–0.54)	170.344
Low-middle SDI	Ischemic heart disease	Both	51,215 (33,613–74,529)	144,714 (93,296–213,336)	9.324 (6.058–13.599)	10.857 (7.054–15.879)	0.59 (0.56–0.63)	182.562
Middle SDI	Ischemic heart disease	Both	89,579 (58,533–130,763)	311,268 (198,445–460,743)	10.021 (6.521–14.617)	12.468 (7.959–18.239)	0.92 (0.85–0.99)	247.479
Andean Latin America	Ischemic heart disease	Both	2,511 (1,570–3,708)	7,913 (4,992–11,937)	12.737 (7.907–18.739)	13.63 (8.614–20.61)	0.02 (−0.12–0.16)	215.133
Australasia	Ischemic heart disease	Both	5,142 (3,148–7,703)	10,017 (6,359–14,879)	21.721 (13.518–32.219)	17.39 (11.131–25.73)	−1.36 (−1.59 to −1.14)	94.807
Caribbean	Ischemic heart disease	Both	4,549 (2,797–6,862)	7,774 (4,898–11,588)	18.251 (11.18–27.636)	14.376 (9.018–21.455)	−0.91 (−0.96 to −0.86)	70.895
Central Asia	Ischemic heart disease	Both	7,864 (4,770–12,086)	11,179 (6,551–17,554)	18.463 (11.104–28.365)	16.058 (9.14–24.99)	−0.64 (−0.74 to −0.54)	42.154
Central Europe	Ischemic heart disease	Both	23,369 (14,334–35,047)	35,634 (22,390–52,961)	16.863 (10.468–25.258)	15.228 (9.645–22.569)	−0.4 (−0.47 to −0.32)	52.484
Central Latin America	Ischemic heart disease	Both	10,886 (7,206–15,703)	36,667 (23,682–54,176)	13.911 (8.831–20.557)	15.162 (9.712–22.361)	0.32 (0.29–0.36)	236.827
Central Sub-Saharan Africa	Ischemic heart disease	Both	1,268 (762–1,996)	3,424 (2,100–5,342)	7.593 (4.657–11.872)	7.964 (4.898–12.304)	0.08 (0.06–0.11)	170.032
East Asia	Ischemic heart disease	Both	59,410 (37,771–87,850)	264,495 (167,320–394,035)	8.192 (5.268–12.3)	12.773 (8.163–18.931)	1.89 (1.7–2.08)	345.203
Eastern Europe	Ischemic heart disease	Both	49,086 (30,884–72,305)	64,250 (40,523–95,967)	18.988 (12.186–28.054)	17.723 (11.229–26.466)	−0.21 (−0.28 to −0.14)	30.893
Eastern Sub-Saharan Africa	Ischemic heart disease	Both	4,103 (2,517–6,423)	10,840 (6,823–16,397)	6.88 (4.388–10.591)	7.645 (4.85–11.928)	0.32 (0.28–0.36)	164.197
High-income Asia Pacific	Ischemic heart disease	Both	12,785 (7,963–19,820)	35,120 (22,805–51,064)	6.81 (4.26–10.422)	7.461 (4.925–11.049)	0.24 (0.12–0.37)	174.697
High-income North America	Ischemic heart disease	Both	67,625 (42,025–101,632)	109,239 (69,989–156,574)	18.749 (11.801–28.047)	15.969 (10.27–23.035)	−0.88 (−0.98 to −0.78)	61.536
North Africa and Middle East	Ischemic heart disease	Both	27,307 (17,919–39,850)	78,167 (50,327–114,200)	16.66 (10.708–24.375)	17.776 (11.652–25.987)	0.17 (0.12–0.21)	186.253
Oceania	Ischemic heart disease	Both	231 (146–343)	697 (444–1,031)	10.108 (6.363–15.042)	11.809 (7.63–17.621)	0.54 (0.51–0.58)	201.732
South Asia	Ischemic heart disease	Both	49,437 (32,606–71,498)	160,878 (106,309–236,348)	9.495 (6.253–13.744)	11.683 (7.598–16.981)	0.83 (0.78–0.88)	225.42
Southeast Asia	Ischemic heart disease	Both	20,002 (12,973–29,336)	61,101 (39,866–90,537)	8.956 (5.632–13.344)	10.633 (6.843–15.802)	0.68 (0.64–0.71)	205.474
Southern Latin America	Ischemic heart disease	Both	3,734 (2,252–5,629)	6,132 (3,654–9,398)	8.563 (5.225–12.853)	6.95 (4.181–10.622)	−0.87 (−0.97 to −0.78)	64.221
Southern Sub-Saharan Africa	Ischemic heart disease	Both	2,388 (1,534–3,568)	5,090 (3,289–7,657)	9.974 (6.415–15.056)	10.128 (6.54–15.245)	0.07 (−0.01–0.15)	113.149
Tropical Latin America	Ischemic heart disease	Both	8,188 (5,215–11,889)	24,201 (15,517–35,996)	9.99 (6.34–14.568)	9.666 (6.213–14.378)	0 (−0.06–0.05)	195.567
Western Europe	Ischemic heart disease	Both	84,516 (51,411–127,191)	131,627 (83,041–193,398)	14.348 (8.936–21.513)	12.846 (8.215–18.875)	−0.53 (−0.69 to −0.37)	55.742
Western Sub-Saharan Africa	Ischemic heart disease	Both	4,016 (2,524–6,195)	11,069 (7,009–16,795)	5.337 (3.421–8.166)	6.629 (4.219–10.218)	0.75 (0.73–0.78)	175.623

SHF due to IHD peaked in individuals aged 70–74 years, with ASRs rising progressively with advancing age, reaching highest levels among those aged ≥95 years. Females consistently exhibited lower burden than males across most age groups. High-middle SDI regions had the highest standardized burden (ASPR: 81.651; EAPC = 0.31), while Middle SDI regions carried the greatest absolute burden (1,811,640 cases; 311,268 YLDs) and largest increases in ASPR and ASYR. High SDI regions uniquely showed declining trends (ASPR EAPC = −0.17; ASYR EAPC = −0.16) (Tables 3, 4, Figures 1, 2).

At the super-region level, North Africa and the Middle East had the highest standardized SHF burden due to IHD (ASPR: 103.137; ASYR: 17.776). China had the largest national absolute burden (1,494,576 cases; 256,916 YLDs), followed by India and the United States of America. Sweden recorded the highest national ASPR (147.253) and ASYR (25.803). Between 1990 and 2021, China had the steepest increases in both ASPR (EAPC = 1.92) and ASYR (EAPC = 1.94), while Georgia showed the largest declines (ASPR EAPC = −3.77; ASYR EAPC = −3.76) (Tables 3, 4, Supplementary Tables S5, S6, Figures 1–3).

#### Severe heart failure due to RHD

Globally, SHF due to RHD caused 740,018 prevalent cases in 2021 (ASPR: 9.237 per 100,000; EAPC = −0.53), increasing by 38.95% since 1990. Females had higher absolute burden (412,024 cases; ASPR: 9.971; EAPC = −0.51) compared to males (327,994 cases; ASPR: 8.412; EAPC = −0.56). SHF-related YLDs reached 129,155 (ASYR: 1.613; EAPC = −0.53), higher among females (ASYR: 1.737; 71,664 YLDs; EAPC = −0.52) than males (ASYR: 1.474; 57,491 YLDs; EAPC = −0.55). Trends in ASRs between sexes were similar over the study period (Tables 5, 6, Supplementary Tables S1, S2 and Figure 1).

**Table 5:** The prevalence of SHF due–RHD in 1990 and 2021.

Locations	Cause	Sex	1990 prevalence cases(95% UI)	2021 prevalence cases(95% UI)	1990 ASPR(95% UI)	2021 ASPR(95% UI)	1990–2021 EAPC (95%CI)	1990–2021 prevalence cases changes(%)
Global	Rheumatic heart disease	Both	532,566 (441,502–641,872)	740,018 (605,227–906,872)	10.603 (8.84–12.778)	9.237 (7.527–11.336)	−0.53 (−0.56 to −0.5)	38.953
High SDI	Rheumatic heart disease	Both	46,739 (36,788–58,468)	64,824 (50,144–81,826)	4.493 (3.592–5.531)	3.48 (2.826–4.25)	−0.85 (−0.96 to −0.75)	38.694
High-middle SDI	Rheumatic heart disease	Both	70,650 (57,105–86,672)	91,781 (69,030–120,083)	6.954 (5.648–8.535)	5.262 (4.142–6.747)	−1.19 (−1.28 to −1.1)	29.909
Low SDI	Rheumatic heart disease	Both	59,952 (48,910–74,989)	104,948 (84,367–131,020)	12.478 (10.278–15.256)	10.259 (8.36–12.631)	−0.63 (−0.65 to −0.6)	75.053
Low-middle SDI	Rheumatic heart disease	Both	192,636 (157,898–234,773)	268,803 (222,023–330,835)	17.559 (14.69–21.066)	14.581 (12.017–17.912)	−0.68 (−0.71 to −0.64)	39.539
Middle SDI	Rheumatic heart disease	Both	162,209 (134,497–195,540)	209,309 (166,018–257,403)	10.729 (8.923–12.893)	8.49 (6.836–10.442)	−0.87 (−0.91 to −0.84)	29.037
Andean Latin America	Rheumatic heart disease	Both	1,655 (1,329–2,048)	1,818 (1,477–2,237)	5.064 (4.12–6.177)	2.868 (2.328–3.518)	−1.93 (−2.01 to −1.86)	9.849
Australasia	Rheumatic heart disease	Both	1,211 (979–1,462)	2,469 (2,002–2,996)	5.519 (4.474–6.561)	5.633 (4.619–6.683)	0.19 (0.04–0.35)	103.881
Caribbean	Rheumatic heart disease	Both	3,089 (2,404–3,987)	2,562 (2,011–3,213)	8.213 (6.449–10.498)	5.633 (4.389–7.099)	−1.45 (−1.53 to −1.36)	−17.061
Central Asia	Rheumatic heart disease	Both	6,655 (5,290–8,379)	4,863 (3,833–5,988)	9.114 (7.272–11.203)	5.363 (4.225–6.652)	−1.82 (−1.95 to −1.68)	−26.927
Central Europe	Rheumatic heart disease	Both	8,217 (6,543–10,246)	4,902 (3,762–6,375)	5.916 (4.783–7.31)	2.77 (2.258–3.425)	−2.65 (−2.89 to −2.4)	−40.343
Central Latin America	Rheumatic heart disease	Both	8,619 (7,193–10,493)	3,831 (3,035–4,805)	5.879 (4.918–7.054)	1.519 (1.202–1.907)	−4.94 (−5.12 to −4.75)	−55.552
Central Sub-Saharan Africa	Rheumatic heart disease	Both	4,051 (2,767–5,793)	6,873 (4,575–9,946)	6.181 (4.359–8.643)	4.537 (3.105–6.318)	−1.14 (−1.21 to −1.07)	69.662
East Asia	Rheumatic heart disease	Both	104,756 (84,302–128,381)	154,517 (112,249–205,189)	11.092 (8.855–13.439)	7.52 (5.717–9.77)	−1.57 (−1.68 to −1.46)	47.502
Eastern Europe	Rheumatic heart disease	Both	10,186 (7,899–13,053)	4,395 (3,035–6,200)	4.128 (3.285–5.233)	1.476 (1.088–1.954)	−3.82 (−4.1 to −3.53)	−56.853
Eastern Sub-Saharan Africa	Rheumatic heart disease	Both	9,455 (6,618–13,139)	14,736 (10,159–20,296)	4.369 (3.178–5.83)	3.342 (2.427–4.507)	−0.93 (−0.97 to −0.89)	55.854
High-income Asia Pacific	Rheumatic heart disease	Both	5,544 (4,030–7,268)	8,887 (6,456–11,498)	2.974 (2.205–3.86)	2.171 (1.713–2.693)	−1.17 (−1.26 to −1.08)	60.299
High-income North America	Rheumatic heart disease	Both	13,513 (10,357–17,415)	15,341 (11,542–19,703)	4.112 (3.218–5.206)	2.767 (2.151–3.451)	−1.37 (−1.76 to −0.97)	13.528
North Africa and Middle East	Rheumatic heart disease	Both	29,315 (23,249–37,317)	31,946 (25,675–39,845)	7.735 (6.279–9.628)	5.281 (4.26–6.517)	−1.43 (−1.5 to −1.36)	8.975
Oceania	Rheumatic heart disease	Both	951 (738–1,228)	1,778 (1,382–2,265)	15.192 (12.257–18.658)	13.281 (10.746–16.424)	−0.52 (−0.55 to −0.49)	86.961
South Asia	Rheumatic heart disease	Both	240,080 (196,850–292,317)	376,330 (310,054–462,471)	23.73 (19.848–28.641)	21.01 (17.409–25.693)	−0.45 (−0.47 to −0.42)	56.752
Southeast Asia	Rheumatic heart disease	Both	27,912 (22,869–34,330)	25,772 (21,274–31,563)	5.5 (4.57–6.581)	3.883 (3.199–4.746)	−1.35 (−1.42 to −1.28)	−7.667
Southern Latin America	Rheumatic heart disease	Both	2,412 (1,942–3,000)	1,480 (1,141–1,871)	5.06 (4.059–6.276)	1.87 (1.466–2.32)	−3.77 (−4.1 to −3.44)	−38.64
Southern Sub-Saharan Africa	Rheumatic heart disease	Both	5,974 (4,389–7,918)	6,333 (4,505–8,548)	9.422 (7.092–12.326)	7.668 (5.581–10.302)	−0.69 (−0.74 to −0.65)	6.009
Tropical Latin America	Rheumatic heart disease	Both	10,620 (8,227–13,696)	8,267 (6,408–10,436)	6.461 (5.138–8.121)	3.704 (2.866–4.664)	−2.05 (−2.12 to −1.98)	−22.156
Western Europe	Rheumatic heart disease	Both	24,518 (19,222–31,117)	40,030 (31,380–49,575)	4.566 (3.661–5.68)	4.549 (3.674–5.561)	0.13 (0.05–0.2)	63.268
Western Sub-Saharan Africa	Rheumatic heart disease	Both	13,832 (9,907–18,800)	22,889 (15,911–31,751)	6.275 (4.63–8.405)	4.304 (3.11–5.742)	−1.4 (−1.48 to −1.33)	65.479

**Table 6:** The YLDs of SHF due–RHD in 1990 and 2021.

Locations	Cause	Sex	1990 YLDs cases(95% UI)	2021 YLDs cases(95% UI)	1990 ASYR(95% UI)	2021 ASYR(95% UI)	1990–2021 EAPC (95%CI)	1990–2021 YLDs cases changes
Global	Rheumatic heart disease	Both	93,234 (60,128–135,172)	129,155 (82,012–188,537)	1.851 (1.21–2.664)	1.613 (1.026–2.357)	−0.53 (−0.56 to −0.5)	38.528
High SDI	Rheumatic heart disease	Both	8,200 (5,150–12,419)	11,300 (7,144–16,902)	0.79 (0.501–1.163)	0.611 (0.389–0.891)	−0.86 (−0.97 to −0.76)	37.805
High-middle SDI	Rheumatic heart disease	Both	12,401 (7,993–17,825)	15,978 (9,648–24,251)	1.218 (0.789–1.749)	0.919 (0.57–1.377)	−1.2 (−1.29 to −1.11)	28.844
Low SDI	Rheumatic heart disease	Both	10,477 (6,672–15,321)	18,396 (11,667–27,671)	2.167 (1.377–3.182)	1.784 (1.127–2.596)	−0.62 (−0.64 to −0.59)	75.585
Low-middle SDI	Rheumatic heart disease	Both	33,589 (21,784–48,698)	46,913 (29,707–68,432)	3.042 (1.968–4.393)	2.535 (1.609–3.694)	−0.67 (−0.7 to −0.63)	39.668
Middle SDI	Rheumatic heart disease	Both	28,501 (18,337–41,156)	36,506 (22,975–53,040)	1.874 (1.211–2.713)	1.481 (0.938–2.136)	−0.88 (−0.91 to −0.84)	28.087
Andean Latin America	Rheumatic heart disease	Both	294 (189–432)	323 (205–473)	0.893 (0.581–1.288)	0.508 (0.323–0.746)	−1.93 (−2 to −1.85)	9.864
Australasia	Rheumatic heart disease	Both	213 (136–311)	432 (268–656)	0.973 (0.622–1.404)	0.992 (0.63–1.458)	0.17 (0.02–0.33)	102.817
Caribbean	Rheumatic heart disease	Both	545 (340–823)	456 (290–695)	1.45 (0.912–2.194)	1.001 (0.631–1.548)	−1.43 (−1.52 to −1.35)	−16.33
Central Asia	Rheumatic heart disease	Both	1,174 (731–1,761)	868 (528–1,282)	1.607 (1.022–2.375)	0.955 (0.576–1.415)	−1.79 (−1.92 to −1.66)	−26.065
Central Europe	Rheumatic heart disease	Both	1,446 (920–2,146)	858 (533–1,297)	1.042 (0.665–1.537)	0.488 (0.308–0.718)	−2.65 (−2.9 to −2.4)	−40.664
Central Latin America	Rheumatic heart disease	Both	1,531 (985–2,193)	681 (431–1,005)	1.04 (0.661–1.473)	0.27 (0.17–0.4)	−4.92 (−5.1 to −4.73)	−55.519
Central Sub-Saharan Africa	Rheumatic heart disease	Both	712 (409–1,137)	1,226 (706–1,940)	1.089 (0.624–1.715)	0.809 (0.48–1.271)	−1.11 (−1.19 to −1.04)	72.191
East Asia	Rheumatic heart disease	Both	18,314 (11,782–26,799)	26,732 (15,864–40,910)	1.926 (1.265–2.837)	1.304 (0.787–1.96)	−1.57 (−1.68 to −1.46)	45.965
Eastern Europe	Rheumatic heart disease	Both	1,802 (1,132–2,652)	774 (437–1,241)	0.732 (0.47–1.076)	0.261 (0.154–0.405)	−3.82 (−4.1 to −3.55)	−57.048
Eastern Sub-Saharan Africa	Rheumatic heart disease	Both	1,676 (979–2,565)	2,629 (1,537–4,073)	0.774 (0.47–1.184)	0.595 (0.36–0.92)	−0.91 (−0.95 to −0.88)	56.862
High-income Asia Pacific	Rheumatic heart disease	Both	979 (613–1,505)	1,547 (951–2,357)	0.524 (0.326–0.799)	0.382 (0.238–0.566)	−1.17 (−1.26 to −1.08)	58.018
High-income North America	Rheumatic heart disease	Both	2,378 (1,445–3,601)	2,682 (1,657–4,092)	0.725 (0.452–1.067)	0.486 (0.301–0.726)	−1.38 (−1.78 to −0.98)	12.784
North Africa and Middle East	Rheumatic heart disease	Both	5,196 (3,236–7,687)	5,688 (3,628–8,451)	1.368 (0.874–2.013)	0.939 (0.594–1.401)	−1.41 (−1.48 to −1.35)	9.469
Oceania	Rheumatic heart disease	Both	167 (105–252)	313 (184–485)	2.656 (1.711–3.869)	2.329 (1.431–3.538)	−0.51 (−0.54 to −0.49)	87.425
South Asia	Rheumatic heart disease	Both	41,754 (26,831–60,776)	65,471 (41,803–95,491)	4.097 (2.652–5.918)	3.643 (2.331–5.278)	−0.43 (−0.45 to −0.41)	56.802
Southeast Asia	Rheumatic heart disease	Both	4,955 (3,221–7,311)	4,595 (2,922–6,681)	0.976 (0.634–1.409)	0.692 (0.439–1.01)	−1.34 (−1.41 to −1.27)	−7.265
Southern Latin America	Rheumatic heart disease	Both	426 (270–637)	261 (159–395)	0.892 (0.56–1.331)	0.331 (0.201–0.495)	−3.76 (−4.09 to −3.43)	−38.732
Southern Sub-Saharan Africa	Rheumatic heart disease	Both	1,054 (628–1,646)	1,114 (646–1,749)	1.664 (1.008–2.576)	1.349 (0.787–2.108)	−0.68 (−0.73 to −0.64)	5.693
Tropical Latin America	Rheumatic heart disease	Both	1,883 (1,153–2,812)	1,464 (907–2,163)	1.144 (0.718–1.708)	0.656 (0.403–0.968)	−2.04 (−2.11 to −1.97)	−22.252
Western Europe	Rheumatic heart disease	Both	4,294 (2,609–6,493)	6,974 (4,360–10,300)	0.802 (0.498–1.183)	0.797 (0.511–1.155)	0.11 (0.04–0.19)	62.413
Western Sub-Saharan Africa	Rheumatic heart disease	Both	2,441 (1,485–3,691)	4,069 (2,407–6,277)	1.108 (0.687–1.685)	0.765 (0.459–1.18)	−1.38 (−1.46 to −1.31)	66.694

SHF burden due to RHD displayed a bimodal age distribution, peaking among individuals aged 5–29 and 60–84 years, whereas ASRs were predominantly concentrated among individuals aged ≥65 years, with relatively even distribution in elderly groups. Low-middle SDI regions had the highest standardized burden (ASPR: 14.581; EAPC = −0.68; ASYR: 2.535; EAPC = −0.67), followed by Low and Middle SDI regions. The largest absolute burden was also observed in Low-middle SDI regions (268,803 cases; 46,913 YLDs). ASPR and ASYR declined across all SDI levels, with the slowest decline in Low SDI and the most pronounced decline in High-middle SDI regions (Tables 5, 6, Figures 1, 2).

At the super-region level, South Asia reported the highest standardized SHF burden due to RHD (ASPR: 21.010; EAPC = −0.45; ASYR: 3.643; EAPC = −0.43) and the largest absolute burden (376,330 cases; 65,471 YLDs). At the national level, India had the highest absolute burden (290,436 cases; 50,498 YLDs), followed by China and Pakistan. Pakistan showed the highest national ASPR (21.855) and ASYR (3.792). Between 1990 and 2021, the Netherlands experienced the greatest increases in ASPR (EAPC = 2.27) and ASYR (EAPC = 2.22), while Guatemala exhibited the sharpest declines (ASPR EAPC = −5.81; ASYR EAPC = −5.82) (Tables 5, 6, Supplementary Tables S7, S8, Figures 1–3).

#### Correlation between SHF-related ASRs and sociodemographic development

**Figure 4:**
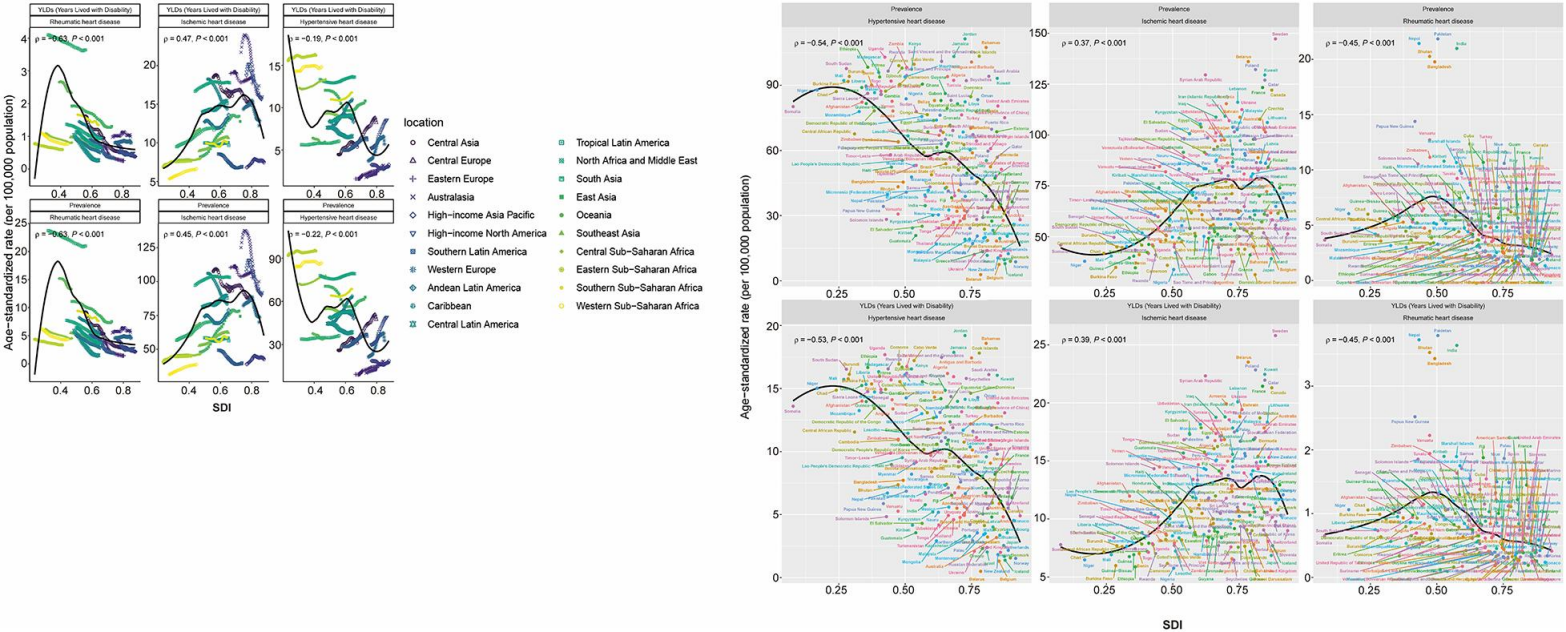
Trends in ASPR and ASYR of IHD, HHD, and RHD in 21 GBD regions by SDI from 1990 to 2021 and across countries and territories in 2021. ASPR, age-standardized prevalence rate; ASYR, age-standardized YLD rate; SDI, sociodemographic index; GBD, Global Burden of Disease.

From 1990 to 2021, ASPR and ASYR of SHF due to HHD, IHD, and RHD demonstrated distinct, non-linear relationships with SDI across the 21 GBD regions. For HHD, both ASPR and ASYR generally declined as SDI increased (ASPR: *ρ* = –0.22; ASYR: *ρ* = –0.19; *P* < 0.001), with moderate increases at SDI levels between 0.4 and 0.6, and again slightly above 0.8, indicating a bimodal distribution. In contrast, IHD-related ASPR and ASYR positively correlated with rising SDI, peaking at approximately SDI 0.75 (ASPR: *ρ* = 0.45; ASYR: *ρ* = 0.47; *P* < 0.001) and subsequently declining slightly. For RHD, ASPR and ASYR increased rapidly at lower SDI levels, peaked at around SDI∼0.4, and then decreased markedly, with the decline slowing at higher SDI levels (*ρ* = –0.63; *P* < 0.001) (Figure 4).

In 2021, similar patterns were observed across 204 countries and territories. SHF due to HHD displayed weak negative correlations with national SDI (ASPR: *ρ* = –0.45; ASYR: *ρ* = –0.45; *P* < 0.001), predominantly affecting low-SDI countries, although some high-SDI countries also exhibited high rates. SHF due to IHD positively correlated with SDI (ASPR: *ρ* = 0.37; ASYR: *ρ* = 0.39; *P* < 0.001), increasing steadily until SDI exceeded ∼0.8, followed by slight declines. SHF from RHD negatively correlated with SDI (ASPR: *ρ* = –0.54; ASYR: *ρ* = –0.53; *P* < 0.001), reaching the highest levels around SDI ∼0.5, thereafter declining with rising SDI (Figure 4).

#### Cross-country and gender inequality in SHF burden

**Figure 5:**
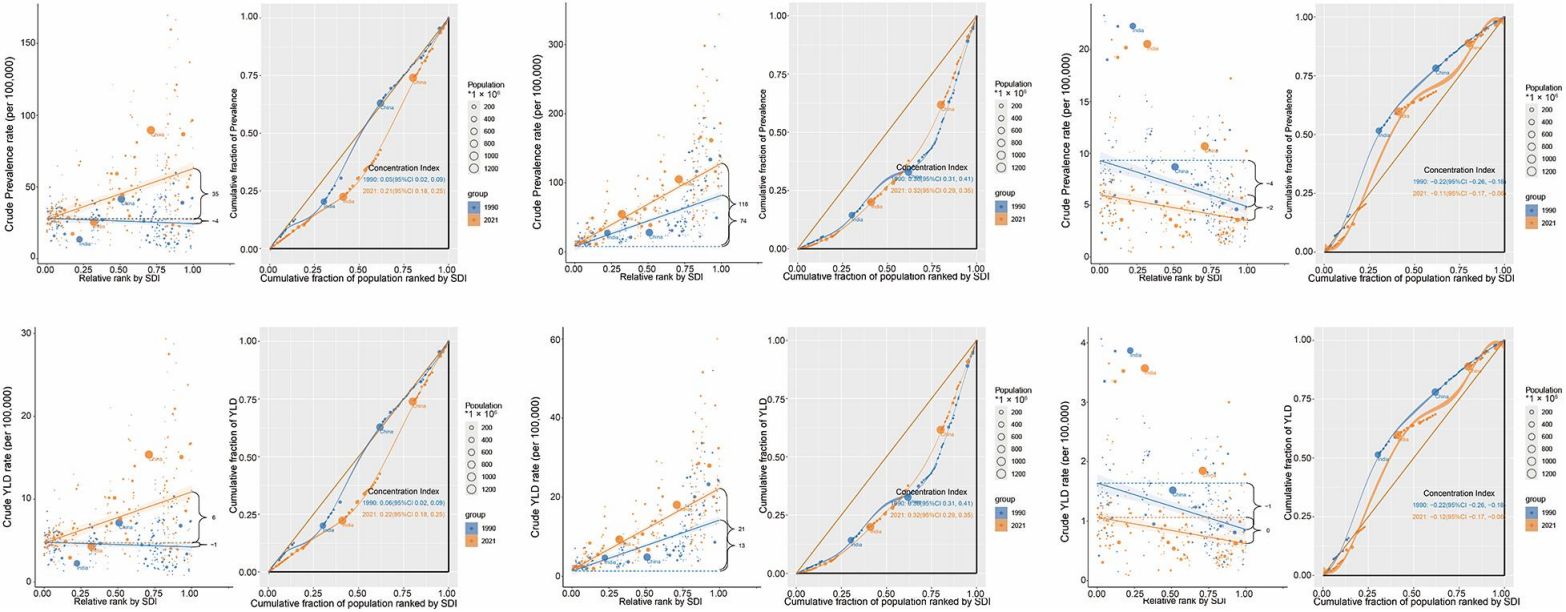
Health inequality regression and concentration curves of ASPR and ASYR for HHD, IHD, and RHD by SDI, 2021. ASPR, age-standardized prevalence rate; ASYR, age-standardized YLD rate; SDI, sociodemographic index; YLD: Years Lived with Disability.

Health inequalities in SHF due to HHD, IHD, and RHD were assessed using regression and concentration curve analyses from 1990 to 2021. For SHF due to HHD, prevalence and YLD rates increasingly concentrated in higher-SDI countries, as shown by regression trends (prevalence fitted values: −4–35; YLDs: −1–6). The CI increased notably (prevalence: 0.05–0.21; YLDs: 0.06–0.22), indicating rising inequality. India and China substantially influenced this burden. For SHF from IHD, burdens remained concentrated in high-SDI countries, with stable regression patterns (prevalence: 74–118; YLDs: 13–21). The CI slightly decreased from 0.36 to 0.32 for both indicators, reflecting minimal changes in inequality. India and China remained major contributors. In contrast, SHF due to RHD burden concentrated in lower-SDI countries, reflected by negative regression slopes (prevalence: −4 to −2; YLDs: −1–0). The CI moderately increased (prevalence: −0.22 to −0.11; YLDs: −0.22 to −0.12), suggesting reduced inequality over time. India and China maintained substantial but less disproportionate burdens than in 1990 (Figure 5).

**Figure 6:**
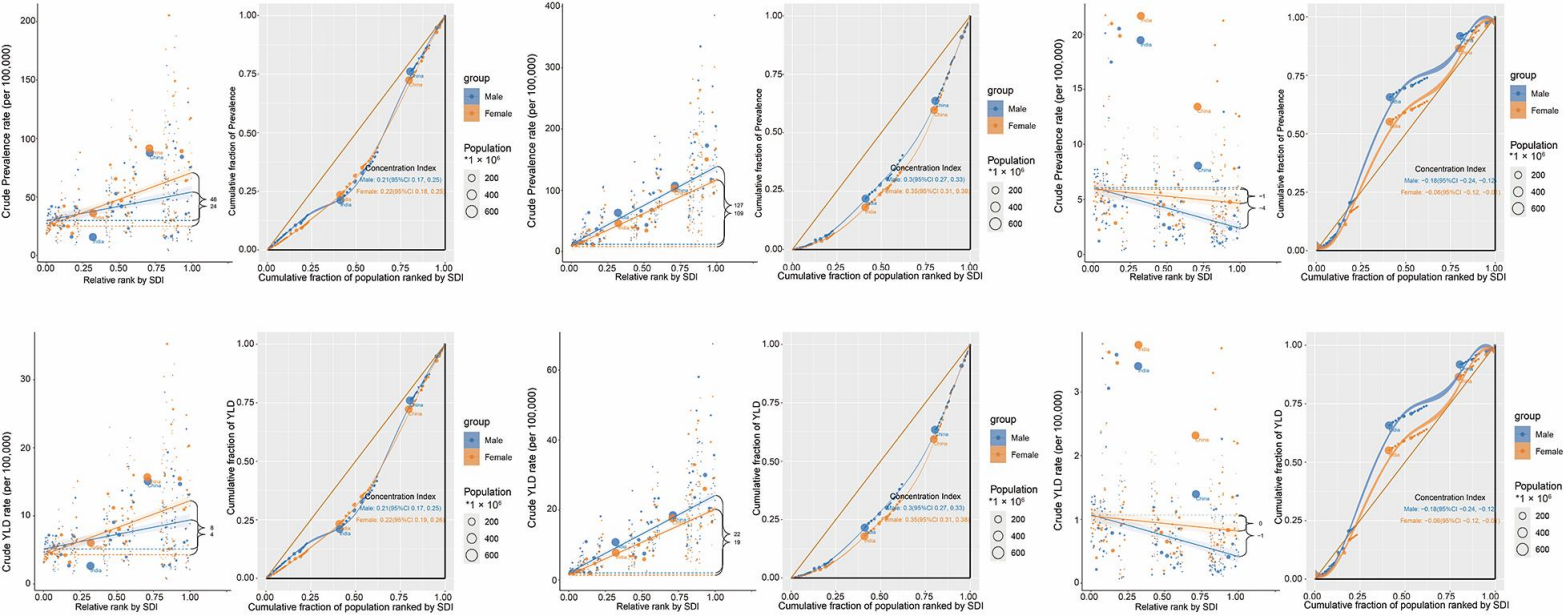
Sex-specific inequality regression and concentration curves of ASPR and ASYR for HHD, IHD, and RHD, 2021. ASPR, age-standardized prevalence rate; ASYR, age-standardized YLD rate; SDI, sociodemographic index; YLD: Years Lived with Disability.

Gender inequalities were minimal for SHF from HHD. For SHF due to IHD, females showed slightly greater inequalities (CI: 0.35 females vs. 0.30 males). Conversely, males experienced higher inequalities for SHF related to RHD in low-SDI settings (CI: −0.18 males vs. −0.06 females). Overall, gender disparities were modest compared with cross-country inequalities (Figure 6).

#### Projected global SHF burden due to HHD, IHD, and RHD

**Figure 7:**
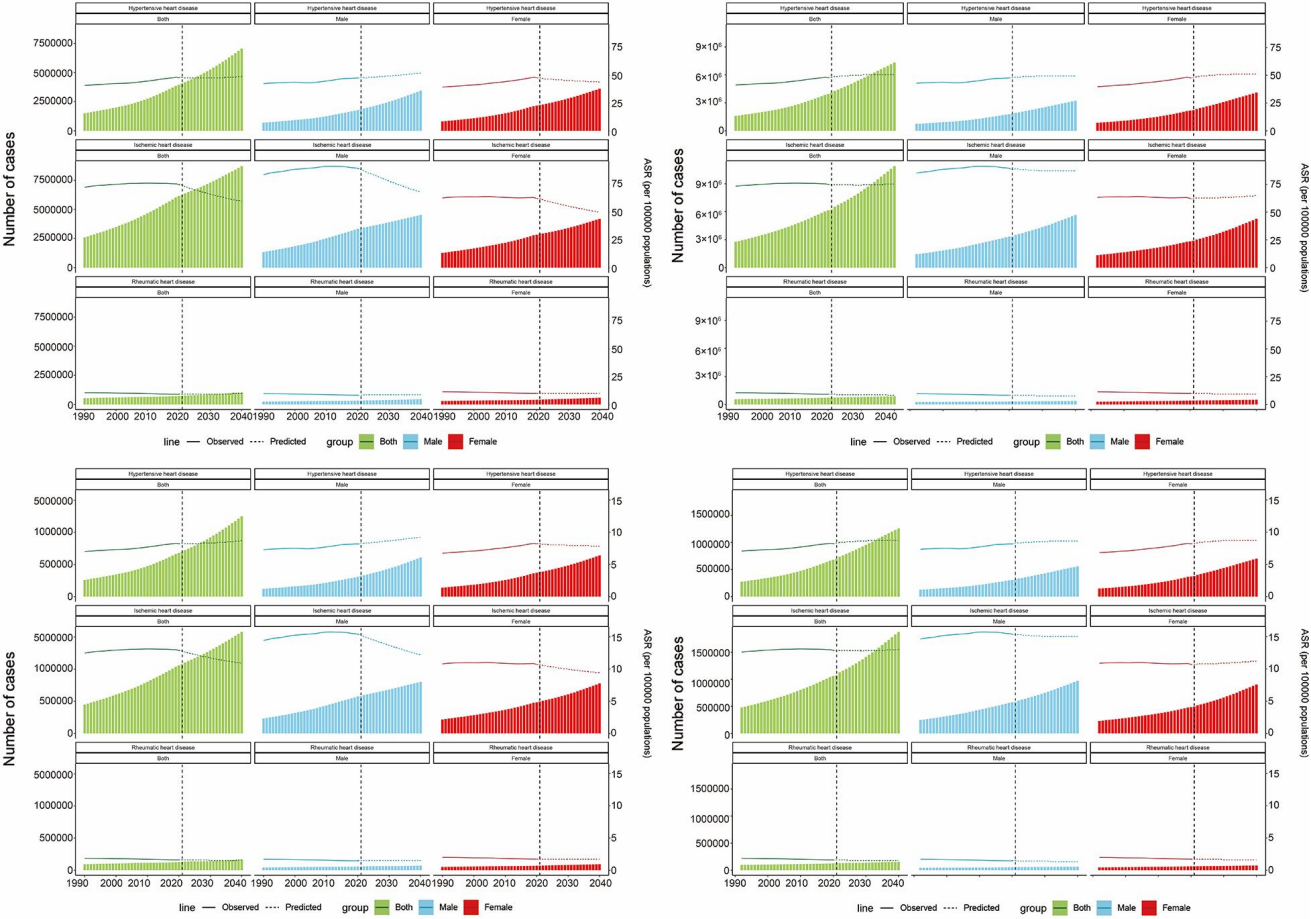
BAPC(left column) and nordpred(right column) projections of ASPR, ASYR, and number of HHD, IHD, and RHD cases from 2022 to 2040 with prevalence on the first row and YLDs on the second row. ASPR, age-standardized prevalence rate; ASYR, age-standardized YLD rate; BAPC, Bayesian age-period-cohort model; ASR, Age-Standardized Rate.

Global SHF burdens from HHD, IHD, and RHD (2022–2040) were projected using Nordpred and validated by BAPC. For SHF due to HHD, prevalent cases and YLDs are expected to steadily rise, with stable to slightly increasing ASPR and ASYR. SHF burden from IHD is projected to continuously increase in absolute numbers, while ASPR and ASYR remain relatively stable. SHF from RHD is projected to remain stable or slightly increase in absolute terms, with steady ASPR and ASYR. BAPC models confirmed Nordpred's overall forecasts. Specifically, for SHF from HHD, BAPC indicated slight ASPR and ASYR increases among males but modest declines among females. For SHF from IHD, BAPC projected mild declines in ASPR and ASYR across both sexes. SHF from RHD remained stable, consistent with Nordpred projections (Figure 7).

## Discussion

The present study provides a comprehensive overview of the global burden and evolving trends of severe heart failure resulting from HHD, IHD, and RHD from 1990 to 2021, highlighting significant shifts in prevalence, disability burden, and distinct epidemiological patterns. Notably, severe heart failure related to HHD and IHD exhibited marked increases in global prevalence, while severe heart failure resulting from RHD showed relatively modest changes, indicating varied epidemiological transitions and differing public health responses internationally.

Our findings emphasize a substantial rise in severe heart failure prevalence and associated disability burden due to HHD, particularly pronounced in regions with lower SDI, such as Eastern Sub-Saharan Africa. This increase likely stems from demographic changes, notably aging populations, alongside escalating hypertension prevalence driven by rapid urbanization, shifts towards energy-dense diets high in saturated fats, sugars, and sodium, increased sedentary behavior, and inadequate preventive healthcare services, particularly in resource-poor settings (18, 19). Additionally, limited awareness and poor adherence to antihypertensive treatments may exacerbate progression from hypertension to severe heart failure, highlighting critical gaps in public health education and healthcare accessibility (20, 21). The disparity between low and high SDI regions underscores a dual epidemiological challenge, with resource-limited areas struggling with persistent high-baseline burdens due to inadequate infrastructure, whereas affluent regions face growing severe heart failure incidence associated with aging populations and lifestyle factors, necessitating tailored intervention programs (22).

In contrast, severe heart failure resulting from IHD demonstrated significant absolute growth but relatively stable standardized rates, reflecting differing population dynamics and healthcare contexts. Higher-SDI regions exhibited consistently elevated prevalence, likely related to increased life expectancy, improved acute cardiac care prolonging survival, and chronic exposure to cardiovascular risk factors such as diabetes, obesity, smoking, and dyslipidemia, cumulatively leading to advanced disease states including severe heart failure (23). Rapidly developing regions, particularly East Asia, have faced pronounced increases in severe heart failure due to dietary shifts, and environmental risk factors like air pollution, exacerbating cardiovascular risk profiles despite economic improvements (24, 25). These trends underscore persistent challenges and suggest economic development alone may be insufficient without parallel advancements in public health infrastructure and preventive interventions (26, 27).

Severe heart failure due to RHD showed distinct epidemiological characteristics, with a slower global increase and declining trends in standardized prevalence, particularly in high and high-middle SDI regions. The observed bimodal age distribution is consistent with RHD's pathophysiology, linking early-life streptococcal exposure in lower-resource settings to chronic disease progression and eventual heart failure in older populations previously exposed decades earlier (28, 29). Declines in high-resource settings reflect successful implementation of primary prevention measures such as improved hygiene, widespread antibiotic use, and early medical interventions, significantly reducing progression to severe heart failure (30). Persistent high burdens in low-resource regions indicate unmet needs in primary and secondary prevention, reflecting broader socioeconomic inequities, insufficient healthcare access, and inadequate sanitation, demanding sustained international efforts and resource allocation (31).

Gender disparities observed across these cardiovascular conditions further highlight critical public health considerations. Males exhibited higher disability burdens associated with severe heart failure due to HHD and IHD, whereas females disproportionately experienced severe heart failure resulting from RHD. Complex interactions among biological factors, hormonal differences, differential risk factor exposure, health-seeking behaviors, and potential biases in healthcare delivery may drive these disparities, warranting targeted research and interventions to mitigate gender-based inequalities (32, 33).

Correlation analyses between SDI and severe heart failure burdens due to cardiovascular diseases reveal distinct epidemiological patterns. HHD-related severe heart failure generally declined with higher SDI, yet demonstrated a bimodal distribution indicative of transitional epidemiological dynamics in intermediate SDI regions. These patterns likely result from transitional healthcare infrastructures, partial implementation of effective preventive strategies, and concurrent increases in lifestyle-related risk factors (34). Conversely, IHD-related severe heart failure prevalence positively correlated with SDI, peaking at intermediate-high SDI levels, possibly due to increased cardiovascular risk factors during economic transitions and varying effectiveness of healthcare interventions across different development stages (35). Severe heart failure from RHD followed an inverted U-shaped pattern with highest prevalence in transitioning regions, reflecting disparities in healthcare accessibility, sanitation, and prevention strategies (36, 37).

Significant cross-country inequalities and gender-specific differences in severe heart failure burdens underscore broader systemic health inequities. Increased concentration of HHD-related severe heart failure in higher SDI countries highlights persistent lifestyle risk factors despite advanced healthcare, emphasizing preventive strategies alongside economic growth (38, 39). Persistent high burdens of severe heart failure from IHD in affluent nations illustrate ongoing challenges posed by traditional cardiovascular risks, requiring intensified public health measures (18, 23). The dominance of RHD-related severe heart failure in lower-SDI settings highlights fundamental socioeconomic disparities, underscoring the need for targeted interventions addressing social determinants of health (28, 39).

Future projections indicate divergent ways in severe heart failure burdens from these diseases, necessitating continuous monitoring and adaptive strategies. Stable or increasing HHD-related severe heart failure emphasizes enhanced hypertension management in resource-limited settings. Anticipated declines in IHD-related severe heart failure suggest advancements in preventive medicine; however, demographic aging may sustain overall burdens, emphasizing holistic risk management policies (40, 41). Stable burdens projected for RHD-related severe heart failure highlight significant public health challenges, particularly where prevention remains inadequate, necessitating sustained international cooperation and healthcare infrastructure investments (31).

## Limitations

This study's strengths include extensive global coverage, longitudinal analysis, and incorporation of socioeconomic determinants, enhancing understanding of severe heart failure epidemiology. Several limitations should be acknowledged in the interpretation of this study. Although the GBD study provides extensive global coverage with standardized methods, inherent variations in the quality, completeness, and reporting practices of data across different countries and regions may introduce bias. Our projections were conducted using Nordpred and validated by the Bayesian Age-Period-Cohort (BAPC) model, but these predictions depend on assumptions regarding current epidemiological, demographic, and healthcare trends continuing unchanged into the future. Sudden changes in risk factors, healthcare policy, or global health events may significantly alter actual future burdens. While we explored correlations between severe heart failure burdens and the SDI, other potentially relevant socioeconomic, behavioral, and environmental factors were not explicitly assessed, and their contributions to the disease burden may thus have been overlooked.

## Conclusion

The global and regional burdens of severe heart failure attributable to HHD, IHD, and RHD exhibited distinct epidemiological patterns from 1990 to 2021, underscoring significant health inequities across different SDI settings and between genders. While HHD and IHD related severe heart failure burdens continue to rise substantially, particularly driven by demographic aging and lifestyle factors, RHD related severe heart failure burdens remained relatively stable, reflecting uneven implementation of preventive measures globally. Projections to 2040 highlight ongoing challenges, with increasing prevalent cases and disability burdens, particularly for HHD and IHD, emphasizing the critical need for enhanced prevention, targeted public health interventions, and equitable healthcare resource allocation. Future public health strategies should prioritize addressing socioeconomic disparities, reinforcing primary and secondary prevention measures, and strengthening global cooperation to effectively mitigate the severe heart failure burden.

## Data Availability

The original contributions presented in the study are included in the article/Supplementary Material, further inquiries can be directed to the corresponding authors.
