# Supplementary material for: Global, regional, and national burden and projections of severe heart failure attributable to hypertensive, ischemic, and rheumatic heart diseases: an analysis from the global burden of disease study 2021
Source: Front Cardiovasc Med. 2026 Mar 9;13:1649064. doi: 10.3389/fcvm.2026.1649064 (PMC13006317; doi:10.3389/fcvm.2026.1649064)
Supplement: Supplementary file 1 [file Table1.docx]

**Table S1 The female and male prevalence of SHF due to HHD, IHD and RHD in 1990 and 2021.**

| Locations | cause | sex | 1990 Prevalence cases(95% UI) | 2021 Prevalence cases(95% UI) | 1990 ASPR(95% UI) | 2021 ASPR(95% UI) | 1990-2021 EAPC（95%CI） | 1990-2021 Prevalence cases changes(%) |
| --- | --- | --- | --- | --- | --- | --- | --- | --- |
| Global | Hypertensive heart disease | Female | 811611 ( 626305 to 1046389 ) | 2216111 ( 1694824 to 2873716 ) | 39.352 ( 30.381 to 50.333 ) | 47.767 ( 36.63 to 61.82 ) | 0.68 (0.65 to 0.71) | 173.051 |
| Global | Hypertensive heart disease | Male | 695368 ( 528410 to 888963 ) | 1857191 ( 1378748 to 2441922 ) | 42.545 ( 32.248 to 54.985 ) | 48.487 ( 36.491 to 63.621 ) | 0.38 (0.33 to 0.44) | 167.08 |
| Global | Ischemic heart disease | Female | 1258294 ( 976422 to 1613782 ) | 2891259 ( 2250438 to 3653894 ) | 62.691 ( 48.85 to 80.34 ) | 62.29 ( 48.617 to 78.59 ) | -0.04 (-0.07 to -0.02) | 129.776 |
| Global | Ischemic heart disease | Male | 1335143 ( 1045626 to 1673591 ) | 3351396 ( 2667633 to 4206246 ) | 83.675 ( 64.615 to 106.68 ) | 88.469 ( 70.064 to 110.791 ) | 0.22 (0.17 to 0.27) | 151.014 |
| Global | Rheumatic heart disease | Female | 288327 ( 238287 to 347562 ) | 412024 ( 334758 to 502760 ) | 11.39 ( 9.455 to 13.7 ) | 9.971 ( 8.154 to 12.15 ) | -0.51 (-0.54 to -0.48) | 42.902 |
| Global | Rheumatic heart disease | Male | 244239 ( 202493 to 296397 ) | 327994 ( 266883 to 406515 ) | 9.693 ( 8.079 to 11.672 ) | 8.412 ( 6.834 to 10.436 ) | -0.56 (-0.59 to -0.52) | 34.292 |
| High SDI | Hypertensive heart disease | Female | 168486 ( 127892 to 221474 ) | 449051 ( 341226 to 586331 ) | 25.522 ( 19.718 to 33.031 ) | 35.553 ( 27.791 to 45.512 ) | 1.5 (1.37 to 1.64) | 166.521 |
| High SDI | Hypertensive heart disease | Male | 106939 ( 80934 to 138759 ) | 344696 ( 259086 to 448599 ) | 23.51 ( 17.924 to 30.22 ) | 37.496 ( 28.839 to 48.278 ) | 1.69 (1.58 to 1.8) | 222.33 |
| High SDI | Ischemic heart disease | Female | 505640 ( 384703 to 658988 ) | 823331 ( 640525 to 1044900 ) | 73.778 ( 56.72 to 95.469 ) | 62.497 ( 49.565 to 78.196 ) | -0.76 (-0.86 to -0.67) | 62.829 |
| High SDI | Ischemic heart disease | Male | 489998 ( 365950 to 628057 ) | 935315 ( 733637 to 1170284 ) | 109.778 ( 81.454 to 140.759 ) | 97.872 ( 77.591 to 121.476 ) | -0.59 (-0.66 to -0.51) | 90.881 |
| High SDI | Rheumatic heart disease | Female | 32589 ( 25488 to 40958 ) | 42803 ( 33100 to 53675 ) | 5.512 ( 4.415 to 6.813 ) | 4.152 ( 3.374 to 5.04 ) | -0.89 (-0.98 to -0.8) | 31.342 |
| High SDI | Rheumatic heart disease | Male | 14150 ( 11174 to 17404 ) | 22021 ( 16808 to 27784 ) | 3.186 ( 2.526 to 3.924 ) | 2.71 ( 2.163 to 3.304 ) | -0.66 (-0.78 to -0.53) | 55.625 |
| High-middle SDI | Hypertensive heart disease | Female | 178185 ( 134075 to 229919 ) | 490941 ( 370290 to 644470 ) | 32.526 ( 24.79 to 41.919 ) | 43.726 ( 33.248 to 57.043 ) | 1 (0.95 to 1.06) | 175.523 |
| High-middle SDI | Hypertensive heart disease | Male | 145034 ( 107457 to 188082 ) | 401860 ( 291431 to 534540 ) | 38.723 ( 28.43 to 50.423 ) | 47.406 ( 34.723 to 62.769 ) | 0.66 (0.61 to 0.71) | 177.08 |
| High-middle SDI | Ischemic heart disease | Female | 369619 ( 285739 to 477500 ) | 801530 ( 623658 to 1014486 ) | 68.839 ( 53.878 to 88.219 ) | 71.155 ( 55.853 to 89.951 ) | 0.18 (0.15 to 0.2) | 116.853 |
| High-middle SDI | Ischemic heart disease | Male | 320390 ( 246004 to 405141 ) | 792740 ( 626335 to 996726 ) | 86.103 ( 64.533 to 109.987 ) | 93.617 ( 73.839 to 117.54 ) | 0.43 (0.33 to 0.52) | 147.43 |
| High-middle SDI | Rheumatic heart disease | Female | 44510 ( 35972 to 55078 ) | 59152 ( 44556 to 77787 ) | 8.014 ( 6.536 to 9.878 ) | 6.229 ( 4.87 to 7.85 ) | -1.12 (-1.22 to -1.01) | 32.896 |
| High-middle SDI | Rheumatic heart disease | Male | 26140 ( 21245 to 32064 ) | 32630 ( 24607 to 43119 ) | 5.704 ( 4.611 to 6.979 ) | 4.169 ( 3.238 to 5.378 ) | -1.27 (-1.34 to -1.2) | 24.828 |
| Low SDI | Hypertensive heart disease | Female | 56232 ( 42063 to 72963 ) | 145607 ( 109988 to 186196 ) | 61.706 ( 46.293 to 80.033 ) | 64.898 ( 48.653 to 84.414 ) | 0.21 (0.19 to 0.23) | 158.94 |
| Low SDI | Hypertensive heart disease | Male | 66003 ( 49395 to 85742 ) | 152650 ( 114968 to 196636 ) | 70.09 ( 52.198 to 90.988 ) | 71.123 ( 53.19 to 93.903 ) | 0.04 (0.01 to 0.07) | 131.277 |
| Low SDI | Ischemic heart disease | Female | 33138 ( 25739 to 42345 ) | 90835 ( 70371 to 116182 ) | 34.968 ( 26.487 to 45.391 ) | 39.564 ( 29.552 to 52.116 ) | 0.4 (0.38 to 0.41) | 174.111 |
| Low SDI | Ischemic heart disease | Male | 50603 ( 39576 to 64348 ) | 135207 ( 106447 to 170461 ) | 53.946 ( 41.016 to 69.328 ) | 63.062 ( 48.797 to 81.254 ) | 0.61 (0.58 to 0.64) | 167.192 |
| Low SDI | Rheumatic heart disease | Female | 28840 ( 23386 to 36191 ) | 52343 ( 42108 to 65844 ) | 12.106 ( 9.885 to 14.857 ) | 10.395 ( 8.468 to 12.882 ) | -0.45 (-0.48 to -0.42) | 81.494 |
| Low SDI | Rheumatic heart disease | Male | 31112 ( 25345 to 39057 ) | 52605 ( 42331 to 66026 ) | 12.849 ( 10.581 to 15.71 ) | 10.112 ( 8.303 to 12.401 ) | -0.8 (-0.83 to -0.77) | 69.083 |
| Low-middle SDI | Hypertensive heart disease | Female | 129603 ( 101616 to 163309 ) | 360974 ( 274080 to 461482 ) | 51.074 ( 40.001 to 64.713 ) | 53.227 ( 40.531 to 68.603 ) | 0.12 (0.11 to 0.13) | 178.523 |
| Low-middle SDI | Hypertensive heart disease | Male | 112072 ( 87108 to 142437 ) | 271870 ( 206512 to 352622 ) | 43.597 ( 33.869 to 55.569 ) | 44.683 ( 33.841 to 57.875 ) | -0.01 (-0.03 to 0.02) | 142.585 |
| Low-middle SDI | Ischemic heart disease | Female | 121670 ( 98002 to 149750 ) | 356541 ( 279854 to 447640 ) | 45.143 ( 35.701 to 56.984 ) | 51.275 ( 40.204 to 64.646 ) | 0.49 (0.45 to 0.53) | 193.039 |
| Low-middle SDI | Ischemic heart disease | Male | 178204 ( 145307 to 217716 ) | 489159 ( 392804 to 607901 ) | 65.402 ( 51.969 to 81.484 ) | 78.368 ( 62.021 to 98.633 ) | 0.71 (0.68 to 0.74) | 174.494 |
| Low-middle SDI | Rheumatic heart disease | Female | 93696 ( 77048 to 113715 ) | 138884 ( 114750 to 170618 ) | 17.651 ( 14.724 to 21.014 ) | 15.181 ( 12.583 to 18.498 ) | -0.53 (-0.57 to -0.5) | 48.228 |
| Low-middle SDI | Rheumatic heart disease | Male | 98940 ( 80767 to 121240 ) | 129919 ( 106965 to 160445 ) | 17.467 ( 14.619 to 20.894 ) | 13.915 ( 11.523 to 17.08 ) | -0.85 (-0.89 to -0.81) | 31.311 |
| Middle SDI | Hypertensive heart disease | Female | 278225 ( 217462 to 352601 ) | 767345 ( 579404 to 1003761 ) | 59.778 ( 46.401 to 76.512 ) | 56.947 ( 43.117 to 74.173 ) | -0.32 (-0.46 to -0.19) | 175.8 |
| Middle SDI | Hypertensive heart disease | Male | 264613 ( 202795 to 340982 ) | 684250 ( 500992 to 907756 ) | 63.962 ( 48.945 to 82.299 ) | 59.001 ( 43.711 to 77.951 ) | -0.37 (-0.48 to -0.26) | 158.585 |
| Middle SDI | Ischemic heart disease | Female | 226474 ( 180410 to 281057 ) | 816049 ( 625845 to 1031454 ) | 48.898 ( 38.641 to 61.78 ) | 61.216 ( 47.014 to 77.586 ) | 0.9 (0.85 to 0.95) | 260.328 |
| Middle SDI | Ischemic heart disease | Male | 293889 ( 238612 to 361037 ) | 995592 ( 785011 to 1243006 ) | 70.65 ( 56.404 to 88.351 ) | 86.309 ( 67.724 to 108.664 ) | 0.89 (0.8 to 0.98) | 238.765 |
| Middle SDI | Rheumatic heart disease | Female | 88477 ( 73436 to 106056 ) | 118639 ( 94346 to 144441 ) | 11.842 ( 9.856 to 14.203 ) | 9.331 ( 7.501 to 11.426 ) | -0.91 (-0.95 to -0.87) | 34.09 |
| Middle SDI | Rheumatic heart disease | Male | 73732 ( 60857 to 89486 ) | 90670 ( 72288 to 111822 ) | 9.597 ( 7.989 to 11.629 ) | 7.592 ( 6.065 to 9.366 ) | -0.85 (-0.89 to -0.82) | 22.972 |
| Andean Latin America | Hypertensive heart disease | Female | 5196 ( 4016 to 6595 ) | 14828 ( 11221 to 19304 ) | 50.488 ( 38.644 to 64.921 ) | 48.153 ( 36.28 to 63.15 ) | 0.03 (-0.05 to 0.12) | 185.373 |
| Andean Latin America | Hypertensive heart disease | Male | 4411 ( 3441 to 5656 ) | 13170 ( 9886 to 17416 ) | 47.305 ( 36.391 to 61.455 ) | 48.549 ( 36.227 to 64.868 ) | 0.33 (0.21 to 0.45) | 198.572 |
| Andean Latin America | Ischemic heart disease | Female | 6121 ( 4786 to 7656 ) | 19584 ( 15030 to 25078 ) | 60.024 ( 46.412 to 77.242 ) | 63.563 ( 48.842 to 81.702 ) | -0.02 (-0.16 to 0.12) | 219.948 |
| Andean Latin America | Ischemic heart disease | Male | 8442 ( 6695 to 10748 ) | 26212 ( 20193 to 33458 ) | 89.844 ( 70.357 to 116.021 ) | 96.387 ( 73.661 to 123.716 ) | 0.01 (-0.13 to 0.15) | 210.495 |
| Andean Latin America | Rheumatic heart disease | Female | 984 ( 786 to 1203 ) | 1116 ( 900 to 1374 ) | 6.08 ( 4.936 to 7.417 ) | 3.453 ( 2.786 to 4.257 ) | -1.87 (-1.96 to -1.78) | 13.415 |
| Andean Latin America | Rheumatic heart disease | Male | 671 ( 531 to 838 ) | 702 ( 565 to 860 ) | 4.006 ( 3.267 to 4.935 ) | 2.255 ( 1.825 to 2.744 ) | -2.04 (-2.14 to -1.94) | 4.62 |
| Australasia | Hypertensive heart disease | Female | 1487 ( 1118 to 1936 ) | 5315 ( 4055 to 6785 ) | 10.779 ( 8.122 to 13.884 ) | 16.403 ( 12.679 to 20.86 ) | 1.78 (1.66 to 1.9) | 257.431 |
| Australasia | Hypertensive heart disease | Male | 887 ( 671 to 1163 ) | 4476 ( 3417 to 5866 ) | 8.898 ( 6.643 to 11.659 ) | 17.407 ( 13.464 to 22.642 ) | 2.28 (2.13 to 2.43) | 404.622 |
| Australasia | Ischemic heart disease | Female | 13830 ( 10420 to 18018 ) | 25100 ( 19654 to 31661 ) | 99.198 ( 74.745 to 128.806 ) | 77.088 ( 60.991 to 96.533 ) | -1.57 (-1.84 to -1.31) | 81.49 |
| Australasia | Ischemic heart disease | Male | 15755 ( 11773 to 20641 ) | 32663 ( 24977 to 41786 ) | 158.104 ( 119.593 to 210.235 ) | 125.397 ( 96.628 to 158.739 ) | -1.32 (-1.51 to -1.13) | 107.318 |
| Australasia | Rheumatic heart disease | Female | 789 ( 638 to 953 ) | 1566 ( 1276 to 1885 ) | 6.619 ( 5.365 to 7.873 ) | 6.654 ( 5.501 to 7.917 ) | 0.29 (0.11 to 0.47) | 98.479 |
| Australasia | Rheumatic heart disease | Male | 422 ( 339 to 513 ) | 904 ( 714 to 1130 ) | 4.225 ( 3.384 to 5.102 ) | 4.515 ( 3.691 to 5.465 ) | 0.11 (-0.04 to 0.25) | 114.218 |
| Caribbean | Hypertensive heart disease | Female | 5925 ( 4615 to 7613 ) | 16913 ( 12839 to 22020 ) | 44.468 ( 34.41 to 57.368 ) | 57.821 ( 43.824 to 75.385 ) | 1.05 (0.99 to 1.11) | 185.451 |
| Caribbean | Hypertensive heart disease | Male | 5806 ( 4429 to 7565 ) | 17363 ( 12885 to 23002 ) | 47.921 ( 36.54 to 62.987 ) | 70.88 ( 52.741 to 94.435 ) | 1.44 (1.38 to 1.5) | 199.053 |
| Caribbean | Ischemic heart disease | Female | 11095 ( 8550 to 14548 ) | 20051 ( 15444 to 26341 ) | 85.293 ( 65.429 to 111.782 ) | 67.284 ( 51.487 to 88.169 ) | -0.9 (-0.95 to -0.85) | 80.721 |
| Caribbean | Ischemic heart disease | Male | 15265 ( 11618 to 20042 ) | 25066 ( 19006 to 33234 ) | 129.404 ( 98.317 to 171.464 ) | 102.534 ( 77.892 to 136.243 ) | -0.9 (-0.96 to -0.84) | 64.206 |
| Caribbean | Rheumatic heart disease | Female | 1764 ( 1370 to 2254 ) | 1484 ( 1177 to 1846 ) | 9.382 ( 7.366 to 11.935 ) | 6.429 ( 5.068 to 8.088 ) | -1.4 (-1.47 to -1.33) | -15.873 |
| Caribbean | Rheumatic heart disease | Male | 1325 ( 1024 to 1739 ) | 1078 ( 845 to 1363 ) | 6.99 ( 5.468 to 9.012 ) | 4.82 ( 3.775 to 6.124 ) | -1.5 (-1.61 to -1.38) | -18.642 |
| Central Asia | Hypertensive heart disease | Female | 6945 ( 5113 to 9509 ) | 12074 ( 8449 to 16173 ) | 25.598 ( 18.785 to 35.128 ) | 29.14 ( 20.178 to 39.769 ) | 0.95 (0.74 to 1.16) | 73.852 |
| Central Asia | Hypertensive heart disease | Male | 4492 ( 3179 to 6002 ) | 10512 ( 6999 to 14848 ) | 25.982 ( 17.449 to 36.054 ) | 32.945 ( 19.862 to 47.596 ) | 0.92 (0.7 to 1.14) | 134.016 |
| Central Asia | Ischemic heart disease | Female | 26017 ( 19046 to 35332 ) | 33948 ( 24919 to 45958 ) | 97.257 ( 71.516 to 131.621 ) | 85.033 ( 61.985 to 115.588 ) | -0.63 (-0.75 to -0.5) | 30.484 |
| Central Asia | Ischemic heart disease | Male | 19592 ( 14114 to 25876 ) | 30715 ( 21090 to 41441 ) | 119.07 ( 80.946 to 164.208 ) | 101.257 ( 66.268 to 141.685 ) | -0.72 (-0.85 to -0.59) | 56.773 |
| Central Asia | Rheumatic heart disease | Female | 3576 ( 2863 to 4472 ) | 2609 ( 2058 to 3221 ) | 9.716 ( 7.747 to 11.972 ) | 5.699 ( 4.484 to 7.058 ) | -1.73 (-1.86 to -1.6) | -27.041 |
| Central Asia | Rheumatic heart disease | Male | 3079 ( 2439 to 3930 ) | 2254 ( 1747 to 2846 ) | 8.377 ( 6.702 to 10.441 ) | 4.939 ( 3.899 to 6.247 ) | -1.95 (-2.1 to -1.81) | -26.794 |
| Central Europe | Hypertensive heart disease | Female | 26832 ( 19547 to 36100 ) | 63346 ( 45928 to 84008 ) | 31.627 ( 23.453 to 42.041 ) | 44.318 ( 32.78 to 58.061 ) | 1.55 (1.41 to 1.69) | 136.084 |
| Central Europe | Hypertensive heart disease | Male | 19650 ( 13860 to 26255 ) | 47121 ( 33264 to 62502 ) | 33.247 ( 23.848 to 44.942 ) | 50.573 ( 36.715 to 66.562 ) | 1.87 (1.7 to 2.03) | 139.802 |
| Central Europe | Ischemic heart disease | Female | 63651 ( 47717 to 84167 ) | 103023 ( 79211 to 132425 ) | 76.958 ( 58.74 to 100.663 ) | 70.072 ( 54.41 to 89.908 ) | -0.36 (-0.46 to -0.26) | 61.856 |
| Central Europe | Ischemic heart disease | Male | 71049 ( 53748 to 91364 ) | 102948 ( 79709 to 128799 ) | 124.06 ( 94.013 to 159.772 ) | 110.647 ( 86.865 to 138.035 ) | -0.46 (-0.51 to -0.41) | 44.897 |
| Central Europe | Rheumatic heart disease | Female | 4772 ( 3755 to 5992 ) | 3183 ( 2451 to 4146 ) | 6.188 ( 4.973 to 7.672 ) | 3.158 ( 2.562 to 3.905 ) | -2.31 (-2.53 to -2.08) | -33.298 |
| Central Europe | Rheumatic heart disease | Male | 3445 ( 2780 to 4322 ) | 1719 ( 1307 to 2207 ) | 5.497 ( 4.48 to 6.78 ) | 2.28 ( 1.842 to 2.8 ) | -3.14 (-3.44 to -2.84) | -50.102 |
| Central Latin America | Hypertensive heart disease | Female | 18640 ( 14536 to 23671 ) | 54071 ( 40695 to 70721 ) | 46.958 ( 36.084 to 60.067 ) | 40.957 ( 30.702 to 53.433 ) | -0.63 (-0.7 to -0.56) | 190.08 |
| Central Latin America | Hypertensive heart disease | Male | 14950 ( 11453 to 19110 ) | 38603 ( 28372 to 50827 ) | 41.993 ( 31.849 to 54.919 ) | 36.177 ( 26.719 to 48.257 ) | -0.7 (-0.77 to -0.64) | 158.214 |
| Central Latin America | Ischemic heart disease | Female | 26799 ( 21412 to 33436 ) | 95704 ( 74834 to 120884 ) | 66.732 ( 52.56 to 84.358 ) | 72.218 ( 56.287 to 91.581 ) | 0.33 (0.29 to 0.36) | 257.118 |
| Central Latin America | Ischemic heart disease | Male | 36187 ( 29234 to 44292 ) | 116928 ( 91898 to 146791 ) | 96.684 ( 76.253 to 120.961 ) | 107.179 ( 83.956 to 134.999 ) | 0.35 (0.31 to 0.4) | 223.122 |
| Central Latin America | Rheumatic heart disease | Female | 5280 ( 4360 to 6426 ) | 2513 ( 1978 to 3176 ) | 7.265 ( 6.07 to 8.71 ) | 1.88 ( 1.479 to 2.361 ) | -4.86 (-5.02 to -4.71) | -52.405 |
| Central Latin America | Rheumatic heart disease | Male | 3340 ( 2792 to 4050 ) | 1318 ( 1065 to 1629 ) | 4.409 ( 3.685 to 5.327 ) | 1.111 ( 0.891 to 1.376 ) | -5.14 (-5.4 to -4.88) | -60.539 |
| Central Sub-Saharan Africa | Hypertensive heart disease | Female | 5847 ( 4109 to 7808 ) | 17223 ( 12432 to 23229 ) | 63.752 ( 44.974 to 87.248 ) | 70.233 ( 49.78 to 96.163 ) | 0.34 (0.3 to 0.39) | 194.561 |
| Central Sub-Saharan Africa | Hypertensive heart disease | Male | 6779 ( 4789 to 9032 ) | 17412 ( 12419 to 23090 ) | 81.788 ( 57.769 to 110.961 ) | 88.794 ( 62.124 to 119.377 ) | 0.3 (0.25 to 0.35) | 156.852 |
| Central Sub-Saharan Africa | Ischemic heart disease | Female | 3245 ( 2292 to 4337 ) | 9561 ( 6950 to 13001 ) | 36.814 ( 25.6 to 50.49 ) | 39.984 ( 28.671 to 55.134 ) | 0.19 (0.16 to 0.21) | 194.638 |
| Central Sub-Saharan Africa | Ischemic heart disease | Male | 4114 ( 2926 to 5595 ) | 10291 ( 7573 to 13610 ) | 53.578 ( 37.365 to 74.194 ) | 56.894 ( 41.274 to 77.957 ) | 0.14 (0.11 to 0.17) | 150.146 |
| Central Sub-Saharan Africa | Rheumatic heart disease | Female | 2083 ( 1436 to 2986 ) | 3595 ( 2389 to 5145 ) | 6.432 ( 4.46 to 9.06 ) | 4.8 ( 3.273 to 6.673 ) | -1.05 (-1.12 to -0.98) | 72.588 |
| Central Sub-Saharan Africa | Rheumatic heart disease | Male | 1969 ( 1346 to 2782 ) | 3278 ( 2145 to 4814 ) | 5.921 ( 4.204 to 8.119 ) | 4.267 ( 2.999 to 5.968 ) | -1.24 (-1.32 to -1.16) | 66.48 |
| East Asia | Hypertensive heart disease | Female | 247181 ( 183947 to 317723 ) | 664852 ( 493512 to 877575 ) | 64.836 ( 48.847 to 82.403 ) | 58.97 ( 44.105 to 77.504 ) | -0.63 (-0.89 to -0.37) | 168.974 |
| East Asia | Hypertensive heart disease | Male | 257810 ( 192401 to 336234 ) | 659617 ( 472197 to 886265 ) | 76.683 ( 57.346 to 99.137 ) | 66.965 ( 49.05 to 89.427 ) | -0.61 (-0.81 to -0.42) | 155.854 |
| East Asia | Ischemic heart disease | Female | 158026 ( 122672 to 204983 ) | 735631 ( 556124 to 946596 ) | 41.961 ( 32.709 to 54.551 ) | 66.266 ( 50.075 to 84.834 ) | 1.82 (1.69 to 1.95) | 365.513 |
| East Asia | Ischemic heart disease | Male | 186255 ( 144293 to 236265 ) | 802663 ( 613890 to 1033907 ) | 54.71 ( 41.744 to 69.884 ) | 82.779 ( 64.101 to 105.171 ) | 1.88 (1.65 to 2.12) | 330.948 |
| East Asia | Rheumatic heart disease | Female | 62494 ( 50725 to 76040 ) | 94947 ( 69420 to 124495 ) | 12.846 ( 10.299 to 15.552 ) | 8.853 ( 6.695 to 11.403 ) | -1.57 (-1.7 to -1.44) | 51.93 |
| East Asia | Rheumatic heart disease | Male | 42262 ( 33519 to 52051 ) | 59570 ( 42929 to 80820 ) | 9.224 ( 7.324 to 11.231 ) | 6.121 ( 4.678 to 8.035 ) | -1.55 (-1.64 to -1.45) | 40.954 |
| Eastern Europe | Hypertensive heart disease | Female | 17675 ( 12580 to 24159 ) | 33968 ( 23252 to 48195 ) | 9.583 ( 6.868 to 12.974 ) | 14.096 ( 9.737 to 19.848 ) | 1.7 (1.51 to 1.88) | 92.181 |
| Eastern Europe | Hypertensive heart disease | Male | 7671 ( 5386 to 10710 ) | 16504 ( 10697 to 23389 ) | 8.305 ( 5.79 to 11.434 ) | 13.261 ( 8.727 to 19.069 ) | 1.92 (1.74 to 2.1) | 115.148 |
| Eastern Europe | Ischemic heart disease | Female | 186544 ( 144739 to 240870 ) | 230601 ( 180167 to 297080 ) | 101.519 ( 79.859 to 130.004 ) | 93.178 ( 73.035 to 119.978 ) | -0.22 (-0.3 to -0.14) | 23.617 |
| Eastern Europe | Ischemic heart disease | Male | 98209 ( 72767 to 123150 ) | 142483 ( 105828 to 184236 ) | 123.044 ( 90.359 to 157.228 ) | 117.165 ( 87.429 to 151.093 ) | -0.19 (-0.27 to -0.11) | 45.081 |
| Eastern Europe | Rheumatic heart disease | Female | 6759 ( 5141 to 8833 ) | 3455 ( 2348 to 4947 ) | 4.559 ( 3.609 to 5.792 ) | 1.838 ( 1.34 to 2.465 ) | -3.35 (-3.68 to -3.01) | -48.883 |
| Eastern Europe | Rheumatic heart disease | Male | 3428 ( 2707 to 4219 ) | 940 ( 666 to 1261 ) | 3.315 ( 2.646 to 4.111 ) | 0.883 ( 0.662 to 1.146 ) | -4.95 (-5.15 to -4.75) | -72.579 |
| Eastern Sub-Saharan Africa | Hypertensive heart disease | Female | 23688 ( 17197 to 31033 ) | 61150 ( 45597 to 78272 ) | 76.841 ( 56.396 to 102.243 ) | 79.905 ( 59.231 to 104.703 ) | 0.16 (0.14 to 0.18) | 158.148 |
| Eastern Sub-Saharan Africa | Hypertensive heart disease | Male | 33326 ( 24641 to 43702 ) | 77464 ( 58362 to 98915 ) | 108.287 ( 79.984 to 142.95 ) | 113.273 ( 84.499 to 149.96 ) | 0.07 (0.04 to 0.1) | 132.443 |
| Eastern Sub-Saharan Africa | Ischemic heart disease | Female | 9464 ( 6876 to 12824 ) | 27305 ( 20022 to 36627 ) | 31.848 ( 22.75 to 44.602 ) | 36.749 ( 26.631 to 50.687 ) | 0.45 (0.4 to 0.51) | 188.514 |
| Eastern Sub-Saharan Africa | Ischemic heart disease | Male | 14510 ( 10630 to 19474 ) | 35837 ( 26711 to 47403 ) | 50.575 ( 36.25 to 68.486 ) | 55.176 ( 40.293 to 74.837 ) | 0.26 (0.22 to 0.29) | 146.981 |
| Eastern Sub-Saharan Africa | Rheumatic heart disease | Female | 5279 ( 3731 to 7311 ) | 8550 ( 5929 to 11737 ) | 4.775 ( 3.495 to 6.38 ) | 3.774 ( 2.752 to 5.08 ) | -0.73 (-0.77 to -0.69) | 61.962 |
| Eastern Sub-Saharan Africa | Rheumatic heart disease | Male | 4176 ( 2898 to 5940 ) | 6186 ( 4186 to 8717 ) | 3.962 ( 2.887 to 5.342 ) | 2.906 ( 2.087 to 3.926 ) | -1.18 (-1.24 to -1.12) | 48.132 |
| High-income Asia Pacific | Hypertensive heart disease | Female | 20405 ( 14290 to 28017 ) | 63810 ( 45128 to 87915 ) | 19.179 ( 13.741 to 25.992 ) | 19.208 ( 14.781 to 24.855 ) | -0.13 (-0.3 to 0.05) | 212.717 |
| High-income Asia Pacific | Hypertensive heart disease | Male | 13835 ( 10239 to 18547 ) | 33028 ( 24142 to 44596 ) | 16.827 ( 12.113 to 22.386 ) | 17.177 ( 12.953 to 22.429 ) | -0.28 (-0.53 to -0.04) | 138.728 |
| High-income Asia Pacific | Ischemic heart disease | Female | 33860 ( 23562 to 45116 ) | 101507 ( 74772 to 132508 ) | 31.432 ( 21.994 to 41.654 ) | 32.606 ( 24.926 to 41.451 ) | -0.07 (-0.21 to 0.06) | 199.784 |
| High-income Asia Pacific | Ischemic heart disease | Male | 39269 ( 29199 to 50867 ) | 101118 ( 77892 to 128989 ) | 47.667 ( 34.319 to 62.844 ) | 53.265 ( 42.447 to 66.069 ) | 0.4 (0.28 to 0.52) | 157.501 |
| High-income Asia Pacific | Rheumatic heart disease | Female | 3872 ( 2777 to 5161 ) | 6278 ( 4606 to 8122 ) | 3.677 ( 2.735 to 4.796 ) | 2.7 ( 2.137 to 3.338 ) | -1.12 (-1.2 to -1.04) | 62.138 |
| High-income Asia Pacific | Rheumatic heart disease | Male | 1672 ( 1242 to 2175 ) | 2609 ( 1921 to 3476 ) | 2.066 ( 1.544 to 2.689 ) | 1.569 ( 1.221 to 1.976 ) | -1.06 (-1.17 to -0.95) | 56.041 |
| High-income North America | Hypertensive heart disease | Female | 59939 ( 44921 to 78468 ) | 162958 ( 125634 to 208407 ) | 30.021 ( 22.873 to 38.614 ) | 45.716 ( 35.867 to 57.28 ) | 1.68 (1.55 to 1.81) | 171.873 |
| High-income North America | Hypertensive heart disease | Male | 43137 ( 32193 to 57232 ) | 146330 ( 110056 to 187865 ) | 28.884 ( 21.548 to 38.112 ) | 52.021 ( 39.9 to 65.575 ) | 2.22 (2.07 to 2.37) | 239.222 |
| High-income North America | Ischemic heart disease | Female | 197192 ( 148280 to 252943 ) | 297432 ( 233105 to 368814 ) | 89.933 ( 68.565 to 113.595 ) | 75.724 ( 59.701 to 93.289 ) | -0.93 (-1.05 to -0.81) | 50.834 |
| High-income North America | Ischemic heart disease | Male | 191202 ( 139832 to 245001 ) | 332625 ( 257423 to 420765 ) | 129.783 ( 95.839 to 166.215 ) | 110.922 ( 85.927 to 139.077 ) | -0.86 (-0.96 to -0.77) | 73.965 |
| High-income North America | Rheumatic heart disease | Female | 9686 ( 7364 to 12516 ) | 10499 ( 7921 to 13435 ) | 5.285 ( 4.131 to 6.684 ) | 3.39 ( 2.668 to 4.208 ) | -1.56 (-1.96 to -1.17) | 8.394 |
| High-income North America | Rheumatic heart disease | Male | 3826 ( 2968 to 4911 ) | 4842 ( 3592 to 6379 ) | 2.677 ( 2.11 to 3.392 ) | 2.029 ( 1.555 to 2.593 ) | -0.9 (-1.32 to -0.47) | 26.555 |
| North Africa and Middle East | Hypertensive heart disease | Female | 56233 ( 44736 to 69753 ) | 153592 ( 118818 to 191983 ) | 74.114 ( 57.685 to 95.103 ) | 73.768 ( 56.454 to 93.666 ) | 0.09 (0.05 to 0.12) | 173.135 |
| North Africa and Middle East | Hypertensive heart disease | Male | 62675 ( 49396 to 78399 ) | 181129 ( 141447 to 227792 ) | 81.984 ( 63.382 to 104.648 ) | 84.605 ( 64.457 to 109.004 ) | 0.16 (0.1 to 0.22) | 188.997 |
| North Africa and Middle East | Ischemic heart disease | Female | 57361 ( 46145 to 70786 ) | 165183 ( 131210 to 204530 ) | 72.691 ( 57.152 to 92.297 ) | 78.663 ( 61.682 to 99.454 ) | 0.19 (0.14 to 0.23) | 187.971 |
| North Africa and Middle East | Ischemic heart disease | Male | 100491 ( 82166 to 123396 ) | 285204 ( 227481 to 355241 ) | 121.8 ( 97.175 to 151.564 ) | 126.897 ( 100.277 to 158.841 ) | 0.11 (0.05 to 0.16) | 183.81 |
| North Africa and Middle East | Rheumatic heart disease | Female | 15591 ( 12460 to 19682 ) | 16793 ( 13491 to 20750 ) | 8.438 ( 6.867 to 10.433 ) | 5.711 ( 4.616 to 6.987 ) | -1.41 (-1.48 to -1.35) | 7.71 |
| North Africa and Middle East | Rheumatic heart disease | Male | 13724 ( 10725 to 17673 ) | 15152 ( 12098 to 19125 ) | 7.075 ( 5.721 to 8.83 ) | 4.883 ( 3.894 to 6.07 ) | -1.44 (-1.52 to -1.37) | 10.405 |
| Oceania | Hypertensive heart disease | Female | 505 ( 395 to 636 ) | 1201 ( 922 to 1530 ) | 43.14 ( 32.943 to 56.241 ) | 38.656 ( 29.033 to 50.069 ) | -0.45 (-0.53 to -0.38) | 137.822 |
| Oceania | Hypertensive heart disease | Male | 396 ( 303 to 517 ) | 993 ( 757 to 1269 ) | 32.021 ( 23.933 to 42.323 ) | 30.995 ( 23.277 to 40.671 ) | -0.22 (-0.25 to -0.18) | 150.758 |
| Oceania | Ischemic heart disease | Female | 497 ( 389 to 627 ) | 1557 ( 1206 to 2004 ) | 48.474 ( 37.047 to 63.866 ) | 57.752 ( 43.763 to 75.425 ) | 0.6 (0.55 to 0.65) | 213.28 |
| Oceania | Ischemic heart disease | Male | 838 ( 654 to 1072 ) | 2488 ( 1954 to 3171 ) | 69.673 ( 54.086 to 90.979 ) | 80.072 ( 62.005 to 105.743 ) | 0.48 (0.46 to 0.51) | 196.897 |
| Oceania | Rheumatic heart disease | Female | 452 ( 349 to 583 ) | 877 ( 673 to 1129 ) | 14.057 ( 11.316 to 17.434 ) | 12.861 ( 10.145 to 16.04 ) | -0.36 (-0.39 to -0.34) | 94.027 |
| Oceania | Rheumatic heart disease | Male | 500 ( 388 to 647 ) | 901 ( 705 to 1146 ) | 16.269 ( 13.262 to 20.015 ) | 13.691 ( 11.058 to 16.844 ) | -0.66 (-0.7 to -0.62) | 80.2 |
| South Asia | Hypertensive heart disease | Female | 94203 ( 74017 to 118115 ) | 310845 ( 233868 to 407196 ) | 43.127 ( 33.65 to 54.833 ) | 46.056 ( 34.657 to 60.195 ) | 0.22 (0.2 to 0.24) | 229.974 |
| South Asia | Hypertensive heart disease | Male | 59139 ( 44905 to 75457 ) | 155844 ( 112488 to 206773 ) | 25.233 ( 18.96 to 32.399 ) | 25.106 ( 18.24 to 33.564 ) | -0.14 (-0.18 to -0.1) | 163.522 |
| South Asia | Ischemic heart disease | Female | 113061 ( 91590 to 137068 ) | 383846 ( 302626 to 477320 ) | 45.324 ( 36.509 to 55.944 ) | 54.331 ( 42.418 to 68.22 ) | 0.72 (0.67 to 0.78) | 239.503 |
| South Asia | Ischemic heart disease | Male | 177950 ( 145190 to 214592 ) | 561675 ( 453053 to 685542 ) | 67.339 ( 54.07 to 82.822 ) | 85.76 ( 68.763 to 106.099 ) | 0.97 (0.92 to 1.02) | 215.636 |
| South Asia | Rheumatic heart disease | Female | 114558 ( 94311 to 139688 ) | 193504 ( 159226 to 237327 ) | 24.087 ( 20.135 to 28.867 ) | 22.068 ( 18.185 to 26.93 ) | -0.32 (-0.35 to -0.29) | 68.914 |
| South Asia | Rheumatic heart disease | Male | 125522 ( 102632 to 153376 ) | 182826 ( 149746 to 225434 ) | 23.409 ( 19.591 to 28.109 ) | 19.891 ( 16.414 to 24.476 ) | -0.6 (-0.63 to -0.58) | 45.653 |
| Southeast Asia | Hypertensive heart disease | Female | 66074 ( 52514 to 84347 ) | 175726 ( 136806 to 223653 ) | 55.522 ( 43.826 to 70.582 ) | 54.524 ( 42.805 to 70.045 ) | -0.14 (-0.18 to -0.1) | 165.953 |
| Southeast Asia | Hypertensive heart disease | Male | 54598 ( 42703 to 68355 ) | 137025 ( 104857 to 175078 ) | 53.361 ( 40.994 to 67.855 ) | 51.057 ( 38.822 to 65.897 ) | -0.25 (-0.31 to -0.2) | 150.971 |
| Southeast Asia | Ischemic heart disease | Female | 55122 ( 44129 to 69420 ) | 169941 ( 132877 to 215959 ) | 46.426 ( 36.257 to 59.156 ) | 54.279 ( 42.237 to 69.641 ) | 0.62 (0.58 to 0.66) | 208.3 |
| Southeast Asia | Ischemic heart disease | Male | 61291 ( 49430 to 76607 ) | 185556 ( 146725 to 231862 ) | 60.303 ( 47.679 to 76.498 ) | 72.095 ( 56.807 to 90.833 ) | 0.7 (0.66 to 0.74) | 202.746 |
| Southeast Asia | Rheumatic heart disease | Female | 15408 ( 12680 to 18880 ) | 14806 ( 12310 to 17884 ) | 6.149 ( 5.109 to 7.358 ) | 4.43 ( 3.652 to 5.368 ) | -1.29 (-1.36 to -1.21) | -3.907 |
| Southeast Asia | Rheumatic heart disease | Male | 12503 ( 10141 to 15451 ) | 10966 ( 8986 to 13524 ) | 4.816 ( 3.95 to 5.814 ) | 3.322 ( 2.727 to 4.089 ) | -1.43 (-1.5 to -1.36) | -12.293 |
| Southern Latin America | Hypertensive heart disease | Female | 6928 ( 4992 to 9256 ) | 17601 ( 12167 to 24166 ) | 27.158 ( 19.816 to 36.163 ) | 33.052 ( 23.43 to 45.242 ) | 0.91 (0.85 to 0.97) | 154.056 |
| Southern Latin America | Hypertensive heart disease | Male | 5783 ( 4124 to 7846 ) | 14588 ( 10225 to 19882 ) | 30.66 ( 21.798 to 41.866 ) | 39.521 ( 27.878 to 53.9 ) | 1.05 (1 to 1.1) | 152.257 |
| Southern Latin America | Ischemic heart disease | Female | 9642 ( 7061 to 12927 ) | 15014 ( 10440 to 20596 ) | 38.665 ( 28.292 to 50.993 ) | 28.866 ( 20.317 to 39.031 ) | -1.19 (-1.29 to -1.09) | 55.715 |
| Southern Latin America | Ischemic heart disease | Male | 11854 ( 8741 to 15784 ) | 20270 ( 14403 to 27166 ) | 63.408 ( 46.329 to 86.021 ) | 54.24 ( 38.411 to 71.989 ) | -0.66 (-0.75 to -0.57) | 70.997 |
| Southern Latin America | Rheumatic heart disease | Female | 1415 ( 1132 to 1777 ) | 949 ( 727 to 1203 ) | 5.531 ( 4.465 to 6.864 ) | 2.154 ( 1.699 to 2.658 ) | -3.61 (-3.91 to -3.31) | -32.933 |
| Southern Latin America | Rheumatic heart disease | Male | 997 ( 786 to 1246 ) | 531 ( 412 to 675 ) | 4.502 ( 3.531 to 5.656 ) | 1.528 ( 1.191 to 1.902 ) | -4.05 (-4.43 to -3.67) | -46.74 |
| Southern Sub-Saharan Africa | Hypertensive heart disease | Female | 8202 ( 6039 to 10795 ) | 19489 ( 14407 to 25525 ) | 57.81 ( 42.291 to 76.696 ) | 63.747 ( 47.092 to 84.204 ) | 0.4 (0.33 to 0.47) | 137.613 |
| Southern Sub-Saharan Africa | Hypertensive heart disease | Male | 7433 ( 5403 to 9824 ) | 15687 ( 11310 to 20828 ) | 73.409 ( 53.525 to 97.725 ) | 76.367 ( 55.655 to 101.676 ) | 0.06 (0.01 to 0.11) | 111.045 |
| Southern Sub-Saharan Africa | Ischemic heart disease | Female | 6522 ( 4856 to 8610 ) | 13936 ( 10463 to 18517 ) | 46.572 ( 34.426 to 61.345 ) | 46.464 ( 34.5 to 62.164 ) | -0.09 (-0.16 to -0.01) | 113.677 |
| Southern Sub-Saharan Africa | Ischemic heart disease | Male | 7374 ( 5620 to 9409 ) | 15568 ( 11899 to 19904 ) | 76.748 ( 58.227 to 99.609 ) | 80.746 ( 60.593 to 103.568 ) | 0.27 (0.18 to 0.36) | 111.12 |
| Southern Sub-Saharan Africa | Rheumatic heart disease | Female | 3123 ( 2283 to 4158 ) | 3368 ( 2462 to 4488 ) | 9.72 ( 7.329 to 12.896 ) | 8.076 ( 5.967 to 10.744 ) | -0.66 (-0.72 to -0.6) | 7.845 |
| Southern Sub-Saharan Africa | Rheumatic heart disease | Male | 2851 ( 2066 to 3890 ) | 2965 ( 2076 to 4136 ) | 9.096 ( 6.748 to 12.107 ) | 7.242 ( 5.167 to 9.962 ) | -0.73 (-0.78 to -0.69) | 3.999 |
| Tropical Latin America | Hypertensive heart disease | Female | 22305 ( 17487 to 28129 ) | 75687 ( 57397 to 99959 ) | 49.641 ( 38.554 to 63.108 ) | 53.412 ( 40.593 to 70.496 ) | 0.25 (0.22 to 0.28) | 239.328 |
| Tropical Latin America | Hypertensive heart disease | Male | 17681 ( 13491 to 22730 ) | 58285 ( 43264 to 77786 ) | 48.118 ( 36.622 to 62.167 ) | 55.297 ( 41.416 to 74.409 ) | 0.41 (0.36 to 0.46) | 229.648 |
| Tropical Latin America | Ischemic heart disease | Female | 21665 ( 17144 to 27047 ) | 67221 ( 51677 to 85991 ) | 49.55 ( 38.961 to 62.771 ) | 47.465 ( 36.389 to 60.732 ) | -0.04 (-0.09 to 0.01) | 210.275 |
| Tropical Latin America | Ischemic heart disease | Male | 25933 ( 20703 to 31736 ) | 73607 ( 57380 to 93566 ) | 69.437 ( 54.786 to 86.508 ) | 67.834 ( 52.65 to 86.41 ) | 0.04 (-0.02 to 0.09) | 183.835 |
| Tropical Latin America | Rheumatic heart disease | Female | 5842 ( 4570 to 7447 ) | 5232 ( 4088 to 6573 ) | 7.163 ( 5.746 to 8.999 ) | 4.416 ( 3.428 to 5.557 ) | -1.73 (-1.79 to -1.68) | -10.442 |
| Tropical Latin America | Rheumatic heart disease | Male | 4777 ( 3653 to 6208 ) | 3035 ( 2335 to 3871 ) | 5.714 ( 4.466 to 7.286 ) | 2.935 ( 2.282 to 3.73 ) | -2.52 (-2.64 to -2.4) | -36.466 |
| Western Europe | Hypertensive heart disease | Female | 88968 ( 65669 to 120480 ) | 224731 ( 165812 to 295258 ) | 23.397 ( 17.477 to 31.03 ) | 34.333 ( 26.041 to 43.975 ) | 2.06 (1.71 to 2.4) | 152.598 |
| Western Europe | Hypertensive heart disease | Male | 39475 ( 27654 to 52829 ) | 131967 ( 97741 to 180078 ) | 17.517 ( 12.437 to 23.195 ) | 30.032 ( 22.459 to 40.359 ) | 2.09 (1.84 to 2.34) | 234.305 |
| Western Europe | Ischemic heart disease | Female | 248752 ( 185600 to 328239 ) | 345583 ( 262176 to 451472 ) | 66.196 ( 50.453 to 87.115 ) | 54.853 ( 41.89 to 70.829 ) | -0.8 (-0.97 to -0.62) | 38.927 |
| Western Europe | Ischemic heart disease | Male | 235936 ( 169507 to 310848 ) | 412724 ( 314488 to 539305 ) | 103.493 ( 75.493 to 135.515 ) | 95.707 ( 74.259 to 124.196 ) | -0.45 (-0.61 to -0.3) | 74.93 |
| Western Europe | Rheumatic heart disease | Female | 18063 ( 14024 to 23102 ) | 26701 ( 20873 to 33002 ) | 5.721 ( 4.549 to 7.145 ) | 5.428 ( 4.399 to 6.628 ) | 0.08 (-0.02 to 0.19) | 47.822 |
| Western Europe | Rheumatic heart disease | Male | 6455 ( 5017 to 8200 ) | 13329 ( 10315 to 16867 ) | 3.004 ( 2.382 to 3.77 ) | 3.51 ( 2.829 to 4.321 ) | 0.42 (0.36 to 0.48) | 106.491 |
| Western Sub-Saharan Africa | Hypertensive heart disease | Female | 28431 ( 21288 to 36858 ) | 66732 ( 50610 to 85401 ) | 73.288 ( 55.36 to 94.588 ) | 75.117 ( 55.876 to 97.712 ) | 0.12 (0.06 to 0.18) | 134.716 |
| Western Sub-Saharan Africa | Hypertensive heart disease | Male | 35435 ( 26920 to 45674 ) | 80074 ( 61310 to 102118 ) | 95.679 ( 72.937 to 123.6 ) | 99.942 ( 75.817 to 130.88 ) | 0.17 (0.14 to 0.19) | 125.974 |
| Western Sub-Saharan Africa | Ischemic heart disease | Female | 9828 ( 7089 to 13296 ) | 29531 ( 22114 to 38811 ) | 25.606 ( 18.373 to 34.868 ) | 33.827 ( 24.24 to 45.697 ) | 0.97 (0.94 to 1) | 200.478 |
| Western Sub-Saharan Africa | Ischemic heart disease | Male | 13627 ( 10048 to 18060 ) | 34756 ( 25707 to 45472 ) | 38.237 ( 27.56 to 51.997 ) | 44.648 ( 32.416 to 60.463 ) | 0.53 (0.51 to 0.56) | 155.052 |
| Western Sub-Saharan Africa | Rheumatic heart disease | Female | 6536 ( 4692 to 8986 ) | 9997 ( 6966 to 14021 ) | 5.852 ( 4.295 to 7.965 ) | 3.76 ( 2.718 to 5.178 ) | -1.58 (-1.65 to -1.51) | 52.953 |
| Western Sub-Saharan Africa | Rheumatic heart disease | Male | 7296 ( 5239 to 10052 ) | 12892 ( 8977 to 17878 ) | 6.72 ( 4.941 to 9.128 ) | 4.868 ( 3.5 to 6.618 ) | -1.25 (-1.34 to -1.17) | 76.7 |

**Table S2 The female and male YLDs of SHF due to HHD, IHD and RHD in 1990 and 2021.**

| Locations | cause | sex | 1990 YLDs cases(95% UI) | 2021 YLDs cases(95% UI) | 1990 ASYR(95% UI) | 2021 ASYR(95% UI) | 1990-2021 EAPC（95%CI） | 1990-2021 YLDs cases changes |
| --- | --- | --- | --- | --- | --- | --- | --- | --- |
| Global | Hypertensive heart disease | Female | 139207 ( 88319 to 201391 ) | 379822 ( 239976 to 549704 ) | 6.735 ( 4.239 to 9.774 ) | 8.19 ( 5.177 to 11.85 ) | 0.69 (0.66 to 0.72) | 172.847 |
| Global | Hypertensive heart disease | Male | 119856 ( 76035 to 173740 ) | 320450 ( 201201 to 480166 ) | 7.289 ( 4.606 to 10.622 ) | 8.346 ( 5.178 to 12.334 ) | 0.4 (0.34 to 0.46) | 167.363 |
| Global | Ischemic heart disease | Female | 216957 ( 138211 to 324050 ) | 496370 ( 315211 to 721980 ) | 10.782 ( 6.853 to 16.063 ) | 10.699 ( 6.81 to 15.543 ) | -0.05 (-0.08 to -0.03) | 128.787 |
| Global | Ischemic heart disease | Male | 231461 ( 146264 to 341641 ) | 579142 ( 370222 to 852044 ) | 14.409 ( 9.135 to 21.635 ) | 15.246 ( 9.78 to 22.161 ) | 0.22 (0.17 to 0.28) | 150.211 |
| Global | Rheumatic heart disease | Female | 50387 ( 32428 to 72775 ) | 71664 ( 45799 to 103317 ) | 1.987 ( 1.289 to 2.869 ) | 1.737 ( 1.114 to 2.525 ) | -0.52 (-0.55 to -0.49) | 42.227 |
| Global | Rheumatic heart disease | Male | 42847 ( 27620 to 62127 ) | 57491 ( 36341 to 83598 ) | 1.694 ( 1.099 to 2.434 ) | 1.474 ( 0.94 to 2.149 ) | -0.55 (-0.58 to -0.52) | 34.177 |
| High SDI | Hypertensive heart disease | Female | 29257 ( 17850 to 43019 ) | 77639 ( 46822 to 114411 ) | 4.44 ( 2.763 to 6.473 ) | 6.181 ( 3.861 to 8.958 ) | 1.5 (1.37 to 1.64) | 165.369 |
| High SDI | Hypertensive heart disease | Male | 18630 ( 11550 to 27128 ) | 59940 ( 36150 to 88867 ) | 4.087 ( 2.535 to 5.926 ) | 6.531 ( 4.011 to 9.637 ) | 1.69 (1.59 to 1.8) | 221.739 |
| High SDI | Ischemic heart disease | Female | 87818 ( 54511 to 132012 ) | 142344 ( 91410 to 207608 ) | 12.831 ( 8.106 to 19.266 ) | 10.859 ( 7.021 to 15.814 ) | -0.77 (-0.86 to -0.67) | 62.09 |
| High SDI | Ischemic heart disease | Male | 85568 ( 53628 to 127917 ) | 162569 ( 102047 to 237040 ) | 19.124 ( 11.927 to 28.802 ) | 17.03 ( 10.929 to 24.735 ) | -0.59 (-0.66 to -0.51) | 89.988 |
| High SDI | Rheumatic heart disease | Female | 5705 ( 3554 to 8652 ) | 7449 ( 4591 to 11111 ) | 0.968 ( 0.617 to 1.434 ) | 0.728 ( 0.453 to 1.062 ) | -0.9 (-0.99 to -0.81) | 30.57 |
| High SDI | Rheumatic heart disease | Male | 2494 ( 1570 to 3685 ) | 3851 ( 2462 to 5867 ) | 0.561 ( 0.353 to 0.83 ) | 0.476 ( 0.305 to 0.701 ) | -0.67 (-0.79 to -0.54) | 54.411 |
| High-middle SDI | Hypertensive heart disease | Female | 30674 ( 18797 to 44885 ) | 84314 ( 51960 to 124610 ) | 5.59 ( 3.462 to 8.176 ) | 7.519 ( 4.658 to 11.088 ) | 1.01 (0.95 to 1.06) | 174.871 |
| High-middle SDI | Hypertensive heart disease | Male | 25063 ( 15468 to 36739 ) | 69431 ( 43070 to 105513 ) | 6.658 ( 4.08 to 9.705 ) | 8.175 ( 5.03 to 12.337 ) | 0.67 (0.61 to 0.72) | 177.026 |
| High-middle SDI | Ischemic heart disease | Female | 63631 ( 40312 to 95390 ) | 137699 ( 87113 to 202906 ) | 11.821 ( 7.581 to 17.665 ) | 12.241 ( 7.774 to 17.963 ) | 0.18 (0.15 to 0.21) | 116.402 |
| High-middle SDI | Ischemic heart disease | Male | 55652 ( 34849 to 82212 ) | 137176 ( 86806 to 202923 ) | 14.859 ( 9.363 to 22.333 ) | 16.168 ( 10.31 to 23.693 ) | 0.43 (0.34 to 0.53) | 146.489 |
| High-middle SDI | Rheumatic heart disease | Female | 7801 ( 5027 to 11228 ) | 10279 ( 6135 to 15548 ) | 1.405 ( 0.909 to 2.031 ) | 1.087 ( 0.675 to 1.619 ) | -1.13 (-1.23 to -1.03) | 31.765 |
| High-middle SDI | Rheumatic heart disease | Male | 4600 ( 2946 to 6718 ) | 5699 ( 3434 to 8623 ) | 1 ( 0.642 to 1.458 ) | 0.73 ( 0.449 to 1.105 ) | -1.28 (-1.35 to -1.2) | 23.891 |
| Low SDI | Hypertensive heart disease | Female | 9550 ( 6080 to 13951 ) | 24830 ( 16271 to 36097 ) | 10.371 ( 6.632 to 15.033 ) | 10.975 ( 7.147 to 16.11 ) | 0.23 (0.21 to 0.25) | 160 |
| Low SDI | Hypertensive heart disease | Male | 11312 ( 7160 to 16522 ) | 26280 ( 17047 to 38995 ) | 11.873 ( 7.517 to 17.299 ) | 12.118 ( 7.744 to 17.82 ) | 0.06 (0.02 to 0.09) | 132.32 |
| Low SDI | Ischemic heart disease | Female | 5639 ( 3657 to 8335 ) | 15477 ( 9831 to 23032 ) | 5.875 ( 3.804 to 8.657 ) | 6.676 ( 4.275 to 10.007 ) | 0.42 (0.4 to 0.43) | 174.464 |
| Low SDI | Ischemic heart disease | Male | 8656 ( 5473 to 12777 ) | 23165 ( 15305 to 34228 ) | 9.095 ( 5.867 to 13.621 ) | 10.677 ( 6.959 to 15.561 ) | 0.63 (0.59 to 0.66) | 167.618 |
| Low SDI | Rheumatic heart disease | Female | 5024 ( 3161 to 7524 ) | 9124 ( 5785 to 13841 ) | 2.096 ( 1.299 to 3.058 ) | 1.798 ( 1.139 to 2.66 ) | -0.44 (-0.47 to -0.41) | 81.608 |
| Low SDI | Rheumatic heart disease | Male | 5452 ( 3496 to 8155 ) | 9272 ( 5824 to 13833 ) | 2.239 ( 1.447 to 3.26 ) | 1.769 ( 1.122 to 2.584 ) | -0.79 (-0.81 to -0.76) | 70.066 |
| Low-middle SDI | Hypertensive heart disease | Female | 21953 ( 13917 to 32437 ) | 61373 ( 39016 to 90206 ) | 8.574 ( 5.5 to 12.383 ) | 9.005 ( 5.727 to 13.069 ) | 0.14 (0.13 to 0.15) | 179.565 |
| Low-middle SDI | Hypertensive heart disease | Male | 19151 ( 12285 to 27941 ) | 46664 ( 29518 to 69272 ) | 7.373 ( 4.671 to 10.616 ) | 7.614 ( 4.751 to 11.154 ) | 0.02 (-0.01 to 0.04) | 143.664 |
| Low-middle SDI | Ischemic heart disease | Female | 20714 ( 13447 to 29996 ) | 60720 ( 38817 to 89370 ) | 7.605 ( 4.886 to 11.079 ) | 8.686 ( 5.591 to 12.669 ) | 0.51 (0.47 to 0.55) | 193.135 |
| Low-middle SDI | Ischemic heart disease | Male | 30501 ( 19891 to 44280 ) | 83994 ( 54766 to 122848 ) | 11.06 ( 7.19 to 16.13 ) | 13.347 ( 8.693 to 19.432 ) | 0.73 (0.7 to 0.77) | 175.381 |
| Low-middle SDI | Rheumatic heart disease | Female | 16296 ( 10551 to 23663 ) | 24136 ( 15359 to 35277 ) | 3.05 ( 1.97 to 4.384 ) | 2.629 ( 1.673 to 3.824 ) | -0.52 (-0.56 to -0.49) | 48.11 |
| Low-middle SDI | Rheumatic heart disease | Male | 17293 ( 11164 to 25506 ) | 22777 ( 14376 to 33248 ) | 3.034 ( 1.939 to 4.398 ) | 2.43 ( 1.549 to 3.533 ) | -0.83 (-0.87 to -0.79) | 31.712 |
| Middle SDI | Hypertensive heart disease | Female | 47621 ( 29673 to 69184 ) | 131288 ( 82850 to 191813 ) | 10.17 ( 6.369 to 14.769 ) | 9.727 ( 6.151 to 14.184 ) | -0.31 (-0.45 to -0.17) | 175.693 |
| Middle SDI | Hypertensive heart disease | Male | 45579 ( 28739 to 66819 ) | 117813 ( 72866 to 176944 ) | 10.93 ( 6.779 to 15.885 ) | 10.119 ( 6.207 to 14.959 ) | -0.36 (-0.47 to -0.25) | 158.481 |
| Middle SDI | Ischemic heart disease | Female | 38852 ( 25249 to 57300 ) | 139616 ( 87910 to 205043 ) | 8.317 ( 5.321 to 12.148 ) | 10.452 ( 6.634 to 15.288 ) | 0.91 (0.87 to 0.96) | 259.353 |
| Middle SDI | Ischemic heart disease | Male | 50726 ( 33155 to 73807 ) | 171652 ( 110986 to 255017 ) | 12.046 ( 7.939 to 17.674 ) | 14.805 ( 9.51 to 21.619 ) | 0.91 (0.82 to 1.01) | 238.391 |
| Middle SDI | Rheumatic heart disease | Female | 15523 ( 9891 to 22357 ) | 20640 ( 13079 to 30331 ) | 2.067 ( 1.334 to 2.993 ) | 1.625 ( 1.031 to 2.34 ) | -0.92 (-0.96 to -0.88) | 32.964 |
| Middle SDI | Rheumatic heart disease | Male | 12978 ( 8345 to 18848 ) | 15866 ( 9892 to 22636 ) | 1.678 ( 1.083 to 2.417 ) | 1.328 ( 0.828 to 1.907 ) | -0.85 (-0.89 to -0.81) | 22.253 |
| Andean Latin America | Hypertensive heart disease | Female | 896 ( 566 to 1327 ) | 2565 ( 1602 to 3801 ) | 8.667 ( 5.421 to 12.879 ) | 8.324 ( 5.163 to 12.436 ) | 0.05 (-0.03 to 0.14) | 186.272 |
| Andean Latin America | Hypertensive heart disease | Male | 763 ( 485 to 1113 ) | 2273 ( 1387 to 3465 ) | 8.127 ( 5.129 to 11.946 ) | 8.362 ( 5.077 to 12.842 ) | 0.35 (0.23 to 0.47) | 197.903 |
| Andean Latin America | Ischemic heart disease | Female | 1054 ( 654 to 1571 ) | 3380 ( 2099 to 5045 ) | 10.285 ( 6.359 to 15.609 ) | 10.964 ( 6.805 to 16.354 ) | 0 (-0.14 to 0.14) | 220.683 |
| Andean Latin America | Ischemic heart disease | Male | 1458 ( 917 to 2166 ) | 4533 ( 2819 to 6927 ) | 15.413 ( 9.533 to 22.766 ) | 16.624 ( 10.452 to 25.176 ) | 0.03 (-0.11 to 0.18) | 210.905 |
| Andean Latin America | Rheumatic heart disease | Female | 175 ( 112 to 257 ) | 198 ( 124 to 286 ) | 1.074 ( 0.672 to 1.55 ) | 0.611 ( 0.388 to 0.885 ) | -1.87 (-1.97 to -1.78) | 13.143 |
| Andean Latin America | Rheumatic heart disease | Male | 119 ( 75 to 178 ) | 125 ( 78 to 184 ) | 0.704 ( 0.453 to 1.048 ) | 0.4 ( 0.252 to 0.592 ) | -2.02 (-2.11 to -1.92) | 5.042 |
| Australasia | Hypertensive heart disease | Female | 259 ( 157 to 382 ) | 918 ( 544 to 1330 ) | 1.876 ( 1.148 to 2.755 ) | 2.847 ( 1.702 to 4.125 ) | 1.77 (1.65 to 1.89) | 254.44 |
| Australasia | Hypertensive heart disease | Male | 154 ( 91 to 237 ) | 777 ( 464 to 1177 ) | 1.541 ( 0.903 to 2.348 ) | 3.026 ( 1.837 to 4.549 ) | 2.28 (2.13 to 2.42) | 404.545 |
| Australasia | Ischemic heart disease | Female | 2403 ( 1467 to 3570 ) | 4348 ( 2787 to 6358 ) | 17.233 ( 10.613 to 25.423 ) | 13.402 ( 8.599 to 19.738 ) | -1.58 (-1.85 to -1.32) | 80.94 |
| Australasia | Ischemic heart disease | Male | 2740 ( 1659 to 4129 ) | 5669 ( 3517 to 8594 ) | 27.411 ( 16.809 to 40.889 ) | 21.784 ( 13.657 to 32.801 ) | -1.32 (-1.51 to -1.13) | 106.898 |
| Australasia | Rheumatic heart disease | Female | 139 ( 87 to 208 ) | 275 ( 169 to 408 ) | 1.168 ( 0.739 to 1.724 ) | 1.174 ( 0.734 to 1.705 ) | 0.26 (0.08 to 0.45) | 97.842 |
| Australasia | Rheumatic heart disease | Male | 74 ( 47 to 110 ) | 157 ( 95 to 249 ) | 0.744 ( 0.477 to 1.093 ) | 0.793 ( 0.489 to 1.214 ) | 0.1 (-0.05 to 0.25) | 112.162 |
| Caribbean | Hypertensive heart disease | Female | 1025 ( 646 to 1531 ) | 2920 ( 1871 to 4377 ) | 7.675 ( 4.862 to 11.397 ) | 9.996 ( 6.396 to 14.982 ) | 1.05 (0.99 to 1.12) | 184.878 |
| Caribbean | Hypertensive heart disease | Male | 1002 ( 618 to 1492 ) | 2995 ( 1813 to 4539 ) | 8.244 ( 5.103 to 12.13 ) | 12.218 ( 7.378 to 18.525 ) | 1.44 (1.38 to 1.5) | 198.902 |
| Caribbean | Ischemic heart disease | Female | 1915 ( 1167 to 2938 ) | 3454 ( 2138 to 5268 ) | 14.685 ( 8.915 to 22.407 ) | 11.61 ( 7.169 to 17.812 ) | -0.89 (-0.94 to -0.84) | 80.366 |
| Caribbean | Ischemic heart disease | Male | 2634 ( 1629 to 3928 ) | 4321 ( 2711 to 6474 ) | 22.247 ( 13.705 to 33.165 ) | 17.663 ( 11.094 to 26.472 ) | -0.9 (-0.95 to -0.84) | 64.047 |
| Caribbean | Rheumatic heart disease | Female | 311 ( 193 to 468 ) | 264 ( 162 to 404 ) | 1.658 ( 1.045 to 2.469 ) | 1.145 ( 0.693 to 1.776 ) | -1.38 (-1.46 to -1.31) | -15.113 |
| Caribbean | Rheumatic heart disease | Male | 234 ( 140 to 352 ) | 191 ( 116 to 292 ) | 1.233 ( 0.749 to 1.859 ) | 0.855 ( 0.51 to 1.326 ) | -1.49 (-1.61 to -1.37) | -18.376 |
| Central Asia | Hypertensive heart disease | Female | 1191 ( 713 to 1778 ) | 2075 ( 1202 to 3222 ) | 4.383 ( 2.631 to 6.569 ) | 5 ( 2.857 to 7.736 ) | 0.95 (0.74 to 1.16) | 74.223 |
| Central Asia | Hypertensive heart disease | Male | 780 ( 467 to 1175 ) | 1830 ( 1032 to 2933 ) | 4.488 ( 2.569 to 7.032 ) | 5.699 ( 3.006 to 9.109 ) | 0.91 (0.69 to 1.13) | 134.615 |
| Central Asia | Ischemic heart disease | Female | 4461 ( 2668 to 6884 ) | 5838 ( 3460 to 8964 ) | 16.651 ( 9.909 to 25.646 ) | 14.591 ( 8.589 to 22.475 ) | -0.62 (-0.74 to -0.49) | 30.868 |
| Central Asia | Ischemic heart disease | Male | 3403 ( 1982 to 5209 ) | 5341 ( 3067 to 8177 ) | 20.547 ( 11.468 to 32.322 ) | 17.493 ( 9.474 to 27.212 ) | -0.71 (-0.84 to -0.58) | 56.95 |
| Central Asia | Rheumatic heart disease | Female | 632 ( 384 to 962 ) | 465 ( 284 to 721 ) | 1.715 ( 1.069 to 2.606 ) | 1.014 ( 0.615 to 1.575 ) | -1.71 (-1.83 to -1.58) | -26.424 |
| Central Asia | Rheumatic heart disease | Male | 542 ( 330 to 825 ) | 403 ( 239 to 627 ) | 1.476 ( 0.924 to 2.225 ) | 0.882 ( 0.527 to 1.357 ) | -1.92 (-2.07 to -1.77) | -25.646 |
| Central Europe | Hypertensive heart disease | Female | 4639 ( 2696 to 6919 ) | 10926 ( 6438 to 16104 ) | 5.464 ( 3.209 to 8.089 ) | 7.663 ( 4.588 to 11.199 ) | 1.55 (1.42 to 1.69) | 135.525 |
| Central Europe | Hypertensive heart disease | Male | 3415 ( 2020 to 5242 ) | 8177 ( 4692 to 12261 ) | 5.759 ( 3.407 to 8.758 ) | 8.772 ( 5.059 to 12.961 ) | 1.87 (1.7 to 2.03) | 139.444 |
| Central Europe | Ischemic heart disease | Female | 11003 ( 6714 to 16742 ) | 17755 ( 11072 to 26246 ) | 13.283 ( 8.237 to 20.044 ) | 12.105 ( 7.644 to 17.899 ) | -0.36 (-0.46 to -0.26) | 61.365 |
| Central Europe | Ischemic heart disease | Male | 12366 ( 7603 to 18609 ) | 17879 ( 11137 to 26447 ) | 21.5 ( 13.361 to 32.206 ) | 19.199 ( 12.106 to 28.449 ) | -0.46 (-0.51 to -0.4) | 44.582 |
| Central Europe | Rheumatic heart disease | Female | 837 ( 525 to 1249 ) | 556 ( 341 to 844 ) | 1.088 ( 0.687 to 1.611 ) | 0.556 ( 0.351 to 0.822 ) | -2.31 (-2.54 to -2.09) | -33.572 |
| Central Europe | Rheumatic heart disease | Male | 608 ( 385 to 899 ) | 302 ( 189 to 458 ) | 0.97 ( 0.616 to 1.436 ) | 0.403 ( 0.255 to 0.586 ) | -3.14 (-3.44 to -2.85) | -50.329 |
| Central Latin America | Hypertensive heart disease | Female | 3218 ( 2003 to 4661 ) | 9312 ( 5834 to 13766 ) | 8.062 ( 5.002 to 11.655 ) | 7.05 ( 4.406 to 10.438 ) | -0.62 (-0.69 to -0.55) | 189.372 |
| Central Latin America | Hypertensive heart disease | Male | 2572 ( 1637 to 3750 ) | 6634 ( 4076 to 10023 ) | 7.179 ( 4.492 to 10.511 ) | 6.204 ( 3.779 to 9.406 ) | -0.7 (-0.76 to -0.63) | 157.932 |
| Central Latin America | Ischemic heart disease | Female | 4628 ( 3012 to 6709 ) | 16481 ( 10504 to 24326 ) | 11.449 ( 7.292 to 16.878 ) | 12.43 ( 7.88 to 18.294 ) | 0.34 (0.3 to 0.37) | 256.115 |
| Central Latin America | Ischemic heart disease | Male | 6258 ( 4085 to 9069 ) | 20185 ( 13150 to 29817 ) | 16.6 ( 10.612 to 24.527 ) | 18.455 ( 11.901 to 27.205 ) | 0.36 (0.32 to 0.41) | 222.547 |
| Central Latin America | Rheumatic heart disease | Female | 936 ( 593 to 1341 ) | 446 ( 281 to 665 ) | 1.283 ( 0.81 to 1.82 ) | 0.334 ( 0.21 to 0.497 ) | -4.85 (-5 to -4.69) | -52.35 |
| Central Latin America | Rheumatic heart disease | Male | 595 ( 382 to 875 ) | 235 ( 146 to 350 ) | 0.782 ( 0.499 to 1.12 ) | 0.198 ( 0.123 to 0.296 ) | -5.11 (-5.37 to -4.86) | -60.504 |
| Central Sub-Saharan Africa | Hypertensive heart disease | Female | 1006 ( 591 to 1544 ) | 2966 ( 1829 to 4461 ) | 10.871 ( 6.373 to 16.662 ) | 12.02 ( 7.249 to 18.115 ) | 0.35 (0.3 to 0.4) | 194.831 |
| Central Sub-Saharan Africa | Hypertensive heart disease | Male | 1171 ( 695 to 1741 ) | 3024 ( 1824 to 4769 ) | 13.963 ( 8.45 to 21.298 ) | 15.225 ( 9.139 to 23.364 ) | 0.31 (0.26 to 0.37) | 158.241 |
| Central Sub-Saharan Africa | Ischemic heart disease | Female | 560 ( 331 to 864 ) | 1641 ( 992 to 2518 ) | 6.273 ( 3.762 to 9.74 ) | 6.818 ( 4.211 to 10.61 ) | 0.2 (0.17 to 0.22) | 193.036 |
| Central Sub-Saharan Africa | Ischemic heart disease | Male | 708 ( 426 to 1125 ) | 1782 ( 1051 to 2798 ) | 9.1 ( 5.59 to 14.379 ) | 9.726 ( 5.833 to 15.22 ) | 0.15 (0.12 to 0.18) | 151.695 |
| Central Sub-Saharan Africa | Rheumatic heart disease | Female | 365 ( 205 to 600 ) | 641 ( 372 to 1008 ) | 1.128 ( 0.648 to 1.87 ) | 0.856 ( 0.521 to 1.363 ) | -1.02 (-1.09 to -0.95) | 75.616 |
| Central Sub-Saharan Africa | Rheumatic heart disease | Male | 347 ( 187 to 564 ) | 585 ( 332 to 937 ) | 1.047 ( 0.58 to 1.67 ) | 0.762 ( 0.446 to 1.23 ) | -1.22 (-1.29 to -1.14) | 68.588 |
| East Asia | Hypertensive heart disease | Female | 42400 ( 25504 to 62714 ) | 113937 ( 71300 to 170711 ) | 11.059 ( 6.741 to 16.1 ) | 10.1 ( 6.334 to 15.11 ) | -0.61 (-0.88 to -0.35) | 168.719 |
| East Asia | Hypertensive heart disease | Male | 44452 ( 27321 to 66300 ) | 113663 ( 69338 to 172907 ) | 13.121 ( 7.933 to 19.305 ) | 11.508 ( 6.987 to 17.332 ) | -0.6 (-0.79 to -0.41) | 155.698 |
| East Asia | Ischemic heart disease | Female | 27181 ( 17140 to 40750 ) | 126033 ( 79899 to 186341 ) | 7.161 ( 4.613 to 10.785 ) | 11.346 ( 7.235 to 16.671 ) | 1.84 (1.71 to 1.97) | 363.681 |
| East Asia | Ischemic heart disease | Male | 32229 ( 20538 to 47480 ) | 138462 ( 86894 to 208745 ) | 9.371 ( 5.98 to 14.042 ) | 14.239 ( 9.099 to 21.04 ) | 1.9 (1.66 to 2.14) | 329.619 |
| East Asia | Rheumatic heart disease | Female | 10908 ( 7032 to 15844 ) | 16414 ( 9718 to 25264 ) | 2.231 ( 1.443 to 3.273 ) | 1.535 ( 0.918 to 2.322 ) | -1.58 (-1.71 to -1.45) | 50.477 |
| East Asia | Rheumatic heart disease | Male | 7406 ( 4745 to 10941 ) | 10318 ( 6095 to 15576 ) | 1.604 ( 1.033 to 2.379 ) | 1.061 ( 0.635 to 1.609 ) | -1.55 (-1.65 to -1.46) | 39.319 |
| Eastern Europe | Hypertensive heart disease | Female | 3040 ( 1762 to 4695 ) | 5835 ( 3286 to 9420 ) | 1.65 ( 0.982 to 2.524 ) | 2.425 ( 1.368 to 3.893 ) | 1.68 (1.5 to 1.87) | 91.941 |
| Eastern Europe | Hypertensive heart disease | Male | 1337 ( 813 to 2091 ) | 2859 ( 1591 to 4520 ) | 1.44 ( 0.885 to 2.196 ) | 2.292 ( 1.273 to 3.634 ) | 1.91 (1.73 to 2.09) | 113.837 |
| Eastern Europe | Ischemic heart disease | Female | 32041 ( 20326 to 47557 ) | 39596 ( 24875 to 58724 ) | 17.411 ( 11.165 to 25.701 ) | 16.019 ( 10.07 to 23.839 ) | -0.22 (-0.3 to -0.14) | 23.579 |
| Eastern Europe | Ischemic heart disease | Male | 17046 ( 10673 to 25132 ) | 24654 ( 15286 to 36341 ) | 21.223 ( 12.92 to 31.375 ) | 20.216 ( 12.442 to 30.126 ) | -0.19 (-0.26 to -0.11) | 44.632 |
| Eastern Europe | Rheumatic heart disease | Female | 1191 ( 730 to 1811 ) | 606 ( 338 to 982 ) | 0.806 ( 0.514 to 1.191 ) | 0.324 ( 0.189 to 0.502 ) | -3.36 (-3.69 to -3.02) | -49.118 |
| Eastern Europe | Rheumatic heart disease | Male | 612 ( 387 to 899 ) | 168 ( 99 to 261 ) | 0.591 ( 0.376 to 0.855 ) | 0.158 ( 0.095 to 0.239 ) | -4.94 (-5.14 to -4.75) | -72.549 |
| Eastern Sub-Saharan Africa | Hypertensive heart disease | Female | 4039 ( 2536 to 5995 ) | 10460 ( 6711 to 15283 ) | 12.974 ( 8.112 to 18.98 ) | 13.559 ( 8.685 to 20.256 ) | 0.18 (0.16 to 0.2) | 158.975 |
| Eastern Sub-Saharan Africa | Hypertensive heart disease | Male | 5717 ( 3598 to 8379 ) | 13348 ( 8620 to 19937 ) | 18.367 ( 11.506 to 26.861 ) | 19.311 ( 12.414 to 28.205 ) | 0.08 (0.05 to 0.11) | 133.479 |
| Eastern Sub-Saharan Africa | Ischemic heart disease | Female | 1614 ( 1004 to 2494 ) | 4665 ( 2924 to 7200 ) | 5.367 ( 3.367 to 8.358 ) | 6.222 ( 3.925 to 9.658 ) | 0.47 (0.42 to 0.52) | 189.033 |
| Eastern Sub-Saharan Africa | Ischemic heart disease | Male | 2489 ( 1524 to 3887 ) | 6175 ( 3877 to 9310 ) | 8.557 ( 5.42 to 13.355 ) | 9.386 ( 6.032 to 14.642 ) | 0.27 (0.23 to 0.31) | 148.092 |
| Eastern Sub-Saharan Africa | Rheumatic heart disease | Female | 935 ( 543 to 1420 ) | 1525 ( 903 to 2342 ) | 0.846 ( 0.501 to 1.286 ) | 0.672 ( 0.41 to 1.048 ) | -0.72 (-0.76 to -0.68) | 63.102 |
| Eastern Sub-Saharan Africa | Rheumatic heart disease | Male | 741 ( 444 to 1182 ) | 1104 ( 637 to 1738 ) | 0.703 ( 0.426 to 1.083 ) | 0.518 ( 0.31 to 0.794 ) | -1.16 (-1.22 to -1.1) | 48.988 |
| High-income Asia Pacific | Hypertensive heart disease | Female | 3554 ( 2073 to 5372 ) | 10968 ( 6509 to 16651 ) | 3.336 ( 1.987 to 4.982 ) | 3.334 ( 2.02 to 4.912 ) | -0.13 (-0.3 to 0.04) | 208.61 |
| High-income Asia Pacific | Hypertensive heart disease | Male | 2417 ( 1478 to 3612 ) | 5743 ( 3469 to 8628 ) | 2.926 ( 1.751 to 4.437 ) | 2.999 ( 1.872 to 4.473 ) | -0.28 (-0.52 to -0.03) | 137.609 |
| High-income Asia Pacific | Ischemic heart disease | Female | 5902 ( 3549 to 9066 ) | 17523 ( 11305 to 26003 ) | 5.469 ( 3.324 to 8.368 ) | 5.683 ( 3.772 to 8.357 ) | -0.07 (-0.2 to 0.07) | 196.899 |
| High-income Asia Pacific | Ischemic heart disease | Male | 6883 ( 4292 to 10544 ) | 17596 ( 11400 to 25883 ) | 8.309 ( 5.152 to 12.55 ) | 9.308 ( 6.159 to 13.666 ) | 0.42 (0.3 to 0.54) | 155.644 |
| High-income Asia Pacific | Rheumatic heart disease | Female | 682 ( 415 to 1064 ) | 1090 ( 668 to 1667 ) | 0.648 ( 0.398 to 0.996 ) | 0.475 ( 0.296 to 0.709 ) | -1.12 (-1.2 to -1.04) | 59.824 |
| High-income Asia Pacific | Rheumatic heart disease | Male | 297 ( 186 to 448 ) | 456 ( 273 to 712 ) | 0.365 ( 0.227 to 0.559 ) | 0.277 ( 0.172 to 0.418 ) | -1.07 (-1.18 to -0.96) | 53.535 |
| High-income North America | Hypertensive heart disease | Female | 10451 ( 6244 to 15244 ) | 28310 ( 17488 to 41262 ) | 5.249 ( 3.226 to 7.69 ) | 7.973 ( 4.934 to 11.523 ) | 1.66 (1.53 to 1.8) | 170.883 |
| High-income North America | Hypertensive heart disease | Male | 7530 ( 4622 to 11373 ) | 25494 ( 15754 to 37405 ) | 5.039 ( 3.086 to 7.536 ) | 9.076 ( 5.626 to 13.096 ) | 2.22 (2.07 to 2.38) | 238.566 |
| High-income North America | Ischemic heart disease | Female | 34289 ( 21170 to 51596 ) | 51500 ( 33745 to 74370 ) | 15.673 ( 9.829 to 23.396 ) | 13.152 ( 8.607 to 19.136 ) | -0.94 (-1.06 to -0.82) | 50.194 |
| High-income North America | Ischemic heart disease | Male | 33335 ( 20511 to 50379 ) | 57738 ( 36616 to 84117 ) | 22.6 ( 13.906 to 34.125 ) | 19.262 ( 12.256 to 28.005 ) | -0.87 (-0.97 to -0.78) | 73.205 |
| High-income North America | Rheumatic heart disease | Female | 1703 ( 1032 to 2599 ) | 1830 ( 1120 to 2831 ) | 0.932 ( 0.583 to 1.37 ) | 0.595 ( 0.367 to 0.895 ) | -1.58 (-1.98 to -1.18) | 7.457 |
| High-income North America | Rheumatic heart disease | Male | 675 ( 416 to 1016 ) | 851 ( 517 to 1296 ) | 0.473 ( 0.295 to 0.696 ) | 0.358 ( 0.219 to 0.533 ) | -0.9 (-1.33 to -0.48) | 26.074 |
| North Africa and Middle East | Hypertensive heart disease | Female | 9652 ( 6160 to 14033 ) | 26482 ( 16714 to 38887 ) | 12.625 ( 7.99 to 18.373 ) | 12.657 ( 7.95 to 18.507 ) | 0.11 (0.07 to 0.14) | 174.368 |
| North Africa and Middle East | Hypertensive heart disease | Male | 10815 ( 6940 to 15700 ) | 31388 ( 20108 to 45489 ) | 14.004 ( 8.861 to 20.555 ) | 14.56 ( 9.192 to 21.43 ) | 0.18 (0.12 to 0.24) | 190.227 |
| North Africa and Middle East | Ischemic heart disease | Female | 9867 ( 6278 to 14472 ) | 28540 ( 17855 to 41651 ) | 12.4 ( 7.735 to 18.412 ) | 13.51 ( 8.628 to 19.878 ) | 0.2 (0.15 to 0.25) | 189.247 |
| North Africa and Middle East | Ischemic heart disease | Male | 17440 ( 11544 to 25305 ) | 49627 ( 32718 to 71786 ) | 20.923 ( 13.511 to 30.7 ) | 21.912 ( 14.487 to 31.773 ) | 0.13 (0.07 to 0.18) | 184.558 |
| North Africa and Middle East | Rheumatic heart disease | Female | 2758 ( 1709 to 4149 ) | 2991 ( 1888 to 4439 ) | 1.492 ( 0.949 to 2.206 ) | 1.016 ( 0.64 to 1.514 ) | -1.39 (-1.45 to -1.33) | 8.448 |
| North Africa and Middle East | Rheumatic heart disease | Male | 2438 ( 1525 to 3542 ) | 2698 ( 1685 to 4002 ) | 1.252 ( 0.797 to 1.822 ) | 0.868 ( 0.541 to 1.289 ) | -1.43 (-1.5 to -1.36) | 10.664 |
| Oceania | Hypertensive heart disease | Female | 87 ( 54 to 132 ) | 208 ( 129 to 319 ) | 7.348 ( 4.607 to 10.877 ) | 6.631 ( 4.035 to 10.288 ) | -0.44 (-0.52 to -0.36) | 139.08 |
| Oceania | Hypertensive heart disease | Male | 68 ( 42 to 103 ) | 172 ( 102 to 260 ) | 5.457 ( 3.465 to 8.335 ) | 5.312 ( 3.076 to 8.117 ) | -0.2 (-0.24 to -0.16) | 152.941 |
| Oceania | Ischemic heart disease | Female | 86 ( 53 to 127 ) | 267 ( 164 to 406 ) | 8.238 ( 5.047 to 12.486 ) | 9.82 ( 6.139 to 14.68 ) | 0.61 (0.56 to 0.66) | 210.465 |
| Oceania | Ischemic heart disease | Male | 145 ( 92 to 218 ) | 430 ( 274 to 642 ) | 11.904 ( 7.477 to 18.069 ) | 13.693 ( 8.765 to 20.344 ) | 0.49 (0.46 to 0.51) | 196.552 |
| Oceania | Rheumatic heart disease | Female | 79 ( 47 to 126 ) | 155 ( 87 to 255 ) | 2.454 ( 1.512 to 3.756 ) | 2.267 ( 1.314 to 3.575 ) | -0.34 (-0.37 to -0.3) | 96.203 |
| Oceania | Rheumatic heart disease | Male | 88 ( 52 to 138 ) | 158 ( 93 to 245 ) | 2.849 ( 1.785 to 4.296 ) | 2.39 ( 1.47 to 3.554 ) | -0.67 (-0.7 to -0.63) | 79.545 |
| South Asia | Hypertensive heart disease | Female | 15776 ( 9953 to 22991 ) | 52346 ( 32399 to 77658 ) | 7.137 ( 4.484 to 10.452 ) | 7.711 ( 4.845 to 11.307 ) | 0.26 (0.24 to 0.28) | 231.808 |
| South Asia | Hypertensive heart disease | Male | 9996 ( 6264 to 14751 ) | 26458 ( 16199 to 40531 ) | 4.209 ( 2.644 to 6.114 ) | 4.227 ( 2.567 to 6.302 ) | -0.1 (-0.14 to -0.07) | 164.686 |
| South Asia | Ischemic heart disease | Female | 19127 ( 12470 to 27454 ) | 64943 ( 42062 to 95134 ) | 7.561 ( 4.913 to 10.895 ) | 9.135 ( 5.919 to 13.338 ) | 0.75 (0.69 to 0.81) | 239.536 |
| South Asia | Ischemic heart disease | Male | 30311 ( 20086 to 43595 ) | 95935 ( 63927 to 141441 ) | 11.305 ( 7.407 to 16.307 ) | 14.518 ( 9.48 to 21.07 ) | 1 (0.95 to 1.05) | 216.502 |
| South Asia | Rheumatic heart disease | Female | 19850 ( 12777 to 29010 ) | 33458 ( 21410 to 48588 ) | 4.141 ( 2.691 to 5.952 ) | 3.805 ( 2.432 to 5.521 ) | -0.31 (-0.33 to -0.28) | 68.554 |
| South Asia | Rheumatic heart disease | Male | 21904 ( 14012 to 31903 ) | 32013 ( 20102 to 46670 ) | 4.059 ( 2.599 to 5.802 ) | 3.47 ( 2.208 to 4.973 ) | -0.58 (-0.61 to -0.56) | 46.151 |
| Southeast Asia | Hypertensive heart disease | Female | 11271 ( 7263 to 16438 ) | 30056 ( 19135 to 43184 ) | 9.406 ( 5.998 to 13.59 ) | 9.292 ( 5.906 to 13.405 ) | -0.12 (-0.16 to -0.08) | 166.667 |
| Southeast Asia | Hypertensive heart disease | Male | 9387 ( 6148 to 13887 ) | 23629 ( 14783 to 34665 ) | 9.106 ( 5.807 to 13.376 ) | 8.756 ( 5.476 to 12.732 ) | -0.24 (-0.29 to -0.19) | 151.72 |
| Southeast Asia | Ischemic heart disease | Female | 9429 ( 6079 to 14054 ) | 29037 ( 18270 to 43227 ) | 7.866 ( 4.909 to 11.648 ) | 9.234 ( 5.913 to 13.616 ) | 0.64 (0.6 to 0.68) | 207.954 |
| Southeast Asia | Ischemic heart disease | Male | 10573 ( 6823 to 15651 ) | 32064 ( 20943 to 47433 ) | 10.283 ( 6.53 to 15.486 ) | 12.363 ( 7.967 to 18.267 ) | 0.72 (0.69 to 0.76) | 203.263 |
| Southeast Asia | Rheumatic heart disease | Female | 2742 ( 1755 to 3990 ) | 2639 ( 1694 to 3836 ) | 1.094 ( 0.712 to 1.601 ) | 0.789 ( 0.51 to 1.151 ) | -1.28 (-1.35 to -1.21) | -3.756 |
| Southeast Asia | Rheumatic heart disease | Male | 2213 ( 1440 to 3311 ) | 1956 ( 1207 to 2886 ) | 0.853 ( 0.558 to 1.259 ) | 0.593 ( 0.367 to 0.873 ) | -1.41 (-1.47 to -1.34) | -11.613 |
| Southern Latin America | Hypertensive heart disease | Female | 1209 ( 709 to 1867 ) | 3051 ( 1781 to 4657 ) | 4.734 ( 2.784 to 7.244 ) | 5.746 ( 3.4 to 8.714 ) | 0.91 (0.85 to 0.97) | 152.357 |
| Southern Latin America | Hypertensive heart disease | Male | 1006 ( 603 to 1551 ) | 2527 ( 1419 to 3924 ) | 5.312 ( 3.183 to 8.089 ) | 6.835 ( 3.893 to 10.549 ) | 1.05 (1 to 1.11) | 151.193 |
| Southern Latin America | Ischemic heart disease | Female | 1670 ( 998 to 2569 ) | 2607 ( 1559 to 3999 ) | 6.684 ( 4.03 to 10.25 ) | 5.027 ( 3.042 to 7.71 ) | -1.17 (-1.28 to -1.07) | 56.108 |
| Southern Latin America | Ischemic heart disease | Male | 2064 ( 1246 to 3131 ) | 3525 ( 2079 to 5411 ) | 10.986 ( 6.78 to 16.65 ) | 9.422 ( 5.655 to 14.444 ) | -0.66 (-0.75 to -0.57) | 70.785 |
| Southern Latin America | Rheumatic heart disease | Female | 250 ( 155 to 379 ) | 167 ( 101 to 254 ) | 0.975 ( 0.612 to 1.473 ) | 0.381 ( 0.233 to 0.57 ) | -3.62 (-3.93 to -3.31) | -33.2 |
| Southern Latin America | Rheumatic heart disease | Male | 176 ( 111 to 263 ) | 94 ( 58 to 144 ) | 0.793 ( 0.498 to 1.213 ) | 0.271 ( 0.168 to 0.414 ) | -4.02 (-4.4 to -3.64) | -46.591 |
| Southern Sub-Saharan Africa | Hypertensive heart disease | Female | 1409 ( 866 to 2093 ) | 3359 ( 2095 to 4973 ) | 9.904 ( 6.078 to 14.684 ) | 10.951 ( 6.81 to 16.106 ) | 0.41 (0.34 to 0.48) | 138.396 |
| Southern Sub-Saharan Africa | Hypertensive heart disease | Male | 1281 ( 799 to 1921 ) | 2717 ( 1678 to 4086 ) | 12.562 ( 7.62 to 18.882 ) | 13.129 ( 8.058 to 20.044 ) | 0.07 (0.02 to 0.12) | 112.1 |
| Southern Sub-Saharan Africa | Ischemic heart disease | Female | 1119 ( 718 to 1688 ) | 2399 ( 1543 to 3660 ) | 7.964 ( 5.045 to 11.979 ) | 7.969 ( 5.117 to 12.167 ) | -0.07 (-0.15 to 0) | 114.388 |
| Southern Sub-Saharan Africa | Ischemic heart disease | Male | 1269 ( 807 to 1903 ) | 2691 ( 1704 to 3998 ) | 13.101 ( 8.5 to 19.442 ) | 13.834 ( 8.979 to 20.422 ) | 0.28 (0.18 to 0.38) | 112.057 |
| Southern Sub-Saharan Africa | Rheumatic heart disease | Female | 551 ( 320 to 862 ) | 594 ( 346 to 932 ) | 1.716 ( 1.015 to 2.623 ) | 1.424 ( 0.838 to 2.217 ) | -0.65 (-0.71 to -0.58) | 7.804 |
| Southern Sub-Saharan Africa | Rheumatic heart disease | Male | 503 ( 296 to 801 ) | 520 ( 299 to 850 ) | 1.605 ( 0.964 to 2.517 ) | 1.271 ( 0.738 to 2.07 ) | -0.72 (-0.77 to -0.68) | 3.38 |
| Tropical Latin America | Hypertensive heart disease | Female | 3831 ( 2392 to 5530 ) | 12956 ( 7889 to 19296 ) | 8.477 ( 5.309 to 12.219 ) | 9.146 ( 5.58 to 13.611 ) | 0.25 (0.23 to 0.28) | 238.188 |
| Tropical Latin America | Hypertensive heart disease | Male | 3041 ( 1911 to 4458 ) | 10017 ( 6081 to 15090 ) | 8.206 ( 5.104 to 11.868 ) | 9.475 ( 5.728 to 14.14 ) | 0.41 (0.37 to 0.46) | 229.398 |
| Tropical Latin America | Ischemic heart disease | Female | 3719 ( 2359 to 5472 ) | 11534 ( 7297 to 17073 ) | 8.453 ( 5.327 to 12.389 ) | 8.148 ( 5.13 to 12.082 ) | -0.02 (-0.07 to 0.03) | 210.137 |
| Tropical Latin America | Ischemic heart disease | Male | 4469 ( 2851 to 6480 ) | 12666 ( 8187 to 18931 ) | 11.859 ( 7.588 to 17.203 ) | 11.639 ( 7.513 to 17.414 ) | 0.05 (0 to 0.11) | 183.419 |
| Tropical Latin America | Rheumatic heart disease | Female | 1033 ( 642 to 1530 ) | 925 ( 571 to 1361 ) | 1.265 ( 0.795 to 1.856 ) | 0.782 ( 0.482 to 1.152 ) | -1.72 (-1.77 to -1.66) | -10.455 |
| Tropical Latin America | Rheumatic heart disease | Male | 850 ( 511 to 1304 ) | 539 ( 334 to 801 ) | 1.014 ( 0.621 to 1.533 ) | 0.521 ( 0.324 to 0.769 ) | -2.51 (-2.63 to -2.39) | -36.588 |
| Western Europe | Hypertensive heart disease | Female | 15410 ( 9071 to 22989 ) | 38727 ( 22691 to 57370 ) | 4.059 ( 2.445 to 5.981 ) | 5.952 ( 3.59 to 8.637 ) | 2.06 (1.71 to 2.4) | 151.311 |
| Western Europe | Hypertensive heart disease | Male | 6869 ( 4079 to 10366 ) | 22901 ( 13418 to 33968 ) | 3.04 ( 1.81 to 4.538 ) | 5.223 ( 3.078 to 7.709 ) | 2.09 (1.85 to 2.34) | 233.396 |
| Western Europe | Ischemic heart disease | Female | 43212 ( 26124 to 64753 ) | 59762 ( 38194 to 88653 ) | 11.519 ( 7.067 to 17.158 ) | 9.548 ( 6.097 to 14.293 ) | -0.79 (-0.97 to -0.62) | 38.3 |
| Western Europe | Ischemic heart disease | Male | 41304 ( 25050 to 62478 ) | 71865 ( 44637 to 105773 ) | 18.077 ( 11.1 to 27.305 ) | 16.693 ( 10.572 to 24.685 ) | -0.45 (-0.61 to -0.3) | 73.99 |
| Western Europe | Rheumatic heart disease | Female | 3158 ( 1882 to 4767 ) | 4647 ( 2856 to 6922 ) | 1.004 ( 0.621 to 1.483 ) | 0.951 ( 0.59 to 1.399 ) | 0.07 (-0.04 to 0.17) | 47.15 |
| Western Europe | Rheumatic heart disease | Male | 1135 ( 689 to 1718 ) | 2326 ( 1432 to 3474 ) | 0.529 ( 0.326 to 0.784 ) | 0.616 ( 0.392 to 0.896 ) | 0.4 (0.34 to 0.47) | 104.934 |
| Western Sub-Saharan Africa | Hypertensive heart disease | Female | 4844 ( 3097 to 6979 ) | 11445 ( 7354 to 16879 ) | 12.411 ( 8.028 to 17.728 ) | 12.793 ( 8.142 to 18.603 ) | 0.14 (0.08 to 0.2) | 136.272 |
| Western Sub-Saharan Africa | Hypertensive heart disease | Male | 6082 ( 3856 to 8854 ) | 13825 ( 9048 to 20159 ) | 16.257 ( 10.418 to 23.587 ) | 17.1 ( 11.184 to 24.992 ) | 0.19 (0.16 to 0.21) | 127.31 |
| Western Sub-Saharan Africa | Ischemic heart disease | Female | 1678 ( 1053 to 2609 ) | 5065 ( 3228 to 7673 ) | 4.34 ( 2.789 to 6.729 ) | 5.751 ( 3.636 to 8.755 ) | 0.98 (0.95 to 1.01) | 201.847 |
| Western Sub-Saharan Africa | Ischemic heart disease | Male | 2338 ( 1444 to 3578 ) | 6004 ( 3774 to 9140 ) | 6.484 ( 4.113 to 9.948 ) | 7.632 ( 4.854 to 11.849 ) | 0.56 (0.53 to 0.58) | 156.801 |
| Western Sub-Saharan Africa | Rheumatic heart disease | Female | 1152 ( 683 to 1750 ) | 1779 ( 1064 to 2784 ) | 1.033 ( 0.635 to 1.583 ) | 0.669 ( 0.401 to 1.037 ) | -1.56 (-1.63 to -1.48) | 54.427 |
| Western Sub-Saharan Africa | Rheumatic heart disease | Male | 1288 ( 764 to 2010 ) | 2290 ( 1354 to 3563 ) | 1.187 ( 0.719 to 1.805 ) | 0.865 ( 0.518 to 1.323 ) | -1.23 (-1.32 to -1.15) | 77.795 |

**Table S3 The prevalence of SHF due to HHD in 1990 and 2021 across countries and territories.**

| Locations | cause | sex | 1990 Prevalence cases(95% UI) | 2021 Prevalence cases(95% UI) | 1990 ASPR(95% UI) | 2021 ASPR(95% UI) | 1990-2021 EAPC（95%CI） | 1990-2021 Prevalence cases changes |
| --- | --- | --- | --- | --- | --- | --- | --- | --- |
| Afghanistan | Hypertensive heart disease | Both | 4959 ( 3733 to 6253 ) | 7741 ( 6024 to 9786 ) | 84.954 ( 63.763 to 109.065 ) | 82.26 ( 61.408 to 107.178 ) | -0.06 (-0.08 to -0.04) | 56.1 |
| Albania | Hypertensive heart disease | Both | 499 ( 363 to 667 ) | 1211 ( 838 to 1669 ) | 26.722 ( 18.974 to 36.346 ) | 27.551 ( 19.302 to 37.51 ) | -0.05 (-0.08 to -0.02) | 142.685 |
| Algeria | Hypertensive heart disease | Both | 10268 ( 7928 to 12872 ) | 31711 ( 24366 to 40805 ) | 88.681 ( 68.234 to 113.408 ) | 94.018 ( 71.228 to 120.91 ) | 0.42 (0.34 to 0.49) | 208.833 |
| American Samoa | Hypertensive heart disease | Both | 7 ( 5 to 9 ) | 13 ( 9 to 17 ) | 35.801 ( 27.297 to 47.827 ) | 29.846 ( 21.98 to 40.384 ) | -0.82 (-0.98 to -0.66) | 85.714 |
| Andorra | Hypertensive heart disease | Both | 14 ( 10 to 19 ) | 45 ( 34 to 61 ) | 26.989 ( 20.149 to 36.604 ) | 27.775 ( 20.629 to 37.005 ) | 0.36 (0.29 to 0.43) | 221.429 |
| Angola | Hypertensive heart disease | Both | 2135 ( 1556 to 2842 ) | 7407 ( 5378 to 9911 ) | 70.964 ( 50.668 to 95.548 ) | 78.187 ( 55.865 to 104.836 ) | 0.35 (0.33 to 0.37) | 246.932 |
| Antigua and Barbuda | Hypertensive heart disease | Both | 52 ( 40 to 66 ) | 100 ( 75 to 128 ) | 89.736 ( 70.354 to 114.244 ) | 100.729 ( 76.621 to 129.713 ) | 0.55 (0.44 to 0.66) | 92.308 |
| Argentina | Hypertensive heart disease | Both | 8792 ( 6431 to 11904 ) | 18677 ( 12777 to 25908 ) | 28.43 ( 20.965 to 38.322 ) | 32.338 ( 22.467 to 44.376 ) | 0.66 (0.59 to 0.72) | 112.432 |
| Armenia | Hypertensive heart disease | Both | 733 ( 534 to 986 ) | 1806 ( 1350 to 2332 ) | 31.177 ( 22.211 to 42.326 ) | 41.611 ( 31.112 to 53.689 ) | 1.2 (1.09 to 1.32) | 146.385 |
| Australia | Hypertensive heart disease | Both | 1946 ( 1456 to 2528 ) | 8860 ( 6840 to 11140 ) | 10.074 ( 7.529 to 13.008 ) | 18.29 ( 14.297 to 22.549 ) | 2.21 (2.1 to 2.33) | 355.293 |
| Austria | Hypertensive heart disease | Both | 3497 ( 2606 to 4456 ) | 7090 ( 5120 to 9360 ) | 28.099 ( 21.276 to 35.469 ) | 33.806 ( 24.928 to 43.602 ) | 0.96 (0.74 to 1.18) | 102.745 |
| Azerbaijan | Hypertensive heart disease | Both | 1426 ( 1034 to 1952 ) | 2764 ( 1851 to 3773 ) | 32.323 ( 22.858 to 44.802 ) | 31.997 ( 20.661 to 44.416 ) | 0.01 (-0.03 to 0.06) | 93.829 |
| Bahamas | Hypertensive heart disease | Both | 148 ( 116 to 188 ) | 393 ( 304 to 512 ) | 100.745 ( 76.807 to 130.189 ) | 107.437 ( 82.601 to 140.972 ) | 0.57 (0.48 to 0.66) | 165.541 |
| Bahrain | Hypertensive heart disease | Both | 77 ( 61 to 97 ) | 500 ( 384 to 657 ) | 45.585 ( 34.767 to 59.974 ) | 58.863 ( 43.676 to 76.937 ) | 1.16 (1.05 to 1.28) | 549.351 |
| Bangladesh | Hypertensive heart disease | Both | 17305 ( 13468 to 22237 ) | 55773 ( 42858 to 71659 ) | 42.97 ( 32.862 to 55.871 ) | 45.426 ( 34.668 to 57.697 ) | 0.14 (0.08 to 0.2) | 222.294 |
| Barbados | Hypertensive heart disease | Both | 185 ( 138 to 251 ) | 364 ( 278 to 478 ) | 58.166 ( 44.511 to 77.726 ) | 70.208 ( 53.924 to 91.371 ) | 0.82 (0.75 to 0.89) | 96.757 |
| Belarus | Hypertensive heart disease | Both | 930 ( 656 to 1241 ) | 454 ( 302 to 653 ) | 7.292 ( 5.15 to 9.732 ) | 2.831 ( 1.906 to 4.031 ) | -3.77 (-4.16 to -3.37) | -51.183 |
| Belgium | Hypertensive heart disease | Both | 997 ( 696 to 1403 ) | 1948 ( 1418 to 2681 ) | 6.336 ( 4.503 to 8.824 ) | 7.496 ( 5.591 to 9.909 ) | 1.54 (1.2 to 1.87) | 95.386 |
| Belize | Hypertensive heart disease | Both | 57 ( 44 to 73 ) | 228 ( 173 to 291 ) | 60.049 ( 46.197 to 77.449 ) | 83.009 ( 62.407 to 108.067 ) | 1.45 (1.34 to 1.57) | 300 |
| Benin | Hypertensive heart disease | Both | 1612 ( 1180 to 2113 ) | 4147 ( 3110 to 5356 ) | 87.534 ( 63.679 to 116.491 ) | 90.098 ( 66.271 to 118.307 ) | 0.06 (0.04 to 0.08) | 157.258 |
| Bermuda | Hypertensive heart disease | Both | 16 ( 12 to 22 ) | 74 ( 54 to 99 ) | 26.822 ( 20.131 to 35.798 ) | 52.472 ( 39.077 to 69.03 ) | 2.36 (2.25 to 2.47) | 362.5 |
| Bhutan | Hypertensive heart disease | Both | 80 ( 63 to 101 ) | 246 ( 189 to 312 ) | 42.885 ( 33.017 to 55.069 ) | 43.278 ( 33.178 to 55.325 ) | 0.02 (0 to 0.03) | 207.5 |
| Bolivia (Plurinational State of) | Hypertensive heart disease | Both | 1248 ( 959 to 1610 ) | 4099 ( 3055 to 5389 ) | 42.989 ( 32.787 to 56.974 ) | 48.813 ( 36.286 to 64.548 ) | 0.41 (0.35 to 0.47) | 228.446 |
| Bosnia and Herzegovina | Hypertensive heart disease | Both | 869 ( 617 to 1166 ) | 1818 ( 1203 to 2567 ) | 24.617 ( 17.105 to 33.623 ) | 28.634 ( 19.223 to 39.824 ) | 0.93 (0.79 to 1.07) | 109.206 |
| Botswana | Hypertensive heart disease | Both | 304 ( 208 to 418 ) | 857 ( 609 to 1154 ) | 66.139 ( 46.596 to 91.656 ) | 68.324 ( 48.164 to 93.729 ) | 0.1 (0.04 to 0.15) | 181.908 |
| Brazil | Hypertensive heart disease | Both | 38796 ( 30063 to 49273 ) | 130477 ( 97774 to 173150 ) | 48.643 ( 37.603 to 61.964 ) | 53.861 ( 40.439 to 71.681 ) | 0.32 (0.29 to 0.36) | 236.316 |
| Brunei Darussalam | Hypertensive heart disease | Both | 21 ( 15 to 27 ) | 57 ( 40 to 80 ) | 18.896 ( 11.603 to 27.252 ) | 18.559 ( 11.728 to 26.534 ) | -0.2 (-0.27 to -0.13) | 171.429 |
| Bulgaria | Hypertensive heart disease | Both | 5355 ( 3773 to 7393 ) | 11505 ( 7954 to 15799 ) | 45.624 ( 33.351 to 61.423 ) | 77.855 ( 55.865 to 103.309 ) | 2.54 (2.24 to 2.85) | 114.846 |
| Burkina Faso | Hypertensive heart disease | Both | 3168 ( 2303 to 4168 ) | 7490 ( 5560 to 9774 ) | 86.914 ( 62.813 to 116.509 ) | 91.798 ( 67.778 to 124.984 ) | 0.19 (0.17 to 0.21) | 136.427 |
| Burundi | Hypertensive heart disease | Both | 1843 ( 1317 to 2469 ) | 3878 ( 2819 to 5053 ) | 90.574 ( 65.934 to 122.595 ) | 94.084 ( 67.572 to 125.529 ) | 0.15 (0.13 to 0.17) | 110.418 |
| Cabo Verde | Hypertensive heart disease | Both | 228 ( 166 to 305 ) | 421 ( 314 to 551 ) | 96.734 ( 70.669 to 128.104 ) | 99.709 ( 73.279 to 132.378 ) | 0.09 (0.07 to 0.11) | 84.649 |
| Cambodia | Hypertensive heart disease | Both | 2357 ( 1794 to 3060 ) | 6825 ( 5232 to 8941 ) | 63.1 ( 46.766 to 82.633 ) | 64.061 ( 48.586 to 83.61 ) | 0.05 (0.01 to 0.08) | 189.563 |
| Cameroon | Hypertensive heart disease | Both | 3412 ( 2569 to 4445 ) | 9765 ( 7416 to 12812 ) | 91.795 ( 68.281 to 122.213 ) | 90.453 ( 66.253 to 121.303 ) | 0.03 (-0.05 to 0.11) | 186.196 |
| Canada | Hypertensive heart disease | Both | 4068 ( 3203 to 5175 ) | 20777 ( 15762 to 26549 ) | 12.588 ( 9.965 to 15.975 ) | 30.9 ( 24.389 to 39.407 ) | 3.52 (3.26 to 3.78) | 410.742 |
| Central African Republic | Hypertensive heart disease | Both | 550 ( 390 to 743 ) | 1120 ( 806 to 1500 ) | 64.694 ( 45.969 to 89.856 ) | 67.636 ( 48.083 to 92.692 ) | 0.15 (0.13 to 0.17) | 103.636 |
| Chad | Hypertensive heart disease | Both | 2106 ( 1539 to 2815 ) | 4337 ( 3207 to 5660 ) | 81.843 ( 59.861 to 109.658 ) | 87.108 ( 63.043 to 115.019 ) | 0.21 (0.18 to 0.23) | 105.935 |
| Chile | Hypertensive heart disease | Both | 3156 ( 2303 to 4122 ) | 11775 ( 8420 to 15622 ) | 33.328 ( 24.108 to 43.452 ) | 45.448 ( 32.588 to 60.38 ) | 1.28 (1.19 to 1.36) | 273.099 |
| China | Hypertensive heart disease | Both | 489593 ( 365303 to 635452 ) | 1274212 ( 917987 to 1694570 ) | 71.093 ( 53.383 to 91.217 ) | 62.691 ( 46.342 to 82.757 ) | -0.68 (-0.9 to -0.45) | 160.259 |
| Colombia | Hypertensive heart disease | Both | 9698 ( 7479 to 12344 ) | 23885 ( 17830 to 31574 ) | 61.181 ( 46.267 to 79.685 ) | 43.021 ( 31.944 to 57.132 ) | -1.57 (-1.7 to -1.45) | 146.288 |
| Comoros | Hypertensive heart disease | Both | 161 ( 116 to 212 ) | 434 ( 321 to 562 ) | 96.919 ( 70.169 to 129.311 ) | 99.046 ( 72.414 to 129.406 ) | 0.05 (0.04 to 0.07) | 169.565 |
| Congo | Hypertensive heart disease | Both | 602 ( 422 to 800 ) | 1672 ( 1216 to 2221 ) | 70.452 ( 49.735 to 94.889 ) | 76.483 ( 53.649 to 103.146 ) | 0.29 (0.26 to 0.31) | 177.741 |
| Cook Islands | Hypertensive heart disease | Both | 12 ( 9 to 16 ) | 26 ( 20 to 34 ) | 108.442 ( 83.008 to 141.429 ) | 103.403 ( 80.073 to 134.19 ) | 0 (-0.05 to 0.05) | 116.667 |
| Costa Rica | Hypertensive heart disease | Both | 769 ( 589 to 989 ) | 2787 ( 2076 to 3659 ) | 45.386 ( 34.339 to 59.202 ) | 50.961 ( 37.685 to 67.347 ) | 0.19 (0.02 to 0.36) | 262.419 |
| Coted'Ivoire | Hypertensive heart disease | Both | 2679 ( 1984 to 3485 ) | 8209 ( 6164 to 10625 ) | 84.755 ( 62.332 to 113.111 ) | 87.317 ( 64.569 to 115.88 ) | 0.11 (0.07 to 0.14) | 206.42 |
| Croatia | Hypertensive heart disease | Both | 1662 ( 1134 to 2283 ) | 2198 ( 1468 to 3010 ) | 29.928 ( 20.225 to 41.488 ) | 23.771 ( 16.502 to 32.034 ) | -0.96 (-1.18 to -0.73) | 32.25 |
| Cuba | Hypertensive heart disease | Both | 2325 ( 1763 to 3040 ) | 11273 ( 8387 to 14762 ) | 22.32 ( 17.033 to 29.083 ) | 56.789 ( 42.541 to 74.242 ) | 3.43 (3.22 to 3.65) | 384.86 |
| Cyprus | Hypertensive heart disease | Both | 132 ( 86 to 186 ) | 450 ( 317 to 639 ) | 18.531 ( 12.674 to 25.803 ) | 21.962 ( 15.955 to 30.484 ) | 0.44 (0.25 to 0.63) | 240.909 |
| Czechia | Hypertensive heart disease | Both | 1409 ( 976 to 1902 ) | 7687 ( 5161 to 11033 ) | 10.41 ( 7.38 to 14.03 ) | 34.1 ( 23.303 to 47.666 ) | 4.2 (3.59 to 4.82) | 445.564 |
| Democratic People's Republic of Korea | Hypertensive heart disease | Both | 6672 ( 5052 to 8992 ) | 17905 ( 13392 to 23366 ) | 52.19 ( 39.467 to 70.375 ) | 59.081 ( 44.668 to 76.623 ) | 0.47 (0.42 to 0.51) | 168.36 |
| Democratic Republic of the Congo | Hypertensive heart disease | Both | 8831 ( 6183 to 11740 ) | 23357 ( 16952 to 31251 ) | 72.819 ( 50.686 to 98.616 ) | 78.245 ( 55.785 to 106.206 ) | 0.27 (0.19 to 0.34) | 164.489 |
| Denmark | Hypertensive heart disease | Both | 374 ( 259 to 521 ) | 1133 ( 793 to 1635 ) | 4.406 ( 3.13 to 6.045 ) | 8.949 ( 6.353 to 12.378 ) | 3.2 (2.76 to 3.65) | 202.941 |
| Djibouti | Hypertensive heart disease | Both | 107 ( 80 to 139 ) | 520 ( 380 to 688 ) | 97.407 ( 71.293 to 129.538 ) | 97.929 ( 71.699 to 130.484 ) | -0.02 (-0.04 to 0) | 385.981 |
| Dominica | Hypertensive heart disease | Both | 50 ( 37 to 65 ) | 67 ( 51 to 87 ) | 81.915 ( 62.394 to 106.373 ) | 84.775 ( 64.445 to 110.229 ) | 0.26 (0.22 to 0.29) | 34 |
| Dominican Republic | Hypertensive heart disease | Both | 2174 ( 1688 to 2768 ) | 6014 ( 4488 to 8049 ) | 62.464 ( 47.552 to 79.994 ) | 61.401 ( 45.522 to 82.919 ) | -0.22 (-0.27 to -0.17) | 176.633 |
| Ecuador | Hypertensive heart disease | Both | 3583 ( 2777 to 4523 ) | 10095 ( 7601 to 13254 ) | 69.439 ( 52.915 to 88.732 ) | 62.869 ( 47.635 to 83.089 ) | 0.15 (-0.04 to 0.34) | 181.747 |
| Egypt | Hypertensive heart disease | Both | 18864 ( 14987 to 23546 ) | 41028 ( 31360 to 53146 ) | 78.631 ( 60.568 to 100.191 ) | 73.834 ( 55.485 to 95.967 ) | -0.27 (-0.34 to -0.2) | 117.494 |
| El Salvador | Hypertensive heart disease | Both | 792 ( 597 to 1039 ) | 1637 ( 1195 to 2175 ) | 27.699 ( 20.75 to 36.785 ) | 25.095 ( 18.164 to 33.541 ) | -0.38 (-0.42 to -0.33) | 106.692 |
| Equatorial Guinea | Hypertensive heart disease | Both | 107 ( 75 to 144 ) | 344 ( 249 to 461 ) | 69.766 ( 48.15 to 94.908 ) | 79.333 ( 56.032 to 107.238 ) | 0.46 (0.42 to 0.5) | 221.495 |
| Eritrea | Hypertensive heart disease | Both | 733 ( 532 to 959 ) | 2114 ( 1543 to 2746 ) | 89.299 ( 65.271 to 121.542 ) | 93.83 ( 68.449 to 125.542 ) | 0.11 (0.09 to 0.12) | 188.404 |
| Estonia | Hypertensive heart disease | Both | 427 ( 311 to 577 ) | 2005 ( 1281 to 2867 ) | 21.294 ( 15.65 to 28.41 ) | 68.603 ( 45.192 to 96.784 ) | 5.78 (5.19 to 6.38) | 369.555 |
| Eswatini | Hypertensive heart disease | Both | 160 ( 112 to 214 ) | 317 ( 226 to 421 ) | 66.935 ( 47.177 to 90.535 ) | 68.53 ( 48.522 to 92.967 ) | 0.05 (0 to 0.09) | 98.125 |
| Ethiopia | Hypertensive heart disease | Both | 13771 ( 10155 to 17769 ) | 38118 ( 28996 to 48610 ) | 88.198 ( 67.013 to 113.767 ) | 98.343 ( 75.005 to 127.479 ) | 0.34 (0.33 to 0.35) | 176.799 |
| Fiji | Hypertensive heart disease | Both | 116 ( 90 to 146 ) | 246 ( 185 to 321 ) | 39.496 ( 30.18 to 50.753 ) | 38.243 ( 29.025 to 50.071 ) | -0.23 (-0.28 to -0.18) | 112.069 |
| Finland | Hypertensive heart disease | Both | 851 ( 569 to 1165 ) | 5028 ( 3287 to 7399 ) | 11.92 ( 8.182 to 16.035 ) | 35.047 ( 23.753 to 50.387 ) | 4.61 (4.32 to 4.9) | 490.834 |
| France | Hypertensive heart disease | Both | 34542 ( 25330 to 46141 ) | 80470 ( 58623 to 110145 ) | 38.743 ( 28.921 to 51.687 ) | 47.277 ( 35.384 to 64.409 ) | 0.55 (0.36 to 0.73) | 132.963 |
| Gabon | Hypertensive heart disease | Both | 400 ( 285 to 542 ) | 734 ( 525 to 974 ) | 78.949 ( 56.804 to 107.587 ) | 83.007 ( 59.185 to 112.233 ) | 0.17 (0.16 to 0.18) | 83.5 |
| Gambia | Hypertensive heart disease | Both | 253 ( 185 to 329 ) | 757 ( 556 to 994 ) | 83.609 ( 60.05 to 110.159 ) | 85.803 ( 62.462 to 114.373 ) | 0.1 (0.08 to 0.12) | 199.209 |
| Georgia | Hypertensive heart disease | Both | 1510 ( 1063 to 2083 ) | 3028 ( 1556 to 4613 ) | 25.492 ( 17.998 to 34.959 ) | 49.538 ( 26.596 to 74.824 ) | 3.79 (3.13 to 4.45) | 100.53 |
| Germany | Hypertensive heart disease | Both | 33661 ( 24126 to 47064 ) | 82328 ( 56375 to 116393 ) | 25.806 ( 18.768 to 35.626 ) | 36.31 ( 25.881 to 50.06 ) | 2.63 (2.1 to 3.16) | 144.58 |
| Ghana | Hypertensive heart disease | Both | 4575 ( 3484 to 5939 ) | 12741 ( 9165 to 16641 ) | 89.219 ( 66.363 to 118.556 ) | 90.534 ( 65.09 to 118.996 ) | -0.03 (-0.05 to -0.01) | 178.492 |
| Greece | Hypertensive heart disease | Both | 1931 ( 1382 to 2573 ) | 3777 ( 2588 to 5257 ) | 13.097 ( 9.525 to 17.512 ) | 13.392 ( 9.471 to 17.989 ) | 0.07 (-0.04 to 0.18) | 95.598 |
| Greenland | Hypertensive heart disease | Both | 8 ( 6 to 10 ) | 17 ( 13 to 22 ) | 20.727 ( 15.109 to 26.615 ) | 27.452 ( 20.433 to 35.694 ) | 1.13 (0.96 to 1.3) | 112.5 |
| Grenada | Hypertensive heart disease | Both | 45 ( 33 to 59 ) | 73 ( 55 to 95 ) | 57.054 ( 43.122 to 75.385 ) | 71.553 ( 54.68 to 93.716 ) | 0.96 (0.9 to 1.01) | 62.222 |
| Guam | Hypertensive heart disease | Both | 45 ( 35 to 58 ) | 82 ( 59 to 109 ) | 69.089 ( 52.049 to 91.388 ) | 38.969 ( 28.414 to 52.355 ) | -2.16 (-2.33 to -2) | 82.222 |
| Guatemala | Hypertensive heart disease | Both | 650 ( 493 to 866 ) | 2146 ( 1592 to 2997 ) | 22.118 ( 16.662 to 29.722 ) | 20.834 ( 15.433 to 29.205 ) | -0.09 (-0.16 to -0.03) | 230.154 |
| Guinea | Hypertensive heart disease | Both | 2607 ( 1923 to 3477 ) | 4487 ( 3313 to 5810 ) | 87.721 ( 64.451 to 116.721 ) | 88.988 ( 65.386 to 118.303 ) | 0 (-0.02 to 0.03) | 72.114 |
| Guinea-Bissau | Hypertensive heart disease | Both | 256 ( 184 to 341 ) | 479 ( 360 to 629 ) | 79.668 ( 58.375 to 107.82 ) | 81.601 ( 59.444 to 109.619 ) | 0.06 (0.05 to 0.07) | 87.109 |
| Guyana | Hypertensive heart disease | Both | 281 ( 214 to 362 ) | 486 ( 368 to 630 ) | 80.688 ( 60.686 to 105.444 ) | 86.219 ( 65.284 to 112.386 ) | 0.19 (0.09 to 0.29) | 72.954 |
| Haiti | Hypertensive heart disease | Both | 1383 ( 1058 to 1801 ) | 3184 ( 2453 to 4075 ) | 52.013 ( 39.733 to 67.909 ) | 52.299 ( 39.647 to 67.902 ) | 0 (-0.03 to 0.02) | 130.224 |
| Honduras | Hypertensive heart disease | Both | 1216 ( 947 to 1547 ) | 3710 ( 2759 to 4872 ) | 63.63 ( 48.998 to 82.259 ) | 63.527 ( 47.36 to 84.573 ) | -0.13 (-0.16 to -0.09) | 205.099 |
| Hungary | Hypertensive heart disease | Both | 4531 ( 2965 to 6413 ) | 9666 ( 6443 to 13654 ) | 31.254 ( 20.839 to 43.256 ) | 46.364 ( 31.958 to 64.482 ) | 1.66 (1.51 to 1.81) | 113.33 |
| Iceland | Hypertensive heart disease | Both | 12 ( 7 to 17 ) | 48 ( 33 to 69 ) | 4 ( 2.542 to 5.656 ) | 7.896 ( 5.513 to 11.076 ) | 2.49 (2.17 to 2.81) | 300 |
| India | Hypertensive heart disease | Both | 114575 ( 87714 to 146118 ) | 361952 ( 264856 to 480297 ) | 31.878 ( 24.118 to 40.957 ) | 34.582 ( 25.567 to 45.93 ) | 0.26 (0.23 to 0.28) | 215.908 |
| Indonesia | Hypertensive heart disease | Both | 50135 ( 39553 to 62563 ) | 123948 ( 93485 to 160862 ) | 61.726 ( 48.265 to 78.037 ) | 62.017 ( 47.003 to 80.837 ) | -0.09 (-0.13 to -0.06) | 147.228 |
| Iran (Islamic Republic of) | Hypertensive heart disease | Both | 17054 ( 13547 to 21437 ) | 60406 ( 46457 to 77135 ) | 73.661 ( 58.684 to 93.131 ) | 81.508 ( 62.111 to 105.528 ) | 0.49 (0.43 to 0.56) | 254.204 |
| Iraq | Hypertensive heart disease | Both | 5288 ( 4086 to 6695 ) | 13902 ( 10651 to 17691 ) | 67.297 ( 51.859 to 85.862 ) | 63.476 ( 48.403 to 82.867 ) | -0.22 (-0.24 to -0.2) | 162.897 |
| Ireland | Hypertensive heart disease | Both | 190 ( 130 to 262 ) | 790 ( 546 to 1101 ) | 4.779 ( 3.323 to 6.478 ) | 9.619 ( 6.768 to 13.215 ) | 3.15 (2.85 to 3.44) | 315.789 |
| Israel | Hypertensive heart disease | Both | 379 ( 266 to 534 ) | 1537 ( 1095 to 2119 ) | 7.996 ( 5.703 to 11.064 ) | 11.68 ( 8.449 to 16.064 ) | 2 (1.77 to 2.23) | 305.541 |
| Italy | Hypertensive heart disease | Both | 29772 ( 19448 to 41225 ) | 95546 ( 69463 to 126306 ) | 32.863 ( 22.124 to 44.736 ) | 55.355 ( 41.383 to 72.728 ) | 2.24 (1.6 to 2.88) | 220.926 |
| Jamaica | Hypertensive heart disease | Both | 2022 ( 1568 to 2649 ) | 3270 ( 2490 to 4239 ) | 107.652 ( 83.71 to 140.12 ) | 103.273 ( 77.933 to 134.771 ) | 0.13 (0.04 to 0.23) | 61.721 |
| Japan | Hypertensive heart disease | Both | 24448 ( 17039 to 33136 ) | 63740 ( 45094 to 88779 ) | 15.87 ( 11.114 to 21.716 ) | 14.631 ( 10.849 to 19.677 ) | -0.66 (-0.92 to -0.41) | 160.717 |
| Jordan | Hypertensive heart disease | Both | 1265 ( 1012 to 1583 ) | 7802 ( 5980 to 9931 ) | 99.585 ( 76.998 to 128.964 ) | 111.388 ( 84.094 to 142.461 ) | 0.66 (0.58 to 0.74) | 516.759 |
| Kazakhstan | Hypertensive heart disease | Both | 2572 ( 1833 to 3458 ) | 3060 ( 2004 to 4348 ) | 21.571 ( 15.212 to 29.432 ) | 19.1 ( 12.405 to 27.38 ) | -0.47 (-0.99 to 0.05) | 18.974 |
| Kenya | Hypertensive heart disease | Both | 6716 ( 5154 to 8646 ) | 19071 ( 14466 to 24253 ) | 94.557 ( 72.328 to 122.437 ) | 97.383 ( 73.991 to 124.032 ) | -0.37 (-0.55 to -0.19) | 183.964 |
| Kiribati | Hypertensive heart disease | Both | 8 ( 6 to 10 ) | 14 ( 11 to 18 ) | 25.168 ( 18.478 to 33.027 ) | 24.302 ( 17.935 to 32.052 ) | -0.15 (-0.18 to -0.12) | 75 |
| Kuwait | Hypertensive heart disease | Both | 620 ( 502 to 767 ) | 2680 ( 2102 to 3495 ) | 99.806 ( 77.242 to 126.863 ) | 93.215 ( 70.109 to 122.852 ) | -0.28 (-0.32 to -0.24) | 332.258 |
| Kyrgyzstan | Hypertensive heart disease | Both | 563 ( 395 to 758 ) | 1264 ( 867 to 1726 ) | 20.253 ( 14.111 to 27.599 ) | 30.209 ( 20.429 to 41.426 ) | 0.93 (0.56 to 1.29) | 124.512 |
| Lao People's Democratic Republic | Hypertensive heart disease | Both | 921 ( 693 to 1198 ) | 2199 ( 1660 to 2851 ) | 53.53 ( 40.469 to 70.202 ) | 55.41 ( 41.845 to 72.5 ) | 0.05 (0.01 to 0.08) | 138.762 |
| Latvia | Hypertensive heart disease | Both | 199 ( 138 to 279 ) | 1026 ( 647 to 1516 ) | 5.619 ( 3.969 to 7.851 ) | 23.197 ( 14.823 to 33.609 ) | 6.76 (6.16 to 7.38) | 415.578 |
| Lebanon | Hypertensive heart disease | Both | 1226 ( 942 to 1563 ) | 3787 ( 2824 to 4969 ) | 63.079 ( 47.659 to 82 ) | 60.198 ( 45.134 to 78.916 ) | -0.24 (-0.3 to -0.19) | 208.891 |
| Lesotho | Hypertensive heart disease | Both | 492 ( 342 to 686 ) | 608 ( 422 to 822 ) | 65.13 ( 45.655 to 90.165 ) | 66.69 ( 46.527 to 91.346 ) | 0.04 (-0.01 to 0.08) | 23.577 |
| Liberia | Hypertensive heart disease | Both | 902 ( 660 to 1206 ) | 1646 ( 1234 to 2123 ) | 87.817 ( 64.64 to 117.444 ) | 89.623 ( 64.347 to 118.037 ) | 0.08 (0.06 to 0.11) | 82.483 |
| Libya | Hypertensive heart disease | Both | 1537 ( 1208 to 1942 ) | 4059 ( 3134 to 5152 ) | 83.865 ( 64.731 to 108.408 ) | 82.378 ( 61.957 to 106.648 ) | 0.06 (-0.02 to 0.13) | 164.086 |
| Lithuania | Hypertensive heart disease | Both | 321 ( 227 to 437 ) | 1181 ( 761 to 1753 ) | 7.238 ( 5.146 to 9.803 ) | 19.483 ( 12.669 to 28.055 ) | 4.14 (3.9 to 4.38) | 267.913 |
| Luxembourg | Hypertensive heart disease | Both | 64 ( 42 to 92 ) | 231 ( 161 to 321 ) | 12.065 ( 8.013 to 17.016 ) | 20.508 ( 14.519 to 28.37 ) | 2.25 (1.67 to 2.84) | 260.938 |
| Madagascar | Hypertensive heart disease | Both | 4225 ( 3084 to 5522 ) | 8762 ( 6451 to 11398 ) | 97.493 ( 70.121 to 129.546 ) | 95.926 ( 68.162 to 127.5 ) | -0.07 (-0.09 to -0.05) | 107.385 |
| Malawi | Hypertensive heart disease | Both | 2789 ( 2020 to 3712 ) | 5589 ( 4106 to 7219 ) | 87.497 ( 62.933 to 117.356 ) | 86.633 ( 61.756 to 113.602 ) | -0.05 (-0.1 to 0) | 100.394 |
| Malaysia | Hypertensive heart disease | Both | 1778 ( 1367 to 2278 ) | 5042 ( 3762 to 6602 ) | 20.837 ( 15.644 to 27.308 ) | 19.182 ( 14.187 to 25.055 ) | -0.79 (-0.95 to -0.62) | 183.577 |
| Maldives | Hypertensive heart disease | Both | 26 ( 20 to 34 ) | 131 ( 101 to 166 ) | 37.268 ( 28.396 to 49.07 ) | 42.582 ( 31.721 to 55.734 ) | 0.43 (0.37 to 0.48) | 403.846 |
| Mali | Hypertensive heart disease | Both | 2826 ( 2054 to 3746 ) | 6913 ( 5157 to 8904 ) | 87.099 ( 63.497 to 116.863 ) | 92.21 ( 67.846 to 122.034 ) | 0.16 (0.14 to 0.18) | 144.621 |
| Malta | Hypertensive heart disease | Both | 43 ( 31 to 56 ) | 212 ( 147 to 289 ) | 10.703 ( 7.776 to 14.186 ) | 20.025 ( 14.341 to 27.062 ) | 3.32 (2.95 to 3.69) | 393.023 |
| Marshall Islands | Hypertensive heart disease | Both | 6 ( 5 to 8 ) | 11 ( 8 to 14 ) | 42.368 ( 32.366 to 56.433 ) | 39.52 ( 29.419 to 52.088 ) | -0.26 (-0.29 to -0.24) | 83.333 |
| Mauritania | Hypertensive heart disease | Both | 781 ( 562 to 1030 ) | 1839 ( 1375 to 2406 ) | 86.63 ( 62.873 to 114.107 ) | 93.888 ( 70.035 to 125.072 ) | 0.27 (0.26 to 0.29) | 135.467 |
| Mauritius | Hypertensive heart disease | Both | 357 ( 270 to 476 ) | 1186 ( 892 to 1546 ) | 55.298 ( 41.392 to 73.574 ) | 68.474 ( 51.345 to 88.844 ) | 0.79 (0.59 to 0.99) | 232.213 |
| Mexico | Hypertensive heart disease | Both | 13792 ( 10440 to 17765 ) | 38484 ( 27362 to 51788 ) | 35.986 ( 27.052 to 46.661 ) | 32.585 ( 23.266 to 44.492 ) | -0.46 (-0.54 to -0.38) | 179.031 |
| Micronesia (Federated States of) | Hypertensive heart disease | Both | 19 ( 14 to 25 ) | 25 ( 19 to 32 ) | 43.048 ( 32.137 to 56.721 ) | 41.739 ( 30.984 to 54.913 ) | -0.16 (-0.19 to -0.13) | 31.579 |
| Monaco | Hypertensive heart disease | Both | 13 ( 9 to 17 ) | 29 ( 21 to 41 ) | 16.295 ( 11.95 to 21.508 ) | 25.979 ( 19.197 to 35.602 ) | 1.85 (1.57 to 2.14) | 123.077 |
| Mongolia | Hypertensive heart disease | Both | 170 ( 111 to 236 ) | 313 ( 208 to 443 ) | 17.449 ( 11.181 to 24.135 ) | 16.905 ( 10.821 to 24.372 ) | -0.04 (-0.1 to 0.02) | 84.118 |
| Montenegro | Hypertensive heart disease | Both | 113 ( 81 to 151 ) | 191 ( 134 to 269 ) | 19.35 ( 13.768 to 25.935 ) | 19.644 ( 13.957 to 27.227 ) | 0.16 (0.1 to 0.21) | 69.027 |
| Morocco | Hypertensive heart disease | Both | 10249 ( 7947 to 12949 ) | 24197 ( 18080 to 30786 ) | 75.122 ( 56.974 to 95.309 ) | 75.633 ( 56.66 to 96.919 ) | 0.14 (0.08 to 0.19) | 136.091 |
| Mozambique | Hypertensive heart disease | Both | 3915 ( 2806 to 5295 ) | 7437 ( 5356 to 9919 ) | 77.929 ( 55.712 to 106.962 ) | 77.913 ( 54.24 to 105.089 ) | 0 (-0.02 to 0.01) | 89.962 |
| Myanmar | Hypertensive heart disease | Both | 10402 ( 8133 to 13612 ) | 23187 ( 17654 to 29744 ) | 53.016 ( 41.002 to 69.78 ) | 52.985 ( 39.898 to 68.704 ) | -0.07 (-0.14 to -0.01) | 122.909 |
| Namibia | Hypertensive heart disease | Both | 362 ( 253 to 486 ) | 878 ( 629 to 1171 ) | 66.855 ( 46.658 to 92.326 ) | 73.97 ( 52.3 to 101.079 ) | 0.34 (0.32 to 0.37) | 142.541 |
| Nauru | Hypertensive heart disease | Both | 1 ( 1 to 2 ) | 1 ( 1 to 2 ) | 34.81 ( 25.497 to 46.399 ) | 30.355 ( 22.649 to 41.015 ) | -0.43 (-0.48 to -0.38) | 0 |
| Nepal | Hypertensive heart disease | Both | 2391 ( 1879 to 3090 ) | 8002 ( 6043 to 10270 ) | 32.952 ( 24.797 to 43.148 ) | 39.682 ( 30.088 to 50.715 ) | 0.55 (0.44 to 0.67) | 234.672 |
| Netherlands | Hypertensive heart disease | Both | 1246 ( 950 to 1635 ) | 4739 ( 3413 to 6585 ) | 6.002 ( 4.607 to 7.818 ) | 12.461 ( 9.102 to 17.187 ) | 3.27 (2.97 to 3.58) | 280.337 |
| New Zealand | Hypertensive heart disease | Both | 428 ( 299 to 605 ) | 930 ( 654 to 1312 ) | 10.962 ( 7.837 to 15.2 ) | 10.5 ( 7.386 to 14.571 ) | 0.1 (-0.09 to 0.28) | 117.29 |
| Nicaragua | Hypertensive heart disease | Both | 754 ( 584 to 969 ) | 2178 ( 1639 to 2901 ) | 53.566 ( 40.437 to 70.3 ) | 47.772 ( 35.587 to 64.222 ) | -0.51 (-0.56 to -0.46) | 188.859 |
| Niger | Hypertensive heart disease | Both | 1961 ( 1435 to 2529 ) | 6081 ( 4479 to 7919 ) | 86.138 ( 61.973 to 114.522 ) | 87.894 ( 65.126 to 116.969 ) | 0.07 (0.04 to 0.09) | 210.097 |
| Nigeria | Hypertensive heart disease | Both | 31696 ( 24255 to 40199 ) | 65750 ( 50811 to 83059 ) | 81.452 ( 61.908 to 104.28 ) | 83.776 ( 64.022 to 107.066 ) | 0.18 (0.08 to 0.28) | 107.439 |
| Niue | Hypertensive heart disease | Both | 1 ( 1 to 1 ) | 1 ( 1 to 1 ) | 42.073 ( 31.695 to 55.03 ) | 38.835 ( 29.116 to 50.922 ) | -0.3 (-0.33 to -0.26) | 0 |
| North Macedonia | Hypertensive heart disease | Both | 718 ( 518 to 962 ) | 1459 ( 995 to 2076 ) | 43.859 ( 31.534 to 59.858 ) | 47.257 ( 33.357 to 65.755 ) | 0.58 (0.48 to 0.68) | 103.203 |
| Northern Mariana Islands | Hypertensive heart disease | Both | 3 ( 3 to 4 ) | 9 ( 7 to 12 ) | 23.488 ( 17.533 to 30.304 ) | 20.596 ( 14.969 to 27.526 ) | -0.5 (-0.54 to -0.47) | 200 |
| Norway | Hypertensive heart disease | Both | 386 ( 209 to 576 ) | 983 ( 638 to 1457 ) | 5.275 ( 3.062 to 7.709 ) | 8.73 ( 5.732 to 12.808 ) | 2.07 (1.86 to 2.27) | 154.663 |
| Oman | Hypertensive heart disease | Both | 448 ( 366 to 554 ) | 1794 ( 1387 to 2328 ) | 63.351 ( 50.05 to 79.36 ) | 83.401 ( 62.375 to 108.104 ) | 1.17 (1.03 to 1.31) | 300.446 |
| Pakistan | Hypertensive heart disease | Both | 18991 ( 14468 to 24006 ) | 40715 ( 30193 to 53725 ) | 38.996 ( 30.069 to 49.672 ) | 40.753 ( 30.228 to 53.771 ) | 0.06 (0.01 to 0.1) | 114.391 |
| Palau | Hypertensive heart disease | Both | 2 ( 1 to 2 ) | 3 ( 2 to 4 ) | 18.693 ( 14.04 to 24.289 ) | 17.083 ( 12.785 to 22.613 ) | -0.35 (-0.39 to -0.3) | 50 |
| Palestine | Hypertensive heart disease | Both | 634 ( 497 to 804 ) | 1808 ( 1412 to 2298 ) | 75.895 ( 58.431 to 97.997 ) | 76.248 ( 58.469 to 97.973 ) | 0.03 (-0.02 to 0.08) | 185.174 |
| Panama | Hypertensive heart disease | Both | 418 ( 317 to 552 ) | 1973 ( 1480 to 2617 ) | 29.33 ( 21.989 to 39.152 ) | 44.264 ( 33.208 to 58.985 ) | 1.33 (1.18 to 1.49) | 372.01 |
| Papua New Guinea | Hypertensive heart disease | Both | 523 ( 401 to 675 ) | 1457 ( 1125 to 1844 ) | 35.893 ( 27.113 to 46.978 ) | 34.243 ( 25.922 to 43.804 ) | -0.27 (-0.33 to -0.21) | 178.585 |
| Paraguay | Hypertensive heart disease | Both | 1189 ( 924 to 1529 ) | 3495 ( 2668 to 4504 ) | 55.599 ( 42.513 to 72.238 ) | 63.061 ( 47.635 to 82.128 ) | 0.46 (0.38 to 0.54) | 193.944 |
| Peru | Hypertensive heart disease | Both | 4777 ( 3741 to 6127 ) | 13804 ( 10449 to 18087 ) | 41.492 ( 31.847 to 54.281 ) | 41.314 ( 31.014 to 54.428 ) | 0.09 (0.03 to 0.14) | 188.968 |
| Philippines | Hypertensive heart disease | Both | 16358 ( 12844 to 20628 ) | 46964 ( 36238 to 60626 ) | 62.46 ( 48.567 to 79.386 ) | 65.144 ( 49.692 to 84.35 ) | 0.22 (0.19 to 0.25) | 187.101 |
| Poland | Hypertensive heart disease | Both | 16400 ( 12134 to 21822 ) | 44951 ( 33941 to 58541 ) | 38.805 ( 29.246 to 51.032 ) | 59.692 ( 45.409 to 77.088 ) | 1.71 (1.55 to 1.87) | 174.091 |
| Portugal | Hypertensive heart disease | Both | 2332 ( 1721 to 3141 ) | 6905 ( 4956 to 9548 ) | 17.674 ( 13.136 to 23.764 ) | 24.185 ( 17.854 to 32.734 ) | 1.47 (1.3 to 1.65) | 196.098 |
| Puerto Rico | Hypertensive heart disease | Both | 1726 ( 1291 to 2269 ) | 5489 ( 4145 to 7366 ) | 47.105 ( 35.253 to 61.9 ) | 69.785 ( 53.37 to 91.603 ) | 1.54 (1.44 to 1.64) | 218.019 |
| Qatar | Hypertensive heart disease | Both | 65 ( 51 to 83 ) | 678 ( 520 to 899 ) | 46.832 ( 35.559 to 61.571 ) | 56.842 ( 42.313 to 74.776 ) | 0.98 (0.82 to 1.14) | 943.077 |
| Republic of Korea | Hypertensive heart disease | Both | 9223 ( 7000 to 11995 ) | 29926 ( 22838 to 39217 ) | 35.077 ( 25.522 to 46.864 ) | 33.561 ( 25.821 to 43.513 ) | -0.03 (-0.2 to 0.14) | 224.471 |
| Republic of Moldova | Hypertensive heart disease | Both | 428 ( 312 to 570 ) | 1909 ( 1277 to 2726 ) | 9.919 ( 7.261 to 13.253 ) | 31.859 ( 21.496 to 45.389 ) | 5.43 (5.08 to 5.78) | 346.028 |
| Romania | Hypertensive heart disease | Both | 9787 ( 6294 to 14185 ) | 17799 ( 10751 to 25332 ) | 37.373 ( 24.597 to 53.081 ) | 45.203 ( 28.44 to 63.11 ) | 1.57 (1.29 to 1.84) | 81.864 |
| Russian Federation | Hypertensive heart disease | Both | 15660 ( 11114 to 21691 ) | 34141 ( 22992 to 49051 ) | 9.002 ( 6.39 to 12.318 ) | 13.892 ( 9.361 to 19.789 ) | 1.78 (1.49 to 2.07) | 118.014 |
| Rwanda | Hypertensive heart disease | Both | 2116 ( 1540 to 2798 ) | 5228 ( 3762 to 6883 ) | 91.422 ( 66.102 to 122.056 ) | 96.555 ( 69.404 to 129.703 ) | 0.21 (0.19 to 0.24) | 147.07 |
| Saint Kitts and Nevis | Hypertensive heart disease | Both | 18 ( 14 to 25 ) | 39 ( 29 to 51 ) | 46.911 ( 36.095 to 63.136 ) | 67.292 ( 50.059 to 88.129 ) | 1.65 (1.5 to 1.8) | 116.667 |
| Saint Lucia | Hypertensive heart disease | Both | 63 ( 48 to 83 ) | 194 ( 146 to 250 ) | 73.164 ( 55.648 to 94.669 ) | 83.095 ( 62.882 to 107.296 ) | 0.78 (0.68 to 0.88) | 207.937 |
| Saint Vincent and the Grenadines | Hypertensive heart disease | Both | 58 ( 44 to 77 ) | 138 ( 104 to 182 ) | 80.977 ( 61.819 to 106.936 ) | 101.031 ( 76.481 to 132.168 ) | 0.95 (0.85 to 1.05) | 137.931 |
| Samoa | Hypertensive heart disease | Both | 37 ( 28 to 48 ) | 56 ( 43 to 73 ) | 48.728 ( 36.862 to 64.236 ) | 43.176 ( 32.472 to 56.643 ) | -0.49 (-0.55 to -0.43) | 51.351 |
| San Marino | Hypertensive heart disease | Both | 9 ( 6 to 12 ) | 28 ( 20 to 39 ) | 23.772 ( 17.339 to 32.105 ) | 31.562 ( 23.219 to 42.698 ) | 1.46 (1.31 to 1.61) | 211.111 |
| Sao Tome and Principe | Hypertensive heart disease | Both | 54 ( 39 to 71 ) | 93 ( 69 to 120 ) | 90.302 ( 66.27 to 118.153 ) | 94.482 ( 69.135 to 123.837 ) | 0.15 (0.12 to 0.18) | 72.222 |
| Saudi Arabia | Hypertensive heart disease | Both | 5093 ( 4092 to 6385 ) | 16531 ( 12983 to 21233 ) | 91.169 ( 71.068 to 117.944 ) | 94.157 ( 71.446 to 123.659 ) | 0.12 (0.08 to 0.16) | 224.583 |
| Senegal | Hypertensive heart disease | Both | 2366 ( 1720 to 3084 ) | 5994 ( 4539 to 7852 ) | 82.468 ( 60.018 to 109.388 ) | 85.784 ( 64.071 to 114.201 ) | 0.05 (-0.01 to 0.1) | 153.339 |
| Serbia | Hypertensive heart disease | Both | 2559 ( 1680 to 3564 ) | 4992 ( 3054 to 7148 ) | 26.257 ( 16.685 to 36.972 ) | 29.105 ( 18.477 to 40.956 ) | -0.47 (-0.93 to -0.02) | 95.076 |
| Seychelles | Hypertensive heart disease | Both | 53 ( 40 to 68 ) | 96 ( 74 to 125 ) | 91.866 ( 69.85 to 120.324 ) | 92.147 ( 70.707 to 119.662 ) | 0.08 (0.06 to 0.11) | 81.132 |
| Sierra Leone | Hypertensive heart disease | Both | 1508 ( 1076 to 2009 ) | 2894 ( 2136 to 3808 ) | 80.514 ( 57.265 to 107.834 ) | 86.272 ( 62.934 to 114.904 ) | 0.23 (0.21 to 0.25) | 91.91 |
| Singapore | Hypertensive heart disease | Both | 549 ( 407 to 724 ) | 3114 ( 2311 to 4100 ) | 25.521 ( 17.876 to 34.947 ) | 37.28 ( 27.686 to 49.162 ) | 1.69 (1.53 to 1.84) | 467.213 |
| Slovakia | Hypertensive heart disease | Both | 1177 ( 859 to 1580 ) | 3042 ( 2139 to 4199 ) | 20.027 ( 14.731 to 26.794 ) | 31.556 ( 22.564 to 43.23 ) | 2.25 (1.91 to 2.59) | 158.454 |
| Slovenia | Hypertensive heart disease | Both | 660 ( 434 to 912 ) | 2340 ( 1524 to 3250 ) | 27.247 ( 18.021 to 37.475 ) | 48.275 ( 31.752 to 65.925 ) | 2.6 (2.37 to 2.82) | 254.545 |
| Solomon Islands | Hypertensive heart disease | Both | 32 ( 24 to 42 ) | 83 ( 64 to 108 ) | 29.299 ( 21.635 to 38.112 ) | 28.283 ( 21.086 to 37.408 ) | -0.08 (-0.13 to -0.02) | 159.375 |
| Somalia | Hypertensive heart disease | Both | 1571 ( 1168 to 2057 ) | 4027 ( 2921 to 5327 ) | 84.133 ( 61.1 to 111.68 ) | 80.199 ( 57.724 to 107.259 ) | -0.19 (-0.21 to -0.16) | 156.334 |
| South Africa | Hypertensive heart disease | Both | 12042 ( 8857 to 15761 ) | 28922 ( 21428 to 37693 ) | 63.707 ( 47.123 to 83.891 ) | 69.187 ( 51.858 to 90.952 ) | 0.31 (0.26 to 0.37) | 140.176 |
| South Sudan | Hypertensive heart disease | Both | 2266 ( 1638 to 2958 ) | 3144 ( 2319 to 4106 ) | 99.032 ( 71.772 to 131.727 ) | 97.717 ( 70.095 to 132.415 ) | -0.08 (-0.09 to -0.07) | 38.747 |
| Spain | Hypertensive heart disease | Both | 7926 ( 5750 to 10996 ) | 32192 ( 25203 to 39888 ) | 14.482 ( 10.461 to 19.735 ) | 28.253 ( 22.254 to 34.987 ) | 2.46 (2.33 to 2.58) | 306.157 |
| Sri Lanka | Hypertensive heart disease | Both | 5345 ( 4083 to 6957 ) | 13982 ( 10482 to 18529 ) | 56.236 ( 43.254 to 73.75 ) | 53.662 ( 40.696 to 70.195 ) | -0.31 (-0.41 to -0.21) | 161.59 |
| Sudan | Hypertensive heart disease | Both | 6592 ( 5082 to 8375 ) | 14634 ( 11220 to 19112 ) | 76.373 ( 57.575 to 98.453 ) | 77.855 ( 58.316 to 101.705 ) | 0.21 (0.16 to 0.27) | 121.996 |
| Suriname | Hypertensive heart disease | Both | 146 ( 113 to 190 ) | 410 ( 310 to 537 ) | 61.733 ( 47.29 to 80.507 ) | 69.081 ( 52.056 to 90.381 ) | 0.49 (0.45 to 0.52) | 180.822 |
| Sweden | Hypertensive heart disease | Both | 2353 ( 1569 to 3488 ) | 9900 ( 6838 to 14074 ) | 14.304 ( 9.676 to 20.742 ) | 38.674 ( 27.517 to 53.901 ) | 4.56 (4.21 to 4.91) | 320.739 |
| Switzerland | Hypertensive heart disease | Both | 1655 ( 1079 to 2347 ) | 5681 ( 3973 to 7982 ) | 14.925 ( 9.944 to 20.893 ) | 28.086 ( 20.002 to 38.588 ) | 3.4 (2.94 to 3.85) | 243.263 |
| Syrian Arab Republic | Hypertensive heart disease | Both | 2963 ( 2315 to 3754 ) | 6765 ( 4994 to 8807 ) | 61.521 ( 46.086 to 79.549 ) | 58.278 ( 43.358 to 76.63 ) | -0.35 (-0.43 to -0.27) | 128.316 |
| Taiwan (Province of China) | Hypertensive heart disease | Both | 8726 ( 6335 to 11870 ) | 32353 ( 23943 to 42892 ) | 64.024 ( 46.407 to 86.375 ) | 75.114 ( 56.097 to 99.658 ) | 0.82 (0.4 to 1.25) | 270.766 |
| Tajikistan | Hypertensive heart disease | Both | 1426 ( 1032 to 1887 ) | 2696 ( 1933 to 3557 ) | 55.439 ( 39.961 to 74.315 ) | 53.159 ( 36.333 to 72.273 ) | -0.01 (-0.09 to 0.06) | 89.06 |
| Thailand | Hypertensive heart disease | Both | 6425 ( 4988 to 8330 ) | 28864 ( 21357 to 37764 ) | 20.688 ( 15.791 to 27.069 ) | 26.935 ( 20.109 to 35.209 ) | 0.87 (0.76 to 0.99) | 349.245 |
| Timor-Leste | Hypertensive heart disease | Both | 138 ( 107 to 174 ) | 467 ( 351 to 623 ) | 62.418 ( 47.038 to 81.051 ) | 58.047 ( 43.805 to 76.629 ) | -0.36 (-0.41 to -0.32) | 238.406 |
| Togo | Hypertensive heart disease | Both | 873 ( 649 to 1145 ) | 2762 ( 2062 to 3579 ) | 83.727 ( 61.172 to 110.101 ) | 87.269 ( 64.503 to 116.036 ) | 0.13 (0.09 to 0.17) | 216.38 |
| Tokelau | Hypertensive heart disease | Both | 1 ( 0 to 1 ) | 1 ( 0 to 1 ) | 45.012 ( 34.115 to 57.879 ) | 39.138 ( 29.077 to 51.666 ) | -0.52 (-0.56 to -0.47) | 0 |
| Tonga | Hypertensive heart disease | Both | 13 ( 10 to 17 ) | 19 ( 14 to 25 ) | 25.604 ( 19.56 to 33.19 ) | 24.297 ( 17.936 to 32.385 ) | -0.25 (-0.31 to -0.2) | 46.154 |
| Trinidad and Tobago | Hypertensive heart disease | Both | 541 ( 411 to 699 ) | 1204 ( 884 to 1583 ) | 66.158 ( 50.049 to 85.073 ) | 64.382 ( 47.761 to 84.425 ) | 0 (-0.06 to 0.05) | 122.551 |
| Tunisia | Hypertensive heart disease | Both | 4011 ( 3103 to 5062 ) | 11675 ( 8806 to 14835 ) | 86.064 ( 66.478 to 110.367 ) | 92.291 ( 69.354 to 116.25 ) | 0.35 (0.29 to 0.41) | 191.075 |
| Turkey | Hypertensive heart disease | Both | 23771 ( 18449 to 29997 ) | 67758 ( 50908 to 87500 ) | 76.851 ( 59.493 to 98.075 ) | 76.368 ( 57.095 to 98.739 ) | 0.06 (0.01 to 0.12) | 185.045 |
| Turkmenistan | Hypertensive heart disease | Both | 344 ( 239 to 476 ) | 925 ( 633 to 1256 ) | 20.391 ( 13.988 to 28.521 ) | 26.713 ( 17.837 to 37.125 ) | 1.19 (1.1 to 1.28) | 168.895 |
| Tuvalu | Hypertensive heart disease | Both | 2 ( 2 to 3 ) | 4 ( 3 to 5 ) | 43.433 ( 32.357 to 56.333 ) | 38.683 ( 28.645 to 50.564 ) | -0.43 (-0.46 to -0.39) | 100 |
| Uganda | Hypertensive heart disease | Both | 5457 ( 3970 to 7181 ) | 13410 ( 9964 to 17322 ) | 97.066 ( 70.115 to 127.262 ) | 102.99 ( 75.127 to 135.337 ) | 0.29 (0.24 to 0.35) | 145.739 |
| Ukraine | Hypertensive heart disease | Both | 7380 ( 5129 to 10291 ) | 9756 ( 6478 to 13831 ) | 10.439 ( 7.309 to 14.458 ) | 12.134 ( 8.177 to 17.158 ) | 0.48 (0.32 to 0.64) | 32.195 |
| United Arab Emirates | Hypertensive heart disease | Both | 314 ( 246 to 396 ) | 3922 ( 2978 to 5306 ) | 63.041 ( 47.728 to 81.987 ) | 80.471 ( 60.159 to 105.075 ) | 0.77 (0.76 to 0.79) | 1149.045 |
| United Kingdom | Hypertensive heart disease | Both | 5960 ( 4104 to 8242 ) | 15294 ( 10773 to 20788 ) | 6.553 ( 4.68 to 8.937 ) | 11.495 ( 8.188 to 15.387 ) | 2.11 (2.02 to 2.2) | 156.611 |
| United Republic of Tanzania | Hypertensive heart disease | Both | 9046 ( 6515 to 11927 ) | 20958 ( 15318 to 27375 ) | 97.036 ( 69.803 to 130.135 ) | 91.954 ( 67.126 to 123.066 ) | -0.03 (-0.11 to 0.05) | 131.683 |
| United States of America | Hypertensive heart disease | Both | 98998 ( 74427 to 129523 ) | 288489 ( 218565 to 367477 ) | 31.689 ( 24.049 to 41.007 ) | 51.322 ( 40.133 to 64.913 ) | 1.86 (1.73 to 2) | 191.409 |
| United States Virgin Islands | Hypertensive heart disease | Both | 42 ( 32 to 54 ) | 115 ( 83 to 155 ) | 55.098 ( 42.235 to 71.432 ) | 60.837 ( 45.802 to 80.235 ) | 0.32 (0.31 to 0.34) | 173.81 |
| Uruguay | Hypertensive heart disease | Both | 763 ( 544 to 1069 ) | 1736 ( 1097 to 2466 ) | 19.577 ( 14.087 to 27.278 ) | 28.634 ( 19.055 to 39.793 ) | 1.56 (1.49 to 1.63) | 127.523 |
| Uzbekistan | Hypertensive heart disease | Both | 2692 ( 1961 to 3574 ) | 6730 ( 4546 to 9107 ) | 23.961 ( 17.084 to 32.503 ) | 28.577 ( 18.655 to 40.14 ) | 0.75 (0.63 to 0.87) | 150 |
| Vanuatu | Hypertensive heart disease | Both | 16 ( 12 to 21 ) | 45 ( 34 to 59 ) | 30.897 ( 23.335 to 40.479 ) | 30.736 ( 22.904 to 40.007 ) | 0.01 (-0.01 to 0.04) | 181.25 |
| Venezuela (Bolivarian Republic of) | Hypertensive heart disease | Both | 5502 ( 4250 to 7161 ) | 15873 ( 11778 to 20981 ) | 61.162 ( 46.2 to 81.077 ) | 56.404 ( 41.675 to 75.362 ) | -0.31 (-0.33 to -0.29) | 188.495 |
| Viet Nam | Hypertensive heart disease | Both | 26202 ( 20207 to 34339 ) | 59423 ( 45250 to 76582 ) | 69.963 ( 53.704 to 91.831 ) | 66.999 ( 50.46 to 86.588 ) | -0.29 (-0.37 to -0.21) | 126.788 |
| Yemen | Hypertensive heart disease | Both | 3546 ( 2757 to 4474 ) | 11032 ( 8608 to 13991 ) | 81.881 ( 62.729 to 105.912 ) | 82.647 ( 62.983 to 106.874 ) | 0.06 (0.05 to 0.07) | 211.111 |
| Zambia | Hypertensive heart disease | Both | 2255 ( 1628 to 3001 ) | 5804 ( 4332 to 7505 ) | 95.357 ( 69.012 to 129.215 ) | 98.23 ( 71.05 to 129.891 ) | 0.07 (0.04 to 0.09) | 157.384 |
| Zimbabwe | Hypertensive heart disease | Both | 2276 ( 1602 to 3104 ) | 3595 ( 2514 to 4927 ) | 65.62 ( 45.618 to 89.76 ) | 63.869 ( 44.791 to 87.596 ) | -0.17 (-0.23 to -0.11) | 57.953 |

**Table S4 The YLDs of SHF due to HHD in 1990 and 2021 across countries and territories.**

| Locations | cause | sex | 1990 YLDs cases(95% UI) | 2021 YLDs cases(95% UI) | 1990 ASYR(95% UI) | 2021 ASYR(95% UI) | 1990-2021 EAPC（95%CI） | 1990-2021 YLDs cases changes |
| --- | --- | --- | --- | --- | --- | --- | --- | --- |
| Afghanistan | Hypertensive heart disease | Both | 848 ( 529 to 1260 ) | 1338 ( 859 to 1932 ) | 14.392 ( 9.077 to 21.586 ) | 14.005 ( 8.944 to 20.245 ) | -0.04 (-0.06 to -0.02) | 57.783 |
| Albania | Hypertensive heart disease | Both | 87 ( 51 to 130 ) | 210 ( 120 to 329 ) | 4.628 ( 2.711 to 6.959 ) | 4.778 ( 2.791 to 7.474 ) | -0.04 (-0.08 to -0.01) | 141.379 |
| Algeria | Hypertensive heart disease | Both | 1776 ( 1099 to 2582 ) | 5490 ( 3491 to 8041 ) | 15.201 ( 9.616 to 22.025 ) | 16.19 ( 10.397 to 23.899 ) | 0.44 (0.36 to 0.51) | 209.122 |
| American Samoa | Hypertensive heart disease | Both | 1 ( 1 to 2 ) | 2 ( 1 to 3 ) | 6.141 ( 3.767 to 9.353 ) | 5.116 ( 3 to 7.673 ) | -0.82 (-0.97 to -0.66) | 100 |
| Andorra | Hypertensive heart disease | Both | 2 ( 1 to 4 ) | 8 ( 5 to 12 ) | 4.695 ( 2.745 to 7.158 ) | 4.82 ( 2.93 to 7.235 ) | 0.35 (0.28 to 0.42) | 300 |
| Angola | Hypertensive heart disease | Both | 368 ( 227 to 550 ) | 1280 ( 784 to 1923 ) | 12.083 ( 7.422 to 17.991 ) | 13.381 ( 8.122 to 20.351 ) | 0.37 (0.34 to 0.39) | 247.826 |
| Antigua and Barbuda | Hypertensive heart disease | Both | 9 ( 6 to 13 ) | 17 ( 11 to 26 ) | 15.55 ( 9.68 to 23.118 ) | 17.427 ( 10.718 to 25.902 ) | 0.55 (0.44 to 0.66) | 88.889 |
| Argentina | Hypertensive heart disease | Both | 1533 ( 909 to 2363 ) | 3240 ( 1820 to 4903 ) | 4.945 ( 2.949 to 7.62 ) | 5.615 ( 3.185 to 8.452 ) | 0.65 (0.59 to 0.72) | 111.35 |
| Armenia | Hypertensive heart disease | Both | 126 ( 74 to 195 ) | 312 ( 187 to 475 ) | 5.352 ( 3.102 to 8.191 ) | 7.181 ( 4.341 to 10.908 ) | 1.21 (1.1 to 1.31) | 147.619 |
| Australia | Hypertensive heart disease | Both | 338 ( 203 to 498 ) | 1534 ( 926 to 2245 ) | 1.75 ( 1.088 to 2.54 ) | 3.176 ( 1.908 to 4.636 ) | 2.2 (2.09 to 2.31) | 353.846 |
| Austria | Hypertensive heart disease | Both | 609 ( 384 to 884 ) | 1228 ( 741 to 1820 ) | 4.894 ( 3.089 to 7.189 ) | 5.873 ( 3.565 to 8.606 ) | 0.95 (0.73 to 1.17) | 101.642 |
| Azerbaijan | Hypertensive heart disease | Both | 246 ( 144 to 379 ) | 478 ( 283 to 739 ) | 5.556 ( 3.234 to 8.657 ) | 5.507 ( 3.152 to 8.736 ) | 0.02 (-0.03 to 0.07) | 94.309 |
| Bahamas | Hypertensive heart disease | Both | 26 ( 16 to 38 ) | 68 ( 43 to 102 ) | 17.38 ( 10.938 to 26.208 ) | 18.587 ( 11.628 to 27.649 ) | 0.57 (0.48 to 0.66) | 161.538 |
| Bahrain | Hypertensive heart disease | Both | 13 ( 8 to 20 ) | 87 ( 54 to 130 ) | 7.78 ( 4.698 to 11.556 ) | 10.121 ( 6.303 to 14.99 ) | 1.19 (1.08 to 1.3) | 569.231 |
| Bangladesh | Hypertensive heart disease | Both | 2920 ( 1872 to 4222 ) | 9529 ( 5957 to 14102 ) | 7.195 ( 4.598 to 10.569 ) | 7.719 ( 4.888 to 11.304 ) | 0.18 (0.12 to 0.23) | 226.336 |
| Barbados | Hypertensive heart disease | Both | 32 ( 20 to 49 ) | 63 ( 39 to 94 ) | 10.096 ( 6.195 to 15.159 ) | 12.192 ( 7.639 to 18.132 ) | 0.83 (0.77 to 0.9) | 96.875 |
| Belarus | Hypertensive heart disease | Both | 161 ( 90 to 255 ) | 80 ( 43 to 137 ) | 1.265 ( 0.703 to 1.974 ) | 0.497 ( 0.268 to 0.845 ) | -3.74 (-4.13 to -3.34) | -50.311 |
| Belgium | Hypertensive heart disease | Both | 173 ( 100 to 272 ) | 338 ( 196 to 524 ) | 1.1 ( 0.653 to 1.694 ) | 1.306 ( 0.778 to 1.961 ) | 1.54 (1.22 to 1.87) | 95.376 |
| Belize | Hypertensive heart disease | Both | 10 ( 6 to 15 ) | 39 ( 24 to 58 ) | 10.342 ( 6.573 to 15.621 ) | 14.314 ( 8.877 to 21.558 ) | 1.46 (1.34 to 1.57) | 290 |
| Benin | Hypertensive heart disease | Both | 274 ( 171 to 400 ) | 712 ( 449 to 1039 ) | 14.797 ( 9.172 to 21.902 ) | 15.362 ( 9.596 to 22.53 ) | 0.08 (0.06 to 0.1) | 159.854 |
| Bermuda | Hypertensive heart disease | Both | 3 ( 2 to 4 ) | 13 ( 8 to 19 ) | 4.631 ( 2.835 to 6.872 ) | 9.078 ( 5.642 to 13.589 ) | 2.36 (2.25 to 2.47) | 333.333 |
| Bhutan | Hypertensive heart disease | Both | 14 ( 9 to 20 ) | 42 ( 26 to 62 ) | 7.261 ( 4.524 to 10.958 ) | 7.36 ( 4.557 to 10.818 ) | 0.04 (0.02 to 0.05) | 200 |
| Bolivia (Plurinational State of) | Hypertensive heart disease | Both | 215 ( 138 to 315 ) | 709 ( 434 to 1045 ) | 7.354 ( 4.585 to 10.929 ) | 8.4 ( 5.122 to 12.401 ) | 0.44 (0.38 to 0.5) | 229.767 |
| Bosnia and Herzegovina | Hypertensive heart disease | Both | 151 ( 86 to 236 ) | 314 ( 176 to 486 ) | 4.254 ( 2.416 to 6.629 ) | 4.946 ( 2.763 to 7.702 ) | 0.93 (0.79 to 1.08) | 107.947 |
| Botswana | Hypertensive heart disease | Both | 52 ( 31 to 80 ) | 148 ( 90 to 222 ) | 11.293 ( 6.53 to 16.985 ) | 11.725 ( 7.103 to 17.785 ) | 0.12 (0.06 to 0.17) | 184.615 |
| Brazil | Hypertensive heart disease | Both | 6668 ( 4219 to 9703 ) | 22372 ( 13579 to 33287 ) | 8.301 ( 5.17 to 12.017 ) | 9.226 ( 5.578 to 13.698 ) | 0.32 (0.29 to 0.36) | 235.513 |
| Brunei Darussalam | Hypertensive heart disease | Both | 4 ( 2 to 6 ) | 10 ( 6 to 16 ) | 3.271 ( 1.737 to 5.191 ) | 3.217 ( 1.683 to 4.949 ) | -0.2 (-0.27 to -0.13) | 150 |
| Bulgaria | Hypertensive heart disease | Both | 929 ( 525 to 1468 ) | 1992 ( 1087 to 3111 ) | 7.897 ( 4.644 to 12.195 ) | 13.486 ( 7.649 to 20.768 ) | 2.55 (2.24 to 2.86) | 114.424 |
| Burkina Faso | Hypertensive heart disease | Both | 544 ( 341 to 811 ) | 1297 ( 804 to 1950 ) | 14.807 ( 9.385 to 21.813 ) | 15.773 ( 9.738 to 23.526 ) | 0.21 (0.2 to 0.23) | 138.419 |
| Burundi | Hypertensive heart disease | Both | 317 ( 196 to 475 ) | 671 ( 417 to 1012 ) | 15.46 ( 9.416 to 23.038 ) | 16.127 ( 9.832 to 24.373 ) | 0.16 (0.14 to 0.18) | 111.672 |
| Cabo Verde | Hypertensive heart disease | Both | 39 ( 24 to 58 ) | 73 ( 46 to 107 ) | 16.573 ( 10.36 to 24.598 ) | 17.22 ( 10.516 to 25.395 ) | 0.11 (0.09 to 0.12) | 87.179 |
| Cambodia | Hypertensive heart disease | Both | 401 ( 255 to 595 ) | 1172 ( 713 to 1721 ) | 10.614 ( 6.675 to 15.788 ) | 10.918 ( 6.611 to 15.939 ) | 0.1 (0.07 to 0.13) | 192.269 |
| Cameroon | Hypertensive heart disease | Both | 586 ( 373 to 848 ) | 1694 ( 1085 to 2505 ) | 15.646 ( 9.747 to 22.778 ) | 15.546 ( 9.775 to 23.302 ) | 0.05 (-0.03 to 0.14) | 189.078 |
| Canada | Hypertensive heart disease | Both | 712 ( 429 to 1088 ) | 3633 ( 2294 to 5301 ) | 2.203 ( 1.325 to 3.343 ) | 5.423 ( 3.48 to 8.02 ) | 3.51 (3.25 to 3.77) | 410.253 |
| Central African Republic | Hypertensive heart disease | Both | 95 ( 59 to 146 ) | 194 ( 119 to 292 ) | 11.014 ( 6.826 to 16.643 ) | 11.547 ( 7.048 to 17.544 ) | 0.17 (0.15 to 0.18) | 104.211 |
| Chad | Hypertensive heart disease | Both | 359 ( 219 to 532 ) | 747 ( 475 to 1104 ) | 13.886 ( 8.567 to 20.229 ) | 14.875 ( 9.383 to 22.01 ) | 0.23 (0.2 to 0.25) | 108.078 |
| Chile | Hypertensive heart disease | Both | 549 ( 331 to 846 ) | 2038 ( 1202 to 3110 ) | 5.782 ( 3.509 to 8.808 ) | 7.874 ( 4.654 to 12.046 ) | 1.28 (1.19 to 1.37) | 271.22 |
| China | Hypertensive heart disease | Both | 84173 ( 50516 to 124760 ) | 218890 ( 135098 to 330696 ) | 12.139 ( 7.323 to 17.789 ) | 10.75 ( 6.641 to 16.12 ) | -0.66 (-0.89 to -0.44) | 160.048 |
| Colombia | Hypertensive heart disease | Both | 1673 ( 1066 to 2509 ) | 4108 ( 2474 to 6258 ) | 10.491 ( 6.509 to 15.696 ) | 7.406 ( 4.396 to 11.323 ) | -1.57 (-1.69 to -1.44) | 145.547 |
| Comoros | Hypertensive heart disease | Both | 28 ( 17 to 42 ) | 75 ( 47 to 112 ) | 16.461 ( 10.298 to 24.98 ) | 16.936 ( 10.565 to 25.034 ) | 0.07 (0.06 to 0.09) | 167.857 |
| Congo | Hypertensive heart disease | Both | 103 ( 63 to 160 ) | 288 ( 179 to 441 ) | 11.99 ( 7.337 to 18.394 ) | 13.082 ( 7.946 to 20.293 ) | 0.3 (0.28 to 0.33) | 179.612 |
| Cook Islands | Hypertensive heart disease | Both | 2 ( 1 to 3 ) | 4 ( 3 to 7 ) | 18.567 ( 11.584 to 27.597 ) | 17.813 ( 10.753 to 26.501 ) | 0.02 (-0.04 to 0.07) | 100 |
| Costa Rica | Hypertensive heart disease | Both | 132 ( 82 to 194 ) | 480 ( 292 to 715 ) | 7.795 ( 4.759 to 11.634 ) | 8.782 ( 5.305 to 13.131 ) | 0.19 (0.03 to 0.36) | 263.636 |
| Coted'Ivoire | Hypertensive heart disease | Both | 461 ( 298 to 695 ) | 1416 ( 902 to 2101 ) | 14.419 ( 9.033 to 21.603 ) | 14.935 ( 9.219 to 21.915 ) | 0.12 (0.08 to 0.16) | 207.158 |
| Croatia | Hypertensive heart disease | Both | 288 ( 161 to 456 ) | 380 ( 201 to 599 ) | 5.185 ( 2.88 to 8.17 ) | 4.127 ( 2.337 to 6.419 ) | -0.95 (-1.18 to -0.73) | 31.944 |
| Cuba | Hypertensive heart disease | Both | 403 ( 247 to 608 ) | 1943 ( 1169 to 3043 ) | 3.868 ( 2.382 to 5.819 ) | 9.808 ( 5.943 to 15.153 ) | 3.42 (3.21 to 3.64) | 382.134 |
| Cyprus | Hypertensive heart disease | Both | 23 ( 13 to 37 ) | 78 ( 43 to 122 ) | 3.22 ( 1.829 to 5.037 ) | 3.819 ( 2.171 to 5.841 ) | 0.44 (0.24 to 0.63) | 239.13 |
| Czechia | Hypertensive heart disease | Both | 245 ( 135 to 381 ) | 1333 ( 737 to 2098 ) | 1.81 ( 1.018 to 2.782 ) | 5.921 ( 3.32 to 9.083 ) | 4.2 (3.59 to 4.82) | 444.082 |
| Democratic People's Republic of Korea | Hypertensive heart disease | Both | 1158 ( 711 to 1763 ) | 3086 ( 1878 to 4790 ) | 9.003 ( 5.479 to 13.542 ) | 10.167 ( 6.342 to 15.552 ) | 0.47 (0.41 to 0.52) | 166.494 |
| Democratic Republic of the Congo | Hypertensive heart disease | Both | 1524 ( 890 to 2327 ) | 4042 ( 2507 to 6213 ) | 12.44 ( 7.249 to 18.76 ) | 13.422 ( 8.162 to 20.928 ) | 0.28 (0.2 to 0.36) | 165.223 |
| Denmark | Hypertensive heart disease | Both | 66 ( 37 to 103 ) | 197 ( 108 to 316 ) | 0.777 ( 0.443 to 1.208 ) | 1.563 ( 0.878 to 2.471 ) | 3.17 (2.72 to 3.62) | 198.485 |
| Djibouti | Hypertensive heart disease | Both | 19 ( 11 to 28 ) | 89 ( 56 to 132 ) | 16.631 ( 10.207 to 24.414 ) | 16.694 ( 10.434 to 24.806 ) | -0.01 (-0.03 to 0.01) | 368.421 |
| Dominica | Hypertensive heart disease | Both | 9 ( 5 to 13 ) | 12 ( 7 to 18 ) | 14.126 ( 8.722 to 21.333 ) | 14.628 ( 9.035 to 22.066 ) | 0.26 (0.23 to 0.3) | 33.333 |
| Dominican Republic | Hypertensive heart disease | Both | 376 ( 239 to 555 ) | 1038 ( 632 to 1543 ) | 10.74 ( 6.851 to 15.905 ) | 10.584 ( 6.415 to 15.679 ) | -0.22 (-0.27 to -0.16) | 176.064 |
| Ecuador | Hypertensive heart disease | Both | 622 ( 393 to 928 ) | 1751 ( 1069 to 2606 ) | 11.986 ( 7.487 to 17.881 ) | 10.888 ( 6.666 to 16.281 ) | 0.16 (-0.03 to 0.35) | 181.511 |
| Egypt | Hypertensive heart disease | Both | 3259 ( 2054 to 4838 ) | 7114 ( 4399 to 10680 ) | 13.4 ( 8.339 to 19.937 ) | 12.673 ( 7.78 to 19.021 ) | -0.25 (-0.32 to -0.18) | 118.288 |
| El Salvador | Hypertensive heart disease | Both | 136 ( 84 to 203 ) | 280 ( 170 to 429 ) | 4.74 ( 2.886 to 7.172 ) | 4.3 ( 2.605 to 6.635 ) | -0.36 (-0.4 to -0.31) | 105.882 |
| Equatorial Guinea | Hypertensive heart disease | Both | 18 ( 11 to 28 ) | 59 ( 37 to 90 ) | 11.724 ( 7.254 to 17.839 ) | 13.553 ( 8.029 to 20.749 ) | 0.51 (0.47 to 0.56) | 227.778 |
| Eritrea | Hypertensive heart disease | Both | 127 ( 77 to 191 ) | 365 ( 225 to 536 ) | 15.11 ( 9.244 to 21.962 ) | 15.972 ( 9.998 to 23.659 ) | 0.13 (0.11 to 0.15) | 187.402 |
| Estonia | Hypertensive heart disease | Both | 74 ( 44 to 114 ) | 347 ( 186 to 556 ) | 3.702 ( 2.223 to 5.63 ) | 11.892 ( 6.583 to 18.927 ) | 5.76 (5.17 to 6.36) | 368.919 |
| Eswatini | Hypertensive heart disease | Both | 28 ( 17 to 42 ) | 55 ( 34 to 85 ) | 11.467 ( 6.995 to 17.331 ) | 11.771 ( 7.079 to 18.167 ) | 0.06 (0.01 to 0.11) | 96.429 |
| Ethiopia | Hypertensive heart disease | Both | 2353 ( 1483 to 3425 ) | 6513 ( 4186 to 9479 ) | 14.836 ( 9.305 to 22.351 ) | 16.668 ( 10.565 to 24.367 ) | 0.37 (0.35 to 0.38) | 176.796 |
| Fiji | Hypertensive heart disease | Both | 20 ( 12 to 30 ) | 42 ( 25 to 65 ) | 6.745 ( 4.148 to 10.125 ) | 6.541 ( 3.928 to 10.114 ) | -0.23 (-0.28 to -0.18) | 110 |
| Finland | Hypertensive heart disease | Both | 148 ( 81 to 237 ) | 868 ( 478 to 1376 ) | 2.073 ( 1.154 to 3.232 ) | 6.072 ( 3.505 to 9.429 ) | 4.61 (4.32 to 4.91) | 486.486 |
| France | Hypertensive heart disease | Both | 5971 ( 3714 to 8998 ) | 13879 ( 8277 to 20920 ) | 6.701 ( 4.154 to 9.956 ) | 8.199 ( 5.028 to 12.397 ) | 0.56 (0.38 to 0.75) | 132.44 |
| Gabon | Hypertensive heart disease | Both | 69 ( 40 to 103 ) | 126 ( 78 to 189 ) | 13.473 ( 8.131 to 20.27 ) | 14.2 ( 8.722 to 21.347 ) | 0.18 (0.17 to 0.19) | 82.609 |
| Gambia | Hypertensive heart disease | Both | 44 ( 28 to 64 ) | 130 ( 80 to 193 ) | 14.304 ( 9.001 to 20.713 ) | 14.688 ( 9.14 to 21.976 ) | 0.11 (0.08 to 0.13) | 195.455 |
| Georgia | Hypertensive heart disease | Both | 260 ( 149 to 403 ) | 519 ( 235 to 884 ) | 4.382 ( 2.525 to 6.772 ) | 8.519 ( 4.042 to 14.475 ) | 3.78 (3.13 to 4.44) | 99.615 |
| Germany | Hypertensive heart disease | Both | 5850 ( 3308 to 8863 ) | 14267 ( 8094 to 21779 ) | 4.486 ( 2.618 to 6.669 ) | 6.317 ( 3.564 to 9.769 ) | 2.63 (2.1 to 3.16) | 143.88 |
| Ghana | Hypertensive heart disease | Both | 790 ( 487 to 1193 ) | 2205 ( 1402 to 3340 ) | 15.269 ( 9.459 to 22.581 ) | 15.546 ( 9.515 to 23.178 ) | -0.02 (-0.04 to 0) | 179.114 |
| Greece | Hypertensive heart disease | Both | 336 ( 196 to 514 ) | 651 ( 364 to 994 ) | 2.278 ( 1.342 to 3.489 ) | 2.32 ( 1.34 to 3.467 ) | 0.08 (-0.04 to 0.19) | 93.75 |
| Greenland | Hypertensive heart disease | Both | 1 ( 1 to 2 ) | 3 ( 2 to 5 ) | 3.607 ( 2.144 to 5.39 ) | 4.802 ( 2.809 to 7.249 ) | 1.14 (0.97 to 1.3) | 200 |
| Grenada | Hypertensive heart disease | Both | 8 ( 5 to 12 ) | 13 ( 8 to 19 ) | 9.823 ( 5.94 to 15.291 ) | 12.36 ( 7.537 to 18.729 ) | 0.97 (0.91 to 1.03) | 62.5 |
| Guam | Hypertensive heart disease | Both | 8 ( 5 to 12 ) | 14 ( 8 to 21 ) | 11.885 ( 7.346 to 17.771 ) | 6.72 ( 3.965 to 10.198 ) | -2.15 (-2.32 to -1.98) | 75 |
| Guatemala | Hypertensive heart disease | Both | 112 ( 69 to 169 ) | 367 ( 213 to 561 ) | 3.784 ( 2.313 to 5.772 ) | 3.555 ( 2.087 to 5.463 ) | -0.09 (-0.15 to -0.03) | 227.679 |
| Guinea | Hypertensive heart disease | Both | 445 ( 277 to 646 ) | 773 ( 482 to 1136 ) | 14.883 ( 9.373 to 21.742 ) | 15.239 ( 9.408 to 22.487 ) | 0.03 (0 to 0.05) | 73.708 |
| Guinea-Bissau | Hypertensive heart disease | Both | 44 ( 27 to 64 ) | 83 ( 52 to 124 ) | 13.524 ( 8.307 to 20.185 ) | 13.965 ( 8.685 to 20.625 ) | 0.09 (0.07 to 0.1) | 88.636 |
| Guyana | Hypertensive heart disease | Both | 49 ( 31 to 73 ) | 84 ( 53 to 126 ) | 13.858 ( 8.61 to 20.962 ) | 14.86 ( 9.203 to 22.398 ) | 0.2 (0.1 to 0.29) | 71.429 |
| Haiti | Hypertensive heart disease | Both | 238 ( 151 to 356 ) | 550 ( 343 to 799 ) | 8.882 ( 5.542 to 12.892 ) | 8.967 ( 5.332 to 13.229 ) | 0.01 (-0.02 to 0.04) | 131.092 |
| Honduras | Hypertensive heart disease | Both | 210 ( 131 to 309 ) | 643 ( 394 to 976 ) | 10.923 ( 6.798 to 16.193 ) | 10.943 ( 6.528 to 16.659 ) | -0.12 (-0.16 to -0.08) | 206.19 |
| Hungary | Hypertensive heart disease | Both | 785 ( 428 to 1274 ) | 1674 ( 915 to 2675 ) | 5.41 ( 3.064 to 8.525 ) | 8.047 ( 4.503 to 12.566 ) | 1.67 (1.51 to 1.82) | 113.248 |
| Iceland | Hypertensive heart disease | Both | 2 ( 1 to 3 ) | 8 ( 5 to 13 ) | 0.697 ( 0.381 to 1.076 ) | 1.376 ( 0.753 to 2.156 ) | 2.48 (2.16 to 2.81) | 300 |
| India | Hypertensive heart disease | Both | 19230 ( 12078 to 28247 ) | 60953 ( 37608 to 91429 ) | 5.273 ( 3.305 to 7.704 ) | 5.782 ( 3.561 to 8.573 ) | 0.29 (0.27 to 0.32) | 216.968 |
| Indonesia | Hypertensive heart disease | Both | 8585 ( 5387 to 12521 ) | 21309 ( 13410 to 31582 ) | 10.473 ( 6.562 to 15.108 ) | 10.582 ( 6.58 to 15.54 ) | -0.07 (-0.11 to -0.04) | 148.212 |
| Iran (Islamic Republic of) | Hypertensive heart disease | Both | 2941 ( 1894 to 4202 ) | 10410 ( 6626 to 15316 ) | 12.551 ( 8.062 to 18.061 ) | 13.985 ( 8.739 to 20.842 ) | 0.52 (0.45 to 0.58) | 253.961 |
| Iraq | Hypertensive heart disease | Both | 907 ( 584 to 1319 ) | 2414 ( 1526 to 3568 ) | 11.488 ( 7.294 to 16.901 ) | 10.926 ( 6.775 to 16.037 ) | -0.19 (-0.22 to -0.17) | 166.152 |
| Ireland | Hypertensive heart disease | Both | 33 ( 18 to 53 ) | 138 ( 77 to 211 ) | 0.83 ( 0.488 to 1.293 ) | 1.677 ( 0.94 to 2.558 ) | 3.15 (2.85 to 3.46) | 318.182 |
| Israel | Hypertensive heart disease | Both | 66 ( 38 to 106 ) | 266 ( 159 to 415 ) | 1.395 ( 0.831 to 2.199 ) | 2.029 ( 1.221 to 3.191 ) | 1.99 (1.76 to 2.22) | 303.03 |
| Italy | Hypertensive heart disease | Both | 5158 ( 2857 to 7822 ) | 16452 ( 9871 to 24760 ) | 5.69 ( 3.179 to 8.532 ) | 9.582 ( 5.789 to 14.128 ) | 2.24 (1.6 to 2.88) | 218.961 |
| Jamaica | Hypertensive heart disease | Both | 348 ( 214 to 522 ) | 564 ( 357 to 843 ) | 18.557 ( 11.431 to 27.566 ) | 17.868 ( 11.243 to 26.997 ) | 0.14 (0.05 to 0.24) | 62.069 |
| Japan | Hypertensive heart disease | Both | 4260 ( 2477 to 6556 ) | 10962 ( 6554 to 16836 ) | 2.758 ( 1.638 to 4.197 ) | 2.542 ( 1.528 to 3.84 ) | -0.66 (-0.92 to -0.41) | 157.324 |
| Jordan | Hypertensive heart disease | Both | 219 ( 142 to 318 ) | 1357 ( 853 to 1993 ) | 17.062 ( 10.751 to 25.009 ) | 19.201 ( 11.818 to 28.588 ) | 0.68 (0.6 to 0.76) | 519.635 |
| Kazakhstan | Hypertensive heart disease | Both | 444 ( 258 to 669 ) | 529 ( 300 to 844 ) | 3.714 ( 2.128 to 5.646 ) | 3.289 ( 1.831 to 5.226 ) | -0.47 (-0.99 to 0.04) | 19.144 |
| Kenya | Hypertensive heart disease | Both | 1144 ( 724 to 1707 ) | 3273 ( 2092 to 4768 ) | 15.979 ( 10.052 to 23.474 ) | 16.553 ( 10.611 to 24.201 ) | -0.36 (-0.54 to -0.18) | 186.101 |
| Kiribati | Hypertensive heart disease | Both | 1 ( 1 to 2 ) | 2 ( 2 to 4 ) | 4.298 ( 2.591 to 6.532 ) | 4.163 ( 2.524 to 6.263 ) | -0.13 (-0.17 to -0.1) | 100 |
| Kuwait | Hypertensive heart disease | Both | 108 ( 68 to 156 ) | 465 ( 292 to 678 ) | 17.147 ( 10.825 to 25.13 ) | 16.05 ( 9.982 to 24.043 ) | -0.27 (-0.31 to -0.23) | 330.556 |
| Kyrgyzstan | Hypertensive heart disease | Both | 97 ( 57 to 150 ) | 219 ( 125 to 333 ) | 3.488 ( 2.055 to 5.317 ) | 5.206 ( 2.898 to 8.103 ) | 0.92 (0.56 to 1.29) | 125.773 |
| Lao People's Democratic Republic | Hypertensive heart disease | Both | 159 ( 99 to 238 ) | 380 ( 232 to 569 ) | 9.144 ( 5.684 to 13.831 ) | 9.513 ( 5.744 to 14.417 ) | 0.06 (0.03 to 0.1) | 138.994 |
| Latvia | Hypertensive heart disease | Both | 35 ( 19 to 55 ) | 177 ( 93 to 290 ) | 0.98 ( 0.558 to 1.546 ) | 4.015 ( 2.111 to 6.513 ) | 6.74 (6.13 to 7.35) | 405.714 |
| Lebanon | Hypertensive heart disease | Both | 211 ( 132 to 310 ) | 651 ( 406 to 947 ) | 10.79 ( 6.671 to 16.114 ) | 10.363 ( 6.431 to 15.247 ) | -0.23 (-0.28 to -0.17) | 208.531 |
| Lesotho | Hypertensive heart disease | Both | 85 ( 51 to 129 ) | 105 ( 64 to 162 ) | 11.134 ( 6.628 to 16.962 ) | 11.45 ( 7.04 to 17.6 ) | 0.04 (0 to 0.09) | 23.529 |
| Liberia | Hypertensive heart disease | Both | 154 ( 94 to 230 ) | 284 ( 178 to 419 ) | 14.904 ( 9.25 to 21.929 ) | 15.325 ( 9.559 to 22.55 ) | 0.1 (0.08 to 0.13) | 84.416 |
| Libya | Hypertensive heart disease | Both | 264 ( 172 to 390 ) | 701 ( 451 to 1016 ) | 14.339 ( 9.245 to 21.141 ) | 14.145 ( 8.826 to 20.869 ) | 0.07 (-0.01 to 0.14) | 165.53 |
| Lithuania | Hypertensive heart disease | Both | 56 ( 33 to 86 ) | 203 ( 108 to 327 ) | 1.258 ( 0.742 to 1.937 ) | 3.362 ( 1.806 to 5.347 ) | 4.11 (3.88 to 4.35) | 262.5 |
| Luxembourg | Hypertensive heart disease | Both | 11 ( 6 to 18 ) | 40 ( 23 to 61 ) | 2.102 ( 1.171 to 3.248 ) | 3.56 ( 2.041 to 5.375 ) | 2.25 (1.66 to 2.83) | 263.636 |
| Madagascar | Hypertensive heart disease | Both | 722 ( 453 to 1061 ) | 1507 ( 938 to 2266 ) | 16.554 ( 10.26 to 24.625 ) | 16.336 ( 10.202 to 24.492 ) | -0.05 (-0.08 to -0.03) | 108.726 |
| Malawi | Hypertensive heart disease | Both | 479 ( 293 to 734 ) | 959 ( 604 to 1450 ) | 14.863 ( 9.021 to 22.728 ) | 14.729 ( 9.105 to 22.02 ) | -0.03 (-0.08 to 0.03) | 100.209 |
| Malaysia | Hypertensive heart disease | Both | 305 ( 188 to 455 ) | 867 ( 527 to 1337 ) | 3.552 ( 2.128 to 5.326 ) | 3.281 ( 1.977 to 5.036 ) | -0.77 (-0.94 to -0.61) | 184.262 |
| Maldives | Hypertensive heart disease | Both | 5 ( 3 to 7 ) | 23 ( 14 to 33 ) | 6.361 ( 3.886 to 9.727 ) | 7.327 ( 4.425 to 10.845 ) | 0.45 (0.4 to 0.5) | 360 |
| Mali | Hypertensive heart disease | Both | 483 ( 297 to 726 ) | 1190 ( 741 to 1753 ) | 14.745 ( 9.21 to 21.827 ) | 15.727 ( 9.852 to 23.28 ) | 0.19 (0.17 to 0.2) | 146.377 |
| Malta | Hypertensive heart disease | Both | 7 ( 4 to 12 ) | 37 ( 21 to 56 ) | 1.855 ( 1.125 to 2.955 ) | 3.501 ( 2.009 to 5.272 ) | 3.32 (2.96 to 3.69) | 428.571 |
| Marshall Islands | Hypertensive heart disease | Both | 1 ( 1 to 2 ) | 2 ( 1 to 3 ) | 7.224 ( 4.566 to 10.893 ) | 6.763 ( 4.245 to 10.244 ) | -0.25 (-0.28 to -0.22) | 100 |
| Mauritania | Hypertensive heart disease | Both | 134 ( 82 to 198 ) | 317 ( 196 to 473 ) | 14.73 ( 9.18 to 21.49 ) | 16.094 ( 9.887 to 24.073 ) | 0.29 (0.28 to 0.31) | 136.567 |
| Mauritius | Hypertensive heart disease | Both | 61 ( 38 to 92 ) | 203 ( 125 to 305 ) | 9.422 ( 5.791 to 14.098 ) | 11.696 ( 7.168 to 17.507 ) | 0.79 (0.6 to 0.99) | 232.787 |
| Mexico | Hypertensive heart disease | Both | 2375 ( 1469 to 3477 ) | 6623 ( 4050 to 10140 ) | 6.161 ( 3.787 to 9.014 ) | 5.595 ( 3.414 to 8.502 ) | -0.46 (-0.54 to -0.37) | 178.863 |
| Micronesia (Federated States of) | Hypertensive heart disease | Both | 3 ( 2 to 5 ) | 4 ( 3 to 6 ) | 7.338 ( 4.398 to 11.117 ) | 7.159 ( 4.351 to 10.661 ) | -0.14 (-0.17 to -0.11) | 33.333 |
| Monaco | Hypertensive heart disease | Both | 2 ( 1 to 3 ) | 5 ( 3 to 8 ) | 2.829 ( 1.692 to 4.268 ) | 4.537 ( 2.773 to 6.898 ) | 1.87 (1.58 to 2.15) | 150 |
| Mongolia | Hypertensive heart disease | Both | 29 ( 16 to 47 ) | 54 ( 31 to 84 ) | 2.989 ( 1.601 to 4.839 ) | 2.917 ( 1.582 to 4.622 ) | -0.04 (-0.1 to 0.02) | 86.207 |
| Montenegro | Hypertensive heart disease | Both | 20 ( 11 to 30 ) | 33 ( 19 to 52 ) | 3.355 ( 1.947 to 5.164 ) | 3.409 ( 1.975 to 5.249 ) | 0.15 (0.1 to 0.2) | 65 |
| Morocco | Hypertensive heart disease | Both | 1763 ( 1121 to 2647 ) | 4176 ( 2544 to 6377 ) | 12.851 ( 8.194 to 19.383 ) | 13.007 ( 7.939 to 19.735 ) | 0.15 (0.1 to 0.2) | 136.869 |
| Mozambique | Hypertensive heart disease | Both | 667 ( 414 to 982 ) | 1277 ( 784 to 1935 ) | 13.158 ( 7.905 to 19.305 ) | 13.247 ( 8.093 to 19.841 ) | 0.01 (0 to 0.03) | 91.454 |
| Myanmar | Hypertensive heart disease | Both | 1773 ( 1133 to 2716 ) | 3984 ( 2544 to 6003 ) | 8.952 ( 5.728 to 13.738 ) | 9.054 ( 5.705 to 13.76 ) | -0.03 (-0.1 to 0.03) | 124.704 |
| Namibia | Hypertensive heart disease | Both | 63 ( 36 to 95 ) | 152 ( 94 to 235 ) | 11.477 ( 6.814 to 17.249 ) | 12.746 ( 7.668 to 19.557 ) | 0.36 (0.33 to 0.4) | 141.27 |
| Nauru | Hypertensive heart disease | Both | 0 ( 0 to 0 ) | 0 ( 0 to 0 ) | 5.944 ( 3.632 to 9.275 ) | 5.181 ( 3.13 to 7.71 ) | -0.43 (-0.47 to -0.38) | #NUM! |
| Nepal | Hypertensive heart disease | Both | 404 ( 258 to 611 ) | 1361 ( 840 to 1985 ) | 5.518 ( 3.559 to 8.11 ) | 6.712 ( 4.201 to 9.747 ) | 0.59 (0.48 to 0.7) | 236.881 |
| Netherlands | Hypertensive heart disease | Both | 218 ( 129 to 335 ) | 821 ( 460 to 1243 ) | 1.048 ( 0.629 to 1.596 ) | 2.164 ( 1.233 to 3.232 ) | 3.26 (2.95 to 3.57) | 276.606 |
| New Zealand | Hypertensive heart disease | Both | 74 ( 40 to 116 ) | 161 ( 93 to 255 ) | 1.9 ( 1.062 to 2.923 ) | 1.824 ( 1.059 to 2.856 ) | 0.1 (-0.09 to 0.29) | 117.568 |
| Nicaragua | Hypertensive heart disease | Both | 130 ( 80 to 191 ) | 375 ( 228 to 562 ) | 9.158 ( 5.73 to 13.614 ) | 8.201 ( 5.026 to 12.342 ) | -0.5 (-0.55 to -0.45) | 188.462 |
| Niger | Hypertensive heart disease | Both | 336 ( 212 to 491 ) | 1048 ( 649 to 1589 ) | 14.586 ( 9.004 to 21.371 ) | 15.017 ( 9.174 to 22.699 ) | 0.09 (0.07 to 0.12) | 211.905 |
| Nigeria | Hypertensive heart disease | Both | 5409 ( 3491 to 7832 ) | 11280 ( 7322 to 16382 ) | 13.798 ( 8.839 to 20.206 ) | 14.252 ( 9.251 to 20.583 ) | 0.2 (0.1 to 0.29) | 108.541 |
| Niue | Hypertensive heart disease | Both | 0 ( 0 to 0 ) | 0 ( 0 to 0 ) | 7.204 ( 4.478 to 10.746 ) | 6.658 ( 4.053 to 9.983 ) | -0.3 (-0.33 to -0.27) | #NUM! |
| North Macedonia | Hypertensive heart disease | Both | 124 ( 74 to 189 ) | 253 ( 142 to 396 ) | 7.568 ( 4.471 to 11.601 ) | 8.175 ( 4.64 to 12.452 ) | 0.59 (0.48 to 0.69) | 104.032 |
| Northern Mariana Islands | Hypertensive heart disease | Both | 1 ( 0 to 1 ) | 2 ( 1 to 2 ) | 4.041 ( 2.473 to 6.136 ) | 3.546 ( 2.179 to 5.444 ) | -0.51 (-0.54 to -0.47) | 100 |
| Norway | Hypertensive heart disease | Both | 67 ( 33 to 105 ) | 171 ( 94 to 277 ) | 0.916 ( 0.473 to 1.418 ) | 1.521 ( 0.83 to 2.43 ) | 2.07 (1.87 to 2.28) | 155.224 |
| Oman | Hypertensive heart disease | Both | 78 ( 50 to 111 ) | 312 ( 198 to 462 ) | 10.767 ( 6.911 to 15.821 ) | 14.29 ( 9.182 to 21.24 ) | 1.19 (1.05 to 1.33) | 300 |
| Pakistan | Hypertensive heart disease | Both | 3205 ( 1958 to 4811 ) | 6919 ( 4248 to 10436 ) | 6.531 ( 4.051 to 9.543 ) | 6.853 ( 4.119 to 10.302 ) | 0.07 (0.03 to 0.12) | 115.881 |
| Palau | Hypertensive heart disease | Both | 0 ( 0 to 0 ) | 1 ( 0 to 1 ) | 3.203 ( 1.943 to 4.781 ) | 2.931 ( 1.799 to 4.459 ) | -0.34 (-0.39 to -0.29) | #NUM! |
| Palestine | Hypertensive heart disease | Both | 109 ( 70 to 163 ) | 313 ( 199 to 457 ) | 12.954 ( 8.248 to 19.227 ) | 13.105 ( 8.045 to 19.577 ) | 0.05 (0 to 0.1) | 187.156 |
| Panama | Hypertensive heart disease | Both | 72 ( 44 to 111 ) | 338 ( 203 to 509 ) | 5.022 ( 3.047 to 7.765 ) | 7.583 ( 4.552 to 11.448 ) | 1.33 (1.18 to 1.48) | 369.444 |
| Papua New Guinea | Hypertensive heart disease | Both | 90 ( 55 to 136 ) | 252 ( 157 to 379 ) | 6.092 ( 3.77 to 9.079 ) | 5.87 ( 3.522 to 9.002 ) | -0.25 (-0.31 to -0.18) | 180 |
| Paraguay | Hypertensive heart disease | Both | 204 ( 130 to 299 ) | 601 ( 364 to 912 ) | 9.515 ( 6.084 to 13.956 ) | 10.816 ( 6.584 to 16.444 ) | 0.47 (0.38 to 0.55) | 194.608 |
| Peru | Hypertensive heart disease | Both | 821 ( 517 to 1201 ) | 2378 ( 1460 to 3637 ) | 7.105 ( 4.443 to 10.488 ) | 7.114 ( 4.338 to 10.948 ) | 0.11 (0.06 to 0.17) | 189.647 |
| Philippines | Hypertensive heart disease | Both | 2805 ( 1792 to 4053 ) | 8051 ( 5068 to 11984 ) | 10.631 ( 6.742 to 15.39 ) | 11.107 ( 6.982 to 16.442 ) | 0.22 (0.19 to 0.25) | 187.023 |
| Poland | Hypertensive heart disease | Both | 2839 ( 1687 to 4280 ) | 7757 ( 4667 to 11581 ) | 6.709 ( 4.024 to 10.032 ) | 10.315 ( 6.273 to 15.194 ) | 1.71 (1.55 to 1.86) | 173.23 |
| Portugal | Hypertensive heart disease | Both | 405 ( 238 to 603 ) | 1197 ( 694 to 1818 ) | 3.063 ( 1.849 to 4.508 ) | 4.212 ( 2.506 to 6.172 ) | 1.47 (1.3 to 1.65) | 195.556 |
| Puerto Rico | Hypertensive heart disease | Both | 299 ( 185 to 448 ) | 947 ( 572 to 1457 ) | 8.14 ( 5.096 to 12.1 ) | 12.098 ( 7.399 to 18.505 ) | 1.55 (1.45 to 1.65) | 216.722 |
| Qatar | Hypertensive heart disease | Both | 11 ( 7 to 17 ) | 119 ( 72 to 182 ) | 8.02 ( 4.988 to 12.016 ) | 9.799 ( 6.128 to 14.867 ) | 1.01 (0.85 to 1.18) | 981.818 |
| Republic of Korea | Hypertensive heart disease | Both | 1611 ( 1000 to 2368 ) | 5196 ( 3209 to 7698 ) | 6.07 ( 3.614 to 8.996 ) | 5.825 ( 3.643 to 8.533 ) | -0.03 (-0.2 to 0.13) | 222.533 |
| Republic of Moldova | Hypertensive heart disease | Both | 74 ( 44 to 120 ) | 329 ( 186 to 521 ) | 1.714 ( 1.016 to 2.69 ) | 5.493 ( 3.127 to 8.717 ) | 5.41 (5.06 to 5.75) | 344.595 |
| Romania | Hypertensive heart disease | Both | 1693 ( 887 to 2744 ) | 3082 ( 1596 to 4988 ) | 6.456 ( 3.5 to 10.165 ) | 7.844 ( 4.204 to 12.618 ) | 1.58 (1.3 to 1.85) | 82.044 |
| Russian Federation | Hypertensive heart disease | Both | 2707 ( 1609 to 4198 ) | 5872 ( 3330 to 9376 ) | 1.554 ( 0.931 to 2.368 ) | 2.39 ( 1.364 to 3.832 ) | 1.76 (1.47 to 2.05) | 116.919 |
| Rwanda | Hypertensive heart disease | Both | 364 ( 222 to 546 ) | 903 ( 575 to 1345 ) | 15.563 ( 9.724 to 23.173 ) | 16.559 ( 10.318 to 24.696 ) | 0.24 (0.21 to 0.26) | 148.077 |
| Saint Kitts and Nevis | Hypertensive heart disease | Both | 3 ( 2 to 5 ) | 7 ( 4 to 10 ) | 8.052 ( 4.997 to 12.148 ) | 11.616 ( 7.113 to 17.788 ) | 1.67 (1.52 to 1.82) | 133.333 |
| Saint Lucia | Hypertensive heart disease | Both | 11 ( 7 to 17 ) | 34 ( 21 to 51 ) | 12.591 ( 7.713 to 18.907 ) | 14.365 ( 8.905 to 21.764 ) | 0.78 (0.68 to 0.88) | 209.091 |
| Saint Vincent and the Grenadines | Hypertensive heart disease | Both | 10 ( 6 to 15 ) | 24 ( 15 to 36 ) | 13.953 ( 8.429 to 21.018 ) | 17.477 ( 10.772 to 26.266 ) | 0.96 (0.86 to 1.06) | 140 |
| Samoa | Hypertensive heart disease | Both | 6 ( 4 to 10 ) | 10 ( 6 to 15 ) | 8.36 ( 5.255 to 12.715 ) | 7.42 ( 4.599 to 11.338 ) | -0.49 (-0.56 to -0.43) | 66.667 |
| San Marino | Hypertensive heart disease | Both | 2 ( 1 to 2 ) | 5 ( 3 to 7 ) | 4.125 ( 2.443 to 6.286 ) | 5.513 ( 3.251 to 8.368 ) | 1.46 (1.31 to 1.61) | 150 |
| Sao Tome and Principe | Hypertensive heart disease | Both | 9 ( 6 to 14 ) | 16 ( 10 to 24 ) | 15.456 ( 9.461 to 23.063 ) | 16.224 ( 10.305 to 24.1 ) | 0.17 (0.14 to 0.2) | 77.778 |
| Saudi Arabia | Hypertensive heart disease | Both | 873 ( 560 to 1276 ) | 2875 ( 1827 to 4176 ) | 15.39 ( 9.794 to 22.485 ) | 16.105 ( 9.919 to 23.543 ) | 0.17 (0.13 to 0.2) | 229.324 |
| Senegal | Hypertensive heart disease | Both | 405 ( 250 to 607 ) | 1031 ( 645 to 1520 ) | 14.016 ( 8.466 to 20.601 ) | 14.687 ( 9.059 to 21.748 ) | 0.07 (0.02 to 0.13) | 154.568 |
| Serbia | Hypertensive heart disease | Both | 444 ( 245 to 699 ) | 864 ( 449 to 1336 ) | 4.543 ( 2.526 to 7.175 ) | 5.043 ( 2.66 to 7.745 ) | -0.46 (-0.91 to -0.01) | 94.595 |
| Seychelles | Hypertensive heart disease | Both | 9 ( 5 to 13 ) | 17 ( 10 to 25 ) | 15.682 ( 9.506 to 23.274 ) | 15.803 ( 9.798 to 23.568 ) | 0.1 (0.07 to 0.12) | 88.889 |
| Sierra Leone | Hypertensive heart disease | Both | 258 ( 163 to 386 ) | 499 ( 313 to 738 ) | 13.692 ( 8.604 to 20.199 ) | 14.757 ( 9.313 to 21.922 ) | 0.25 (0.23 to 0.28) | 93.411 |
| Singapore | Hypertensive heart disease | Both | 97 ( 58 to 147 ) | 543 ( 340 to 822 ) | 4.451 ( 2.621 to 6.822 ) | 6.498 ( 4.076 to 9.845 ) | 1.7 (1.54 to 1.86) | 459.794 |
| Slovakia | Hypertensive heart disease | Both | 204 ( 118 to 317 ) | 528 ( 296 to 837 ) | 3.466 ( 2.028 to 5.318 ) | 5.483 ( 3.176 to 8.619 ) | 2.25 (1.91 to 2.59) | 158.824 |
| Slovenia | Hypertensive heart disease | Both | 115 ( 62 to 180 ) | 405 ( 222 to 635 ) | 4.743 ( 2.587 to 7.402 ) | 8.366 ( 4.741 to 12.936 ) | 2.59 (2.37 to 2.82) | 252.174 |
| Solomon Islands | Hypertensive heart disease | Both | 6 ( 3 to 8 ) | 14 ( 9 to 21 ) | 5.004 ( 3.063 to 7.47 ) | 4.833 ( 2.909 to 7.171 ) | -0.06 (-0.11 to 0) | 133.333 |
| Somalia | Hypertensive heart disease | Both | 271 ( 165 to 406 ) | 692 ( 427 to 1038 ) | 14.266 ( 8.645 to 21.286 ) | 13.597 ( 8.325 to 20.111 ) | -0.18 (-0.21 to -0.15) | 155.351 |
| South Africa | Hypertensive heart disease | Both | 2071 ( 1276 to 3091 ) | 4991 ( 3096 to 7415 ) | 10.915 ( 6.668 to 16.156 ) | 11.893 ( 7.32 to 17.739 ) | 0.32 (0.27 to 0.38) | 140.995 |
| South Sudan | Hypertensive heart disease | Both | 385 ( 240 to 586 ) | 538 ( 338 to 816 ) | 16.65 ( 10.41 to 25.077 ) | 16.534 ( 10.338 to 24.851 ) | -0.06 (-0.08 to -0.05) | 39.74 |
| Spain | Hypertensive heart disease | Both | 1372 ( 772 to 2160 ) | 5551 ( 3509 to 8101 ) | 2.503 ( 1.42 to 3.859 ) | 4.898 ( 3.154 to 7.109 ) | 2.46 (2.33 to 2.58) | 304.592 |
| Sri Lanka | Hypertensive heart disease | Both | 918 ( 569 to 1354 ) | 2401 ( 1448 to 3564 ) | 9.595 ( 5.878 to 14.211 ) | 9.188 ( 5.655 to 13.521 ) | -0.29 (-0.39 to -0.19) | 161.547 |
| Sudan | Hypertensive heart disease | Both | 1127 ( 703 to 1681 ) | 2521 ( 1588 to 3641 ) | 12.937 ( 8.13 to 19.141 ) | 13.316 ( 8.256 to 19.208 ) | 0.25 (0.19 to 0.31) | 123.691 |
| Suriname | Hypertensive heart disease | Both | 25 ( 16 to 38 ) | 71 ( 43 to 104 ) | 10.623 ( 6.694 to 15.917 ) | 11.872 ( 7.28 to 17.6 ) | 0.48 (0.45 to 0.52) | 184 |
| Sweden | Hypertensive heart disease | Both | 411 ( 231 to 660 ) | 1723 ( 960 to 2662 ) | 2.497 ( 1.42 to 4.033 ) | 6.749 ( 3.778 to 10.487 ) | 4.55 (4.2 to 4.9) | 319.221 |
| Switzerland | Hypertensive heart disease | Both | 288 ( 161 to 451 ) | 987 ( 572 to 1516 ) | 2.6 ( 1.492 to 4 ) | 4.899 ( 2.845 to 7.542 ) | 3.39 (2.94 to 3.85) | 242.708 |
| Syrian Arab Republic | Hypertensive heart disease | Both | 509 ( 321 to 747 ) | 1167 ( 703 to 1728 ) | 10.474 ( 6.604 to 15.673 ) | 9.986 ( 5.955 to 14.895 ) | -0.33 (-0.41 to -0.26) | 129.273 |
| Taiwan (Province of China) | Hypertensive heart disease | Both | 1521 ( 934 to 2345 ) | 5624 ( 3429 to 8502 ) | 11.101 ( 6.693 to 16.922 ) | 13.072 ( 8.033 to 19.714 ) | 0.83 (0.41 to 1.25) | 269.757 |
| Tajikistan | Hypertensive heart disease | Both | 246 ( 146 to 364 ) | 468 ( 273 to 713 ) | 9.52 ( 5.522 to 14.205 ) | 9.159 ( 5.333 to 14.077 ) | -0.01 (-0.08 to 0.07) | 90.244 |
| Thailand | Hypertensive heart disease | Both | 1096 ( 684 to 1642 ) | 4927 ( 2971 to 7304 ) | 3.507 ( 2.159 to 5.302 ) | 4.6 ( 2.782 to 6.806 ) | 0.89 (0.78 to 1.01) | 349.544 |
| Timor-Leste | Hypertensive heart disease | Both | 24 ( 15 to 35 ) | 80 ( 48 to 121 ) | 10.531 ( 6.569 to 15.69 ) | 9.864 ( 6.075 to 14.847 ) | -0.34 (-0.38 to -0.29) | 233.333 |
| Togo | Hypertensive heart disease | Both | 150 ( 93 to 218 ) | 476 ( 299 to 700 ) | 14.27 ( 8.697 to 20.409 ) | 14.921 ( 9.266 to 22.039 ) | 0.14 (0.1 to 0.18) | 217.333 |
| Tokelau | Hypertensive heart disease | Both | 0 ( 0 to 0 ) | 0 ( 0 to 0 ) | 7.692 ( 4.679 to 11.551 ) | 6.72 ( 4.171 to 10.138 ) | -0.51 (-0.55 to -0.47) | #NUM! |
| Tonga | Hypertensive heart disease | Both | 2 ( 1 to 3 ) | 3 ( 2 to 5 ) | 4.416 ( 2.653 to 6.488 ) | 4.175 ( 2.549 to 6.36 ) | -0.25 (-0.3 to -0.2) | 50 |
| Trinidad and Tobago | Hypertensive heart disease | Both | 94 ( 58 to 139 ) | 208 ( 128 to 310 ) | 11.394 ( 7.037 to 16.898 ) | 11.118 ( 6.906 to 16.531 ) | 0.01 (-0.04 to 0.07) | 121.277 |
| Tunisia | Hypertensive heart disease | Both | 691 ( 439 to 1031 ) | 2022 ( 1272 to 2983 ) | 14.727 ( 9.392 to 21.7 ) | 15.939 ( 9.965 to 23.552 ) | 0.37 (0.31 to 0.43) | 192.619 |
| Turkey | Hypertensive heart disease | Both | 4086 ( 2530 to 6007 ) | 11696 ( 7014 to 17345 ) | 13.139 ( 8.015 to 19.127 ) | 13.155 ( 7.922 to 19.486 ) | 0.08 (0.02 to 0.13) | 186.246 |
| Turkmenistan | Hypertensive heart disease | Both | 59 ( 34 to 92 ) | 160 ( 93 to 245 ) | 3.496 ( 1.985 to 5.326 ) | 4.597 ( 2.606 to 7.226 ) | 1.19 (1.09 to 1.28) | 171.186 |
| Tuvalu | Hypertensive heart disease | Both | 0 ( 0 to 1 ) | 1 ( 0 to 1 ) | 7.393 ( 4.633 to 10.992 ) | 6.623 ( 3.957 to 10.021 ) | -0.41 (-0.44 to -0.38) | #NUM! |
| Uganda | Hypertensive heart disease | Both | 941 ( 588 to 1397 ) | 2322 ( 1427 to 3448 ) | 16.596 ( 10.344 to 24.237 ) | 17.685 ( 10.912 to 26.494 ) | 0.31 (0.26 to 0.37) | 146.759 |
| Ukraine | Hypertensive heart disease | Both | 1271 ( 710 to 2047 ) | 1686 ( 931 to 2748 ) | 1.796 ( 1.024 to 2.848 ) | 2.097 ( 1.171 to 3.387 ) | 0.48 (0.32 to 0.64) | 32.651 |
| United Arab Emirates | Hypertensive heart disease | Both | 55 ( 34 to 82 ) | 687 ( 418 to 1031 ) | 10.789 ( 6.81 to 15.702 ) | 13.859 ( 8.853 to 20.346 ) | 0.79 (0.78 to 0.81) | 1149.091 |
| United Kingdom | Hypertensive heart disease | Both | 1040 ( 602 to 1612 ) | 2659 ( 1509 to 4052 ) | 1.145 ( 0.676 to 1.745 ) | 2.006 ( 1.173 to 3.054 ) | 2.11 (2.02 to 2.2) | 155.673 |
| United Republic of Tanzania | Hypertensive heart disease | Both | 1547 ( 959 to 2314 ) | 3603 ( 2292 to 5322 ) | 16.438 ( 10.141 to 24.565 ) | 15.685 ( 9.888 to 23.202 ) | -0.01 (-0.09 to 0.07) | 132.902 |
| United States of America | Hypertensive heart disease | Both | 17268 ( 10254 to 25565 ) | 50167 ( 30514 to 73530 ) | 5.534 ( 3.355 to 8.11 ) | 8.948 ( 5.47 to 13 ) | 1.86 (1.73 to 1.99) | 190.52 |
| United States Virgin Islands | Hypertensive heart disease | Both | 7 ( 4 to 11 ) | 20 ( 12 to 30 ) | 9.517 ( 5.749 to 14.127 ) | 10.535 ( 6.286 to 15.906 ) | 0.33 (0.32 to 0.35) | 185.714 |
| Uruguay | Hypertensive heart disease | Both | 133 ( 78 to 206 ) | 300 ( 165 to 462 ) | 3.406 ( 2.003 to 5.225 ) | 4.96 ( 2.759 to 7.781 ) | 1.56 (1.49 to 1.63) | 125.564 |
| Uzbekistan | Hypertensive heart disease | Both | 463 ( 278 to 703 ) | 1167 ( 671 to 1827 ) | 4.107 ( 2.339 to 6.3 ) | 4.931 ( 2.688 to 7.745 ) | 0.76 (0.64 to 0.87) | 152.052 |
| Vanuatu | Hypertensive heart disease | Both | 3 ( 2 to 4 ) | 8 ( 5 to 12 ) | 5.282 ( 3.232 to 8.286 ) | 5.269 ( 3.126 to 7.986 ) | 0.01 (-0.01 to 0.04) | 166.667 |
| Venezuela (Bolivarian Republic of) | Hypertensive heart disease | Both | 949 ( 591 to 1411 ) | 2732 ( 1637 to 4152 ) | 10.497 ( 6.491 to 15.651 ) | 9.693 ( 5.799 to 14.665 ) | -0.3 (-0.33 to -0.28) | 187.882 |
| Viet Nam | Hypertensive heart disease | Both | 4489 ( 2817 to 6712 ) | 10197 ( 6393 to 15128 ) | 11.934 ( 7.439 to 17.924 ) | 11.447 ( 7.16 to 16.808 ) | -0.27 (-0.35 to -0.19) | 127.155 |
| Yemen | Hypertensive heart disease | Both | 608 ( 393 to 904 ) | 1899 ( 1226 to 2802 ) | 13.903 ( 8.87 to 20.318 ) | 14.115 ( 8.83 to 20.839 ) | 0.08 (0.07 to 0.1) | 212.336 |
| Zambia | Hypertensive heart disease | Both | 387 ( 240 to 570 ) | 999 ( 627 to 1485 ) | 16.193 ( 10.072 to 23.663 ) | 16.741 ( 10.534 to 25.017 ) | 0.09 (0.06 to 0.11) | 158.14 |
| Zimbabwe | Hypertensive heart disease | Both | 392 ( 236 to 596 ) | 624 ( 382 to 957 ) | 11.228 ( 6.894 to 17.287 ) | 10.982 ( 6.66 to 16.67 ) | -0.16 (-0.22 to -0.1) | 59.184 |

**Table S5 The Prevalence of SHF due to IHD in 1990 and 2021 across countries and territories.**

| Locations | cause | sex | 1990 Prevalence cases(95% UI) | 2021 Prevalence cases(95% UI) | 1990 ASPR(95% UI) | 2021 ASPR(95% UI) | 1990-2021 EAPC（95%CI） | 1990-2021 Prevalence cases changes |
| --- | --- | --- | --- | --- | --- | --- | --- | --- |
| Afghanistan | Ischemic heart disease | Both | 4107 ( 3130 to 5134 ) | 7231 ( 5801 to 8925 ) | 68.052 ( 52.186 to 86.153 ) | 69.991 ( 54.076 to 87.949 ) | 0.05 (0 to 0.1) | 76.065 |
| Albania | Ischemic heart disease | Both | 1601 ( 1193 to 2068 ) | 3753 ( 2739 to 5124 ) | 82.501 ( 60.401 to 108.386 ) | 86.303 ( 63.408 to 115.69 ) | 0.37 (0.29 to 0.45) | 134.416 |
| Algeria | Ischemic heart disease | Both | 11638 ( 9216 to 14599 ) | 32706 ( 25789 to 41085 ) | 100.607 ( 78.726 to 127.05 ) | 97.149 ( 75.907 to 122.624 ) | -0.32 (-0.38 to -0.25) | 181.028 |
| American Samoa | Ischemic heart disease | Both | 12 ( 9 to 15 ) | 31 ( 23 to 40 ) | 65.82 ( 49.738 to 87.805 ) | 75.267 ( 57.174 to 99.114 ) | 0.48 (0.43 to 0.54) | 158.333 |
| Andorra | Ischemic heart disease | Both | 32 ( 24 to 43 ) | 99 ( 73 to 131 ) | 59.543 ( 44.44 to 79.018 ) | 61.794 ( 45.324 to 81.028 ) | -0.18 (-0.24 to -0.11) | 209.375 |
| Angola | Ischemic heart disease | Both | 1308 ( 953 to 1732 ) | 4307 ( 3143 to 5698 ) | 45.933 ( 32.184 to 63.214 ) | 47.661 ( 34.202 to 65.236 ) | 0.08 (0.06 to 0.09) | 229.281 |
| Antigua and Barbuda | Ischemic heart disease | Both | 39 ( 30 to 51 ) | 49 ( 35 to 66 ) | 68.185 ( 52.729 to 87.362 ) | 50.226 ( 36.473 to 67.49 ) | -1.69 (-1.87 to -1.5) | 25.641 |
| Argentina | Ischemic heart disease | Both | 14321 ( 10596 to 18857 ) | 21518 ( 14833 to 29188 ) | 47.353 ( 35.21 to 62.995 ) | 37.799 ( 26.447 to 50.837 ) | -0.89 (-0.98 to -0.81) | 50.255 |
| Armenia | Ischemic heart disease | Both | 2732 ( 2027 to 3592 ) | 4981 ( 3826 to 6419 ) | 115.173 ( 84.95 to 153.841 ) | 115.421 ( 89.039 to 148.22 ) | 0.04 (-0.05 to 0.12) | 82.321 |
| Australia | Ischemic heart disease | Both | 25802 ( 19607 to 33800 ) | 50107 ( 38922 to 63180 ) | 131.108 ( 99.371 to 171.201 ) | 102.648 ( 80.498 to 129.434 ) | -1.43 (-1.67 to -1.18) | 94.198 |
| Austria | Ischemic heart disease | Both | 14030 ( 10530 to 17504 ) | 14727 ( 10665 to 19134 ) | 114.693 ( 88.316 to 142.19 ) | 72.231 ( 53.178 to 92.826 ) | -2.38 (-2.65 to -2.11) | 4.968 |
| Azerbaijan | Ischemic heart disease | Both | 4308 ( 3093 to 5751 ) | 7992 ( 5711 to 10751 ) | 98.434 ( 70.895 to 132.669 ) | 97.554 ( 69.276 to 132.645 ) | 0.02 (-0.05 to 0.08) | 85.515 |
| Bahamas | Ischemic heart disease | Both | 68 ( 52 to 89 ) | 161 ( 117 to 216 ) | 48.409 ( 36.558 to 63.662 ) | 45.039 ( 32.191 to 60.429 ) | -1.43 (-1.69 to -1.16) | 136.765 |
| Bahrain | Ischemic heart disease | Both | 245 ( 198 to 302 ) | 1092 ( 884 to 1364 ) | 120.086 ( 94.929 to 150.463 ) | 108.755 ( 86.538 to 138.094 ) | -0.45 (-0.5 to -0.41) | 345.714 |
| Bangladesh | Ischemic heart disease | Both | 21537 ( 17225 to 27047 ) | 80725 ( 62272 to 102607 ) | 46.919 ( 36.731 to 59.858 ) | 61.631 ( 47.768 to 79.091 ) | 0.98 (0.87 to 1.09) | 274.82 |
| Barbados | Ischemic heart disease | Both | 242 ( 177 to 327 ) | 326 ( 246 to 432 ) | 76.044 ( 56.647 to 101.453 ) | 62.093 ( 47.155 to 81.639 ) | -1.03 (-1.16 to -0.89) | 34.711 |
| Belarus | Ischemic heart disease | Both | 15019 ( 10781 to 20141 ) | 22312 ( 16387 to 29812 ) | 121.448 ( 87.595 to 162.798 ) | 136.086 ( 100.143 to 181.271 ) | 0.55 (0.49 to 0.61) | 48.558 |
| Belgium | Ischemic heart disease | Both | 10632 ( 7521 to 14406 ) | 12492 ( 9626 to 16284 ) | 68.542 ( 49.504 to 91.711 ) | 49.332 ( 38.615 to 63.546 ) | -0.83 (-0.98 to -0.68) | 17.494 |
| Belize | Ischemic heart disease | Both | 80 ( 62 to 104 ) | 161 ( 121 to 216 ) | 85.966 ( 65.424 to 112.478 ) | 60.844 ( 45.063 to 82.376 ) | -1.76 (-1.91 to -1.62) | 101.25 |
| Benin | Ischemic heart disease | Both | 643 ( 456 to 870 ) | 1854 ( 1366 to 2452 ) | 35.508 ( 24.618 to 48.871 ) | 41.223 ( 29.013 to 56.038 ) | 0.5 (0.48 to 0.53) | 188.336 |
| Bermuda | Ischemic heart disease | Both | 73 ( 56 to 96 ) | 130 ( 95 to 172 ) | 123.136 ( 93.953 to 160.074 ) | 88.893 ( 66.499 to 117.557 ) | -1.18 (-1.26 to -1.11) | 78.082 |
| Bhutan | Ischemic heart disease | Both | 104 ( 84 to 129 ) | 372 ( 292 to 464 ) | 46.327 ( 36.02 to 58.94 ) | 62.785 ( 48.715 to 78.972 ) | 1.13 (1.08 to 1.18) | 257.692 |
| Bolivia (Plurinational State of) | Ischemic heart disease | Both | 1975 ( 1546 to 2522 ) | 5430 ( 4201 to 6993 ) | 67.989 ( 52.691 to 88.952 ) | 65.2 ( 50.03 to 85.038 ) | -0.17 (-0.24 to -0.09) | 174.937 |
| Bosnia and Herzegovina | Ischemic heart disease | Both | 2860 ( 2096 to 3729 ) | 4478 ( 3020 to 6222 ) | 80.799 ( 57.388 to 107.902 ) | 71.218 ( 49.356 to 97.922 ) | -0.6 (-0.67 to -0.53) | 56.573 |
| Botswana | Ischemic heart disease | Both | 205 ( 144 to 279 ) | 656 ( 477 to 880 ) | 47.795 ( 33.757 to 66.375 ) | 55.226 ( 40.008 to 75.679 ) | 0.42 (0.4 to 0.44) | 220 |
| Brazil | Ischemic heart disease | Both | 46059 ( 36804 to 56768 ) | 136971 ( 106210 to 173829 ) | 58.124 ( 45.565 to 72.911 ) | 56.018 ( 43.527 to 71.355 ) | -0.01 (-0.06 to 0.04) | 197.382 |
| Brunei Darussalam | Ischemic heart disease | Both | 39 ( 29 to 51 ) | 109 ( 78 to 147 ) | 33.033 ( 21.355 to 45.54 ) | 31.42 ( 20.682 to 43.779 ) | -0.33 (-0.38 to -0.29) | 179.487 |
| Bulgaria | Ischemic heart disease | Both | 8773 ( 6234 to 11844 ) | 7951 ( 5457 to 11186 ) | 81.796 ( 60.255 to 108.71 ) | 53.811 ( 38.254 to 74.037 ) | -2.54 (-2.86 to -2.23) | -9.37 |
| Burkina Faso | Ischemic heart disease | Both | 1125 ( 784 to 1568 ) | 2780 ( 1998 to 3732 ) | 31.884 ( 22.065 to 44.837 ) | 34.937 ( 24.723 to 47.287 ) | 0.29 (0.27 to 0.31) | 147.111 |
| Burundi | Ischemic heart disease | Both | 792 ( 556 to 1112 ) | 1757 ( 1270 to 2335 ) | 40.393 ( 28.071 to 56.873 ) | 45.045 ( 31.221 to 61.872 ) | 0.36 (0.34 to 0.37) | 121.843 |
| Cabo Verde | Ischemic heart disease | Both | 96 ( 66 to 134 ) | 200 ( 147 to 266 ) | 40.533 ( 28.204 to 56.493 ) | 47.565 ( 34.114 to 64.946 ) | 0.54 (0.48 to 0.6) | 108.333 |
| Cambodia | Ischemic heart disease | Both | 1469 ( 1142 to 1881 ) | 4964 ( 3865 to 6338 ) | 39.799 ( 29.826 to 51.79 ) | 48.44 ( 37.5 to 63.026 ) | 0.69 (0.68 to 0.71) | 237.917 |
| Cameroon | Ischemic heart disease | Both | 1023 ( 739 to 1399 ) | 3780 ( 2720 to 5155 ) | 28.349 ( 19.695 to 39.384 ) | 35.988 ( 24.894 to 49.83 ) | 0.82 (0.71 to 0.94) | 269.501 |
| Canada | Ischemic heart disease | Both | 48728 ( 37176 to 62658 ) | 92987 ( 69183 to 123418 ) | 148.155 ( 113.695 to 187.663 ) | 122.083 ( 92.486 to 159.986 ) | -0.72 (-0.93 to -0.5) | 90.829 |
| Central African Republic | Ischemic heart disease | Both | 327 ( 232 to 449 ) | 698 ( 506 to 920 ) | 40.786 ( 28.508 to 57.421 ) | 44.723 ( 31.778 to 60.657 ) | 0.26 (0.23 to 0.3) | 113.456 |
| Chad | Ischemic heart disease | Both | 921 ( 652 to 1262 ) | 2024 ( 1474 to 2701 ) | 36.513 ( 25.196 to 49.775 ) | 41.919 ( 29.514 to 57.956 ) | 0.45 (0.44 to 0.46) | 119.761 |
| Chile | Ischemic heart disease | Both | 4880 ( 3631 to 6470 ) | 11228 ( 8155 to 15065 ) | 52.339 ( 38.782 to 70.466 ) | 43.858 ( 32.167 to 58.56 ) | -0.85 (-0.97 to -0.74) | 130.082 |
| China | Ischemic heart disease | Both | 330244 ( 254502 to 425143 ) | 1494576 ( 1136074 to 1925406 ) | 47.748 ( 36.972 to 61.552 ) | 75.003 ( 57.285 to 95.449 ) | 1.92 (1.73 to 2.12) | 352.567 |
| Colombia | Ischemic heart disease | Both | 12704 ( 9948 to 15825 ) | 51954 ( 39595 to 67883 ) | 76.335 ( 58.927 to 98.414 ) | 92.977 ( 70.979 to 121.798 ) | 0.82 (0.78 to 0.86) | 308.958 |
| Comoros | Ischemic heart disease | Both | 72 ( 50 to 97 ) | 213 ( 153 to 290 ) | 45.17 ( 32.033 to 62.127 ) | 50.537 ( 35.924 to 68.772 ) | 0.35 (0.3 to 0.41) | 195.833 |
| Congo | Ischemic heart disease | Both | 364 ( 257 to 495 ) | 1073 ( 785 to 1406 ) | 44.895 ( 31.304 to 61.837 ) | 51.669 ( 36.626 to 68.836 ) | 0.43 (0.42 to 0.44) | 194.78 |
| Cook Islands | Ischemic heart disease | Both | 4 ( 3 to 5 ) | 13 ( 10 to 18 ) | 38.435 ( 28.7 to 50.506 ) | 52.828 ( 38.762 to 69.928 ) | 0.74 (0.63 to 0.85) | 225 |
| Costa Rica | Ischemic heart disease | Both | 1530 ( 1190 to 1936 ) | 4247 ( 3244 to 5538 ) | 89.22 ( 69.614 to 112.804 ) | 77.154 ( 58.622 to 101.652 ) | -0.43 (-0.51 to -0.34) | 177.582 |
| Coted'Ivoire | Ischemic heart disease | Both | 1146 ( 847 to 1510 ) | 4135 ( 3057 to 5495 ) | 37.777 ( 26.351 to 51.971 ) | 45.43 ( 32.712 to 62.051 ) | 0.6 (0.58 to 0.62) | 260.82 |
| Croatia | Ischemic heart disease | Both | 4014 ( 2855 to 5345 ) | 4885 ( 3341 to 6824 ) | 72.447 ( 51.137 to 97.293 ) | 51.697 ( 36.238 to 71.04 ) | -1.79 (-2.22 to -1.36) | 21.699 |
| Cuba | Ischemic heart disease | Both | 14362 ( 10905 to 18783 ) | 19050 ( 14453 to 25126 ) | 141.5 ( 107.585 to 183.726 ) | 94.245 ( 71.998 to 122.555 ) | -1.5 (-1.58 to -1.43) | 32.642 |
| Cyprus | Ischemic heart disease | Both | 388 ( 268 to 540 ) | 1184 ( 853 to 1625 ) | 51.193 ( 35.556 to 70.035 ) | 57.284 ( 41.801 to 76.823 ) | -0.48 (-0.78 to -0.17) | 205.155 |
| Czechia | Ischemic heart disease | Both | 19124 ( 13493 to 25976 ) | 24410 ( 17152 to 32968 ) | 141.443 ( 103.526 to 189.332 ) | 107.748 ( 77.849 to 143.77 ) | -0.17 (-0.52 to 0.18) | 27.641 |
| Democratic People's Republic of Korea | Ischemic heart disease | Both | 6477 ( 5084 to 8368 ) | 18545 ( 13947 to 24344 ) | 48.515 ( 37.351 to 63.057 ) | 61.729 ( 47.389 to 80.121 ) | 0.83 (0.79 to 0.88) | 186.321 |
| Democratic Republic of the Congo | Ischemic heart disease | Both | 5087 ( 3616 to 6937 ) | 13134 ( 9661 to 17612 ) | 44.734 ( 31.187 to 61.343 ) | 45.877 ( 33.325 to 63.404 ) | 0 (-0.03 to 0.02) | 158.188 |
| Denmark | Ischemic heart disease | Both | 6331 ( 4561 to 8529 ) | 7730 ( 5408 to 10730 ) | 75.892 ( 55.758 to 100.426 ) | 61.526 ( 44.115 to 84.142 ) | -0.91 (-1.09 to -0.72) | 22.098 |
| Djibouti | Ischemic heart disease | Both | 48 ( 35 to 63 ) | 276 ( 201 to 366 ) | 46.629 ( 33.51 to 63.709 ) | 55.525 ( 39.279 to 75.615 ) | 0.61 (0.57 to 0.64) | 475 |
| Dominica | Ischemic heart disease | Both | 29 ( 21 to 39 ) | 34 ( 25 to 47 ) | 47.994 ( 36.248 to 64.083 ) | 43.97 ( 32.141 to 59.401 ) | -0.93 (-1.11 to -0.75) | 17.241 |
| Dominican Republic | Ischemic heart disease | Both | 3084 ( 2393 to 3875 ) | 8896 ( 6732 to 11711 ) | 90.02 ( 68.818 to 115.142 ) | 91.517 ( 68.84 to 121.266 ) | 0.2 (0.13 to 0.26) | 188.457 |
| Ecuador | Ischemic heart disease | Both | 3053 ( 2370 to 3833 ) | 11093 ( 8301 to 14431 ) | 59.479 ( 45.658 to 76.127 ) | 69.283 ( 51.8 to 89.919 ) | -0.01 (-0.28 to 0.27) | 263.348 |
| Egypt | Ischemic heart disease | Both | 21226 ( 17070 to 26422 ) | 59626 ( 47508 to 74713 ) | 83.762 ( 65.384 to 105.715 ) | 103.944 ( 81.274 to 132.234 ) | 0.77 (0.7 to 0.85) | 180.91 |
| El Salvador | Ischemic heart disease | Both | 2921 ( 2305 to 3656 ) | 6618 ( 5103 to 8650 ) | 99.127 ( 77.55 to 125.796 ) | 100.579 ( 76.956 to 131.291 ) | -0.02 (-0.05 to 0) | 126.566 |
| Equatorial Guinea | Ischemic heart disease | Both | 64 ( 46 to 86 ) | 221 ( 162 to 292 ) | 43.901 ( 30.404 to 60.184 ) | 52.442 ( 37.49 to 72.632 ) | 0.63 (0.6 to 0.66) | 245.312 |
| Eritrea | Ischemic heart disease | Both | 289 ( 210 to 387 ) | 895 ( 646 to 1192 ) | 38.031 ( 26.457 to 52.309 ) | 42.382 ( 30.099 to 58.309 ) | 0.37 (0.34 to 0.4) | 209.689 |
| Estonia | Ischemic heart disease | Both | 2224 ( 1551 to 3062 ) | 1874 ( 1234 to 2701 ) | 110.819 ( 77.782 to 151.087 ) | 61.121 ( 41.146 to 86.411 ) | -3.32 (-3.7 to -2.93) | -15.737 |
| Eswatini | Ischemic heart disease | Both | 100 ( 72 to 135 ) | 218 ( 157 to 295 ) | 44.387 ( 31.482 to 60.93 ) | 50.342 ( 35.424 to 69.086 ) | 0.43 (0.4 to 0.46) | 118 |
| Ethiopia | Ischemic heart disease | Both | 4697 ( 3479 to 6339 ) | 13080 ( 9659 to 17566 ) | 31.513 ( 22.649 to 43.234 ) | 34.757 ( 25.164 to 47.829 ) | 0.29 (0.28 to 0.3) | 178.476 |
| Fiji | Ischemic heart disease | Both | 219 ( 171 to 281 ) | 512 ( 396 to 662 ) | 76.295 ( 58.934 to 98.622 ) | 82.669 ( 63.088 to 108.867 ) | 0.28 (0.26 to 0.3) | 133.79 |
| Finland | Ischemic heart disease | Both | 6908 ( 4565 to 9320 ) | 12087 ( 8426 to 16712 ) | 96.493 ( 65.732 to 127.735 ) | 84.182 ( 60.755 to 112.712 ) | -0.61 (-1 to -0.21) | 74.971 |
| France | Ischemic heart disease | Both | 111851 ( 83717 to 147254 ) | 197996 ( 145995 to 261572 ) | 129.199 ( 98.268 to 169.695 ) | 123.35 ( 92.364 to 160.95 ) | -0.53 (-0.72 to -0.33) | 77.018 |
| Gabon | Ischemic heart disease | Both | 210 ( 145 to 289 ) | 419 ( 304 to 568 ) | 42.405 ( 29.643 to 58.389 ) | 49.256 ( 35.362 to 67.708 ) | 0.48 (0.47 to 0.49) | 99.524 |
| Gambia | Ischemic heart disease | Both | 115 ( 83 to 153 ) | 399 ( 291 to 535 ) | 39.229 ( 27.184 to 53.241 ) | 46.242 ( 32.805 to 63.028 ) | 0.47 (0.43 to 0.5) | 246.957 |
| Georgia | Ischemic heart disease | Both | 6710 ( 4723 to 9164 ) | 3276 ( 1698 to 4914 ) | 115.806 ( 81.745 to 156.89 ) | 53.115 ( 29.122 to 78.536 ) | -3.77 (-4.15 to -3.39) | -51.177 |
| Germany | Ischemic heart disease | Both | 110922 ( 79843 to 149496 ) | 158869 ( 115070 to 214163 ) | 85.787 ( 63.142 to 114.519 ) | 74.008 ( 54.797 to 98.547 ) | 0.06 (-0.2 to 0.32) | 43.226 |
| Ghana | Ischemic heart disease | Both | 2295 ( 1682 to 3107 ) | 7431 ( 5467 to 9992 ) | 46.838 ( 33.51 to 63.35 ) | 54.048 ( 38.82 to 74.585 ) | 0.49 (0.47 to 0.51) | 223.791 |
| Greece | Ischemic heart disease | Both | 10008 ( 7443 to 13171 ) | 10533 ( 7660 to 14039 ) | 67.541 ( 50.849 to 88.769 ) | 41.126 ( 31.34 to 53.843 ) | -2.56 (-2.85 to -2.26) | 5.246 |
| Greenland | Ischemic heart disease | Both | 22 ( 15 to 29 ) | 41 ( 27 to 57 ) | 78.162 ( 50.235 to 109.895 ) | 70.085 ( 44.864 to 99.447 ) | -0.53 (-0.62 to -0.43) | 86.364 |
| Grenada | Ischemic heart disease | Both | 54 ( 40 to 73 ) | 52 ( 38 to 69 ) | 68.152 ( 51.278 to 91.052 ) | 51.321 ( 37.95 to 68.153 ) | -1.38 (-1.49 to -1.27) | -3.704 |
| Guam | Ischemic heart disease | Both | 39 ( 30 to 50 ) | 211 ( 162 to 278 ) | 65.158 ( 49.077 to 86.915 ) | 97.897 ( 74.735 to 129.152 ) | 1.44 (1.27 to 1.6) | 441.026 |
| Guatemala | Ischemic heart disease | Both | 2794 ( 2109 to 3512 ) | 9586 ( 7447 to 12411 ) | 92.346 ( 70.746 to 120.167 ) | 91.498 ( 69.892 to 119.711 ) | -0.07 (-0.12 to -0.03) | 243.092 |
| Guinea | Ischemic heart disease | Both | 957 ( 683 to 1323 ) | 1957 ( 1429 to 2627 ) | 32.911 ( 22.925 to 45.709 ) | 39.595 ( 27.846 to 54.642 ) | 0.61 (0.59 to 0.63) | 104.493 |
| Guinea-Bissau | Ischemic heart disease | Both | 108 ( 75 to 148 ) | 229 ( 168 to 309 ) | 34.695 ( 24.249 to 47.744 ) | 40.495 ( 28.562 to 56.163 ) | 0.54 (0.52 to 0.56) | 112.037 |
| Guyana | Ischemic heart disease | Both | 184 ( 142 to 237 ) | 261 ( 193 to 352 ) | 54.431 ( 41.417 to 71.188 ) | 48.169 ( 35.292 to 65.792 ) | -0.55 (-0.71 to -0.39) | 41.848 |
| Haiti | Ischemic heart disease | Both | 1727 ( 1299 to 2228 ) | 4218 ( 3237 to 5320 ) | 68.008 ( 51.091 to 88.852 ) | 74.078 ( 56.097 to 97.502 ) | 0.26 (0.26 to 0.27) | 144.239 |
| Honduras | Ischemic heart disease | Both | 1096 ( 865 to 1385 ) | 3777 ( 2876 to 4850 ) | 59.456 ( 46.093 to 77.142 ) | 66.488 ( 50.043 to 86.968 ) | 0.42 (0.38 to 0.46) | 244.617 |
| Hungary | Ischemic heart disease | Both | 10179 ( 6831 to 13996 ) | 12547 ( 8344 to 17808 ) | 72.058 ( 49.37 to 98.681 ) | 59.517 ( 40.435 to 83.383 ) | -0.74 (-0.84 to -0.64) | 23.264 |
| Iceland | Ischemic heart disease | Both | 144 ( 94 to 196 ) | 266 ( 189 to 368 ) | 49.081 ( 32.769 to 66.471 ) | 44.034 ( 31.69 to 59.938 ) | -0.86 (-1.15 to -0.57) | 84.722 |
| India | Ischemic heart disease | Both | 234457 ( 190749 to 283820 ) | 775717 ( 615909 to 955284 ) | 57.815 ( 46.333 to 71.373 ) | 70.426 ( 55.645 to 87.233 ) | 0.8 (0.75 to 0.84) | 230.857 |
| Indonesia | Ischemic heart disease | Both | 39103 ( 31583 to 47370 ) | 108417 ( 83758 to 135824 ) | 46.046 ( 36.183 to 57.857 ) | 53.894 ( 41.616 to 68.077 ) | 0.68 (0.63 to 0.72) | 177.26 |
| Iran (Islamic Republic of) | Ischemic heart disease | Both | 27335 ( 22199 to 32670 ) | 82268 ( 66067 to 99099 ) | 112.958 ( 92.371 to 137.302 ) | 109.243 ( 86.498 to 133.828 ) | -0.26 (-0.33 to -0.19) | 200.962 |
| Iraq | Ischemic heart disease | Both | 7805 ( 6185 to 9942 ) | 24445 ( 19423 to 30418 ) | 97.779 ( 76.016 to 127.277 ) | 108.833 ( 85.528 to 138.62 ) | 0.39 (0.36 to 0.42) | 213.197 |
| Ireland | Ischemic heart disease | Both | 3261 ( 2299 to 4451 ) | 6282 ( 4693 to 8360 ) | 80.574 ( 57.631 to 108.06 ) | 76.435 ( 57.974 to 101.045 ) | -0.31 (-0.36 to -0.25) | 92.64 |
| Israel | Ischemic heart disease | Both | 3330 ( 2359 to 4541 ) | 7999 ( 5842 to 10659 ) | 70.596 ( 50.275 to 95.295 ) | 60.791 ( 44.554 to 80.72 ) | -0.97 (-1.14 to -0.8) | 140.21 |
| Italy | Ischemic heart disease | Both | 50678 ( 35454 to 66860 ) | 97870 ( 74242 to 126487 ) | 57.895 ( 41.373 to 74.372 ) | 62.824 ( 49.131 to 79.899 ) | 0.35 (-0.23 to 0.94) | 93.121 |
| Jamaica | Ischemic heart disease | Both | 795 ( 613 to 1050 ) | 1585 ( 1148 to 2188 ) | 42.51 ( 33.069 to 55.786 ) | 48.631 ( 34.912 to 67.681 ) | -0.28 (-0.44 to -0.12) | 99.371 |
| Japan | Ischemic heart disease | Both | 62103 ( 44321 to 81392 ) | 148398 ( 108562 to 192251 ) | 39.215 ( 28.057 to 51.564 ) | 39.278 ( 30.663 to 48.738 ) | -0.05 (-0.17 to 0.07) | 138.955 |
| Jordan | Ischemic heart disease | Both | 1274 ( 1024 to 1556 ) | 6517 ( 5155 to 8178 ) | 90.299 ( 70.296 to 114.841 ) | 86.607 ( 67.322 to 111.478 ) | -0.45 (-0.53 to -0.37) | 411.538 |
| Kazakhstan | Ischemic heart disease | Both | 12208 ( 8675 to 16410 ) | 13529 ( 9555 to 18499 ) | 109.802 ( 77.515 to 148.776 ) | 90.609 ( 63.629 to 124.345 ) | -1.07 (-1.29 to -0.85) | 10.821 |
| Kenya | Ischemic heart disease | Both | 3217 ( 2390 to 4270 ) | 9306 ( 6985 to 12260 ) | 46.332 ( 34.087 to 62.026 ) | 49.397 ( 36.364 to 66.462 ) | -0.19 (-0.39 to 0.01) | 189.276 |
| Kiribati | Ischemic heart disease | Both | 18 ( 13 to 23 ) | 38 ( 29 to 48 ) | 60.634 ( 45.215 to 79.528 ) | 68.441 ( 52.63 to 89.249 ) | 0.39 (0.38 to 0.41) | 111.111 |
| Kuwait | Ischemic heart disease | Both | 858 ( 690 to 1055 ) | 4271 ( 3382 to 5292 ) | 118.101 ( 92.889 to 152.405 ) | 129.515 ( 99.981 to 163.323 ) | 0.4 (0.36 to 0.44) | 397.786 |
| Kyrgyzstan | Ischemic heart disease | Both | 2828 ( 2035 to 3864 ) | 4067 ( 2968 to 5415 ) | 104.22 ( 74.329 to 144.061 ) | 105.386 ( 75.164 to 143.263 ) | 0.22 (0.15 to 0.29) | 43.812 |
| Lao People's Democratic Republic | Ischemic heart disease | Both | 876 ( 677 to 1116 ) | 2289 ( 1740 to 2946 ) | 51.799 ( 39.972 to 68.224 ) | 58.622 ( 44.329 to 77.767 ) | 0.52 (0.48 to 0.56) | 161.301 |
| Latvia | Ischemic heart disease | Both | 4228 ( 2937 to 5916 ) | 3959 ( 2715 to 5481 ) | 119.108 ( 83.332 to 164.472 ) | 86.514 ( 59.991 to 118.878 ) | -1.6 (-1.79 to -1.41) | -6.362 |
| Lebanon | Ischemic heart disease | Both | 2282 ( 1800 to 2857 ) | 7249 ( 5741 to 9151 ) | 112.351 ( 87.56 to 141.728 ) | 116.094 ( 92.042 to 145.69 ) | 0.14 (0.11 to 0.16) | 217.66 |
| Lesotho | Ischemic heart disease | Both | 267 ( 191 to 370 ) | 364 ( 255 to 502 ) | 36.469 ( 25.787 to 50.635 ) | 42.239 ( 30.148 to 58.08 ) | 0.5 (0.48 to 0.51) | 36.33 |
| Liberia | Ischemic heart disease | Both | 393 ( 277 to 542 ) | 825 ( 607 to 1078 ) | 39.173 ( 27.198 to 53.979 ) | 46.308 ( 32.911 to 64.001 ) | 0.58 (0.54 to 0.61) | 109.924 |
| Libya | Ischemic heart disease | Both | 1997 ( 1623 to 2436 ) | 5380 ( 4320 to 6824 ) | 102.555 ( 80.576 to 126.687 ) | 103.813 ( 80.813 to 133.41 ) | 0.01 (-0.09 to 0.1) | 169.404 |
| Lithuania | Ischemic heart disease | Both | 5750 ( 4133 to 7872 ) | 6918 ( 4839 to 9622 ) | 128.75 ( 93.361 to 175.478 ) | 106.151 ( 74.956 to 144.019 ) | -0.74 (-0.83 to -0.64) | 20.313 |
| Luxembourg | Ischemic heart disease | Both | 384 ( 245 to 539 ) | 813 ( 585 to 1135 ) | 71.872 ( 47.772 to 98.012 ) | 73.257 ( 53.274 to 101.512 ) | -0.73 (-1.18 to -0.28) | 111.719 |
| Madagascar | Ischemic heart disease | Both | 1869 ( 1349 to 2586 ) | 4281 ( 3123 to 5730 ) | 45.027 ( 32.287 to 63.452 ) | 50.177 ( 35.818 to 69.148 ) | 0.34 (0.31 to 0.37) | 129.053 |
| Malawi | Ischemic heart disease | Both | 1389 ( 994 to 1874 ) | 3191 ( 2319 to 4274 ) | 45.909 ( 32.805 to 63.143 ) | 51.91 ( 36.877 to 71.141 ) | 0.33 (0.3 to 0.37) | 129.734 |
| Malaysia | Ischemic heart disease | Both | 7720 ( 6009 to 9862 ) | 26374 ( 20221 to 34124 ) | 90.855 ( 69.187 to 117.714 ) | 102.676 ( 78.981 to 133.195 ) | 0.62 (0.55 to 0.68) | 241.632 |
| Maldives | Ischemic heart disease | Both | 53 ( 42 to 67 ) | 247 ( 195 to 307 ) | 70.845 ( 54.601 to 92.402 ) | 76.299 ( 58.461 to 97.816 ) | 0.28 (0.22 to 0.33) | 366.038 |
| Mali | Ischemic heart disease | Both | 1074 ( 765 to 1456 ) | 2942 ( 2153 to 3898 ) | 34.303 ( 23.863 to 46.625 ) | 40.582 ( 28.684 to 55.284 ) | 0.55 (0.54 to 0.57) | 173.929 |
| Malta | Ischemic heart disease | Both | 334 ( 252 to 436 ) | 854 ( 638 to 1140 ) | 82.012 ( 61.857 to 106.937 ) | 80.926 ( 61.599 to 105.847 ) | 0.4 (0.2 to 0.6) | 155.689 |
| Marshall Islands | Ischemic heart disease | Both | 8 ( 6 to 10 ) | 18 ( 14 to 23 ) | 59.315 ( 45.373 to 78.454 ) | 71.9 ( 55.045 to 95.139 ) | 0.65 (0.63 to 0.68) | 125 |
| Mauritania | Ischemic heart disease | Both | 368 ( 262 to 496 ) | 956 ( 696 to 1295 ) | 41.759 ( 29.178 to 56.408 ) | 49.846 ( 36.061 to 68.336 ) | 0.59 (0.58 to 0.6) | 159.783 |
| Mauritius | Ischemic heart disease | Both | 444 ( 344 to 570 ) | 961 ( 720 to 1266 ) | 69.565 ( 54.075 to 90.966 ) | 56.109 ( 42.496 to 73.311 ) | -0.81 (-0.97 to -0.65) | 116.441 |
| Mexico | Ischemic heart disease | Both | 32437 ( 26091 to 39412 ) | 104754 ( 82718 to 130028 ) | 82.09 ( 65.324 to 101.979 ) | 87.71 ( 68.809 to 109.821 ) | 0.22 (0.15 to 0.28) | 222.946 |
| Micronesia (Federated States of) | Ischemic heart disease | Both | 25 ( 19 to 32 ) | 39 ( 30 to 50 ) | 57.832 ( 43.845 to 77.337 ) | 69.042 ( 52.426 to 91.494 ) | 0.63 (0.61 to 0.66) | 56 |
| Monaco | Ischemic heart disease | Both | 49 ( 36 to 65 ) | 65 ( 47 to 86 ) | 63.382 ( 47.867 to 83.262 ) | 59.084 ( 43.871 to 78.554 ) | -0.53 (-0.6 to -0.45) | 32.653 |
| Mongolia | Ischemic heart disease | Both | 805 ( 527 to 1116 ) | 1435 ( 977 to 1955 ) | 87.12 ( 57.479 to 121.71 ) | 83.556 ( 54.768 to 116.018 ) | -0.06 (-0.13 to 0.01) | 78.261 |
| Montenegro | Ischemic heart disease | Both | 471 ( 352 to 624 ) | 757 ( 549 to 1013 ) | 80.453 ( 59.585 to 107.234 ) | 79.6 ( 59.377 to 106.484 ) | 0.01 (-0.02 to 0.03) | 60.722 |
| Morocco | Ischemic heart disease | Both | 14231 ( 11294 to 17713 ) | 32531 ( 25348 to 41148 ) | 100.493 ( 78.229 to 127.902 ) | 100.688 ( 77.608 to 128.932 ) | -0.09 (-0.13 to -0.05) | 128.593 |
| Mozambique | Ischemic heart disease | Both | 2018 ( 1441 to 2737 ) | 4105 ( 2896 to 5640 ) | 42.319 ( 29.494 to 57.801 ) | 45.211 ( 32.098 to 62.595 ) | 0.11 (0.07 to 0.15) | 103.419 |
| Myanmar | Ischemic heart disease | Both | 9347 ( 7301 to 12039 ) | 23285 ( 18006 to 30328 ) | 49.195 ( 38.201 to 64.167 ) | 55.487 ( 42.617 to 72.54 ) | 0.51 (0.46 to 0.55) | 149.117 |
| Namibia | Ischemic heart disease | Both | 254 ( 183 to 349 ) | 566 ( 406 to 763 ) | 50.178 ( 36.043 to 68.322 ) | 50.209 ( 35.928 to 68.357 ) | -0.04 (-0.06 to -0.01) | 122.835 |
| Nauru | Ischemic heart disease | Both | 3 ( 2 to 3 ) | 4 ( 3 to 5 ) | 73.363 ( 55.322 to 97.421 ) | 83.454 ( 63.144 to 109.4 ) | 0.4 (0.39 to 0.41) | 33.333 |
| Nepal | Ischemic heart disease | Both | 4142 ( 3265 to 5183 ) | 12968 ( 10031 to 16412 ) | 49.581 ( 38.585 to 63.317 ) | 60.617 ( 46.537 to 77.675 ) | 0.8 (0.7 to 0.91) | 213.085 |
| Netherlands | Ischemic heart disease | Both | 15819 ( 12145 to 20412 ) | 24204 ( 17795 to 32174 ) | 77.235 ( 59.707 to 98.318 ) | 64.913 ( 48.387 to 85.6 ) | -1.05 (-1.23 to -0.86) | 53.006 |
| New Zealand | Ischemic heart disease | Both | 3784 ( 2775 to 4984 ) | 7656 ( 6010 to 9775 ) | 95.538 ( 70.919 to 123.264 ) | 86.087 ( 67.888 to 109.076 ) | -0.94 (-1.1 to -0.78) | 102.326 |
| Nicaragua | Ischemic heart disease | Both | 1127 ( 886 to 1442 ) | 3845 ( 2970 to 4914 ) | 77.027 ( 60.012 to 100.544 ) | 83.258 ( 63.9 to 109.181 ) | 0.27 (0.25 to 0.29) | 241.171 |
| Niger | Ischemic heart disease | Both | 731 ( 526 to 991 ) | 2538 ( 1844 to 3422 ) | 33.246 ( 23.241 to 46.538 ) | 37.972 ( 26.688 to 51.687 ) | 0.4 (0.36 to 0.44) | 247.196 |
| Nigeria | Ischemic heart disease | Both | 9990 ( 7295 to 13323 ) | 26080 ( 19632 to 34031 ) | 26.103 ( 18.832 to 35.522 ) | 33.913 ( 24.745 to 46.087 ) | 0.94 (0.9 to 0.98) | 161.061 |
| Niue | Ischemic heart disease | Both | 2 ( 1 to 2 ) | 2 ( 1 to 2 ) | 71.927 ( 55.269 to 93.458 ) | 84.473 ( 64.604 to 110.503 ) | 0.51 (0.48 to 0.55) | 0 |
| North Macedonia | Ischemic heart disease | Both | 1132 ( 841 to 1471 ) | 1797 ( 1287 to 2472 ) | 65.015 ( 48.065 to 85.304 ) | 59.031 ( 43.169 to 79.22 ) | -0.44 (-0.49 to -0.4) | 58.746 |
| Northern Mariana Islands | Ischemic heart disease | Both | 11 ( 9 to 14 ) | 38 ( 28 to 49 ) | 79.101 ( 59.798 to 102.764 ) | 90.926 ( 68.426 to 119.966 ) | 0.47 (0.45 to 0.49) | 245.455 |
| Norway | Ischemic heart disease | Both | 4786 ( 2714 to 6761 ) | 7388 ( 5316 to 9753 ) | 68.758 ( 42.959 to 93.563 ) | 69.248 ( 51.203 to 89.553 ) | -0.21 (-0.38 to -0.05) | 54.367 |
| Oman | Ischemic heart disease | Both | 693 ( 565 to 840 ) | 2076 ( 1666 to 2651 ) | 94.313 ( 74.47 to 117.749 ) | 90.029 ( 69.725 to 115.368 ) | -0.37 (-0.51 to -0.23) | 199.567 |
| Pakistan | Ischemic heart disease | Both | 30769 ( 24761 to 37154 ) | 75740 ( 60859 to 91985 ) | 58.935 ( 47.304 to 72.147 ) | 67.525 ( 52.546 to 84.301 ) | 0.62 (0.56 to 0.69) | 146.157 |
| Palau | Ischemic heart disease | Both | 7 ( 6 to 10 ) | 17 ( 13 to 22 ) | 88.035 ( 66.037 to 115.232 ) | 97.209 ( 74.473 to 127.562 ) | 0.31 (0.28 to 0.33) | 142.857 |
| Palestine | Ischemic heart disease | Both | 817 ( 652 to 1027 ) | 2551 ( 2037 to 3128 ) | 93.323 ( 72.654 to 118.58 ) | 98.515 ( 77.358 to 124.826 ) | 0.12 (0.1 to 0.14) | 212.24 |
| Panama | Ischemic heart disease | Both | 1384 ( 1072 to 1836 ) | 3590 ( 2760 to 4693 ) | 97.454 ( 74.537 to 130.338 ) | 80.009 ( 61.65 to 104.558 ) | -0.71 (-0.76 to -0.66) | 159.393 |
| Papua New Guinea | Ischemic heart disease | Both | 698 ( 544 to 894 ) | 2426 ( 1923 to 3068 ) | 51.02 ( 39.139 to 66.298 ) | 61.634 ( 47.626 to 80.896 ) | 0.63 (0.57 to 0.69) | 247.564 |
| Paraguay | Ischemic heart disease | Both | 1539 ( 1192 to 1950 ) | 3856 ( 2978 to 4998 ) | 71.92 ( 55.203 to 93.02 ) | 68.581 ( 52.47 to 89.85 ) | -0.17 (-0.25 to -0.1) | 150.552 |
| Peru | Ischemic heart disease | Both | 9535 ( 7560 to 12030 ) | 29273 ( 22620 to 37707 ) | 82.16 ( 64.245 to 105.795 ) | 87.021 ( 67.21 to 113.124 ) | 0.04 (-0.06 to 0.14) | 207.006 |
| Philippines | Ischemic heart disease | Both | 14875 ( 11975 to 18206 ) | 41998 ( 32920 to 52501 ) | 57.786 ( 45.5 to 72.282 ) | 58.883 ( 46.178 to 74.466 ) | -0.05 (-0.1 to -0.01) | 182.339 |
| Poland | Ischemic heart disease | Both | 55063 ( 43465 to 68503 ) | 101022 ( 81229 to 123088 ) | 132.423 ( 105.425 to 164.912 ) | 132.168 ( 107.023 to 160.375 ) | -0.14 (-0.27 to -0.01) | 83.466 |
| Portugal | Ischemic heart disease | Both | 8869 ( 6634 to 11650 ) | 17046 ( 12762 to 22751 ) | 65.891 ( 50.268 to 85.736 ) | 63.023 ( 47.662 to 83.254 ) | -0.87 (-1.03 to -0.7) | 92.198 |
| Puerto Rico | Ischemic heart disease | Both | 3690 ( 2759 to 4909 ) | 6385 ( 4788 to 8625 ) | 102.436 ( 76.968 to 135.751 ) | 78.601 ( 59.917 to 104.352 ) | -1.1 (-1.18 to -1.02) | 73.035 |
| Qatar | Ischemic heart disease | Both | 210 ( 170 to 265 ) | 1795 ( 1423 to 2299 ) | 130.502 ( 102.716 to 167.479 ) | 126.998 ( 98.572 to 158.23 ) | -0.18 (-0.24 to -0.11) | 754.762 |
| Republic of Korea | Ischemic heart disease | Both | 9813 ( 7543 to 12452 ) | 49034 ( 37822 to 61810 ) | 35.666 ( 25.944 to 46.697 ) | 54.26 ( 42.532 to 67.992 ) | 1.37 (1.08 to 1.67) | 399.684 |
| Republic of Moldova | Ischemic heart disease | Both | 4633 ( 3266 to 6211 ) | 6114 ( 4520 to 8325 ) | 121.88 ( 88.707 to 164.139 ) | 101.582 ( 75.783 to 137.975 ) | -0.87 (-0.96 to -0.78) | 31.966 |
| Romania | Ischemic heart disease | Both | 15747 ( 10465 to 21940 ) | 20952 ( 12910 to 29904 ) | 62.252 ( 42.241 to 86.669 ) | 53.416 ( 34.493 to 75.431 ) | -0.59 (-0.68 to -0.51) | 33.054 |
| Russian Federation | Ischemic heart disease | Both | 175965 ( 136452 to 222813 ) | 240640 ( 187058 to 309248 ) | 107.325 ( 83.975 to 135.312 ) | 98.272 ( 76.724 to 125.896 ) | -0.3 (-0.41 to -0.2) | 36.754 |
| Rwanda | Ischemic heart disease | Both | 819 ( 584 to 1112 ) | 2120 ( 1521 to 2874 ) | 37.142 ( 26.124 to 51.723 ) | 41.095 ( 29.376 to 57.155 ) | 0.26 (0.24 to 0.28) | 158.852 |
| Saint Kitts and Nevis | Ischemic heart disease | Both | 29 ( 21 to 39 ) | 31 ( 23 to 42 ) | 74.314 ( 56.531 to 100.063 ) | 54.189 ( 39.595 to 73.728 ) | -1.64 (-1.82 to -1.46) | 6.897 |
| Saint Lucia | Ischemic heart disease | Both | 46 ( 35 to 61 ) | 104 ( 74 to 141 ) | 55.264 ( 41.589 to 71.888 ) | 44.784 ( 32.244 to 60.996 ) | -1.77 (-2.02 to -1.52) | 126.087 |
| Saint Vincent and the Grenadines | Ischemic heart disease | Both | 48 ( 36 to 63 ) | 70 ( 51 to 98 ) | 68.054 ( 51.52 to 89.259 ) | 51.978 ( 37.94 to 72.241 ) | -1.31 (-1.48 to -1.14) | 45.833 |
| Samoa | Ischemic heart disease | Both | 46 ( 36 to 60 ) | 98 ( 74 to 127 ) | 64.036 ( 48.961 to 83.911 ) | 77.932 ( 58.467 to 101.893 ) | 0.7 (0.63 to 0.76) | 113.043 |
| San Marino | Ischemic heart disease | Both | 22 ( 16 to 30 ) | 48 ( 35 to 65 ) | 59.748 ( 44.131 to 80.759 ) | 55.797 ( 40.876 to 75.079 ) | -0.71 (-0.82 to -0.59) | 118.182 |
| Sao Tome and Principe | Ischemic heart disease | Both | 22 ( 16 to 30 ) | 43 ( 31 to 57 ) | 37.629 ( 26.689 to 51.366 ) | 45.009 ( 32.301 to 61.179 ) | 0.57 (0.52 to 0.63) | 95.455 |
| Saudi Arabia | Ischemic heart disease | Both | 4694 ( 3787 to 5814 ) | 18494 ( 14839 to 23292 ) | 76.221 ( 59.977 to 96.99 ) | 86.43 ( 67.404 to 108.99 ) | 0.42 (0.37 to 0.47) | 293.992 |
| Senegal | Ischemic heart disease | Both | 1286 ( 919 to 1758 ) | 3409 ( 2504 to 4587 ) | 45.966 ( 31.947 to 63.262 ) | 49.882 ( 36.088 to 68.416 ) | 0.3 (0.22 to 0.38) | 165.086 |
| Serbia | Ischemic heart disease | Both | 5534 ( 3803 to 7577 ) | 8521 ( 5398 to 12039 ) | 55.11 ( 36.712 to 75.352 ) | 50.401 ( 32.927 to 69.992 ) | -1.47 (-1.96 to -0.98) | 53.975 |
| Seychelles | Ischemic heart disease | Both | 18 ( 14 to 23 ) | 39 ( 29 to 51 ) | 31.07 ( 23.874 to 40.342 ) | 37.783 ( 28.263 to 49.795 ) | 0.45 (0.38 to 0.52) | 116.667 |
| Sierra Leone | Ischemic heart disease | Both | 776 ( 549 to 1068 ) | 1424 ( 1040 to 1932 ) | 42.177 ( 29.662 to 58.405 ) | 43.459 ( 30.629 to 59.416 ) | 0.04 (0.01 to 0.08) | 83.505 |
| Singapore | Ischemic heart disease | Both | 1173 ( 876 to 1536 ) | 5084 ( 3866 to 6489 ) | 54.3 ( 38.359 to 72.299 ) | 60.575 ( 46.075 to 76.784 ) | 0.26 (0.14 to 0.37) | 333.419 |
| Slovakia | Ischemic heart disease | Both | 6886 ( 5155 to 9111 ) | 9429 ( 6870 to 12498 ) | 119.078 ( 90.714 to 155.642 ) | 97.931 ( 72.124 to 128.23 ) | 0 (-0.19 to 0.18) | 36.93 |
| Slovenia | Ischemic heart disease | Both | 1163 ( 769 to 1607 ) | 2471 ( 1617 to 3471 ) | 48.243 ( 32.336 to 66.555 ) | 51.979 ( 35.442 to 72.169 ) | -0.12 (-0.29 to 0.06) | 112.468 |
| Solomon Islands | Ischemic heart disease | Both | 78 ( 61 to 102 ) | 228 ( 179 to 290 ) | 76.623 ( 58.569 to 100.935 ) | 84.015 ( 64.704 to 110.583 ) | 0.22 (0.18 to 0.27) | 192.308 |
| Somalia | Ischemic heart disease | Both | 750 ( 550 to 987 ) | 2140 ( 1579 to 2888 ) | 43.191 ( 30.836 to 60.399 ) | 45.939 ( 32.855 to 63.316 ) | 0.19 (0.17 to 0.22) | 185.333 |
| South Africa | Ischemic heart disease | Both | 11588 ( 8743 to 14860 ) | 25062 ( 19221 to 32540 ) | 62.12 ( 46.626 to 80.208 ) | 61.054 ( 46.043 to 80.072 ) | -0.04 (-0.14 to 0.07) | 116.275 |
| South Sudan | Ischemic heart disease | Both | 927 ( 652 to 1267 ) | 1501 ( 1100 to 1969 ) | 41.985 ( 29.388 to 58.179 ) | 49.73 ( 35.174 to 68.202 ) | 0.58 (0.53 to 0.62) | 61.92 |
| Spain | Ischemic heart disease | Both | 34614 ( 24917 to 45942 ) | 68446 ( 54313 to 83990 ) | 63.612 ( 46.864 to 83.698 ) | 64.867 ( 52.41 to 78.48 ) | -0.64 (-0.81 to -0.46) | 97.741 |
| Sri Lanka | Ischemic heart disease | Both | 4959 ( 3837 to 6405 ) | 17473 ( 13344 to 22973 ) | 52.603 ( 39.824 to 68.981 ) | 68.784 ( 52.288 to 90.483 ) | 1.1 (1 to 1.2) | 252.349 |
| Sudan | Ischemic heart disease | Both | 8427 ( 6637 to 10402 ) | 19499 ( 15438 to 24090 ) | 93.141 ( 71.719 to 117.588 ) | 98.274 ( 76.655 to 124.838 ) | 0.06 (0.01 to 0.12) | 131.387 |
| Suriname | Ischemic heart disease | Both | 188 ( 146 to 237 ) | 402 ( 303 to 523 ) | 80.079 ( 60.733 to 102.801 ) | 68.057 ( 50.986 to 88.789 ) | -0.76 (-0.82 to -0.69) | 113.83 |
| Sweden | Ischemic heart disease | Both | 25499 ( 19546 to 32363 ) | 35647 ( 27019 to 45842 ) | 159.382 ( 123.378 to 200.086 ) | 147.253 ( 114.02 to 186.99 ) | -0.71 (-0.9 to -0.52) | 39.798 |
| Switzerland | Ischemic heart disease | Both | 6554 ( 4344 to 9030 ) | 10242 ( 7265 to 14349 ) | 60.62 ( 41.266 to 81.207 ) | 51.459 ( 36.908 to 70.932 ) | -0.34 (-0.85 to 0.18) | 56.271 |
| Syrian Arab Republic | Ischemic heart disease | Both | 6378 ( 5139 to 7855 ) | 15441 ( 12085 to 19695 ) | 119.702 ( 94.248 to 150.989 ) | 129.557 ( 100.13 to 165.583 ) | 0.38 (0.28 to 0.47) | 142.098 |
| Taiwan (Province of China) | Ischemic heart disease | Both | 7560 ( 5641 to 10011 ) | 25172 ( 18969 to 33554 ) | 55.491 ( 41.287 to 73.567 ) | 58.372 ( 44.061 to 77.944 ) | 0.1 (0.03 to 0.16) | 232.963 |
| Tajikistan | Ischemic heart disease | Both | 2208 ( 1604 to 2935 ) | 4191 ( 3117 to 5487 ) | 85.07 ( 59.809 to 116.526 ) | 89.023 ( 63.676 to 120.757 ) | 0.09 (0.03 to 0.15) | 89.81 |
| Thailand | Ischemic heart disease | Both | 24548 ( 19210 to 31332 ) | 87026 ( 66925 to 112947 ) | 80.646 ( 61.376 to 105.355 ) | 81.405 ( 62.847 to 105.131 ) | 0.03 (-0.03 to 0.09) | 254.514 |
| Timor-Leste | Ischemic heart disease | Both | 109 ( 87 to 135 ) | 460 ( 347 to 603 ) | 47.031 ( 35.904 to 60.638 ) | 59.826 ( 45.71 to 78.521 ) | 1.01 (0.94 to 1.07) | 322.018 |
| Togo | Ischemic heart disease | Both | 384 ( 281 to 516 ) | 1279 ( 947 to 1715 ) | 37.99 ( 27.03 to 52.39 ) | 41.817 ( 29.96 to 57.465 ) | 0.27 (0.23 to 0.31) | 233.073 |
| Tokelau | Ischemic heart disease | Both | 1 ( 1 to 1 ) | 1 ( 1 to 2 ) | 67.598 ( 51.239 to 88.983 ) | 83.732 ( 63.209 to 111.13 ) | 0.71 (0.68 to 0.74) | 0 |
| Tonga | Ischemic heart disease | Both | 40 ( 30 to 51 ) | 70 ( 53 to 90 ) | 82.647 ( 62.338 to 107.191 ) | 90.615 ( 68.626 to 118.361 ) | 0.31 (0.29 to 0.33) | 75 |
| Trinidad and Tobago | Ischemic heart disease | Both | 667 ( 500 to 865 ) | 1519 ( 1159 to 2022 ) | 83.646 ( 63.377 to 109.584 ) | 80.846 ( 61.291 to 107.472 ) | -0.29 (-0.37 to -0.22) | 127.736 |
| Tunisia | Ischemic heart disease | Both | 4889 ( 3836 to 6127 ) | 12352 ( 9649 to 15316 ) | 102.794 ( 79.762 to 131.578 ) | 97.463 ( 76.255 to 122.62 ) | -0.3 (-0.36 to -0.24) | 152.649 |
| Turkey | Ischemic heart disease | Both | 34259 ( 27413 to 42653 ) | 97155 ( 76066 to 122669 ) | 101.079 ( 79.32 to 128.978 ) | 107.053 ( 83.907 to 134.411 ) | 0.16 (0.1 to 0.22) | 183.59 |
| Turkmenistan | Ischemic heart disease | Both | 1681 ( 1175 to 2289 ) | 3266 ( 2302 to 4376 ) | 105.024 ( 72.369 to 144.355 ) | 99.908 ( 69.34 to 135.495 ) | -0.16 (-0.24 to -0.08) | 94.289 |
| Tuvalu | Ischemic heart disease | Both | 3 ( 2 to 4 ) | 7 ( 5 to 9 ) | 61.455 ( 46.338 to 79.432 ) | 78.459 ( 60.018 to 101.456 ) | 0.83 (0.78 to 0.88) | 133.333 |
| Uganda | Ischemic heart disease | Both | 2348 ( 1680 to 3237 ) | 5731 ( 4144 to 7686 ) | 43.472 ( 30.391 to 60.218 ) | 46.036 ( 32.158 to 64.265 ) | 0.07 (0.02 to 0.12) | 144.08 |
| Ukraine | Ischemic heart disease | Both | 76933 ( 58859 to 98522 ) | 91267 ( 71140 to 116454 ) | 113.245 ( 88.227 to 143.873 ) | 112.492 ( 88.31 to 142.434 ) | 0.11 (0.05 to 0.18) | 18.632 |
| United Arab Emirates | Ischemic heart disease | Both | 601 ( 488 to 746 ) | 5281 ( 4114 to 6822 ) | 106.69 ( 83.289 to 137.524 ) | 101.445 ( 77.846 to 127.306 ) | -0.15 (-0.22 to -0.09) | 778.702 |
| United Kingdom | Ischemic heart disease | Both | 58845 ( 42185 to 78218 ) | 64754 ( 46834 to 85203 ) | 63.057 ( 46.053 to 82.122 ) | 46.333 ( 34.104 to 59.767 ) | -1.59 (-1.75 to -1.43) | 10.042 |
| United Republic of Tanzania | Ischemic heart disease | Both | 3730 ( 2617 to 5172 ) | 11895 ( 8700 to 15926 ) | 41.808 ( 28.875 to 58.109 ) | 54.34 ( 38.562 to 75.555 ) | 1.21 (1.09 to 1.33) | 218.901 |
| United States of America | Ischemic heart disease | Both | 339635 ( 252780 to 434485 ) | 537020 ( 415961 to 676249 ) | 103.512 ( 78.376 to 131.155 ) | 88.185 ( 69.038 to 109.698 ) | -0.93 (-1.02 to -0.83) | 58.117 |
| United States Virgin Islands | Ischemic heart disease | Both | 61 ( 47 to 79 ) | 157 ( 113 to 213 ) | 85.827 ( 65.394 to 110.957 ) | 82.674 ( 62.313 to 110.847 ) | -0.23 (-0.27 to -0.19) | 157.377 |
| Uruguay | Ischemic heart disease | Both | 2295 ( 1644 to 3146 ) | 2536 ( 1721 to 3508 ) | 59.778 ( 43.71 to 80.754 ) | 43.688 ( 30.532 to 59.051 ) | -1.15 (-1.24 to -1.06) | 10.501 |
| Uzbekistan | Ischemic heart disease | Both | 12128 ( 8658 to 16332 ) | 21926 ( 15452 to 29284 ) | 110.676 ( 77.638 to 151.494 ) | 101.03 ( 68.75 to 140.931 ) | -0.24 (-0.32 to -0.17) | 80.788 |
| Vanuatu | Ischemic heart disease | Both | 36 ( 28 to 47 ) | 112 ( 87 to 145 ) | 74.566 ( 57.002 to 96.421 ) | 81.955 ( 62.133 to 107.176 ) | 0.28 (0.26 to 0.3) | 211.111 |
| Venezuela (Bolivarian Republic of) | Ischemic heart disease | Both | 6992 ( 5383 to 8830 ) | 24259 ( 18488 to 31391 ) | 75.686 ( 57.261 to 97.697 ) | 85.001 ( 64.528 to 110.732 ) | 0.42 (0.4 to 0.45) | 246.954 |
| Viet Nam | Ischemic heart disease | Both | 12725 ( 9816 to 16646 ) | 41470 ( 31731 to 54581 ) | 34.246 ( 26.367 to 45.039 ) | 47.528 ( 36.24 to 63.35 ) | 1.36 (1.26 to 1.46) | 225.894 |
| Yemen | Ischemic heart disease | Both | 3801 ( 3037 to 4794 ) | 12005 ( 9516 to 14894 ) | 79.833 ( 62.849 to 101.622 ) | 84.538 ( 65.713 to 106.894 ) | 0.19 (0.16 to 0.22) | 215.838 |
| Zambia | Ischemic heart disease | Both | 995 ( 705 to 1349 ) | 2597 ( 1916 to 3484 ) | 44.334 ( 31.182 to 61.68 ) | 46.491 ( 33.167 to 64.426 ) | 0.09 (0.06 to 0.11) | 161.005 |
| Zimbabwe | Ischemic heart disease | Both | 1482 ( 1054 to 2046 ) | 2637 ( 1885 to 3633 ) | 45.639 ( 32.057 to 63.254 ) | 50.896 ( 36.481 to 70.409 ) | 0.34 (0.28 to 0.39) | 77.935 |

**Table S6 The YLDs of SHF due to IHD in 1990 and 2021 across countries and territories.**

| Region | cause | sex | 1990 YLDs cases(95% UI) | 2021 YLDs cases(95% UI) | 1990 ASYR(95% UI) | 2021 ASYR(95% UI) | 1990-2021 EAPC（95%CI） | 1990-2021 YLDs cases changes |
| --- | --- | --- | --- | --- | --- | --- | --- | --- |
| Afghanistan | Ischemic heart disease | Both | 705 ( 439 to 1053 ) | 1256 ( 786 to 1900 ) | 11.569 ( 7.223 to 17.334 ) | 11.97 ( 7.394 to 18.226 ) | 0.07 (0.01 to 0.13) | 78.156 |
| Albania | Ischemic heart disease | Both | 279 ( 167 to 424 ) | 652 ( 368 to 1006 ) | 14.335 ( 8.533 to 21.808 ) | 14.985 ( 8.672 to 22.894 ) | 0.37 (0.29 to 0.46) | 133.692 |
| Algeria | Ischemic heart disease | Both | 2018 ( 1252 to 3004 ) | 5679 ( 3463 to 8373 ) | 17.217 ( 10.928 to 25.125 ) | 16.753 ( 10.517 to 24.819 ) | -0.3 (-0.37 to -0.23) | 181.417 |
| American Samoa | Ischemic heart disease | Both | 2 ( 1 to 3 ) | 5 ( 3 to 8 ) | 11.261 ( 7.008 to 16.862 ) | 12.889 ( 8.029 to 19.334 ) | 0.48 (0.43 to 0.54) | 150 |
| Andorra | Ischemic heart disease | Both | 6 ( 3 to 9 ) | 17 ( 11 to 26 ) | 10.399 ( 6.208 to 15.964 ) | 10.799 ( 6.588 to 16.69 ) | -0.18 (-0.25 to -0.11) | 183.333 |
| Angola | Ischemic heart disease | Both | 225 ( 140 to 347 ) | 745 ( 447 to 1135 ) | 7.785 ( 4.82 to 12.248 ) | 8.137 ( 4.854 to 12.734 ) | 0.1 (0.08 to 0.11) | 231.111 |
| Antigua and Barbuda | Ischemic heart disease | Both | 7 ( 4 to 10 ) | 8 ( 5 to 13 ) | 11.773 ( 7.219 to 17.488 ) | 8.667 ( 5.309 to 13.332 ) | -1.69 (-1.87 to -1.51) | 14.286 |
| Argentina | Ischemic heart disease | Both | 2490 ( 1484 to 3806 ) | 3737 ( 2222 to 5732 ) | 8.211 ( 4.898 to 12.401 ) | 6.571 ( 3.94 to 10.004 ) | -0.9 (-0.99 to -0.81) | 50.08 |
| Armenia | Ischemic heart disease | Both | 472 ( 284 to 716 ) | 860 ( 526 to 1266 ) | 19.826 ( 11.661 to 30.014 ) | 19.926 ( 12.205 to 29.294 ) | 0.05 (-0.03 to 0.13) | 82.203 |
| Australia | Ischemic heart disease | Both | 4485 ( 2695 to 6733 ) | 8688 ( 5514 to 12966 ) | 22.765 ( 14.028 to 34.094 ) | 17.839 ( 11.447 to 26.597 ) | -1.43 (-1.68 to -1.18) | 93.712 |
| Austria | Ischemic heart disease | Both | 2446 ( 1534 to 3644 ) | 2562 ( 1574 to 3930 ) | 20.007 ( 12.598 to 29.783 ) | 12.606 ( 7.667 to 19.245 ) | -2.39 (-2.66 to -2.12) | 4.742 |
| Azerbaijan | Ischemic heart disease | Both | 744 ( 443 to 1149 ) | 1388 ( 811 to 2145 ) | 16.928 ( 9.923 to 26.308 ) | 16.845 ( 9.667 to 26.484 ) | 0.03 (-0.03 to 0.09) | 86.559 |
| Bahamas | Ischemic heart disease | Both | 12 ( 7 to 18 ) | 28 ( 17 to 44 ) | 8.359 ( 5.107 to 12.545 ) | 7.782 ( 4.73 to 12.186 ) | -1.43 (-1.69 to -1.16) | 133.333 |
| Bahrain | Ischemic heart disease | Both | 43 ( 27 to 63 ) | 192 ( 120 to 280 ) | 20.701 ( 13.576 to 30.77 ) | 18.818 ( 12.062 to 27.701 ) | -0.43 (-0.48 to -0.39) | 346.512 |
| Bangladesh | Ischemic heart disease | Both | 3675 ( 2344 to 5543 ) | 13855 ( 8745 to 20638 ) | 7.935 ( 5.095 to 11.839 ) | 10.527 ( 6.634 to 15.669 ) | 1 (0.9 to 1.11) | 277.007 |
| Barbados | Ischemic heart disease | Both | 42 ( 26 to 64 ) | 56 ( 34 to 85 ) | 13.165 ( 8.163 to 20.011 ) | 10.741 ( 6.7 to 16.057 ) | -1.03 (-1.16 to -0.9) | 33.333 |
| Belarus | Ischemic heart disease | Both | 2598 ( 1521 to 3957 ) | 3855 ( 2296 to 5839 ) | 20.985 ( 12.31 to 31.881 ) | 23.517 ( 13.933 to 35.475 ) | 0.56 (0.49 to 0.62) | 48.383 |
| Belgium | Ischemic heart disease | Both | 1852 ( 1073 to 2849 ) | 2173 ( 1372 to 3180 ) | 11.945 ( 7.037 to 18.392 ) | 8.615 ( 5.455 to 12.696 ) | -0.82 (-0.97 to -0.67) | 17.333 |
| Belize | Ischemic heart disease | Both | 14 ( 9 to 21 ) | 28 ( 17 to 42 ) | 14.772 ( 9.322 to 22.468 ) | 10.482 ( 6.353 to 16.195 ) | -1.76 (-1.9 to -1.61) | 100 |
| Benin | Ischemic heart disease | Both | 110 ( 67 to 172 ) | 319 ( 195 to 485 ) | 6.01 ( 3.655 to 9.401 ) | 7.021 ( 4.401 to 10.717 ) | 0.53 (0.5 to 0.56) | 190 |
| Bermuda | Ischemic heart disease | Both | 13 ( 8 to 19 ) | 22 ( 13 to 33 ) | 21.273 ( 13.059 to 31.986 ) | 15.399 ( 9.268 to 23.047 ) | -1.18 (-1.26 to -1.1) | 69.231 |
| Bhutan | Ischemic heart disease | Both | 18 ( 11 to 27 ) | 64 ( 40 to 96 ) | 7.889 ( 4.945 to 11.895 ) | 10.75 ( 6.825 to 16.036 ) | 1.15 (1.1 to 1.2) | 255.556 |
| Bolivia (Plurinational State of) | Ischemic heart disease | Both | 341 ( 214 to 515 ) | 939 ( 572 to 1404 ) | 11.637 ( 7.347 to 17.37 ) | 11.211 ( 7.038 to 16.876 ) | -0.15 (-0.23 to -0.08) | 175.367 |
| Bosnia and Herzegovina | Ischemic heart disease | Both | 498 ( 299 to 763 ) | 776 ( 444 to 1182 ) | 13.993 ( 8.354 to 21.266 ) | 12.353 ( 7.246 to 18.854 ) | -0.6 (-0.67 to -0.53) | 55.823 |
| Botswana | Ischemic heart disease | Both | 35 ( 21 to 57 ) | 114 ( 69 to 170 ) | 8.153 ( 4.941 to 12.771 ) | 9.486 ( 5.813 to 14.745 ) | 0.44 (0.42 to 0.46) | 225.714 |
| Brazil | Ischemic heart disease | Both | 7923 ( 5038 to 11510 ) | 23535 ( 15068 to 34984 ) | 9.924 ( 6.274 to 14.497 ) | 9.616 ( 6.174 to 14.263 ) | 0 (-0.05 to 0.06) | 197.047 |
| Brunei Darussalam | Ischemic heart disease | Both | 7 ( 4 to 11 ) | 19 ( 12 to 30 ) | 5.786 ( 3.304 to 9.029 ) | 5.488 ( 3.136 to 8.521 ) | -0.32 (-0.37 to -0.28) | 171.429 |
| Bulgaria | Ischemic heart disease | Both | 1530 ( 890 to 2459 ) | 1378 ( 797 to 2157 ) | 14.19 ( 8.527 to 22.078 ) | 9.34 ( 5.676 to 14.284 ) | -2.53 (-2.85 to -2.22) | -9.935 |
| Burkina Faso | Ischemic heart disease | Both | 193 ( 116 to 312 ) | 480 ( 288 to 749 ) | 5.424 ( 3.406 to 8.575 ) | 5.977 ( 3.537 to 9.546 ) | 0.31 (0.28 to 0.33) | 148.705 |
| Burundi | Ischemic heart disease | Both | 136 ( 83 to 218 ) | 303 ( 186 to 465 ) | 6.884 ( 4.278 to 10.822 ) | 7.696 ( 4.718 to 12.181 ) | 0.36 (0.34 to 0.39) | 122.794 |
| Cabo Verde | Ischemic heart disease | Both | 16 ( 10 to 26 ) | 34 ( 21 to 53 ) | 6.902 ( 4.211 to 10.882 ) | 8.187 ( 4.961 to 12.77 ) | 0.56 (0.5 to 0.62) | 112.5 |
| Cambodia | Ischemic heart disease | Both | 251 ( 157 to 371 ) | 853 ( 555 to 1285 ) | 6.683 ( 4.27 to 9.848 ) | 8.233 ( 5.22 to 12.48 ) | 0.74 (0.72 to 0.76) | 239.841 |
| Cameroon | Ischemic heart disease | Both | 176 ( 105 to 282 ) | 655 ( 392 to 1048 ) | 4.824 ( 2.928 to 7.569 ) | 6.164 ( 3.727 to 9.823 ) | 0.84 (0.73 to 0.95) | 272.159 |
| Canada | Ischemic heart disease | Both | 8514 ( 5262 to 12409 ) | 16188 ( 9919 to 24229 ) | 25.883 ( 16.245 to 37.845 ) | 21.304 ( 13.349 to 32.012 ) | -0.72 (-0.94 to -0.51) | 90.134 |
| Central African Republic | Ischemic heart disease | Both | 56 ( 33 to 89 ) | 121 ( 72 to 189 ) | 6.927 ( 4.218 to 10.705 ) | 7.616 ( 4.682 to 11.899 ) | 0.27 (0.23 to 0.3) | 116.071 |
| Chad | Ischemic heart disease | Both | 157 ( 94 to 249 ) | 348 ( 210 to 541 ) | 6.195 ( 3.844 to 9.866 ) | 7.142 ( 4.292 to 11.406 ) | 0.46 (0.44 to 0.48) | 121.656 |
| Chile | Ischemic heart disease | Both | 846 ( 508 to 1268 ) | 1956 ( 1191 to 2993 ) | 9.038 ( 5.466 to 13.667 ) | 7.649 ( 4.685 to 11.667 ) | -0.83 (-0.94 to -0.71) | 131.206 |
| China | Ischemic heart disease | Both | 56966 ( 36250 to 84239 ) | 256916 ( 162518 to 382038 ) | 8.16 ( 5.25 to 12.294 ) | 12.868 ( 8.229 to 19.008 ) | 1.94 (1.74 to 2.14) | 350.999 |
| Colombia | Ischemic heart disease | Both | 2205 ( 1375 to 3258 ) | 8963 ( 5618 to 13391 ) | 13.155 ( 8.04 to 19.93 ) | 16.059 ( 10.029 to 24.311 ) | 0.83 (0.78 to 0.87) | 306.485 |
| Comoros | Ischemic heart disease | Both | 12 ( 7 to 20 ) | 37 ( 22 to 58 ) | 7.683 ( 4.661 to 12.011 ) | 8.599 ( 5.292 to 13.863 ) | 0.36 (0.31 to 0.41) | 208.333 |
| Congo | Ischemic heart disease | Both | 63 ( 37 to 97 ) | 185 ( 110 to 279 ) | 7.64 ( 4.726 to 11.864 ) | 8.833 ( 5.363 to 13.519 ) | 0.45 (0.44 to 0.46) | 193.651 |
| Cook Islands | Ischemic heart disease | Both | 1 ( 0 to 1 ) | 2 ( 1 to 3 ) | 6.593 ( 3.99 to 9.908 ) | 9.068 ( 5.495 to 13.76 ) | 0.74 (0.63 to 0.84) | 100 |
| Costa Rica | Ischemic heart disease | Both | 264 ( 169 to 389 ) | 734 ( 453 to 1091 ) | 15.364 ( 9.728 to 22.913 ) | 13.343 ( 8.24 to 19.926 ) | -0.41 (-0.49 to -0.32) | 178.03 |
| Coted'Ivoire | Ischemic heart disease | Both | 198 ( 119 to 309 ) | 715 ( 434 to 1100 ) | 6.426 ( 3.929 to 10.117 ) | 7.774 ( 4.759 to 11.949 ) | 0.62 (0.6 to 0.64) | 261.111 |
| Croatia | Ischemic heart disease | Both | 699 ( 407 to 1066 ) | 847 ( 460 to 1355 ) | 12.578 ( 7.319 to 19.284 ) | 8.991 ( 5.018 to 14.292 ) | -1.79 (-2.22 to -1.36) | 21.173 |
| Cuba | Ischemic heart disease | Both | 2475 ( 1522 to 3740 ) | 3277 ( 2067 to 4910 ) | 24.331 ( 15.142 to 36.814 ) | 16.249 ( 10.33 to 24.206 ) | -1.5 (-1.57 to -1.42) | 32.404 |
| Cyprus | Ischemic heart disease | Both | 68 ( 38 to 107 ) | 207 ( 125 to 314 ) | 8.959 ( 5.273 to 13.802 ) | 10.012 ( 6.154 to 15.001 ) | -0.48 (-0.79 to -0.18) | 204.412 |
| Czechia | Ischemic heart disease | Both | 3327 ( 1896 to 5170 ) | 4230 ( 2440 to 6468 ) | 24.582 ( 14.23 to 37.699 ) | 18.706 ( 11.14 to 28.434 ) | -0.17 (-0.52 to 0.18) | 27.142 |
| Democratic People's Republic of Korea | Ischemic heart disease | Both | 1122 ( 694 to 1660 ) | 3208 ( 2007 to 4877 ) | 8.354 ( 5.161 to 12.458 ) | 10.656 ( 6.687 to 16.043 ) | 0.85 (0.8 to 0.89) | 185.918 |
| Democratic Republic of the Congo | Ischemic heart disease | Both | 877 ( 523 to 1380 ) | 2263 ( 1370 to 3546 ) | 7.62 ( 4.659 to 11.897 ) | 7.839 ( 4.799 to 12.182 ) | 0.01 (-0.02 to 0.03) | 158.039 |
| Denmark | Ischemic heart disease | Both | 1107 ( 643 to 1679 ) | 1351 ( 811 to 2037 ) | 13.281 ( 7.813 to 19.976 ) | 10.784 ( 6.419 to 16.125 ) | -0.9 (-1.09 to -0.71) | 22.042 |
| Djibouti | Ischemic heart disease | Both | 8 ( 5 to 13 ) | 48 ( 29 to 74 ) | 7.911 ( 4.758 to 12.632 ) | 9.475 ( 5.915 to 15.044 ) | 0.62 (0.59 to 0.66) | 500 |
| Dominica | Ischemic heart disease | Both | 5 ( 3 to 8 ) | 6 ( 4 to 9 ) | 8.259 ( 5.028 to 12.56 ) | 7.565 ( 4.584 to 11.663 ) | -0.92 (-1.1 to -0.75) | 20 |
| Dominican Republic | Ischemic heart disease | Both | 534 ( 336 to 798 ) | 1534 ( 942 to 2303 ) | 15.47 ( 9.694 to 22.835 ) | 15.764 ( 9.65 to 23.663 ) | 0.2 (0.14 to 0.27) | 187.266 |
| Ecuador | Ischemic heart disease | Both | 529 ( 329 to 793 ) | 1922 ( 1207 to 2895 ) | 10.26 ( 6.219 to 15.499 ) | 11.981 ( 7.516 to 18.04 ) | 0.01 (-0.27 to 0.29) | 263.327 |
| Egypt | Ischemic heart disease | Both | 3685 ( 2360 to 5392 ) | 10358 ( 6530 to 15162 ) | 14.324 ( 9.232 to 21.408 ) | 17.854 ( 11.383 to 26.145 ) | 0.79 (0.71 to 0.87) | 181.085 |
| El Salvador | Ischemic heart disease | Both | 500 ( 317 to 744 ) | 1135 ( 726 to 1729 ) | 16.937 ( 10.697 to 25.182 ) | 17.285 ( 10.984 to 26.459 ) | 0 (-0.03 to 0.02) | 127 |
| Equatorial Guinea | Ischemic heart disease | Both | 11 ( 7 to 17 ) | 38 ( 24 to 58 ) | 7.35 ( 4.54 to 11.308 ) | 8.952 ( 5.652 to 13.929 ) | 0.69 (0.66 to 0.72) | 245.455 |
| Eritrea | Ischemic heart disease | Both | 50 ( 30 to 78 ) | 155 ( 93 to 237 ) | 6.402 ( 3.938 to 10.028 ) | 7.204 ( 4.437 to 11.451 ) | 0.39 (0.37 to 0.42) | 210 |
| Estonia | Ischemic heart disease | Both | 385 ( 226 to 608 ) | 323 ( 182 to 528 ) | 19.167 ( 11.387 to 29.833 ) | 10.577 ( 6.085 to 17.124 ) | -3.31 (-3.7 to -2.93) | -16.104 |
| Eswatini | Ischemic heart disease | Both | 17 ( 10 to 27 ) | 38 ( 23 to 59 ) | 7.575 ( 4.708 to 11.945 ) | 8.638 ( 5.37 to 13.536 ) | 0.44 (0.42 to 0.47) | 123.529 |
| Ethiopia | Ischemic heart disease | Both | 802 ( 497 to 1245 ) | 2233 ( 1398 to 3399 ) | 5.288 ( 3.321 to 8.107 ) | 5.875 ( 3.804 to 9.048 ) | 0.31 (0.3 to 0.32) | 178.429 |
| Fiji | Ischemic heart disease | Both | 38 ( 24 to 56 ) | 88 ( 56 to 132 ) | 13.04 ( 8.02 to 19.379 ) | 14.131 ( 8.875 to 20.718 ) | 0.28 (0.25 to 0.3) | 131.579 |
| Finland | Ischemic heart disease | Both | 1204 ( 691 to 1877 ) | 2096 ( 1240 to 3202 ) | 16.802 ( 9.824 to 25.956 ) | 14.653 ( 8.961 to 22.389 ) | -0.6 (-1 to -0.21) | 74.086 |
| France | Ischemic heart disease | Both | 19474 ( 11730 to 29197 ) | 34254 ( 21770 to 50693 ) | 22.526 ( 13.742 to 33.648 ) | 21.439 ( 13.371 to 32.319 ) | -0.52 (-0.71 to -0.32) | 75.896 |
| Gabon | Ischemic heart disease | Both | 36 ( 21 to 57 ) | 72 ( 44 to 111 ) | 7.19 ( 4.359 to 11.336 ) | 8.421 ( 5.233 to 13.311 ) | 0.51 (0.49 to 0.52) | 100 |
| Gambia | Ischemic heart disease | Both | 20 ( 12 to 31 ) | 69 ( 42 to 106 ) | 6.707 ( 4.079 to 10.714 ) | 7.914 ( 4.87 to 12.541 ) | 0.47 (0.44 to 0.51) | 245 |
| Georgia | Ischemic heart disease | Both | 1156 ( 680 to 1767 ) | 564 ( 259 to 929 ) | 19.911 ( 11.869 to 30.401 ) | 9.17 ( 4.414 to 15.257 ) | -3.76 (-4.14 to -3.38) | -51.211 |
| Germany | Ischemic heart disease | Both | 19349 ( 11637 to 29975 ) | 27654 ( 16637 to 41669 ) | 14.97 ( 9.082 to 22.832 ) | 12.933 ( 7.774 to 19.48 ) | 0.06 (-0.2 to 0.33) | 42.922 |
| Ghana | Ischemic heart disease | Both | 396 ( 235 to 620 ) | 1284 ( 777 to 1949 ) | 7.995 ( 4.832 to 12.582 ) | 9.259 ( 5.622 to 14.41 ) | 0.51 (0.48 to 0.53) | 224.242 |
| Greece | Ischemic heart disease | Both | 1749 ( 1067 to 2576 ) | 1832 ( 1112 to 2739 ) | 11.788 ( 7.331 to 17.267 ) | 7.189 ( 4.485 to 10.794 ) | -2.55 (-2.84 to -2.26) | 4.746 |
| Greenland | Ischemic heart disease | Both | 4 ( 2 to 6 ) | 7 ( 4 to 11 ) | 13.576 ( 7.363 to 21.362 ) | 12.198 ( 6.464 to 19.129 ) | -0.52 (-0.62 to -0.43) | 75 |
| Grenada | Ischemic heart disease | Both | 9 ( 6 to 14 ) | 9 ( 5 to 14 ) | 11.71 ( 7.248 to 17.78 ) | 8.868 ( 5.112 to 13.723 ) | -1.37 (-1.49 to -1.26) | 0 |
| Guam | Ischemic heart disease | Both | 7 ( 4 to 10 ) | 36 ( 23 to 55 ) | 11.168 ( 6.859 to 16.763 ) | 16.828 ( 10.489 to 25.515 ) | 1.45 (1.28 to 1.62) | 414.286 |
| Guatemala | Ischemic heart disease | Both | 481 ( 296 to 714 ) | 1649 ( 1022 to 2434 ) | 15.731 ( 9.749 to 23.734 ) | 15.679 ( 9.689 to 23.283 ) | -0.06 (-0.1 to -0.01) | 242.827 |
| Guinea | Ischemic heart disease | Both | 164 ( 98 to 259 ) | 336 ( 206 to 518 ) | 5.594 ( 3.415 to 8.989 ) | 6.756 ( 4.143 to 10.423 ) | 0.62 (0.6 to 0.64) | 104.878 |
| Guinea-Bissau | Ischemic heart disease | Both | 18 ( 11 to 29 ) | 40 ( 24 to 62 ) | 5.887 ( 3.543 to 9.292 ) | 6.921 ( 4.332 to 10.79 ) | 0.56 (0.54 to 0.57) | 122.222 |
| Guyana | Ischemic heart disease | Both | 32 ( 19 to 48 ) | 45 ( 27 to 71 ) | 9.351 ( 5.639 to 14.163 ) | 8.29 ( 4.951 to 12.952 ) | -0.55 (-0.71 to -0.39) | 40.625 |
| Haiti | Ischemic heart disease | Both | 298 ( 174 to 447 ) | 731 ( 449 to 1070 ) | 11.612 ( 6.918 to 17.233 ) | 12.717 ( 7.83 to 18.984 ) | 0.28 (0.27 to 0.29) | 145.302 |
| Honduras | Ischemic heart disease | Both | 189 ( 113 to 286 ) | 654 ( 409 to 987 ) | 10.172 ( 6.105 to 15.334 ) | 11.427 ( 7.048 to 17.328 ) | 0.44 (0.39 to 0.48) | 246.032 |
| Hungary | Ischemic heart disease | Both | 1764 ( 968 to 2844 ) | 2174 ( 1184 to 3428 ) | 12.477 ( 7.02 to 19.568 ) | 10.327 ( 5.825 to 16.281 ) | -0.73 (-0.83 to -0.63) | 23.243 |
| Iceland | Ischemic heart disease | Both | 25 ( 14 to 39 ) | 46 ( 27 to 70 ) | 8.589 ( 4.882 to 13.083 ) | 7.696 ( 4.493 to 11.677 ) | -0.87 (-1.15 to -0.58) | 84 |
| India | Ischemic heart disease | Both | 39813 ( 26072 to 57261 ) | 131779 ( 85516 to 192361 ) | 9.662 ( 6.326 to 13.965 ) | 11.876 ( 7.705 to 17.266 ) | 0.83 (0.78 to 0.88) | 230.995 |
| Indonesia | Ischemic heart disease | Both | 6730 ( 4346 to 9855 ) | 18717 ( 12207 to 27477 ) | 7.828 ( 5.003 to 11.712 ) | 9.212 ( 5.901 to 13.656 ) | 0.7 (0.65 to 0.74) | 178.113 |
| Iran (Islamic Republic of) | Ischemic heart disease | Both | 4734 ( 3072 to 6770 ) | 14226 ( 9351 to 20520 ) | 19.317 ( 12.904 to 27.905 ) | 18.79 ( 12.399 to 27.29 ) | -0.24 (-0.31 to -0.17) | 200.507 |
| Iraq | Ischemic heart disease | Both | 1340 ( 853 to 1972 ) | 4253 ( 2690 to 6205 ) | 16.707 ( 10.633 to 24.766 ) | 18.761 ( 11.889 to 27.503 ) | 0.41 (0.39 to 0.44) | 217.388 |
| Ireland | Ischemic heart disease | Both | 570 ( 326 to 867 ) | 1098 ( 660 to 1679 ) | 14.065 ( 8.305 to 21.394 ) | 13.37 ( 8.093 to 20.439 ) | -0.3 (-0.36 to -0.24) | 92.632 |
| Israel | Ischemic heart disease | Both | 583 ( 334 to 904 ) | 1394 ( 818 to 2097 ) | 12.335 ( 7.327 to 18.983 ) | 10.619 ( 6.3 to 16.162 ) | -0.98 (-1.15 to -0.8) | 139.108 |
| Italy | Ischemic heart disease | Both | 8820 ( 5143 to 13631 ) | 16919 ( 10770 to 24995 ) | 10.078 ( 6.092 to 15.331 ) | 10.929 ( 6.958 to 16.058 ) | 0.35 (-0.23 to 0.93) | 91.825 |
| Jamaica | Ischemic heart disease | Both | 137 ( 84 to 211 ) | 272 ( 166 to 425 ) | 7.325 ( 4.505 to 11.197 ) | 8.381 ( 5.013 to 13.492 ) | -0.28 (-0.44 to -0.12) | 98.54 |
| Japan | Ischemic heart disease | Both | 10848 ( 6665 to 16653 ) | 25658 ( 16305 to 37064 ) | 6.834 ( 4.275 to 10.445 ) | 6.858 ( 4.446 to 10.063 ) | -0.05 (-0.17 to 0.08) | 136.523 |
| Jordan | Ischemic heart disease | Both | 223 ( 143 to 327 ) | 1139 ( 715 to 1648 ) | 15.576 ( 10.04 to 23.425 ) | 14.976 ( 9.662 to 22.335 ) | -0.44 (-0.52 to -0.36) | 410.762 |
| Kazakhstan | Ischemic heart disease | Both | 2107 ( 1273 to 3336 ) | 2334 ( 1351 to 3708 ) | 18.886 ( 11.454 to 29.694 ) | 15.569 ( 8.851 to 24.533 ) | -1.06 (-1.29 to -0.84) | 10.774 |
| Kenya | Ischemic heart disease | Both | 548 ( 351 to 848 ) | 1595 ( 1019 to 2409 ) | 7.815 ( 5.092 to 11.895 ) | 8.372 ( 5.376 to 12.945 ) | -0.18 (-0.38 to 0.02) | 191.058 |
| Kiribati | Ischemic heart disease | Both | 3 ( 2 to 4 ) | 7 ( 4 to 10 ) | 10.338 ( 6.318 to 15.111 ) | 11.691 ( 7.124 to 17.141 ) | 0.4 (0.39 to 0.41) | 133.333 |
| Kuwait | Ischemic heart disease | Both | 151 ( 95 to 228 ) | 748 ( 484 to 1124 ) | 20.431 ( 12.852 to 30.989 ) | 22.462 ( 14.526 to 33.426 ) | 0.42 (0.37 to 0.46) | 395.364 |
| Kyrgyzstan | Ischemic heart disease | Both | 488 ( 294 to 742 ) | 704 ( 419 to 1079 ) | 17.918 ( 10.6 to 27.639 ) | 18.152 ( 10.611 to 28.008 ) | 0.22 (0.15 to 0.29) | 44.262 |
| Lao People's Democratic Republic | Ischemic heart disease | Both | 151 ( 92 to 224 ) | 395 ( 252 to 590 ) | 8.832 ( 5.578 to 13.193 ) | 10.026 ( 6.249 to 15.15 ) | 0.54 (0.49 to 0.58) | 161.589 |
| Latvia | Ischemic heart disease | Both | 729 ( 432 to 1120 ) | 681 ( 391 to 1055 ) | 20.534 ( 12.263 to 31.395 ) | 14.927 ( 8.587 to 23.07 ) | -1.6 (-1.79 to -1.41) | -6.584 |
| Lebanon | Ischemic heart disease | Both | 395 ( 249 to 577 ) | 1252 ( 786 to 1854 ) | 19.308 ( 12.159 to 28.469 ) | 20.092 ( 12.566 to 29.594 ) | 0.15 (0.12 to 0.18) | 216.962 |
| Lesotho | Ischemic heart disease | Both | 46 ( 28 to 73 ) | 63 ( 38 to 99 ) | 6.216 ( 3.812 to 9.832 ) | 7.234 ( 4.556 to 11.351 ) | 0.5 (0.49 to 0.52) | 36.957 |
| Liberia | Ischemic heart disease | Both | 67 ( 39 to 108 ) | 143 ( 87 to 216 ) | 6.637 ( 4.01 to 10.395 ) | 7.921 ( 4.987 to 12.282 ) | 0.6 (0.57 to 0.64) | 113.433 |
| Libya | Ischemic heart disease | Both | 346 ( 218 to 509 ) | 931 ( 598 to 1370 ) | 17.625 ( 11.121 to 26.635 ) | 17.838 ( 11.376 to 26.502 ) | 0.02 (-0.08 to 0.11) | 169.075 |
| Lithuania | Ischemic heart disease | Both | 993 ( 568 to 1509 ) | 1192 ( 663 to 1843 ) | 22.221 ( 12.751 to 33.515 ) | 18.347 ( 10.61 to 28.388 ) | -0.74 (-0.83 to -0.64) | 20.04 |
| Luxembourg | Ischemic heart disease | Both | 67 ( 37 to 103 ) | 142 ( 84 to 220 ) | 12.565 ( 7.11 to 18.951 ) | 12.829 ( 7.638 to 19.918 ) | -0.73 (-1.18 to -0.27) | 111.94 |
| Madagascar | Ischemic heart disease | Both | 320 ( 196 to 504 ) | 736 ( 444 to 1140 ) | 7.645 ( 4.689 to 12.173 ) | 8.525 ( 5.249 to 13.502 ) | 0.35 (0.32 to 0.38) | 130 |
| Malawi | Ischemic heart disease | Both | 238 ( 142 to 379 ) | 549 ( 336 to 881 ) | 7.754 ( 4.78 to 12.239 ) | 8.826 ( 5.496 to 14.114 ) | 0.35 (0.31 to 0.38) | 130.672 |
| Malaysia | Ischemic heart disease | Both | 1322 ( 821 to 1967 ) | 4551 ( 2898 to 6673 ) | 15.483 ( 9.371 to 23.433 ) | 17.623 ( 11.266 to 26.059 ) | 0.64 (0.57 to 0.71) | 244.251 |
| Maldives | Ischemic heart disease | Both | 9 ( 6 to 14 ) | 43 ( 28 to 63 ) | 12.116 ( 7.672 to 18.409 ) | 13.118 ( 8.393 to 19.793 ) | 0.3 (0.24 to 0.35) | 377.778 |
| Mali | Ischemic heart disease | Both | 184 ( 107 to 290 ) | 508 ( 310 to 775 ) | 5.804 ( 3.508 to 9.223 ) | 6.923 ( 4.29 to 10.655 ) | 0.58 (0.56 to 0.6) | 176.087 |
| Malta | Ischemic heart disease | Both | 58 ( 36 to 90 ) | 149 ( 90 to 226 ) | 14.303 ( 8.897 to 21.899 ) | 14.157 ( 8.58 to 21.624 ) | 0.4 (0.2 to 0.61) | 156.897 |
| Marshall Islands | Ischemic heart disease | Both | 1 ( 1 to 2 ) | 3 ( 2 to 5 ) | 10.125 ( 6.124 to 15.222 ) | 12.295 ( 7.745 to 18.054 ) | 0.67 (0.64 to 0.69) | 200 |
| Mauritania | Ischemic heart disease | Both | 63 ( 39 to 99 ) | 165 ( 102 to 257 ) | 7.108 ( 4.411 to 11.019 ) | 8.544 ( 5.373 to 13.49 ) | 0.62 (0.6 to 0.63) | 161.905 |
| Mauritius | Ischemic heart disease | Both | 76 ( 47 to 114 ) | 165 ( 100 to 246 ) | 11.874 ( 7.22 to 17.714 ) | 9.6 ( 5.905 to 14.19 ) | -0.79 (-0.95 to -0.63) | 117.105 |
| Mexico | Ischemic heart disease | Both | 5606 ( 3672 to 8089 ) | 18057 ( 11718 to 26167 ) | 14.084 ( 9.278 to 20.618 ) | 15.08 ( 9.743 to 21.68 ) | 0.22 (0.15 to 0.29) | 222.101 |
| Micronesia (Federated States of) | Ischemic heart disease | Both | 4 ( 3 to 6 ) | 7 ( 4 to 10 ) | 9.858 ( 6.116 to 14.789 ) | 11.793 ( 7.133 to 17.681 ) | 0.65 (0.62 to 0.68) | 75 |
| Monaco | Ischemic heart disease | Both | 8 ( 5 to 13 ) | 11 ( 7 to 17 ) | 11.078 ( 6.831 to 16.679 ) | 10.302 ( 6.356 to 15.644 ) | -0.53 (-0.6 to -0.46) | 37.5 |
| Mongolia | Ischemic heart disease | Both | 138 ( 74 to 223 ) | 248 ( 134 to 389 ) | 14.877 ( 8.003 to 23.596 ) | 14.352 ( 7.489 to 22.656 ) | -0.05 (-0.11 to 0.02) | 79.71 |
| Montenegro | Ischemic heart disease | Both | 82 ( 49 to 124 ) | 132 ( 77 to 202 ) | 13.964 ( 8.393 to 21.077 ) | 13.84 ( 8.272 to 20.97 ) | 0.01 (-0.02 to 0.03) | 60.976 |
| Morocco | Ischemic heart disease | Both | 2460 ( 1537 to 3642 ) | 5635 ( 3613 to 8282 ) | 17.278 ( 10.98 to 25.519 ) | 17.363 ( 11.16 to 25.748 ) | -0.08 (-0.12 to -0.04) | 129.065 |
| Mozambique | Ischemic heart disease | Both | 345 ( 206 to 549 ) | 703 ( 435 to 1098 ) | 7.147 ( 4.358 to 11.179 ) | 7.653 ( 4.776 to 12.207 ) | 0.13 (0.08 to 0.17) | 103.768 |
| Myanmar | Ischemic heart disease | Both | 1598 ( 976 to 2409 ) | 3980 ( 2494 to 5946 ) | 8.293 ( 5.163 to 12.312 ) | 9.422 ( 5.915 to 14.304 ) | 0.54 (0.5 to 0.59) | 149.061 |
| Namibia | Ischemic heart disease | Both | 44 ( 26 to 67 ) | 98 ( 59 to 152 ) | 8.569 ( 5.29 to 13.297 ) | 8.647 ( 5.281 to 13.478 ) | -0.01 (-0.03 to 0.01) | 122.727 |
| Nauru | Ischemic heart disease | Both | 0 ( 0 to 1 ) | 1 ( 0 to 1 ) | 12.533 ( 7.52 to 18.877 ) | 14.255 ( 8.883 to 21.163 ) | 0.4 (0.39 to 0.42) | #NUM! |
| Nepal | Ischemic heart disease | Both | 708 ( 447 to 1056 ) | 2220 ( 1387 to 3268 ) | 8.376 ( 5.332 to 12.683 ) | 10.312 ( 6.518 to 15.424 ) | 0.83 (0.72 to 0.93) | 213.559 |
| Netherlands | Ischemic heart disease | Both | 2771 ( 1742 to 4127 ) | 4231 ( 2529 to 6616 ) | 13.53 ( 8.535 to 20.225 ) | 11.372 ( 6.809 to 17.726 ) | -1.05 (-1.23 to -0.86) | 52.689 |
| New Zealand | Ischemic heart disease | Both | 657 ( 404 to 976 ) | 1329 ( 839 to 1958 ) | 16.571 ( 10.27 to 24.484 ) | 14.963 ( 9.515 to 22.004 ) | -0.94 (-1.11 to -0.78) | 102.283 |
| Nicaragua | Ischemic heart disease | Both | 194 ( 123 to 280 ) | 663 ( 410 to 997 ) | 13.163 ( 8.227 to 19.406 ) | 14.299 ( 8.983 to 21.493 ) | 0.28 (0.26 to 0.3) | 241.753 |
| Niger | Ischemic heart disease | Both | 125 ( 75 to 200 ) | 438 ( 268 to 683 ) | 5.617 ( 3.442 to 8.867 ) | 6.477 ( 4 to 10.268 ) | 0.42 (0.37 to 0.46) | 250.4 |
| Nigeria | Ischemic heart disease | Both | 1705 ( 1079 to 2596 ) | 4473 ( 2907 to 6784 ) | 4.416 ( 2.858 to 6.68 ) | 5.759 ( 3.736 to 8.774 ) | 0.95 (0.91 to 1) | 162.346 |
| Niue | Ischemic heart disease | Both | 0 ( 0 to 0 ) | 0 ( 0 to 0 ) | 12.326 ( 7.631 to 18.58 ) | 14.488 ( 9.137 to 22.215 ) | 0.52 (0.48 to 0.55) | #NUM! |
| North Macedonia | Ischemic heart disease | Both | 197 ( 119 to 296 ) | 313 ( 177 to 481 ) | 11.29 ( 6.744 to 16.865 ) | 10.233 ( 5.921 to 15.585 ) | -0.44 (-0.49 to -0.39) | 58.883 |
| Northern Mariana Islands | Ischemic heart disease | Both | 2 ( 1 to 3 ) | 7 ( 4 to 10 ) | 13.591 ( 8.39 to 20.683 ) | 15.642 ( 9.572 to 23.643 ) | 0.47 (0.45 to 0.48) | 250 |
| Norway | Ischemic heart disease | Both | 834 ( 431 to 1321 ) | 1286 ( 783 to 1917 ) | 12.007 ( 6.571 to 18.553 ) | 12.085 ( 7.538 to 18.15 ) | -0.22 (-0.38 to -0.05) | 54.197 |
| Oman | Ischemic heart disease | Both | 120 ( 79 to 173 ) | 362 ( 228 to 547 ) | 16.066 ( 10.283 to 23.482 ) | 15.426 ( 9.768 to 23.367 ) | -0.36 (-0.5 to -0.22) | 201.667 |
| Pakistan | Ischemic heart disease | Both | 5223 ( 3443 to 7492 ) | 12960 ( 8588 to 18851 ) | 9.915 ( 6.493 to 14.316 ) | 11.417 ( 7.393 to 16.623 ) | 0.65 (0.58 to 0.71) | 148.133 |
| Palau | Ischemic heart disease | Both | 1 ( 1 to 2 ) | 3 ( 2 to 5 ) | 15.101 ( 9.237 to 23.097 ) | 16.695 ( 10.48 to 24.911 ) | 0.3 (0.28 to 0.33) | 200 |
| Palestine | Ischemic heart disease | Both | 141 ( 88 to 207 ) | 445 ( 284 to 668 ) | 15.971 ( 9.993 to 23.778 ) | 17.024 ( 10.824 to 25.009 ) | 0.15 (0.13 to 0.17) | 215.603 |
| Panama | Ischemic heart disease | Both | 238 ( 147 to 363 ) | 617 ( 381 to 909 ) | 16.716 ( 10.314 to 25.485 ) | 13.76 ( 8.442 to 20.423 ) | -0.7 (-0.75 to -0.65) | 159.244 |
| Papua New Guinea | Ischemic heart disease | Both | 121 ( 76 to 182 ) | 418 ( 266 to 637 ) | 8.684 ( 5.496 to 13.086 ) | 10.5 ( 6.781 to 15.931 ) | 0.64 (0.58 to 0.7) | 245.455 |
| Paraguay | Ischemic heart disease | Both | 264 ( 162 to 383 ) | 665 ( 413 to 991 ) | 12.315 ( 7.577 to 17.962 ) | 11.805 ( 7.288 to 17.589 ) | -0.16 (-0.23 to -0.09) | 151.894 |
| Peru | Ischemic heart disease | Both | 1641 ( 1029 to 2431 ) | 5052 ( 3163 to 7594 ) | 14.075 ( 8.758 to 20.747 ) | 15.014 ( 9.411 to 22.642 ) | 0.07 (-0.03 to 0.18) | 207.861 |
| Philippines | Ischemic heart disease | Both | 2558 ( 1648 to 3693 ) | 7212 ( 4592 to 10630 ) | 9.812 ( 6.263 to 14.387 ) | 10.03 ( 6.349 to 14.889 ) | -0.05 (-0.09 to 0) | 181.939 |
| Poland | Ischemic heart disease | Both | 9527 ( 6152 to 13930 ) | 17439 ( 11629 to 25292 ) | 22.872 ( 14.914 to 33.547 ) | 22.844 ( 15.249 to 33.092 ) | -0.14 (-0.27 to -0.01) | 83.048 |
| Portugal | Ischemic heart disease | Both | 1548 ( 916 to 2350 ) | 2968 ( 1785 to 4497 ) | 11.474 ( 6.954 to 17.369 ) | 11.015 ( 6.646 to 16.822 ) | -0.86 (-1.02 to -0.69) | 91.731 |
| Puerto Rico | Ischemic heart disease | Both | 639 ( 387 to 986 ) | 1100 ( 671 to 1673 ) | 17.711 ( 10.801 to 27.231 ) | 13.62 ( 8.254 to 20.715 ) | -1.1 (-1.18 to -1.02) | 72.144 |
| Qatar | Ischemic heart disease | Both | 37 ( 23 to 54 ) | 316 ( 196 to 476 ) | 22.506 ( 14.342 to 32.878 ) | 21.961 ( 14.089 to 32.668 ) | -0.16 (-0.23 to -0.1) | 754.054 |
| Republic of Korea | Ischemic heart disease | Both | 1725 ( 1078 to 2656 ) | 8556 ( 5629 to 12943 ) | 6.204 ( 3.806 to 9.534 ) | 9.466 ( 6.24 to 14.244 ) | 1.38 (1.09 to 1.68) | 396 |
| Republic of Moldova | Ischemic heart disease | Both | 801 ( 477 to 1272 ) | 1052 ( 630 to 1580 ) | 20.955 ( 12.519 to 32.866 ) | 17.491 ( 10.434 to 26.226 ) | -0.87 (-0.96 to -0.78) | 31.336 |
| Romania | Ischemic heart disease | Both | 2730 ( 1542 to 4374 ) | 3631 ( 1940 to 5757 ) | 10.757 ( 6.194 to 16.777 ) | 9.277 ( 5.185 to 14.668 ) | -0.58 (-0.66 to -0.49) | 33.004 |
| Russian Federation | Ischemic heart disease | Both | 30320 ( 19248 to 44971 ) | 41392 ( 25667 to 61060 ) | 18.435 ( 11.762 to 27.287 ) | 16.901 ( 10.523 to 24.927 ) | -0.3 (-0.4 to -0.19) | 36.517 |
| Rwanda | Ischemic heart disease | Both | 141 ( 86 to 228 ) | 366 ( 227 to 569 ) | 6.316 ( 3.925 to 10.109 ) | 7.025 ( 4.403 to 11.038 ) | 0.28 (0.26 to 0.31) | 159.574 |
| Saint Kitts and Nevis | Ischemic heart disease | Both | 5 ( 3 to 8 ) | 5 ( 3 to 8 ) | 12.764 ( 7.723 to 19.405 ) | 9.353 ( 5.661 to 14.266 ) | -1.64 (-1.82 to -1.45) | 0 |
| Saint Lucia | Ischemic heart disease | Both | 8 ( 5 to 12 ) | 18 ( 11 to 28 ) | 9.501 ( 5.643 to 14.224 ) | 7.705 ( 4.7 to 11.906 ) | -1.76 (-2.02 to -1.51) | 125 |
| Saint Vincent and the Grenadines | Ischemic heart disease | Both | 8 ( 5 to 13 ) | 12 ( 7 to 19 ) | 11.718 ( 7.026 to 17.852 ) | 8.957 ( 5.259 to 14.185 ) | -1.31 (-1.48 to -1.14) | 50 |
| Samoa | Ischemic heart disease | Both | 8 ( 5 to 12 ) | 17 ( 11 to 26 ) | 10.95 ( 6.8 to 16.356 ) | 13.354 ( 8.503 to 20.305 ) | 0.7 (0.64 to 0.77) | 112.5 |
| San Marino | Ischemic heart disease | Both | 4 ( 2 to 6 ) | 8 ( 5 to 13 ) | 10.45 ( 6.282 to 15.942 ) | 9.766 ( 5.885 to 15.206 ) | -0.71 (-0.83 to -0.6) | 100 |
| Sao Tome and Principe | Ischemic heart disease | Both | 4 ( 2 to 6 ) | 7 ( 5 to 12 ) | 6.418 ( 3.881 to 10.185 ) | 7.723 ( 4.729 to 12.158 ) | 0.59 (0.54 to 0.64) | 75 |
| Saudi Arabia | Ischemic heart disease | Both | 808 ( 522 to 1182 ) | 3237 ( 2007 to 4836 ) | 12.929 ( 8.044 to 19.151 ) | 14.86 ( 9.222 to 21.816 ) | 0.47 (0.42 to 0.52) | 300.619 |
| Senegal | Ischemic heart disease | Both | 221 ( 137 to 339 ) | 589 ( 375 to 904 ) | 7.817 ( 4.962 to 12.179 ) | 8.554 ( 5.392 to 13.136 ) | 0.32 (0.24 to 0.39) | 166.516 |
| Serbia | Ischemic heart disease | Both | 967 ( 544 to 1514 ) | 1477 ( 779 to 2308 ) | 9.585 ( 5.361 to 14.981 ) | 8.749 ( 4.806 to 13.463 ) | -1.46 (-1.95 to -0.97) | 52.74 |
| Seychelles | Ischemic heart disease | Both | 3 ( 2 to 5 ) | 7 ( 4 to 10 ) | 5.318 ( 3.274 to 7.932 ) | 6.466 ( 3.897 to 9.852 ) | 0.46 (0.39 to 0.53) | 133.333 |
| Sierra Leone | Ischemic heart disease | Both | 133 ( 77 to 208 ) | 245 ( 151 to 371 ) | 7.17 ( 4.267 to 11.265 ) | 7.425 ( 4.579 to 11.371 ) | 0.06 (0.03 to 0.1) | 84.211 |
| Singapore | Ischemic heart disease | Both | 205 ( 125 to 314 ) | 887 ( 557 to 1365 ) | 9.455 ( 5.752 to 14.646 ) | 10.562 ( 6.614 to 16.15 ) | 0.26 (0.15 to 0.38) | 332.683 |
| Slovakia | Ischemic heart disease | Both | 1194 ( 713 to 1835 ) | 1637 ( 988 to 2541 ) | 20.626 ( 12.648 to 31.367 ) | 17.002 ( 10.421 to 26.113 ) | -0.01 (-0.19 to 0.18) | 37.102 |
| Slovenia | Ischemic heart disease | Both | 202 ( 112 to 312 ) | 429 ( 244 to 686 ) | 8.363 ( 4.69 to 12.814 ) | 9.045 ( 5.237 to 14.073 ) | -0.11 (-0.28 to 0.07) | 112.376 |
| Solomon Islands | Ischemic heart disease | Both | 14 ( 8 to 20 ) | 39 ( 24 to 58 ) | 13.025 ( 8.248 to 19.125 ) | 14.333 ( 8.906 to 20.944 ) | 0.23 (0.19 to 0.28) | 178.571 |
| Somalia | Ischemic heart disease | Both | 129 ( 79 to 204 ) | 370 ( 214 to 576 ) | 7.292 ( 4.444 to 11.569 ) | 7.788 ( 4.788 to 12.103 ) | 0.21 (0.18 to 0.23) | 186.822 |
| South Africa | Ischemic heart disease | Both | 1990 ( 1287 to 2946 ) | 4321 ( 2796 to 6489 ) | 10.623 ( 6.848 to 16.014 ) | 10.476 ( 6.826 to 15.751 ) | -0.03 (-0.14 to 0.08) | 117.136 |
| South Sudan | Ischemic heart disease | Both | 157 ( 94 to 249 ) | 256 ( 157 to 396 ) | 7.039 ( 4.298 to 11.312 ) | 8.374 ( 5.169 to 13.131 ) | 0.59 (0.55 to 0.63) | 63.057 |
| Spain | Ischemic heart disease | Both | 6031 ( 3525 to 9197 ) | 11857 ( 8035 to 17064 ) | 11.077 ( 6.581 to 16.736 ) | 11.3 ( 7.574 to 16.143 ) | -0.63 (-0.8 to -0.45) | 96.601 |
| Sri Lanka | Ischemic heart disease | Both | 853 ( 513 to 1291 ) | 3001 ( 1861 to 4439 ) | 8.967 ( 5.398 to 13.437 ) | 11.782 ( 7.411 to 17.515 ) | 1.12 (1.02 to 1.22) | 251.817 |
| Sudan | Ischemic heart disease | Both | 1446 ( 928 to 2174 ) | 3366 ( 2158 to 4983 ) | 15.837 ( 10.103 to 23.628 ) | 16.828 ( 10.889 to 25.359 ) | 0.08 (0.03 to 0.13) | 132.78 |
| Suriname | Ischemic heart disease | Both | 32 ( 20 to 49 ) | 69 ( 42 to 103 ) | 13.757 ( 8.425 to 21.166 ) | 11.702 ( 7.182 to 17.311 ) | -0.75 (-0.82 to -0.69) | 115.625 |
| Sweden | Ischemic heart disease | Both | 4469 ( 2756 to 6584 ) | 6227 ( 4004 to 9405 ) | 27.969 ( 17.967 to 40.973 ) | 25.803 ( 16.531 to 39.123 ) | -0.72 (-0.9 to -0.53) | 39.338 |
| Switzerland | Ischemic heart disease | Both | 1147 ( 622 to 1794 ) | 1784 ( 1026 to 2712 ) | 10.625 ( 5.875 to 16.439 ) | 8.993 ( 5.23 to 13.83 ) | -0.34 (-0.85 to 0.17) | 55.536 |
| Syrian Arab Republic | Ischemic heart disease | Both | 1103 ( 716 to 1581 ) | 2680 ( 1719 to 3956 ) | 20.524 ( 13.012 to 30.186 ) | 22.329 ( 14.172 to 32.5 ) | 0.4 (0.3 to 0.49) | 142.974 |
| Taiwan (Province of China) | Ischemic heart disease | Both | 1322 ( 796 to 2048 ) | 4372 ( 2689 to 6730 ) | 9.648 ( 5.746 to 14.663 ) | 10.147 ( 6.273 to 15.757 ) | 0.11 (0.04 to 0.17) | 230.711 |
| Tajikistan | Ischemic heart disease | Both | 381 ( 231 to 579 ) | 727 ( 425 to 1113 ) | 14.632 ( 8.52 to 22.62 ) | 15.307 ( 8.785 to 23.553 ) | 0.09 (0.02 to 0.15) | 90.814 |
| Thailand | Ischemic heart disease | Both | 4226 ( 2680 to 6259 ) | 14885 ( 9482 to 22460 ) | 13.756 ( 8.405 to 20.5 ) | 13.932 ( 8.841 to 21.107 ) | 0.05 (-0.01 to 0.11) | 252.224 |
| Timor-Leste | Ischemic heart disease | Both | 19 ( 12 to 28 ) | 79 ( 48 to 117 ) | 7.93 ( 4.92 to 11.949 ) | 10.163 ( 6.369 to 14.849 ) | 1.03 (0.97 to 1.1) | 315.789 |
| Togo | Ischemic heart disease | Both | 66 ( 40 to 104 ) | 220 ( 135 to 340 ) | 6.449 ( 3.951 to 10.118 ) | 7.129 ( 4.417 to 11.42 ) | 0.29 (0.26 to 0.33) | 233.333 |
| Tokelau | Ischemic heart disease | Both | 0 ( 0 to 0 ) | 0 ( 0 to 0 ) | 11.526 ( 6.983 to 17.182 ) | 14.346 ( 9.043 to 21.447 ) | 0.73 (0.7 to 0.76) | #NUM! |
| Tonga | Ischemic heart disease | Both | 7 ( 4 to 10 ) | 12 ( 7 to 18 ) | 14.176 ( 8.753 to 21.138 ) | 15.551 ( 9.53 to 23.152 ) | 0.31 (0.29 to 0.33) | 71.429 |
| Trinidad and Tobago | Ischemic heart disease | Both | 116 ( 68 to 171 ) | 263 ( 162 to 399 ) | 14.419 ( 8.657 to 21.435 ) | 13.968 ( 8.649 to 21.265 ) | -0.28 (-0.36 to -0.21) | 126.724 |
| Tunisia | Ischemic heart disease | Both | 847 ( 528 to 1258 ) | 2135 ( 1327 to 3141 ) | 17.662 ( 10.729 to 26.315 ) | 16.806 ( 10.595 to 24.856 ) | -0.28 (-0.35 to -0.22) | 152.066 |
| Turkey | Ischemic heart disease | Both | 5931 ( 3863 to 8802 ) | 16872 ( 10500 to 24921 ) | 17.408 ( 11.289 to 25.919 ) | 18.544 ( 11.599 to 27.498 ) | 0.17 (0.11 to 0.23) | 184.471 |
| Turkmenistan | Ischemic heart disease | Both | 289 ( 176 to 446 ) | 564 ( 337 to 876 ) | 17.981 ( 10.73 to 27.632 ) | 17.17 ( 10.214 to 26.999 ) | -0.15 (-0.23 to -0.07) | 95.156 |
| Tuvalu | Ischemic heart disease | Both | 1 ( 0 to 1 ) | 1 ( 1 to 2 ) | 10.489 ( 6.348 to 15.465 ) | 13.416 ( 8.373 to 20.066 ) | 0.84 (0.78 to 0.89) | 0 |
| Uganda | Ischemic heart disease | Both | 404 ( 245 to 636 ) | 989 ( 606 to 1503 ) | 7.411 ( 4.575 to 11.72 ) | 7.874 ( 4.83 to 12.274 ) | 0.09 (0.03 to 0.14) | 144.802 |
| Ukraine | Ischemic heart disease | Both | 13259 ( 8232 to 19723 ) | 15755 ( 9707 to 23584 ) | 19.48 ( 12.457 to 28.902 ) | 19.42 ( 12.137 to 28.969 ) | 0.12 (0.05 to 0.19) | 18.825 |
| United Arab Emirates | Ischemic heart disease | Both | 105 ( 63 to 159 ) | 936 ( 554 to 1427 ) | 18.338 ( 11.888 to 27.26 ) | 17.52 ( 10.844 to 26.125 ) | -0.14 (-0.21 to -0.08) | 791.429 |
| United Kingdom | Ischemic heart disease | Both | 10257 ( 6077 to 15692 ) | 11243 ( 7001 to 16677 ) | 11.001 ( 6.715 to 16.558 ) | 8.072 ( 5.103 to 11.928 ) | -1.59 (-1.75 to -1.43) | 9.613 |
| United Republic of Tanzania | Ischemic heart disease | Both | 640 ( 366 to 996 ) | 2043 ( 1276 to 3078 ) | 7.086 ( 4.235 to 11.059 ) | 9.25 ( 5.723 to 14.348 ) | 1.22 (1.1 to 1.34) | 219.219 |
| United States of America | Ischemic heart disease | Both | 59105 ( 36088 to 89666 ) | 93042 ( 60525 to 134515 ) | 18.03 ( 11.197 to 27.051 ) | 15.305 ( 9.952 to 22.101 ) | -0.94 (-1.04 to -0.84) | 57.418 |
| United States Virgin Islands | Ischemic heart disease | Both | 11 ( 7 to 16 ) | 27 ( 16 to 42 ) | 14.8 ( 8.834 to 22.127 ) | 14.314 ( 8.77 to 21.774 ) | -0.23 (-0.27 to -0.19) | 145.455 |
| Uruguay | Ischemic heart disease | Both | 397 ( 234 to 603 ) | 439 ( 252 to 682 ) | 10.348 ( 6.195 to 15.676 ) | 7.589 ( 4.468 to 11.806 ) | -1.14 (-1.23 to -1.05) | 10.579 |
| Uzbekistan | Ischemic heart disease | Both | 2088 ( 1229 to 3210 ) | 3791 ( 2208 to 5866 ) | 18.999 ( 11.071 to 29.578 ) | 17.364 ( 9.762 to 27.006 ) | -0.23 (-0.31 to -0.16) | 81.561 |
| Vanuatu | Ischemic heart disease | Both | 6 ( 4 to 10 ) | 19 ( 12 to 29 ) | 12.781 ( 7.89 to 19.455 ) | 14.019 ( 8.738 to 20.899 ) | 0.28 (0.26 to 0.29) | 216.667 |
| Venezuela (Bolivarian Republic of) | Ischemic heart disease | Both | 1208 ( 760 to 1785 ) | 4195 ( 2680 to 6361 ) | 12.997 ( 8.133 to 19.292 ) | 14.677 ( 9.338 to 22.152 ) | 0.44 (0.42 to 0.47) | 247.268 |
| Viet Nam | Ischemic heart disease | Both | 2177 ( 1362 to 3272 ) | 7128 ( 4410 to 10493 ) | 5.819 ( 3.657 to 8.72 ) | 8.129 ( 4.956 to 12.002 ) | 1.39 (1.29 to 1.5) | 227.423 |
| Yemen | Ischemic heart disease | Both | 655 ( 404 to 977 ) | 2077 ( 1321 to 3048 ) | 13.603 ( 8.474 to 20.249 ) | 14.48 ( 9.313 to 21.33 ) | 0.2 (0.17 to 0.23) | 217.099 |
| Zambia | Ischemic heart disease | Both | 170 ( 105 to 272 ) | 448 ( 273 to 709 ) | 7.508 ( 4.532 to 11.965 ) | 7.923 ( 4.857 to 12.643 ) | 0.11 (0.08 to 0.13) | 163.529 |
| Zimbabwe | Ischemic heart disease | Both | 256 ( 154 to 408 ) | 457 ( 270 to 706 ) | 7.81 ( 4.779 to 12.035 ) | 8.718 ( 5.279 to 13.45 ) | 0.34 (0.29 to 0.39) | 78.516 |

**Table S7 The Prevalence of SHF due to RHD in 1990 and 2021 across countries and territories.**

| Locations | cause | sex | 1990 Prevalence cases(95% UI) | 2021 Prevalence cases(95% UI) | 1990 ASPR(95% UI) | 2021 ASPR(95% UI) | 1990-2021 EAPC（95%CI） | 1990-2021 Prevalence cases changes |
| --- | --- | --- | --- | --- | --- | --- | --- | --- |
| Afghanistan | Rheumatic heart disease | Both | 923 ( 702 to 1202 ) | 2216 ( 1649 to 2914 ) | 8.155 ( 6.504 to 10.23 ) | 5.986 ( 4.696 to 7.6 ) | -1.26 (-1.35 to -1.17) | 140.087 |
| Albania | Rheumatic heart disease | Both | 249 ( 202 to 304 ) | 112 ( 87 to 141 ) | 8.545 ( 6.858 to 10.413 ) | 3.513 ( 2.841 to 4.341 ) | -3.24 (-3.45 to -3.04) | -55.02 |
| Algeria | Rheumatic heart disease | Both | 2026 ( 1541 to 2684 ) | 1796 ( 1433 to 2279 ) | 6.673 ( 5.241 to 8.545 ) | 4.145 ( 3.331 to 5.205 ) | -1.62 (-1.67 to -1.57) | -11.352 |
| American Samoa | Rheumatic heart disease | Both | 5 ( 4 to 6 ) | 3 ( 3 to 4 ) | 10.198 ( 8.032 to 12.818 ) | 6.912 ( 5.479 to 8.691 ) | -1.4 (-1.53 to -1.26) | -40 |
| Andorra | Rheumatic heart disease | Both | 1 ( 1 to 1 ) | 2 ( 1 to 2 ) | 1.871 ( 1.482 to 2.326 ) | 1.496 ( 1.175 to 1.876 ) | -0.64 (-0.68 to -0.6) | 100 |
| Angola | Rheumatic heart disease | Both | 751 ( 517 to 1071 ) | 1702 ( 1136 to 2449 ) | 6.152 ( 4.3 to 8.588 ) | 4.74 ( 3.26 to 6.595 ) | -1.01 (-1.08 to -0.94) | 126.631 |
| Antigua and Barbuda | Rheumatic heart disease | Both | 4 ( 3 to 5 ) | 2 ( 2 to 3 ) | 5.891 ( 4.663 to 7.513 ) | 2.527 ( 1.984 to 3.187 ) | -3.01 (-3.14 to -2.89) | -50 |
| Argentina | Rheumatic heart disease | Both | 1632 ( 1273 to 2077 ) | 978 ( 741 to 1256 ) | 5.035 ( 3.945 to 6.369 ) | 1.853 ( 1.43 to 2.32 ) | -3.84 (-4.24 to -3.44) | -40.074 |
| Armenia | Rheumatic heart disease | Both | 311 ( 244 to 391 ) | 153 ( 124 to 188 ) | 9.218 ( 7.364 to 11.468 ) | 4.789 ( 3.875 to 5.906 ) | -1.95 (-2.12 to -1.78) | -50.804 |
| Australia | Rheumatic heart disease | Both | 945 ( 762 to 1148 ) | 2062 ( 1645 to 2509 ) | 5.18 ( 4.205 to 6.173 ) | 5.504 ( 4.517 to 6.599 ) | 0.34 (0.17 to 0.5) | 118.201 |
| Austria | Rheumatic heart disease | Both | 457 ( 346 to 577 ) | 565 ( 425 to 716 ) | 3.974 ( 3.076 to 4.925 ) | 3.281 ( 2.564 to 4.009 ) | -1.45 (-1.79 to -1.11) | 23.632 |
| Azerbaijan | Rheumatic heart disease | Both | 218 ( 172 to 279 ) | 216 ( 169 to 274 ) | 2.974 ( 2.399 to 3.722 ) | 2.278 ( 1.772 to 2.918 ) | -1.1 (-1.3 to -0.9) | -0.917 |
| Bahamas | Rheumatic heart disease | Both | 9 ( 7 to 12 ) | 5 ( 4 to 7 ) | 3.116 ( 2.412 to 4.097 ) | 1.471 ( 1.113 to 1.923 ) | -2.95 (-3.12 to -2.78) | -44.444 |
| Bahrain | Rheumatic heart disease | Both | 22 ( 17 to 27 ) | 31 ( 25 to 38 ) | 4.595 ( 3.735 to 5.595 ) | 2.464 ( 1.99 to 3.004 ) | -2.54 (-2.76 to -2.31) | 40.909 |
| Bangladesh | Rheumatic heart disease | Both | 20752 ( 16518 to 26049 ) | 31686 ( 25749 to 38495 ) | 21.856 ( 17.881 to 26.62 ) | 19.777 ( 16.25 to 23.927 ) | -0.32 (-0.35 to -0.29) | 52.689 |
| Barbados | Rheumatic heart disease | Both | 14 ( 11 to 17 ) | 8 ( 6 to 10 ) | 5.223 ( 4.102 to 6.567 ) | 2.614 ( 2.061 to 3.253 ) | -2.41 (-2.53 to -2.3) | -42.857 |
| Belarus | Rheumatic heart disease | Both | 378 ( 296 to 473 ) | 219 ( 164 to 286 ) | 3.338 ( 2.659 to 4.135 ) | 1.814 ( 1.449 to 2.267 ) | -2.16 (-2.28 to -2.04) | -42.063 |
| Belgium | Rheumatic heart disease | Both | 352 ( 262 to 462 ) | 669 ( 536 to 867 ) | 2.557 ( 1.97 to 3.224 ) | 3.208 ( 2.62 to 4.01 ) | 1.43 (1.24 to 1.61) | 90.057 |
| Belize | Rheumatic heart disease | Both | 13 ( 9 to 17 ) | 10 ( 8 to 14 ) | 5.555 ( 4.297 to 7.132 ) | 2.353 ( 1.812 to 3.02 ) | -2.97 (-3.03 to -2.91) | -23.077 |
| Benin | Rheumatic heart disease | Both | 391 ( 277 to 538 ) | 778 ( 540 to 1094 ) | 7.041 ( 5.071 to 9.67 ) | 5.28 ( 3.74 to 7.197 ) | -0.97 (-1.09 to -0.85) | 98.977 |
| Bermuda | Rheumatic heart disease | Both | 3 ( 2 to 3 ) | 2 ( 1 to 2 ) | 4.238 ( 3.367 to 5.223 ) | 2.674 ( 2.174 to 3.263 ) | -1.82 (-1.92 to -1.72) | -33.333 |
| Bhutan | Rheumatic heart disease | Both | 143 ( 113 to 180 ) | 146 ( 119 to 179 ) | 25.658 ( 21.394 to 30.947 ) | 20.267 ( 16.845 to 24.611 ) | -0.83 (-0.85 to -0.8) | 2.098 |
| Bolivia (Plurinational State of) | Rheumatic heart disease | Both | 327 ( 263 to 410 ) | 385 ( 305 to 473 ) | 6.042 ( 4.908 to 7.403 ) | 3.594 ( 2.878 to 4.42 ) | -1.84 (-1.9 to -1.77) | 17.737 |
| Bosnia and Herzegovina | Rheumatic heart disease | Both | 117 ( 95 to 147 ) | 53 ( 39 to 68 ) | 2.797 ( 2.231 to 3.553 ) | 1.184 ( 0.925 to 1.454 ) | -3.13 (-3.33 to -2.93) | -54.701 |
| Botswana | Rheumatic heart disease | Both | 159 ( 112 to 222 ) | 166 ( 118 to 229 ) | 9.422 ( 6.751 to 12.844 ) | 6.88 ( 5.017 to 9.38 ) | -1.02 (-1.07 to -0.98) | 4.403 |
| Brazil | Rheumatic heart disease | Both | 10346 ( 8018 to 13345 ) | 7981 ( 6162 to 10081 ) | 6.471 ( 5.133 to 8.136 ) | 3.684 ( 2.839 to 4.639 ) | -2.08 (-2.15 to -2) | -22.859 |
| Brunei Darussalam | Rheumatic heart disease | Both | 4 ( 3 to 5 ) | 6 ( 4 to 7 ) | 1.968 ( 1.478 to 2.483 ) | 1.538 ( 1.17 to 1.968 ) | -0.93 (-1.01 to -0.85) | 50 |
| Bulgaria | Rheumatic heart disease | Both | 436 ( 343 to 537 ) | 129 ( 99 to 163 ) | 4.236 ( 3.391 to 5.184 ) | 1.667 ( 1.341 to 2.053 ) | -3.43 (-3.89 to -2.96) | -70.413 |
| Burkina Faso | Rheumatic heart disease | Both | 771 ( 545 to 1086 ) | 1389 ( 962 to 1926 ) | 7.04 ( 5.073 to 9.771 ) | 5.58 ( 3.888 to 7.616 ) | -0.79 (-0.88 to -0.71) | 80.156 |
| Burundi | Rheumatic heart disease | Both | 345 ( 235 to 503 ) | 522 ( 344 to 754 ) | 5.1 ( 3.62 to 7.139 ) | 3.569 ( 2.516 to 4.943 ) | -1.29 (-1.33 to -1.25) | 51.304 |
| Cabo Verde | Rheumatic heart disease | Both | 49 ( 36 to 69 ) | 33 ( 24 to 44 ) | 11.317 ( 8.168 to 15.482 ) | 5.931 ( 4.273 to 7.974 ) | -2.04 (-2.28 to -1.8) | -32.653 |
| Cambodia | Rheumatic heart disease | Both | 736 ( 559 to 962 ) | 776 ( 584 to 1017 ) | 6.478 ( 5.066 to 8.163 ) | 4.473 ( 3.453 to 5.818 ) | -1.38 (-1.44 to -1.32) | 5.435 |
| Cameroon | Rheumatic heart disease | Both | 611 ( 420 to 864 ) | 1335 ( 912 to 1882 ) | 5.156 ( 3.632 to 7.083 ) | 3.844 ( 2.72 to 5.222 ) | -1.23 (-1.33 to -1.14) | 118.494 |
| Canada | Rheumatic heart disease | Both | 1829 ( 1438 to 2305 ) | 3432 ( 2617 to 4405 ) | 5.903 ( 4.646 to 7.442 ) | 5.443 ( 4.282 to 6.79 ) | -0.16 (-0.32 to 0) | 87.644 |
| Central African Republic | Rheumatic heart disease | Both | 192 ( 129 to 276 ) | 322 ( 216 to 470 ) | 5.918 ( 4.103 to 8.134 ) | 5.141 ( 3.569 to 7.185 ) | -0.55 (-0.59 to -0.51) | 67.708 |
| Chad | Rheumatic heart disease | Both | 544 ( 385 to 761 ) | 1235 ( 873 to 1724 ) | 7.613 ( 5.524 to 10.339 ) | 6.003 ( 4.25 to 8.228 ) | -0.83 (-0.91 to -0.76) | 127.022 |
| Chile | Rheumatic heart disease | Both | 706 ( 567 to 856 ) | 452 ( 356 to 555 ) | 5.86 ( 4.737 to 7.139 ) | 2.117 ( 1.687 to 2.568 ) | -3.71 (-3.89 to -3.53) | -35.977 |
| China | Rheumatic heart disease | Both | 102309 ( 82022 to 125517 ) | 151923 ( 110003 to 202423 ) | 11.221 ( 8.941 to 13.59 ) | 7.649 ( 5.804 to 9.942 ) | -1.55 (-1.66 to -1.44) | 48.494 |
| Colombia | Rheumatic heart disease | Both | 1169 ( 923 to 1441 ) | 469 ( 378 to 579 ) | 3.767 ( 3.027 to 4.555 ) | 0.929 ( 0.746 to 1.142 ) | -5.18 (-5.68 to -4.68) | -59.88 |
| Comoros | Rheumatic heart disease | Both | 28 ( 19 to 40 ) | 25 ( 17 to 36 ) | 5.061 ( 3.58 to 6.918 ) | 3.316 ( 2.318 to 4.551 ) | -1.48 (-1.54 to -1.43) | -10.714 |
| Congo | Rheumatic heart disease | Both | 173 ( 118 to 251 ) | 252 ( 169 to 357 ) | 6.019 ( 4.257 to 8.433 ) | 4.419 ( 3.089 to 6.078 ) | -1.16 (-1.23 to -1.09) | 45.665 |
| Cook Islands | Rheumatic heart disease | Both | 1 ( 1 to 2 ) | 1 ( 1 to 1 ) | 6.856 ( 5.552 to 8.354 ) | 6.206 ( 4.898 to 7.756 ) | -0.47 (-0.54 to -0.4) | 0 |
| Costa Rica | Rheumatic heart disease | Both | 199 ( 159 to 248 ) | 108 ( 88 to 134 ) | 7.201 ( 5.826 to 8.823 ) | 2.094 ( 1.713 to 2.577 ) | -4.28 (-4.45 to -4.11) | -45.729 |
| Coted'Ivoire | Rheumatic heart disease | Both | 982 ( 681 to 1382 ) | 1565 ( 1077 to 2172 ) | 6.953 ( 4.903 to 9.524 ) | 5.216 ( 3.703 to 7.1 ) | -0.91 (-0.99 to -0.83) | 59.369 |
| Croatia | Rheumatic heart disease | Both | 192 ( 146 to 246 ) | 115 ( 84 to 156 ) | 3.444 ( 2.625 to 4.413 ) | 1.717 ( 1.333 to 2.206 ) | -3.12 (-3.95 to -2.27) | -40.104 |
| Cuba | Rheumatic heart disease | Both | 974 ( 766 to 1248 ) | 559 ( 454 to 673 ) | 8.912 ( 7.051 to 11.337 ) | 5.39 ( 4.325 to 6.664 ) | -1.86 (-2.07 to -1.64) | -42.608 |
| Cyprus | Rheumatic heart disease | Both | 31 ( 23 to 40 ) | 61 ( 45 to 81 ) | 3.764 ( 2.825 to 4.858 ) | 3.226 ( 2.462 to 4.185 ) | -0.84 (-1.01 to -0.68) | 96.774 |
| Czechia | Rheumatic heart disease | Both | 694 ( 531 to 890 ) | 590 ( 428 to 793 ) | 5.517 ( 4.31 to 6.98 ) | 3.468 ( 2.699 to 4.334 ) | -1.51 (-1.85 to -1.16) | -14.986 |
| Democratic People's Republic of Korea | Rheumatic heart disease | Both | 1441 ( 1196 to 1729 ) | 1718 ( 1380 to 2162 ) | 8.82 ( 7.268 to 10.689 ) | 5.766 ( 4.684 to 7.206 ) | -1.53 (-1.58 to -1.49) | 19.223 |
| Democratic Republic of the Congo | Rheumatic heart disease | Both | 2841 ( 1931 to 4115 ) | 4456 ( 2943 to 6503 ) | 6.229 ( 4.347 to 8.852 ) | 4.461 ( 3.075 to 6.275 ) | -1.21 (-1.28 to -1.13) | 56.846 |
| Denmark | Rheumatic heart disease | Both | 104 ( 80 to 134 ) | 276 ( 203 to 367 ) | 1.536 ( 1.212 to 1.916 ) | 2.642 ( 2.044 to 3.384 ) | 2.16 (1.93 to 2.39) | 165.385 |
| Djibouti | Rheumatic heart disease | Both | 24 ( 16 to 35 ) | 43 ( 29 to 61 ) | 4.876 ( 3.471 to 6.796 ) | 3.417 ( 2.35 to 4.698 ) | -1.32 (-1.38 to -1.26) | 79.167 |
| Dominica | Rheumatic heart disease | Both | 4 ( 3 to 5 ) | 2 ( 2 to 2 ) | 5.209 ( 4.133 to 6.66 ) | 2.922 ( 2.294 to 3.731 ) | -2.07 (-2.18 to -1.96) | -50 |
| Dominican Republic | Rheumatic heart disease | Both | 645 ( 482 to 844 ) | 548 ( 414 to 711 ) | 7.769 ( 5.982 to 10.032 ) | 5.052 ( 3.846 to 6.494 ) | -1.69 (-1.77 to -1.61) | -15.039 |
| Ecuador | Rheumatic heart disease | Both | 460 ( 371 to 585 ) | 381 ( 304 to 470 ) | 5.156 ( 4.162 to 6.374 ) | 2.17 ( 1.734 to 2.688 ) | -2.72 (-2.87 to -2.57) | -17.174 |
| Egypt | Rheumatic heart disease | Both | 6543 ( 5097 to 8690 ) | 6859 ( 5327 to 8946 ) | 11.053 ( 8.808 to 14.164 ) | 6.375 ( 5.001 to 8.139 ) | -2.08 (-2.17 to -1.99) | 4.83 |
| El Salvador | Rheumatic heart disease | Both | 125 ( 100 to 157 ) | 59 ( 47 to 71 ) | 2.586 ( 2.076 to 3.196 ) | 0.912 ( 0.733 to 1.107 ) | -3.79 (-3.98 to -3.61) | -52.8 |
| Equatorial Guinea | Rheumatic heart disease | Both | 32 ( 22 to 45 ) | 61 ( 39 to 88 ) | 6.321 ( 4.462 to 8.735 ) | 3.651 ( 2.485 to 5.027 ) | -2.31 (-2.51 to -2.12) | 90.625 |
| Eritrea | Rheumatic heart disease | Both | 236 ( 158 to 347 ) | 259 ( 172 to 365 ) | 5.562 ( 3.929 to 7.829 ) | 3.674 ( 2.586 to 5.05 ) | -1.41 (-1.49 to -1.34) | 9.746 |
| Estonia | Rheumatic heart disease | Both | 81 ( 63 to 101 ) | 19 ( 14 to 24 ) | 4.594 ( 3.649 to 5.762 ) | 1.07 ( 0.84 to 1.348 ) | -5.62 (-5.93 to -5.31) | -76.543 |
| Eswatini | Rheumatic heart disease | Both | 99 ( 69 to 140 ) | 97 ( 67 to 134 ) | 9.33 ( 6.682 to 12.812 ) | 7.43 ( 5.362 to 10.155 ) | -0.78 (-0.82 to -0.73) | -2.02 |
| Ethiopia | Rheumatic heart disease | Both | 1191 ( 806 to 1751 ) | 1870 ( 1260 to 2784 ) | 2.295 ( 1.648 to 3.202 ) | 1.91 ( 1.357 to 2.641 ) | -0.51 (-0.59 to -0.44) | 57.011 |
| Fiji | Rheumatic heart disease | Both | 102 ( 79 to 131 ) | 94 ( 74 to 120 ) | 13.557 ( 10.809 to 16.753 ) | 10.688 ( 8.447 to 13.348 ) | -0.91 (-0.95 to -0.86) | -7.843 |
| Finland | Rheumatic heart disease | Both | 124 ( 89 to 161 ) | 183 ( 134 to 247 ) | 1.964 ( 1.497 to 2.513 ) | 1.859 ( 1.447 to 2.379 ) | -0.32 (-0.6 to -0.04) | 47.581 |
| France | Rheumatic heart disease | Both | 7078 ( 5524 to 9074 ) | 11432 ( 8977 to 14076 ) | 8.594 ( 6.74 to 10.89 ) | 8.269 ( 6.62 to 10.014 ) | 0.1 (-0.23 to 0.42) | 61.515 |
| Gabon | Rheumatic heart disease | Both | 62 ( 42 to 88 ) | 80 ( 54 to 113 ) | 5.563 ( 3.869 to 7.653 ) | 4.186 ( 2.889 to 5.856 ) | -1.03 (-1.06 to -1) | 29.032 |
| Gambia | Rheumatic heart disease | Both | 88 ( 63 to 125 ) | 150 ( 106 to 209 ) | 7.555 ( 5.444 to 10.406 ) | 5.53 ( 3.962 to 7.459 ) | -1 (-1.13 to -0.87) | 70.455 |
| Georgia | Rheumatic heart disease | Both | 753 ( 600 to 957 ) | 216 ( 161 to 272 ) | 14.313 ( 11.285 to 18.485 ) | 5.652 ( 4.449 to 6.967 ) | -2.97 (-3.6 to -2.33) | -71.315 |
| Germany | Rheumatic heart disease | Both | 3860 ( 2912 to 5089 ) | 8739 ( 6270 to 11685 ) | 3.399 ( 2.64 to 4.335 ) | 4.657 ( 3.507 to 5.977 ) | 1.5 (1.15 to 1.84) | 126.399 |
| Ghana | Rheumatic heart disease | Both | 1281 ( 984 to 1662 ) | 1534 ( 1119 to 2062 ) | 8.017 ( 6.252 to 10.516 ) | 4.634 ( 3.414 to 6.147 ) | -2.21 (-2.41 to -2.02) | 19.75 |
| Greece | Rheumatic heart disease | Both | 132 ( 100 to 170 ) | 154 ( 113 to 206 ) | 0.984 ( 0.782 to 1.221 ) | 0.848 ( 0.676 to 1.052 ) | -1.14 (-1.66 to -0.62) | 16.667 |
| Greenland | Rheumatic heart disease | Both | 1 ( 1 to 2 ) | 1 ( 1 to 2 ) | 3.75 ( 2.686 to 4.917 ) | 2.211 ( 1.582 to 2.895 ) | -2.17 (-2.37 to -1.98) | 0 |
| Grenada | Rheumatic heart disease | Both | 8 ( 6 to 11 ) | 5 ( 4 to 6 ) | 8.668 ( 6.828 to 10.981 ) | 4.601 ( 3.71 to 5.792 ) | -2.31 (-2.41 to -2.22) | -37.5 |
| Guam | Rheumatic heart disease | Both | 10 ( 7 to 13 ) | 10 ( 8 to 13 ) | 7.407 ( 5.615 to 9.684 ) | 6.874 ( 5.293 to 9.037 ) | -0.54 (-0.66 to -0.43) | 0 |
| Guatemala | Rheumatic heart disease | Both | 228 ( 175 to 302 ) | 79 ( 62 to 98 ) | 2.72 ( 2.168 to 3.413 ) | 0.537 ( 0.426 to 0.665 ) | -5.81 (-6.14 to -5.49) | -65.351 |
| Guinea | Rheumatic heart disease | Both | 497 ( 358 to 688 ) | 849 ( 585 to 1178 ) | 7.413 ( 5.213 to 10.236 ) | 5.677 ( 4.008 to 7.657 ) | -0.86 (-0.94 to -0.78) | 70.825 |
| Guinea-Bissau | Rheumatic heart disease | Both | 89 ( 62 to 125 ) | 133 ( 91 to 184 ) | 7.414 ( 5.338 to 10.087 ) | 5.724 ( 4.045 to 7.768 ) | -0.82 (-0.9 to -0.73) | 49.438 |
| Guyana | Rheumatic heart disease | Both | 55 ( 40 to 74 ) | 19 ( 15 to 26 ) | 5.926 ( 4.487 to 7.923 ) | 2.505 ( 1.922 to 3.279 ) | -3.02 (-3.19 to -2.85) | -65.455 |
| Haiti | Rheumatic heart disease | Both | 715 ( 550 to 943 ) | 1012 ( 782 to 1294 ) | 10.129 ( 8.057 to 12.987 ) | 7.477 ( 5.908 to 9.427 ) | -1.06 (-1.09 to -1.03) | 41.538 |
| Honduras | Rheumatic heart disease | Both | 40 ( 31 to 50 ) | 60 ( 49 to 75 ) | 1.081 ( 0.869 to 1.336 ) | 0.694 ( 0.56 to 0.854 ) | -1.5 (-1.53 to -1.46) | 50 |
| Hungary | Rheumatic heart disease | Both | 502 ( 373 to 658 ) | 210 ( 149 to 281 ) | 3.909 ( 2.975 to 4.959 ) | 1.531 ( 1.206 to 1.908 ) | -3.28 (-3.73 to -2.83) | -58.167 |
| Iceland | Rheumatic heart disease | Both | 3 ( 2 to 3 ) | 7 ( 5 to 8 ) | 0.935 ( 0.727 to 1.188 ) | 1.438 ( 1.16 to 1.786 ) | 1.05 (0.83 to 1.26) | 133.333 |
| India | Rheumatic heart disease | Both | 189947 ( 156052 to 232120 ) | 290436 ( 237373 to 357768 ) | 23.871 ( 19.894 to 28.714 ) | 20.988 ( 17.246 to 25.856 ) | -0.46 (-0.49 to -0.43) | 52.904 |
| Indonesia | Rheumatic heart disease | Both | 9870 ( 8117 to 12132 ) | 8415 ( 6926 to 10214 ) | 5.035 ( 4.185 to 6.125 ) | 3.219 ( 2.647 to 3.913 ) | -1.89 (-2.02 to -1.76) | -14.742 |
| Iran (Islamic Republic of) | Rheumatic heart disease | Both | 5271 ( 4236 to 6592 ) | 4535 ( 3633 to 5591 ) | 8.142 ( 6.65 to 10.002 ) | 5.83 ( 4.669 to 7.244 ) | -1.31 (-1.52 to -1.1) | -13.963 |
| Iraq | Rheumatic heart disease | Both | 1516 ( 1180 to 1952 ) | 1960 ( 1514 to 2508 ) | 7.47 ( 5.979 to 9.247 ) | 4.716 ( 3.735 to 5.855 ) | -1.62 (-1.68 to -1.57) | 29.288 |
| Ireland | Rheumatic heart disease | Both | 72 ( 55 to 92 ) | 173 ( 132 to 223 ) | 1.797 ( 1.397 to 2.271 ) | 2.444 ( 1.917 to 3.139 ) | 1.39 (1.25 to 1.52) | 140.278 |
| Israel | Rheumatic heart disease | Both | 141 ( 111 to 181 ) | 576 ( 437 to 777 ) | 2.849 ( 2.25 to 3.595 ) | 4.768 ( 3.717 to 6.342 ) | 2.04 (1.82 to 2.26) | 308.511 |
| Italy | Rheumatic heart disease | Both | 4303 ( 3180 to 5691 ) | 6776 ( 4705 to 9501 ) | 5.843 ( 4.594 to 7.43 ) | 5.646 ( 4.252 to 7.397 ) | 0.05 (-0.19 to 0.29) | 57.472 |
| Jamaica | Rheumatic heart disease | Both | 279 ( 213 to 365 ) | 132 ( 103 to 169 ) | 10.318 ( 7.993 to 13.174 ) | 4.917 ( 3.817 to 6.308 ) | -2.72 (-2.89 to -2.55) | -52.688 |
| Japan | Rheumatic heart disease | Both | 4719 ( 3305 to 6369 ) | 7295 ( 5152 to 9676 ) | 3.078 ( 2.247 to 4.07 ) | 2.214 ( 1.702 to 2.8 ) | -1.28 (-1.39 to -1.17) | 54.588 |
| Jordan | Rheumatic heart disease | Both | 125 ( 95 to 163 ) | 324 ( 254 to 415 ) | 2.926 ( 2.298 to 3.624 ) | 2.53 ( 1.992 to 3.219 ) | -0.46 (-0.54 to -0.37) | 159.2 |
| Kazakhstan | Rheumatic heart disease | Both | 1268 ( 1018 to 1577 ) | 330 ( 259 to 415 ) | 8 ( 6.481 to 9.87 ) | 1.891 ( 1.482 to 2.381 ) | -5.48 (-6.02 to -4.95) | -73.975 |
| Kenya | Rheumatic heart disease | Both | 1781 ( 1349 to 2288 ) | 2673 ( 1987 to 3474 ) | 7.322 ( 5.643 to 9.5 ) | 5.451 ( 4.143 to 7.067 ) | -1.25 (-1.32 to -1.18) | 50.084 |
| Kiribati | Rheumatic heart disease | Both | 9 ( 7 to 12 ) | 12 ( 9 to 16 ) | 13.06 ( 10.651 to 16.141 ) | 10.797 ( 8.645 to 13.363 ) | -0.59 (-0.63 to -0.56) | 33.333 |
| Kuwait | Rheumatic heart disease | Both | 68 ( 52 to 87 ) | 114 ( 91 to 142 ) | 4.047 ( 3.232 to 5.033 ) | 2.986 ( 2.35 to 3.739 ) | -1.4 (-1.98 to -0.81) | 67.647 |
| Kyrgyzstan | Rheumatic heart disease | Both | 522 ( 409 to 669 ) | 314 ( 250 to 400 ) | 11.074 ( 8.838 to 13.781 ) | 4.762 ( 3.813 to 5.95 ) | -2.92 (-3.14 to -2.71) | -39.847 |
| Lao People's Democratic Republic | Rheumatic heart disease | Both | 353 ( 266 to 469 ) | 401 ( 299 to 526 ) | 7.247 ( 5.629 to 9.316 ) | 5.204 ( 3.942 to 6.76 ) | -1.24 (-1.29 to -1.19) | 13.598 |
| Latvia | Rheumatic heart disease | Both | 140 ( 107 to 180 ) | 39 ( 27 to 54 ) | 4.53 ( 3.591 to 5.703 ) | 1.264 ( 0.951 to 1.652 ) | -4.97 (-5.39 to -4.55) | -72.143 |
| Lebanon | Rheumatic heart disease | Both | 210 ( 168 to 259 ) | 301 ( 244 to 368 ) | 7.768 ( 6.315 to 9.378 ) | 5.291 ( 4.264 to 6.467 ) | -1.39 (-1.46 to -1.31) | 43.333 |
| Lesotho | Rheumatic heart disease | Both | 170 ( 121 to 239 ) | 160 ( 110 to 229 ) | 8.982 ( 6.503 to 12.389 ) | 7.585 ( 5.375 to 10.367 ) | -0.55 (-0.58 to -0.51) | -5.882 |
| Liberia | Rheumatic heart disease | Both | 205 ( 145 to 285 ) | 323 ( 225 to 451 ) | 7.238 ( 5.129 to 9.942 ) | 5.408 ( 3.869 to 7.3 ) | -1.09 (-1.15 to -1.04) | 57.561 |
| Libya | Rheumatic heart disease | Both | 370 ( 283 to 480 ) | 275 ( 217 to 344 ) | 7.183 ( 5.696 to 9.067 ) | 4.831 ( 3.81 to 6.087 ) | -1.49 (-1.68 to -1.29) | -25.676 |
| Lithuania | Rheumatic heart disease | Both | 282 ( 224 to 355 ) | 77 ( 56 to 102 ) | 6.904 ( 5.449 to 8.617 ) | 2.001 ( 1.588 to 2.506 ) | -4.43 (-4.63 to -4.23) | -72.695 |
| Luxembourg | Rheumatic heart disease | Both | 13 ( 10 to 18 ) | 30 ( 22 to 40 ) | 2.826 ( 2.142 to 3.679 ) | 3.341 ( 2.589 to 4.32 ) | 0.61 (0.3 to 0.92) | 130.769 |
| Madagascar | Rheumatic heart disease | Both | 864 ( 587 to 1228 ) | 1610 ( 1054 to 2358 ) | 5.884 ( 4.126 to 8.179 ) | 4.846 ( 3.375 to 6.72 ) | -0.61 (-0.65 to -0.56) | 86.343 |
| Malawi | Rheumatic heart disease | Both | 463 ( 315 to 664 ) | 640 ( 415 to 932 ) | 4.09 ( 2.85 to 5.671 ) | 2.992 ( 2.097 to 4.157 ) | -1.14 (-1.19 to -1.09) | 38.229 |
| Malaysia | Rheumatic heart disease | Both | 1510 ( 1160 to 1963 ) | 1099 ( 857 to 1391 ) | 7.707 ( 6.06 to 9.785 ) | 3.611 ( 2.808 to 4.574 ) | -2.27 (-2.46 to -2.07) | -27.219 |
| Maldives | Rheumatic heart disease | Both | 16 ( 12 to 22 ) | 14 ( 11 to 18 ) | 6.062 ( 4.716 to 7.718 ) | 3.091 ( 2.417 to 3.965 ) | -2.3 (-2.38 to -2.22) | -12.5 |
| Mali | Rheumatic heart disease | Both | 487 ( 336 to 680 ) | 1055 ( 717 to 1483 ) | 5.407 ( 3.836 to 7.443 ) | 4.195 ( 2.927 to 5.771 ) | -0.77 (-0.9 to -0.64) | 116.632 |
| Malta | Rheumatic heart disease | Both | 7 ( 6 to 9 ) | 18 ( 13 to 24 ) | 1.861 ( 1.433 to 2.327 ) | 2.175 ( 1.688 to 2.805 ) | 1.14 (0.95 to 1.34) | 157.143 |
| Marshall Islands | Rheumatic heart disease | Both | 7 ( 5 to 9 ) | 6 ( 4 to 7 ) | 13.531 ( 10.656 to 16.886 ) | 10.668 ( 8.49 to 13.342 ) | -0.89 (-0.93 to -0.86) | -14.286 |
| Mauritania | Rheumatic heart disease | Both | 181 ( 130 to 246 ) | 247 ( 173 to 344 ) | 7.631 ( 5.527 to 10.332 ) | 5.107 ( 3.63 to 7.06 ) | -1.31 (-1.41 to -1.21) | 36.464 |
| Mauritius | Rheumatic heart disease | Both | 64 ( 48 to 84 ) | 24 ( 19 to 31 ) | 5.46 ( 4.156 to 7.103 ) | 2.216 ( 1.699 to 2.909 ) | -0.83 (-1.77 to 0.12) | -62.5 |
| Mexico | Rheumatic heart disease | Both | 5922 ( 4904 to 7241 ) | 2600 ( 1968 to 3399 ) | 7.983 ( 6.589 to 9.75 ) | 2.033 ( 1.539 to 2.656 ) | -4.97 (-5.14 to -4.8) | -56.096 |
| Micronesia (Federated States of) | Rheumatic heart disease | Both | 14 ( 11 to 19 ) | 10 ( 8 to 13 ) | 12.621 ( 10.132 to 15.834 ) | 9.931 ( 7.811 to 12.645 ) | -0.92 (-0.97 to -0.86) | -28.571 |
| Monaco | Rheumatic heart disease | Both | 1 ( 1 to 1 ) | 1 ( 1 to 2 ) | 1.307 ( 1.036 to 1.638 ) | 1.343 ( 1.055 to 1.687 ) | 0.15 (-0.04 to 0.35) | 0 |
| Mongolia | Rheumatic heart disease | Both | 162 ( 126 to 207 ) | 127 ( 101 to 158 ) | 7.506 ( 5.921 to 9.291 ) | 4.417 ( 3.506 to 5.505 ) | -1.81 (-1.88 to -1.74) | -21.605 |
| Montenegro | Rheumatic heart disease | Both | 17 ( 14 to 21 ) | 15 ( 11 to 20 ) | 2.774 ( 2.224 to 3.482 ) | 1.928 ( 1.524 to 2.382 ) | -1.39 (-1.48 to -1.31) | -11.765 |
| Morocco | Rheumatic heart disease | Both | 2899 ( 2185 to 3884 ) | 2387 ( 1862 to 3074 ) | 9.72 ( 7.487 to 12.672 ) | 6.791 ( 5.263 to 8.74 ) | -1.27 (-1.3 to -1.24) | -17.661 |
| Mozambique | Rheumatic heart disease | Both | 525 ( 355 to 741 ) | 1011 ( 664 to 1473 ) | 3.69 ( 2.579 to 5.083 ) | 3.165 ( 2.183 to 4.337 ) | -0.46 (-0.57 to -0.36) | 92.571 |
| Myanmar | Rheumatic heart disease | Both | 3324 ( 2498 to 4382 ) | 2910 ( 2223 to 3813 ) | 7.34 ( 5.615 to 9.345 ) | 5.152 ( 3.95 to 6.669 ) | -1.32 (-1.36 to -1.27) | -12.455 |
| Namibia | Rheumatic heart disease | Both | 174 ( 121 to 250 ) | 199 ( 138 to 274 ) | 9.865 ( 7.109 to 13.552 ) | 7.482 ( 5.341 to 10.004 ) | -0.96 (-0.99 to -0.93) | 14.368 |
| Nauru | Rheumatic heart disease | Both | 1 ( 1 to 1 ) | 1 ( 1 to 1 ) | 10.144 ( 8.185 to 12.442 ) | 8.813 ( 6.971 to 11.009 ) | -0.59 (-0.68 to -0.5) | 0 |
| Nepal | Rheumatic heart disease | Both | 4532 ( 3610 to 5702 ) | 6520 ( 5222 to 8089 ) | 25.225 ( 20.945 to 30.424 ) | 21.439 ( 17.5 to 26.253 ) | -0.65 (-0.69 to -0.62) | 43.866 |
| Netherlands | Rheumatic heart disease | Both | 229 ( 180 to 294 ) | 791 ( 567 to 1055 ) | 1.237 ( 0.993 to 1.552 ) | 2.401 ( 1.84 to 3.048 ) | 2.27 (1.69 to 2.86) | 245.415 |
| New Zealand | Rheumatic heart disease | Both | 266 ( 212 to 337 ) | 407 ( 315 to 526 ) | 7.184 ( 5.806 to 8.914 ) | 6.215 ( 4.954 to 7.67 ) | -0.4 (-0.54 to -0.26) | 53.008 |
| Nicaragua | Rheumatic heart disease | Both | 181 ( 142 to 233 ) | 107 ( 87 to 131 ) | 5.138 ( 4.158 to 6.355 ) | 1.694 ( 1.394 to 2.073 ) | -4.07 (-4.24 to -3.91) | -40.884 |
| Niger | Rheumatic heart disease | Both | 743 ( 530 to 1030 ) | 1738 ( 1214 to 2424 ) | 7.783 ( 5.668 to 10.613 ) | 6.049 ( 4.332 to 8.316 ) | -0.84 (-0.93 to -0.76) | 133.917 |
| Nigeria | Rheumatic heart disease | Both | 5362 ( 3689 to 7571 ) | 8314 ( 5680 to 11992 ) | 5.22 ( 3.725 to 7.155 ) | 3.285 ( 2.348 to 4.475 ) | -1.82 (-1.94 to -1.69) | 55.054 |
| Niue | Rheumatic heart disease | Both | 0 ( 0 to 0 ) | 0 ( 0 to 0 ) | 10.389 ( 8.389 to 12.574 ) | 8.494 ( 6.865 to 10.598 ) | -0.83 (-0.87 to -0.78) | #NUM! |
| North Macedonia | Rheumatic heart disease | Both | 68 ( 54 to 86 ) | 47 ( 35 to 59 ) | 3.558 ( 2.831 to 4.463 ) | 1.816 ( 1.439 to 2.232 ) | -2.39 (-2.55 to -2.24) | -30.882 |
| Northern Mariana Islands | Rheumatic heart disease | Both | 3 ( 3 to 4 ) | 4 ( 3 to 5 ) | 8.488 ( 6.794 to 10.56 ) | 8.405 ( 6.785 to 10.597 ) | 0.09 (0.04 to 0.14) | 33.333 |
| Norway | Rheumatic heart disease | Both | 115 ( 69 to 171 ) | 199 ( 134 to 290 ) | 1.659 ( 1.069 to 2.371 ) | 1.932 ( 1.347 to 2.73 ) | -0.37 (-1.06 to 0.33) | 73.043 |
| Oman | Rheumatic heart disease | Both | 72 ( 56 to 90 ) | 112 ( 89 to 138 ) | 3.878 ( 3.138 to 4.677 ) | 2.693 ( 2.174 to 3.287 ) | -1.26 (-1.39 to -1.12) | 55.556 |
| Pakistan | Rheumatic heart disease | Both | 24706 ( 20027 to 30765 ) | 47542 ( 38350 to 58878 ) | 24.025 ( 19.742 to 29.192 ) | 21.855 ( 17.76 to 26.678 ) | -0.45 (-0.49 to -0.41) | 92.431 |
| Palau | Rheumatic heart disease | Both | 2 ( 1 to 2 ) | 2 ( 1 to 2 ) | 12.815 ( 10.498 to 15.219 ) | 10.599 ( 8.703 to 12.89 ) | -0.72 (-0.76 to -0.69) | 0 |
| Palestine | Rheumatic heart disease | Both | 198 ( 151 to 260 ) | 321 ( 246 to 416 ) | 7.831 ( 6.182 to 9.769 ) | 5.621 ( 4.434 to 7.083 ) | -1.08 (-1.21 to -0.95) | 62.121 |
| Panama | Rheumatic heart disease | Both | 144 ( 116 to 176 ) | 89 ( 72 to 111 ) | 6.886 ( 5.58 to 8.4 ) | 2.045 ( 1.661 to 2.533 ) | -4.4 (-4.68 to -4.12) | -38.194 |
| Papua New Guinea | Rheumatic heart disease | Both | 636 ( 494 to 823 ) | 1409 ( 1095 to 1794 ) | 16.693 ( 13.57 to 20.408 ) | 14.389 ( 11.695 to 17.725 ) | -0.57 (-0.6 to -0.53) | 121.541 |
| Paraguay | Rheumatic heart disease | Both | 273 ( 206 to 360 ) | 286 ( 222 to 366 ) | 6.055 ( 4.684 to 7.678 ) | 3.961 ( 3.133 to 5.008 ) | -1.51 (-1.57 to -1.45) | 4.762 |
| Peru | Rheumatic heart disease | Both | 869 ( 692 to 1066 ) | 1052 ( 856 to 1294 ) | 4.744 ( 3.884 to 5.765 ) | 2.995 ( 2.452 to 3.677 ) | -1.63 (-1.74 to -1.52) | 21.059 |
| Philippines | Rheumatic heart disease | Both | 4545 ( 3567 to 5845 ) | 5895 ( 4536 to 7734 ) | 6.037 ( 4.826 to 7.646 ) | 4.942 ( 3.857 to 6.436 ) | -0.74 (-0.8 to -0.68) | 29.703 |
| Poland | Rheumatic heart disease | Both | 4293 ( 3311 to 5580 ) | 2667 ( 1918 to 3547 ) | 10.2 ( 7.936 to 13.129 ) | 4.258 ( 3.226 to 5.542 ) | -2.87 (-2.97 to -2.77) | -37.876 |
| Portugal | Rheumatic heart disease | Both | 526 ( 408 to 659 ) | 756 ( 560 to 1007 ) | 4.139 ( 3.308 to 5.072 ) | 3.596 ( 2.853 to 4.573 ) | -0.44 (-0.61 to -0.28) | 43.726 |
| Puerto Rico | Rheumatic heart disease | Both | 117 ( 91 to 146 ) | 90 ( 74 to 110 ) | 3.171 ( 2.479 to 3.958 ) | 2.646 ( 2.12 to 3.238 ) | -1.11 (-1.27 to -0.94) | -23.077 |
| Qatar | Rheumatic heart disease | Both | 21 ( 17 to 26 ) | 108 ( 85 to 137 ) | 5.285 ( 4.304 to 6.319 ) | 4.337 ( 3.518 to 5.29 ) | -0.77 (-0.97 to -0.57) | 414.286 |
| Republic of Korea | Rheumatic heart disease | Both | 766 ( 617 to 937 ) | 1471 ( 1157 to 1841 ) | 2.088 ( 1.677 to 2.549 ) | 1.922 ( 1.556 to 2.339 ) | 0.06 (-0.21 to 0.33) | 92.037 |
| Republic of Moldova | Rheumatic heart disease | Both | 321 ( 257 to 392 ) | 70 ( 56 to 89 ) | 7.206 ( 5.747 to 8.795 ) | 1.953 ( 1.554 to 2.396 ) | -4.79 (-4.97 to -4.61) | -78.193 |
| Romania | Rheumatic heart disease | Both | 1012 ( 786 to 1270 ) | 418 ( 297 to 553 ) | 4.071 ( 3.225 to 5.05 ) | 1.777 ( 1.403 to 2.183 ) | -3.32 (-3.77 to -2.87) | -58.696 |
| Russian Federation | Rheumatic heart disease | Both | 7845 ( 6057 to 10123 ) | 3370 ( 2265 to 4897 ) | 4.862 ( 3.83 to 6.164 ) | 1.615 ( 1.163 to 2.192 ) | -4.09 (-4.49 to -3.68) | -57.043 |
| Rwanda | Rheumatic heart disease | Both | 380 ( 255 to 549 ) | 401 ( 271 to 574 ) | 4.453 ( 3.102 to 6.127 ) | 2.916 ( 2.056 to 3.991 ) | -1.64 (-1.74 to -1.54) | 5.526 |
| Saint Kitts and Nevis | Rheumatic heart disease | Both | 2 ( 1 to 2 ) | 1 ( 1 to 1 ) | 3.39 ( 2.648 to 4.164 ) | 1.712 ( 1.348 to 2.146 ) | -2.37 (-2.47 to -2.26) | -50 |
| Saint Lucia | Rheumatic heart disease | Both | 11 ( 9 to 15 ) | 6 ( 5 to 8 ) | 7.709 ( 6.169 to 9.661 ) | 3.903 ( 3.079 to 4.923 ) | -2.55 (-2.71 to -2.39) | -45.455 |
| Saint Vincent and the Grenadines | Rheumatic heart disease | Both | 11 ( 9 to 15 ) | 5 ( 4 to 6 ) | 8.627 ( 6.759 to 11.218 ) | 4.538 ( 3.501 to 5.854 ) | -2.29 (-2.43 to -2.15) | -54.545 |
| Samoa | Rheumatic heart disease | Both | 20 ( 16 to 27 ) | 22 ( 17 to 28 ) | 11.148 ( 8.866 to 13.869 ) | 9.688 ( 7.587 to 12.352 ) | -0.57 (-0.61 to -0.52) | 10 |
| San Marino | Rheumatic heart disease | Both | 2 ( 1 to 2 ) | 3 ( 2 to 4 ) | 4.643 ( 3.585 to 6.067 ) | 4.263 ( 3.289 to 5.385 ) | -0.1 (-0.15 to -0.05) | 50 |
| Sao Tome and Principe | Rheumatic heart disease | Both | 13 ( 9 to 19 ) | 17 ( 12 to 24 ) | 9.23 ( 6.591 to 12.492 ) | 7.373 ( 5.19 to 10.022 ) | -0.79 (-0.85 to -0.73) | 30.769 |
| Saudi Arabia | Rheumatic heart disease | Both | 630 ( 492 to 784 ) | 569 ( 455 to 698 ) | 3.714 ( 3.001 to 4.499 ) | 1.786 ( 1.442 to 2.21 ) | -2.62 (-2.71 to -2.52) | -9.683 |
| Senegal | Rheumatic heart disease | Both | 858 ( 631 to 1147 ) | 1151 ( 826 to 1558 ) | 9.699 ( 7.191 to 12.574 ) | 6.682 ( 4.919 to 8.869 ) | -1.24 (-1.29 to -1.18) | 34.149 |
| Serbia | Rheumatic heart disease | Both | 200 ( 156 to 254 ) | 137 ( 99 to 182 ) | 1.975 ( 1.54 to 2.488 ) | 1.109 ( 0.87 to 1.373 ) | -2.72 (-3.11 to -2.33) | -31.5 |
| Seychelles | Rheumatic heart disease | Both | 2 ( 1 to 2 ) | 1 ( 1 to 2 ) | 2.13 ( 1.624 to 2.798 ) | 1.277 ( 0.955 to 1.648 ) | -1.25 (-1.56 to -0.94) | -50 |
| Sierra Leone | Rheumatic heart disease | Both | 352 ( 255 to 487 ) | 566 ( 386 to 781 ) | 7.435 ( 5.41 to 10.223 ) | 5.732 ( 4.066 to 7.797 ) | -0.82 (-0.86 to -0.78) | 60.795 |
| Singapore | Rheumatic heart disease | Both | 55 ( 44 to 68 ) | 115 ( 94 to 142 ) | 2.138 ( 1.728 to 2.635 ) | 1.808 ( 1.488 to 2.201 ) | -0.63 (-0.73 to -0.54) | 109.091 |
| Slovakia | Rheumatic heart disease | Both | 182 ( 146 to 230 ) | 158 ( 122 to 205 ) | 3.283 ( 2.637 to 4.131 ) | 2.314 ( 1.876 to 2.849 ) | -0.98 (-1.26 to -0.71) | -13.187 |
| Slovenia | Rheumatic heart disease | Both | 123 ( 91 to 161 ) | 180 ( 122 to 252 ) | 5.3 ( 3.926 to 6.846 ) | 4.42 ( 3.167 to 5.954 ) | -0.63 (-1.08 to -0.18) | 46.341 |
| Solomon Islands | Rheumatic heart disease | Both | 42 ( 32 to 55 ) | 72 ( 55 to 93 ) | 12.495 ( 10.119 to 15.306 ) | 10.546 ( 8.44 to 13.183 ) | -0.65 (-0.68 to -0.63) | 71.429 |
| Somalia | Rheumatic heart disease | Both | 492 ( 331 to 711 ) | 916 ( 608 to 1337 ) | 5.022 ( 3.544 to 7.054 ) | 3.662 ( 2.555 to 5.075 ) | -1.24 (-1.32 to -1.16) | 86.179 |
| South Africa | Rheumatic heart disease | Both | 3817 ( 2768 to 5192 ) | 3619 ( 2623 to 4867 ) | 8.815 ( 6.514 to 11.772 ) | 6.498 ( 4.759 to 8.699 ) | -1.03 (-1.11 to -0.95) | -5.187 |
| South Sudan | Rheumatic heart disease | Both | 347 ( 234 to 508 ) | 449 ( 296 to 649 ) | 4.916 ( 3.465 to 6.765 ) | 4.107 ( 2.869 to 5.78 ) | -0.68 (-0.79 to -0.57) | 29.395 |
| Spain | Rheumatic heart disease | Both | 3988 ( 2988 to 5251 ) | 5904 ( 4719 to 7285 ) | 7.572 ( 5.827 to 9.821 ) | 6.406 ( 5.205 to 7.715 ) | -0.7 (-0.81 to -0.6) | 48.044 |
| Sri Lanka | Rheumatic heart disease | Both | 598 ( 465 to 754 ) | 480 ( 379 to 598 ) | 3.333 ( 2.653 to 4.16 ) | 2.247 ( 1.755 to 2.813 ) | -1.21 (-1.29 to -1.13) | -19.732 |
| Sudan | Rheumatic heart disease | Both | 2119 ( 1594 to 2807 ) | 2950 ( 2246 to 3883 ) | 8.93 ( 7.004 to 11.435 ) | 5.994 ( 4.713 to 7.656 ) | -1.36 (-1.4 to -1.31) | 39.217 |
| Suriname | Rheumatic heart disease | Both | 28 ( 21 to 37 ) | 19 ( 15 to 24 ) | 6.422 ( 4.934 to 8.408 ) | 3.423 ( 2.684 to 4.378 ) | -2.25 (-2.34 to -2.17) | -32.143 |
| Sweden | Rheumatic heart disease | Both | 543 ( 392 to 750 ) | 610 ( 424 to 835 ) | 3.676 ( 2.751 to 4.882 ) | 2.753 ( 1.984 to 3.706 ) | -1.33 (-1.66 to -0.99) | 12.339 |
| Switzerland | Rheumatic heart disease | Both | 229 ( 165 to 295 ) | 328 ( 248 to 438 ) | 2.509 ( 1.909 to 3.134 ) | 2.325 ( 1.848 to 2.935 ) | 0.08 (-0.36 to 0.52) | 43.231 |
| Syrian Arab Republic | Rheumatic heart disease | Both | 1545 ( 1179 to 2041 ) | 679 ( 519 to 892 ) | 9.616 ( 7.616 to 12.257 ) | 5.002 ( 3.861 to 6.475 ) | -2.71 (-2.94 to -2.47) | -56.052 |
| Taiwan (Province of China) | Rheumatic heart disease | Both | 1007 ( 801 to 1263 ) | 876 ( 660 to 1152 ) | 6.371 ( 5.007 to 8.137 ) | 2.247 ( 1.737 to 2.878 ) | -4.05 (-4.32 to -3.77) | -13.009 |
| Tajikistan | Rheumatic heart disease | Both | 511 ( 380 to 688 ) | 867 ( 649 to 1141 ) | 7.734 ( 5.914 to 10.123 ) | 7.719 ( 5.88 to 10.086 ) | -0.16 (-0.22 to -0.09) | 69.667 |
| Thailand | Rheumatic heart disease | Both | 2567 ( 1924 to 3382 ) | 1606 ( 1309 to 1962 ) | 4.309 ( 3.312 to 5.555 ) | 2.69 ( 2.091 to 3.394 ) | -1.64 (-1.73 to -1.55) | -37.437 |
| Timor-Leste | Rheumatic heart disease | Both | 72 ( 54 to 93 ) | 102 ( 75 to 135 ) | 8.222 ( 6.403 to 10.265 ) | 6.299 ( 4.794 to 8.172 ) | -1.01 (-1.06 to -0.96) | 41.667 |
| Togo | Rheumatic heart disease | Both | 328 ( 233 to 460 ) | 476 ( 335 to 662 ) | 7.475 ( 5.412 to 10.163 ) | 5.291 ( 3.817 to 7.133 ) | -1.19 (-1.23 to -1.14) | 45.122 |
| Tokelau | Rheumatic heart disease | Both | 0 ( 0 to 0 ) | 0 ( 0 to 0 ) | 12.118 ( 9.901 to 14.524 ) | 8.802 ( 7.147 to 10.88 ) | -1.25 (-1.3 to -1.19) | #NUM! |
| Tonga | Rheumatic heart disease | Both | 12 ( 9 to 15 ) | 10 ( 8 to 12 ) | 11.329 ( 9.056 to 14.136 ) | 8.625 ( 6.83 to 10.748 ) | -0.97 (-1 to -0.93) | -16.667 |
| Trinidad and Tobago | Rheumatic heart disease | Both | 91 ( 69 to 119 ) | 47 ( 38 to 59 ) | 6.818 ( 5.253 to 8.787 ) | 3.716 ( 2.909 to 4.718 ) | -2.27 (-2.44 to -2.09) | -48.352 |
| Tunisia | Rheumatic heart disease | Both | 430 ( 341 to 528 ) | 395 ( 319 to 479 ) | 5.008 ( 4.037 to 6.042 ) | 3.449 ( 2.789 to 4.19 ) | -1.26 (-1.36 to -1.16) | -8.14 |
| Turkey | Rheumatic heart disease | Both | 2533 ( 2031 to 3119 ) | 3032 ( 2497 to 3646 ) | 4.424 ( 3.616 to 5.454 ) | 3.659 ( 3.01 to 4.431 ) | -0.76 (-0.97 to -0.55) | 19.7 |
| Turkmenistan | Rheumatic heart disease | Both | 307 ( 236 to 400 ) | 128 ( 102 to 162 ) | 7.415 ( 5.868 to 9.402 ) | 2.597 ( 2.087 to 3.256 ) | -4.21 (-4.67 to -3.76) | -58.306 |
| Tuvalu | Rheumatic heart disease | Both | 1 ( 1 to 1 ) | 1 ( 1 to 1 ) | 12.462 ( 10.188 to 14.937 ) | 9.232 ( 7.453 to 11.26 ) | -1.17 (-1.24 to -1.11) | 0 |
| Uganda | Rheumatic heart disease | Both | 1288 ( 906 to 1841 ) | 1821 ( 1239 to 2573 ) | 6.134 ( 4.402 to 8.607 ) | 3.9 ( 2.808 to 5.252 ) | -1.62 (-1.67 to -1.57) | 41.382 |
| Ukraine | Rheumatic heart disease | Both | 1140 ( 819 to 1543 ) | 600 ( 392 to 879 ) | 1.802 ( 1.35 to 2.365 ) | 0.935 ( 0.656 to 1.274 ) | -2.43 (-2.67 to -2.2) | -47.368 |
| United Arab Emirates | Rheumatic heart disease | Both | 155 ( 121 to 198 ) | 447 ( 363 to 561 ) | 8.957 ( 7.246 to 11.079 ) | 6.613 ( 5.435 to 8.136 ) | -0.91 (-0.98 to -0.84) | 188.387 |
| United Kingdom | Rheumatic heart disease | Both | 2188 ( 1577 to 3002 ) | 1741 ( 1240 to 2357 ) | 2.601 ( 1.967 to 3.41 ) | 1.471 ( 1.136 to 1.912 ) | -2.39 (-2.54 to -2.24) | -20.43 |
| United Republic of Tanzania | Rheumatic heart disease | Both | 1158 ( 770 to 1694 ) | 1931 ( 1278 to 2743 ) | 3.918 ( 2.717 to 5.452 ) | 3.085 ( 2.163 to 4.253 ) | -0.62 (-0.72 to -0.52) | 66.753 |
| United States of America | Rheumatic heart disease | Both | 11682 ( 8839 to 15160 ) | 11908 ( 8546 to 15821 ) | 3.927 ( 3.025 to 5.009 ) | 2.444 ( 1.845 to 3.107 ) | -1.68 (-2.14 to -1.23) | 1.935 |
| United States Virgin Islands | Rheumatic heart disease | Both | 4 ( 3 to 4 ) | 2 ( 1 to 2 ) | 3.333 ( 2.613 to 4.122 ) | 2.324 ( 1.842 to 2.869 ) | -1.45 (-1.54 to -1.35) | -50 |
| Uruguay | Rheumatic heart disease | Both | 75 ( 59 to 93 ) | 49 ( 35 to 64 ) | 2.181 ( 1.743 to 2.663 ) | 1.032 ( 0.801 to 1.3 ) | -2.4 (-2.52 to -2.28) | -34.667 |
| Uzbekistan | Rheumatic heart disease | Both | 2603 ( 1999 to 3353 ) | 2511 ( 1947 to 3144 ) | 10.693 ( 8.396 to 13.483 ) | 7.744 ( 6.014 to 9.631 ) | -0.97 (-1.23 to -0.7) | -3.534 |
| Vanuatu | Rheumatic heart disease | Both | 24 ( 18 to 31 ) | 41 ( 31 to 52 ) | 15.194 ( 12.132 to 18.726 ) | 12.722 ( 10.086 to 15.761 ) | -0.65 (-0.68 to -0.62) | 70.833 |
| Venezuela (Bolivarian Republic of) | Rheumatic heart disease | Both | 612 ( 482 to 758 ) | 261 ( 209 to 325 ) | 3.275 ( 2.633 to 3.958 ) | 0.973 ( 0.777 to 1.205 ) | -4.66 (-4.95 to -4.37) | -57.353 |
| Viet Nam | Rheumatic heart disease | Both | 4216 ( 3326 to 5303 ) | 4012 ( 3187 to 4994 ) | 5.834 ( 4.702 to 7.224 ) | 4.263 ( 3.395 to 5.275 ) | -1.21 (-1.28 to -1.15) | -4.839 |
| Yemen | Rheumatic heart disease | Both | 1623 ( 1234 to 2165 ) | 2506 ( 1902 to 3300 ) | 9.457 ( 7.489 to 12.164 ) | 6.502 ( 5.16 to 8.209 ) | -1.44 (-1.5 to -1.38) | 54.405 |
| Zambia | Rheumatic heart disease | Both | 326 ( 220 to 474 ) | 552 ( 360 to 795 ) | 3.544 ( 2.508 to 4.964 ) | 2.726 ( 1.882 to 3.844 ) | -0.97 (-1.04 to -0.9) | 69.325 |
| Zimbabwe | Rheumatic heart disease | Both | 1553 ( 1113 to 2154 ) | 2092 ( 1424 to 2957 ) | 11.416 ( 8.493 to 15.096 ) | 11.146 ( 7.86 to 15.274 ) | -0.06 (-0.07 to -0.04) | 34.707 |

**Table S8 The YLDs of SHF due to RHD in 1990 and 2021 across countries and territories.**

| Locations | cause | sex | 1990 YLDs cases(95% UI) | 2021 YLDs cases(95% UI) | 1990 ASYR(95% UI) | 2021 ASYR(95% UI) | 1990-2021 EAPC（95%CI） | 1990-2021 YLDs cases changes |
| --- | --- | --- | --- | --- | --- | --- | --- | --- |
| Afghanistan | Rheumatic heart disease | Both | 163 ( 93 to 268 ) | 393 ( 207 to 680 ) | 1.44 ( 0.856 to 2.268 ) | 1.061 ( 0.627 to 1.707 ) | -1.23 (-1.32 to -1.14) | 141.104 |
| Albania | Rheumatic heart disease | Both | 44 ( 28 to 66 ) | 20 ( 12 to 30 ) | 1.511 ( 0.962 to 2.28 ) | 0.623 ( 0.384 to 0.905 ) | -3.22 (-3.43 to -3.02) | -54.545 |
| Algeria | Rheumatic heart disease | Both | 361 ( 192 to 615 ) | 322 ( 205 to 487 ) | 1.188 ( 0.674 to 1.929 ) | 0.742 ( 0.475 to 1.108 ) | -1.6 (-1.66 to -1.54) | -10.803 |
| American Samoa | Rheumatic heart disease | Both | 1 ( 0 to 1 ) | 1 ( 0 to 1 ) | 1.795 ( 1.099 to 2.751 ) | 1.22 ( 0.742 to 1.832 ) | -1.39 (-1.53 to -1.26) | 0 |
| Andorra | Rheumatic heart disease | Both | 0 ( 0 to 0 ) | 0 ( 0 to 1 ) | 0.333 ( 0.199 to 0.514 ) | 0.265 ( 0.16 to 0.413 ) | -0.63 (-0.67 to -0.6) | #NUM! |
| Angola | Rheumatic heart disease | Both | 132 ( 72 to 226 ) | 302 ( 169 to 483 ) | 1.087 ( 0.617 to 1.773 ) | 0.842 ( 0.502 to 1.33 ) | -1.01 (-1.08 to -0.94) | 128.788 |
| Antigua and Barbuda | Rheumatic heart disease | Both | 1 ( 0 to 1 ) | 0 ( 0 to 1 ) | 1.05 ( 0.609 to 1.643 ) | 0.453 ( 0.283 to 0.668 ) | -2.98 (-3.11 to -2.86) | -100 |
| Argentina | Rheumatic heart disease | Both | 288 ( 177 to 438 ) | 172 ( 103 to 260 ) | 0.886 ( 0.545 to 1.346 ) | 0.326 ( 0.198 to 0.489 ) | -3.84 (-4.24 to -3.44) | -40.278 |
| Armenia | Rheumatic heart disease | Both | 55 ( 33 to 83 ) | 27 ( 16 to 40 ) | 1.628 ( 0.982 to 2.454 ) | 0.843 ( 0.508 to 1.288 ) | -1.93 (-2.1 to -1.76) | -50.909 |
| Australia | Rheumatic heart disease | Both | 166 ( 106 to 242 ) | 361 ( 221 to 550 ) | 0.913 ( 0.588 to 1.318 ) | 0.969 ( 0.603 to 1.437 ) | 0.31 (0.15 to 0.47) | 117.47 |
| Austria | Rheumatic heart disease | Both | 80 ( 47 to 128 ) | 98 ( 57 to 148 ) | 0.699 ( 0.429 to 1.089 ) | 0.576 ( 0.347 to 0.853 ) | -1.45 (-1.79 to -1.11) | 22.5 |
| Azerbaijan | Rheumatic heart disease | Both | 39 ( 25 to 58 ) | 39 ( 24 to 57 ) | 0.532 ( 0.337 to 0.777 ) | 0.408 ( 0.259 to 0.608 ) | -1.1 (-1.31 to -0.9) | 0 |
| Bahamas | Rheumatic heart disease | Both | 2 ( 1 to 2 ) | 1 ( 1 to 1 ) | 0.558 ( 0.354 to 0.844 ) | 0.263 ( 0.165 to 0.405 ) | -2.95 (-3.12 to -2.77) | -50 |
| Bahrain | Rheumatic heart disease | Both | 4 ( 2 to 6 ) | 6 ( 4 to 8 ) | 0.815 ( 0.528 to 1.201 ) | 0.44 ( 0.282 to 0.644 ) | -2.51 (-2.73 to -2.29) | 50 |
| Bangladesh | Rheumatic heart disease | Both | 3657 ( 2229 to 5764 ) | 5511 ( 3441 to 8225 ) | 3.818 ( 2.393 to 5.95 ) | 3.432 ( 2.158 to 5.183 ) | -0.32 (-0.35 to -0.29) | 50.697 |
| Barbados | Rheumatic heart disease | Both | 2 ( 1 to 4 ) | 1 ( 1 to 2 ) | 0.931 ( 0.58 to 1.382 ) | 0.468 ( 0.297 to 0.686 ) | -2.41 (-2.52 to -2.29) | -50 |
| Belarus | Rheumatic heart disease | Both | 67 ( 41 to 99 ) | 39 ( 22 to 64 ) | 0.592 ( 0.366 to 0.878 ) | 0.326 ( 0.191 to 0.496 ) | -2.14 (-2.25 to -2.02) | -41.791 |
| Belgium | Rheumatic heart disease | Both | 62 ( 36 to 97 ) | 117 ( 72 to 175 ) | 0.45 ( 0.269 to 0.68 ) | 0.563 ( 0.356 to 0.817 ) | 1.42 (1.24 to 1.6) | 88.71 |
| Belize | Rheumatic heart disease | Both | 2 ( 1 to 4 ) | 2 ( 1 to 3 ) | 0.987 ( 0.584 to 1.554 ) | 0.421 ( 0.262 to 0.627 ) | -2.95 (-3.02 to -2.88) | 0 |
| Benin | Rheumatic heart disease | Both | 68 ( 36 to 118 ) | 138 ( 75 to 228 ) | 1.23 ( 0.676 to 1.991 ) | 0.935 ( 0.547 to 1.496 ) | -0.97 (-1.09 to -0.85) | 102.941 |
| Bermuda | Rheumatic heart disease | Both | 0 ( 0 to 1 ) | 0 ( 0 to 0 ) | 0.758 ( 0.482 to 1.118 ) | 0.478 ( 0.302 to 0.687 ) | -1.82 (-1.92 to -1.72) | #NUM! |
| Bhutan | Rheumatic heart disease | Both | 25 ( 15 to 36 ) | 25 ( 16 to 40 ) | 4.438 ( 2.806 to 6.424 ) | 3.527 ( 2.243 to 5.327 ) | -0.82 (-0.85 to -0.79) | 0 |
| Bolivia (Plurinational State of) | Rheumatic heart disease | Both | 58 ( 37 to 86 ) | 68 ( 43 to 101 ) | 1.064 ( 0.676 to 1.585 ) | 0.636 ( 0.396 to 0.936 ) | -1.84 (-1.91 to -1.77) | 17.241 |
| Bosnia and Herzegovina | Rheumatic heart disease | Both | 21 ( 13 to 31 ) | 10 ( 6 to 15 ) | 0.495 ( 0.297 to 0.733 ) | 0.212 ( 0.131 to 0.314 ) | -3.08 (-3.28 to -2.89) | -52.381 |
| Botswana | Rheumatic heart disease | Both | 28 ( 15 to 47 ) | 29 ( 16 to 50 ) | 1.662 ( 0.91 to 2.678 ) | 1.218 ( 0.68 to 2.034 ) | -0.98 (-1.02 to -0.93) | 3.571 |
| Brazil | Rheumatic heart disease | Both | 1834 ( 1122 to 2742 ) | 1413 ( 876 to 2091 ) | 1.145 ( 0.719 to 1.715 ) | 0.653 ( 0.399 to 0.963 ) | -2.06 (-2.14 to -1.99) | -22.955 |
| Brunei Darussalam | Rheumatic heart disease | Both | 1 ( 0 to 1 ) | 1 ( 1 to 2 ) | 0.349 ( 0.217 to 0.538 ) | 0.272 ( 0.168 to 0.413 ) | -0.94 (-1.02 to -0.86) | 0 |
| Bulgaria | Rheumatic heart disease | Both | 77 ( 47 to 121 ) | 23 ( 14 to 35 ) | 0.749 ( 0.471 to 1.13 ) | 0.299 ( 0.183 to 0.437 ) | -3.4 (-3.86 to -2.94) | -70.13 |
| Burkina Faso | Rheumatic heart disease | Both | 136 ( 72 to 229 ) | 246 ( 132 to 412 ) | 1.24 ( 0.686 to 2.076 ) | 0.99 ( 0.537 to 1.587 ) | -0.74 (-0.82 to -0.67) | 80.882 |
| Burundi | Rheumatic heart disease | Both | 61 ( 33 to 101 ) | 93 ( 53 to 148 ) | 0.906 ( 0.502 to 1.437 ) | 0.638 ( 0.384 to 0.993 ) | -1.28 (-1.32 to -1.24) | 52.459 |
| Cabo Verde | Rheumatic heart disease | Both | 9 ( 5 to 14 ) | 6 ( 3 to 9 ) | 1.993 ( 1.12 to 3.307 ) | 1.053 ( 0.587 to 1.666 ) | -1.99 (-2.23 to -1.74) | -33.333 |
| Cambodia | Rheumatic heart disease | Both | 131 ( 77 to 199 ) | 139 ( 85 to 207 ) | 1.149 ( 0.706 to 1.712 ) | 0.801 ( 0.491 to 1.198 ) | -1.35 (-1.42 to -1.29) | 6.107 |
| Cameroon | Rheumatic heart disease | Both | 108 ( 60 to 176 ) | 238 ( 130 to 396 ) | 0.915 ( 0.512 to 1.49 ) | 0.686 ( 0.39 to 1.084 ) | -1.22 (-1.31 to -1.12) | 120.37 |
| Canada | Rheumatic heart disease | Both | 322 ( 196 to 482 ) | 600 ( 366 to 906 ) | 1.04 ( 0.64 to 1.537 ) | 0.958 ( 0.588 to 1.424 ) | -0.18 (-0.34 to -0.01) | 86.335 |
| Central African Republic | Rheumatic heart disease | Both | 34 ( 18 to 55 ) | 57 ( 31 to 96 ) | 1.051 ( 0.594 to 1.676 ) | 0.912 ( 0.52 to 1.491 ) | -0.53 (-0.58 to -0.49) | 67.647 |
| Chad | Rheumatic heart disease | Both | 96 ( 52 to 158 ) | 217 ( 115 to 376 ) | 1.347 ( 0.744 to 2.174 ) | 1.057 ( 0.592 to 1.773 ) | -0.82 (-0.89 to -0.75) | 126.042 |
| Chile | Rheumatic heart disease | Both | 125 ( 80 to 185 ) | 80 ( 51 to 121 ) | 1.035 ( 0.644 to 1.536 ) | 0.377 ( 0.24 to 0.561 ) | -3.71 (-3.89 to -3.53) | -36 |
| China | Rheumatic heart disease | Both | 17883 ( 11470 to 26190 ) | 26280 ( 15599 to 40186 ) | 1.948 ( 1.276 to 2.874 ) | 1.326 ( 0.8 to 1.993 ) | -1.55 (-1.67 to -1.44) | 46.955 |
| Colombia | Rheumatic heart disease | Both | 209 ( 133 to 306 ) | 84 ( 53 to 122 ) | 0.671 ( 0.435 to 0.968 ) | 0.166 ( 0.104 to 0.244 ) | -5.17 (-5.67 to -4.66) | -59.809 |
| Comoros | Rheumatic heart disease | Both | 5 ( 3 to 8 ) | 5 ( 3 to 7 ) | 0.898 ( 0.494 to 1.41 ) | 0.592 ( 0.351 to 0.916 ) | -1.47 (-1.52 to -1.42) | 0 |
| Congo | Rheumatic heart disease | Both | 31 ( 17 to 52 ) | 45 ( 26 to 71 ) | 1.068 ( 0.605 to 1.768 ) | 0.789 ( 0.469 to 1.261 ) | -1.14 (-1.21 to -1.07) | 45.161 |
| Cook Islands | Rheumatic heart disease | Both | 0 ( 0 to 0 ) | 0 ( 0 to 0 ) | 1.212 ( 0.66 to 1.94 ) | 1.109 ( 0.64 to 1.683 ) | -0.47 (-0.56 to -0.37) | #NUM! |
| Costa Rica | Rheumatic heart disease | Both | 35 ( 22 to 52 ) | 19 ( 12 to 29 ) | 1.27 ( 0.789 to 1.891 ) | 0.374 ( 0.228 to 0.557 ) | -4.24 (-4.41 to -4.07) | -45.714 |
| Coted'Ivoire | Rheumatic heart disease | Both | 174 ( 91 to 287 ) | 279 ( 157 to 451 ) | 1.23 ( 0.698 to 1.952 ) | 0.93 ( 0.547 to 1.49 ) | -0.91 (-0.98 to -0.84) | 60.345 |
| Croatia | Rheumatic heart disease | Both | 34 ( 20 to 50 ) | 20 ( 11 to 34 ) | 0.603 ( 0.366 to 0.902 ) | 0.305 ( 0.187 to 0.477 ) | -3.07 (-3.89 to -2.24) | -41.176 |
| Cuba | Rheumatic heart disease | Both | 172 ( 102 to 271 ) | 100 ( 64 to 148 ) | 1.577 ( 0.935 to 2.437 ) | 0.968 ( 0.615 to 1.463 ) | -1.84 (-2.05 to -1.62) | -41.86 |
| Cyprus | Rheumatic heart disease | Both | 5 ( 3 to 9 ) | 11 ( 6 to 16 ) | 0.664 ( 0.387 to 1.009 ) | 0.567 ( 0.334 to 0.845 ) | -0.87 (-1.04 to -0.7) | 120 |
| Czechia | Rheumatic heart disease | Both | 122 ( 73 to 192 ) | 103 ( 59 to 164 ) | 0.971 ( 0.594 to 1.484 ) | 0.611 ( 0.387 to 0.925 ) | -1.52 (-1.88 to -1.16) | -15.574 |
| Democratic People's Republic of Korea | Rheumatic heart disease | Both | 254 ( 165 to 372 ) | 299 ( 180 to 461 ) | 1.546 ( 0.993 to 2.237 ) | 1.004 ( 0.611 to 1.518 ) | -1.55 (-1.6 to -1.51) | 17.717 |
| Democratic Republic of the Congo | Rheumatic heart disease | Both | 498 ( 275 to 809 ) | 796 ( 454 to 1285 ) | 1.095 ( 0.61 to 1.761 ) | 0.797 ( 0.468 to 1.272 ) | -1.17 (-1.25 to -1.09) | 59.839 |
| Denmark | Rheumatic heart disease | Both | 19 ( 11 to 28 ) | 48 ( 29 to 76 ) | 0.274 ( 0.174 to 0.395 ) | 0.465 ( 0.287 to 0.701 ) | 2.11 (1.88 to 2.34) | 152.632 |
| Djibouti | Rheumatic heart disease | Both | 4 ( 2 to 7 ) | 8 ( 4 to 12 ) | 0.866 ( 0.468 to 1.335 ) | 0.61 ( 0.356 to 0.945 ) | -1.3 (-1.35 to -1.24) | 100 |
| Dominica | Rheumatic heart disease | Both | 1 ( 0 to 1 ) | 0 ( 0 to 0 ) | 0.924 ( 0.563 to 1.415 ) | 0.523 ( 0.327 to 0.783 ) | -2.05 (-2.16 to -1.94) | -100 |
| Dominican Republic | Rheumatic heart disease | Both | 114 ( 65 to 188 ) | 97 ( 58 to 152 ) | 1.374 ( 0.807 to 2.211 ) | 0.896 ( 0.535 to 1.399 ) | -1.67 (-1.76 to -1.59) | -14.912 |
| Ecuador | Rheumatic heart disease | Both | 82 ( 52 to 123 ) | 68 ( 42 to 102 ) | 0.912 ( 0.566 to 1.352 ) | 0.386 ( 0.239 to 0.581 ) | -2.71 (-2.86 to -2.56) | -17.073 |
| Egypt | Rheumatic heart disease | Both | 1164 ( 690 to 1888 ) | 1230 ( 734 to 1944 ) | 1.956 ( 1.185 to 3.071 ) | 1.138 ( 0.701 to 1.754 ) | -2.05 (-2.15 to -1.96) | 5.67 |
| El Salvador | Rheumatic heart disease | Both | 22 ( 14 to 34 ) | 10 ( 7 to 15 ) | 0.463 ( 0.295 to 0.685 ) | 0.163 ( 0.104 to 0.237 ) | -3.79 (-3.98 to -3.61) | -54.545 |
| Equatorial Guinea | Rheumatic heart disease | Both | 6 ( 3 to 10 ) | 11 ( 6 to 17 ) | 1.127 ( 0.632 to 1.899 ) | 0.652 ( 0.381 to 1.019 ) | -2.29 (-2.49 to -2.1) | 83.333 |
| Eritrea | Rheumatic heart disease | Both | 42 ( 20 to 72 ) | 46 ( 26 to 74 ) | 0.983 ( 0.512 to 1.599 ) | 0.657 ( 0.375 to 1.049 ) | -1.38 (-1.45 to -1.31) | 9.524 |
| Estonia | Rheumatic heart disease | Both | 14 ( 9 to 21 ) | 3 ( 2 to 5 ) | 0.807 ( 0.508 to 1.217 ) | 0.192 ( 0.117 to 0.286 ) | -5.57 (-5.88 to -5.27) | -78.571 |
| Eswatini | Rheumatic heart disease | Both | 17 ( 9 to 29 ) | 17 ( 8 to 29 ) | 1.642 ( 0.859 to 2.647 ) | 1.3 ( 0.648 to 2.153 ) | -0.77 (-0.81 to -0.73) | 0 |
| Ethiopia | Rheumatic heart disease | Both | 212 ( 120 to 339 ) | 332 ( 189 to 522 ) | 0.406 ( 0.242 to 0.627 ) | 0.338 ( 0.201 to 0.528 ) | -0.52 (-0.59 to -0.44) | 56.604 |
| Fiji | Rheumatic heart disease | Both | 18 ( 10 to 28 ) | 17 ( 10 to 25 ) | 2.384 ( 1.434 to 3.604 ) | 1.878 ( 1.128 to 2.884 ) | -0.94 (-0.99 to -0.89) | -5.556 |
| Finland | Rheumatic heart disease | Both | 22 ( 12 to 35 ) | 32 ( 19 to 51 ) | 0.348 ( 0.204 to 0.545 ) | 0.328 ( 0.202 to 0.489 ) | -0.32 (-0.6 to -0.05) | 45.455 |
| France | Rheumatic heart disease | Both | 1231 ( 757 to 1848 ) | 1991 ( 1217 to 2983 ) | 1.497 ( 0.928 to 2.224 ) | 1.448 ( 0.888 to 2.181 ) | 0.11 (-0.22 to 0.44) | 61.738 |
| Gabon | Rheumatic heart disease | Both | 11 ( 6 to 18 ) | 14 ( 8 to 23 ) | 0.976 ( 0.545 to 1.557 ) | 0.747 ( 0.442 to 1.171 ) | -1 (-1.04 to -0.97) | 27.273 |
| Gambia | Rheumatic heart disease | Both | 16 ( 8 to 27 ) | 27 ( 14 to 45 ) | 1.337 ( 0.759 to 2.226 ) | 0.978 ( 0.551 to 1.599 ) | -0.99 (-1.13 to -0.86) | 68.75 |
| Georgia | Rheumatic heart disease | Both | 134 ( 81 to 206 ) | 38 ( 23 to 58 ) | 2.539 ( 1.508 to 3.925 ) | 1.004 ( 0.642 to 1.476 ) | -2.96 (-3.59 to -2.33) | -71.642 |
| Germany | Rheumatic heart disease | Both | 676 ( 376 to 1043 ) | 1525 ( 893 to 2405 ) | 0.597 ( 0.344 to 0.898 ) | 0.819 ( 0.492 to 1.226 ) | 1.47 (1.11 to 1.82) | 125.592 |
| Ghana | Rheumatic heart disease | Both | 224 ( 128 to 368 ) | 275 ( 163 to 429 ) | 1.402 ( 0.859 to 2.212 ) | 0.827 ( 0.504 to 1.279 ) | -2.18 (-2.38 to -1.97) | 22.768 |
| Greece | Rheumatic heart disease | Both | 24 ( 14 to 35 ) | 27 ( 16 to 42 ) | 0.175 ( 0.109 to 0.258 ) | 0.152 ( 0.093 to 0.223 ) | -1.13 (-1.65 to -0.61) | 12.5 |
| Greenland | Rheumatic heart disease | Both | 0 ( 0 to 0 ) | 0 ( 0 to 0 ) | 0.655 ( 0.39 to 1.059 ) | 0.388 ( 0.218 to 0.628 ) | -2.14 (-2.33 to -1.95) | #NUM! |
| Grenada | Rheumatic heart disease | Both | 1 ( 1 to 2 ) | 1 ( 1 to 1 ) | 1.535 ( 0.936 to 2.476 ) | 0.824 ( 0.532 to 1.199 ) | -2.27 (-2.37 to -2.18) | 0 |
| Guam | Rheumatic heart disease | Both | 2 ( 1 to 3 ) | 2 ( 1 to 3 ) | 1.307 ( 0.734 to 2.038 ) | 1.22 ( 0.672 to 1.865 ) | -0.53 (-0.64 to -0.42) | 0 |
| Guatemala | Rheumatic heart disease | Both | 41 ( 25 to 62 ) | 14 ( 9 to 21 ) | 0.487 ( 0.307 to 0.723 ) | 0.096 ( 0.062 to 0.142 ) | -5.82 (-6.14 to -5.49) | -65.854 |
| Guinea | Rheumatic heart disease | Both | 87 ( 47 to 142 ) | 149 ( 78 to 249 ) | 1.301 ( 0.736 to 2.003 ) | 1.001 ( 0.546 to 1.631 ) | -0.85 (-0.93 to -0.76) | 71.264 |
| Guinea-Bissau | Rheumatic heart disease | Both | 16 ( 8 to 27 ) | 24 ( 13 to 38 ) | 1.307 ( 0.694 to 2.074 ) | 1.022 ( 0.556 to 1.611 ) | -0.77 (-0.87 to -0.68) | 50 |
| Guyana | Rheumatic heart disease | Both | 10 ( 5 to 17 ) | 3 ( 2 to 5 ) | 1.05 ( 0.603 to 1.794 ) | 0.449 ( 0.277 to 0.671 ) | -2.99 (-3.16 to -2.82) | -70 |
| Haiti | Rheumatic heart disease | Both | 125 ( 69 to 196 ) | 179 ( 103 to 289 ) | 1.778 ( 1.059 to 2.749 ) | 1.323 ( 0.762 to 2.087 ) | -1.04 (-1.07 to -1) | 43.2 |
| Honduras | Rheumatic heart disease | Both | 7 ( 5 to 11 ) | 11 ( 7 to 16 ) | 0.194 ( 0.123 to 0.281 ) | 0.124 ( 0.078 to 0.182 ) | -1.5 (-1.53 to -1.46) | 57.143 |
| Hungary | Rheumatic heart disease | Both | 88 ( 51 to 140 ) | 37 ( 21 to 63 ) | 0.688 ( 0.418 to 1.075 ) | 0.273 ( 0.165 to 0.415 ) | -3.25 (-3.7 to -2.79) | -57.955 |
| Iceland | Rheumatic heart disease | Both | 0 ( 0 to 1 ) | 1 ( 1 to 2 ) | 0.167 ( 0.103 to 0.25 ) | 0.257 ( 0.162 to 0.379 ) | 1.04 (0.83 to 1.25) | #NUM! |
| India | Rheumatic heart disease | Both | 32994 ( 21256 to 47946 ) | 50498 ( 32263 to 73443 ) | 4.117 ( 2.668 to 5.925 ) | 3.638 ( 2.333 to 5.275 ) | -0.44 (-0.47 to -0.41) | 53.052 |
| Indonesia | Rheumatic heart disease | Both | 1758 ( 1134 to 2544 ) | 1502 ( 950 to 2141 ) | 0.896 ( 0.58 to 1.302 ) | 0.574 ( 0.364 to 0.82 ) | -1.88 (-2.01 to -1.75) | -14.562 |
| Iran (Islamic Republic of) | Rheumatic heart disease | Both | 933 ( 598 to 1388 ) | 803 ( 494 to 1178 ) | 1.437 ( 0.917 to 2.111 ) | 1.031 ( 0.626 to 1.516 ) | -1.29 (-1.49 to -1.08) | -13.934 |
| Iraq | Rheumatic heart disease | Both | 265 ( 159 to 418 ) | 351 ( 223 to 526 ) | 1.308 ( 0.824 to 1.956 ) | 0.842 ( 0.54 to 1.252 ) | -1.59 (-1.65 to -1.54) | 32.453 |
| Ireland | Rheumatic heart disease | Both | 13 ( 7 to 20 ) | 30 ( 18 to 48 ) | 0.317 ( 0.186 to 0.478 ) | 0.428 ( 0.26 to 0.657 ) | 1.37 (1.25 to 1.5) | 130.769 |
| Israel | Rheumatic heart disease | Both | 25 ( 15 to 40 ) | 101 ( 58 to 155 ) | 0.506 ( 0.307 to 0.794 ) | 0.841 ( 0.505 to 1.289 ) | 2.03 (1.81 to 2.24) | 304 |
| Italy | Rheumatic heart disease | Both | 756 ( 462 to 1177 ) | 1176 ( 700 to 1863 ) | 1.031 ( 0.659 to 1.565 ) | 0.989 ( 0.606 to 1.489 ) | 0.03 (-0.21 to 0.26) | 55.556 |
| Jamaica | Rheumatic heart disease | Both | 49 ( 28 to 78 ) | 24 ( 15 to 37 ) | 1.822 ( 1.071 to 2.84 ) | 0.882 ( 0.535 to 1.383 ) | -2.66 (-2.82 to -2.49) | -51.02 |
| Japan | Rheumatic heart disease | Both | 832 ( 497 to 1308 ) | 1265 ( 784 to 1967 ) | 0.542 ( 0.336 to 0.833 ) | 0.389 ( 0.245 to 0.578 ) | -1.28 (-1.38 to -1.18) | 52.043 |
| Jordan | Rheumatic heart disease | Both | 22 ( 14 to 33 ) | 58 ( 37 to 85 ) | 0.524 ( 0.336 to 0.775 ) | 0.453 ( 0.289 to 0.665 ) | -0.46 (-0.54 to -0.37) | 163.636 |
| Kazakhstan | Rheumatic heart disease | Both | 224 ( 138 to 339 ) | 59 ( 34 to 91 ) | 1.41 ( 0.869 to 2.116 ) | 0.336 ( 0.196 to 0.518 ) | -5.45 (-5.97 to -4.93) | -73.661 |
| Kenya | Rheumatic heart disease | Both | 315 ( 191 to 476 ) | 476 ( 291 to 720 ) | 1.294 ( 0.799 to 1.929 ) | 0.965 ( 0.6 to 1.456 ) | -1.24 (-1.31 to -1.17) | 51.111 |
| Kiribati | Rheumatic heart disease | Both | 2 ( 1 to 3 ) | 2 ( 1 to 3 ) | 2.282 ( 1.4 to 3.41 ) | 1.898 ( 1.173 to 2.957 ) | -0.59 (-0.62 to -0.56) | 0 |
| Kuwait | Rheumatic heart disease | Both | 12 ( 8 to 17 ) | 20 ( 13 to 30 ) | 0.723 ( 0.471 to 1.053 ) | 0.535 ( 0.348 to 0.784 ) | -1.38 (-1.96 to -0.8) | 66.667 |
| Kyrgyzstan | Rheumatic heart disease | Both | 92 ( 54 to 144 ) | 56 ( 35 to 84 ) | 1.941 ( 1.176 to 3.018 ) | 0.846 ( 0.521 to 1.26 ) | -2.88 (-3.08 to -2.67) | -39.13 |
| Lao People's Democratic Republic | Rheumatic heart disease | Both | 61 ( 35 to 101 ) | 71 ( 41 to 115 ) | 1.269 ( 0.773 to 2.057 ) | 0.927 ( 0.535 to 1.491 ) | -1.22 (-1.27 to -1.16) | 16.393 |
| Latvia | Rheumatic heart disease | Both | 25 ( 14 to 37 ) | 7 ( 4 to 11 ) | 0.804 ( 0.483 to 1.2 ) | 0.223 ( 0.127 to 0.342 ) | -4.98 (-5.4 to -4.56) | -72 |
| Lebanon | Rheumatic heart disease | Both | 37 ( 23 to 56 ) | 53 ( 34 to 80 ) | 1.373 ( 0.861 to 2.044 ) | 0.939 ( 0.601 to 1.401 ) | -1.37 (-1.45 to -1.29) | 43.243 |
| Lesotho | Rheumatic heart disease | Both | 30 ( 16 to 53 ) | 28 ( 15 to 46 ) | 1.594 ( 0.871 to 2.701 ) | 1.337 ( 0.739 to 2.149 ) | -0.56 (-0.59 to -0.52) | -6.667 |
| Liberia | Rheumatic heart disease | Both | 36 ( 20 to 60 ) | 57 ( 30 to 94 ) | 1.273 ( 0.731 to 2.091 ) | 0.953 ( 0.535 to 1.526 ) | -1.06 (-1.11 to -1.01) | 58.333 |
| Libya | Rheumatic heart disease | Both | 66 ( 37 to 105 ) | 49 ( 30 to 75 ) | 1.276 ( 0.758 to 1.962 ) | 0.856 ( 0.525 to 1.324 ) | -1.51 (-1.7 to -1.32) | -25.758 |
| Lithuania | Rheumatic heart disease | Both | 50 ( 29 to 75 ) | 14 ( 8 to 22 ) | 1.216 ( 0.734 to 1.839 ) | 0.358 ( 0.224 to 0.548 ) | -4.4 (-4.6 to -4.2) | -72 |
| Luxembourg | Rheumatic heart disease | Both | 2 ( 1 to 4 ) | 5 ( 3 to 8 ) | 0.497 ( 0.292 to 0.766 ) | 0.586 ( 0.358 to 0.906 ) | 0.59 (0.28 to 0.89) | 150 |
| Madagascar | Rheumatic heart disease | Both | 151 ( 66 to 268 ) | 288 ( 162 to 455 ) | 1.034 ( 0.503 to 1.723 ) | 0.866 ( 0.508 to 1.359 ) | -0.57 (-0.61 to -0.53) | 90.728 |
| Malawi | Rheumatic heart disease | Both | 83 ( 47 to 130 ) | 114 ( 65 to 184 ) | 0.731 ( 0.437 to 1.134 ) | 0.534 ( 0.309 to 0.838 ) | -1.14 (-1.19 to -1.09) | 37.349 |
| Malaysia | Rheumatic heart disease | Both | 267 ( 153 to 425 ) | 197 ( 125 to 287 ) | 1.364 ( 0.811 to 2.126 ) | 0.646 ( 0.405 to 0.957 ) | -2.23 (-2.42 to -2.04) | -26.217 |
| Maldives | Rheumatic heart disease | Both | 3 ( 2 to 5 ) | 3 ( 2 to 4 ) | 1.071 ( 0.61 to 1.666 ) | 0.553 ( 0.34 to 0.812 ) | -2.27 (-2.34 to -2.19) | 0 |
| Mali | Rheumatic heart disease | Both | 87 ( 47 to 139 ) | 188 ( 110 to 297 ) | 0.965 ( 0.535 to 1.537 ) | 0.748 ( 0.444 to 1.163 ) | -0.76 (-0.88 to -0.63) | 116.092 |
| Malta | Rheumatic heart disease | Both | 1 ( 1 to 2 ) | 3 ( 2 to 5 ) | 0.328 ( 0.201 to 0.487 ) | 0.382 ( 0.232 to 0.591 ) | 1.13 (0.93 to 1.33) | 200 |
| Marshall Islands | Rheumatic heart disease | Both | 1 ( 1 to 2 ) | 1 ( 1 to 2 ) | 2.383 ( 1.539 to 3.593 ) | 1.895 ( 1.127 to 2.897 ) | -0.86 (-0.9 to -0.83) | 0 |
| Mauritania | Rheumatic heart disease | Both | 32 ( 17 to 53 ) | 44 ( 24 to 75 ) | 1.343 ( 0.76 to 2.198 ) | 0.906 ( 0.511 to 1.51 ) | -1.28 (-1.38 to -1.18) | 37.5 |
| Mauritius | Rheumatic heart disease | Both | 11 ( 6 to 18 ) | 4 ( 3 to 6 ) | 0.97 ( 0.558 to 1.568 ) | 0.397 ( 0.246 to 0.583 ) | -0.82 (-1.76 to 0.12) | -63.636 |
| Mexico | Rheumatic heart disease | Both | 1050 ( 673 to 1508 ) | 461 ( 285 to 695 ) | 1.408 ( 0.898 to 2.035 ) | 0.36 ( 0.223 to 0.546 ) | -4.95 (-5.12 to -4.78) | -56.095 |
| Micronesia (Federated States of) | Rheumatic heart disease | Both | 2 ( 1 to 4 ) | 2 ( 1 to 3 ) | 2.212 ( 1.333 to 3.351 ) | 1.752 ( 1.041 to 2.714 ) | -0.9 (-0.96 to -0.84) | 0 |
| Monaco | Rheumatic heart disease | Both | 0 ( 0 to 0 ) | 0 ( 0 to 0 ) | 0.232 ( 0.142 to 0.36 ) | 0.237 ( 0.142 to 0.361 ) | 0.14 (-0.06 to 0.34) | #NUM! |
| Mongolia | Rheumatic heart disease | Both | 29 ( 17 to 45 ) | 22 ( 14 to 33 ) | 1.322 ( 0.801 to 2.001 ) | 0.781 ( 0.478 to 1.158 ) | -1.79 (-1.86 to -1.72) | -24.138 |
| Montenegro | Rheumatic heart disease | Both | 3 ( 2 to 5 ) | 3 ( 2 to 4 ) | 0.488 ( 0.317 to 0.732 ) | 0.343 ( 0.209 to 0.52 ) | -1.36 (-1.44 to -1.28) | 0 |
| Morocco | Rheumatic heart disease | Both | 514 ( 283 to 827 ) | 421 ( 259 to 672 ) | 1.724 ( 0.991 to 2.696 ) | 1.198 ( 0.736 to 1.91 ) | -1.27 (-1.31 to -1.23) | -18.093 |
| Mozambique | Rheumatic heart disease | Both | 94 ( 55 to 146 ) | 180 ( 105 to 283 ) | 0.659 ( 0.385 to 1.027 ) | 0.565 ( 0.336 to 0.875 ) | -0.46 (-0.57 to -0.36) | 91.489 |
| Myanmar | Rheumatic heart disease | Both | 577 ( 314 to 929 ) | 516 ( 300 to 813 ) | 1.278 ( 0.722 to 1.993 ) | 0.913 ( 0.532 to 1.427 ) | -1.26 (-1.31 to -1.21) | -10.572 |
| Namibia | Rheumatic heart disease | Both | 31 ( 16 to 52 ) | 35 ( 17 to 61 ) | 1.736 ( 0.94 to 2.9 ) | 1.32 ( 0.676 to 2.252 ) | -0.94 (-0.98 to -0.9) | 12.903 |
| Nauru | Rheumatic heart disease | Both | 0 ( 0 to 0 ) | 0 ( 0 to 0 ) | 1.781 ( 1.112 to 2.715 ) | 1.55 ( 0.954 to 2.454 ) | -0.59 (-0.69 to -0.5) | #NUM! |
| Nepal | Rheumatic heart disease | Both | 791 ( 491 to 1156 ) | 1136 ( 734 to 1759 ) | 4.371 ( 2.775 to 6.275 ) | 3.726 ( 2.39 to 5.656 ) | -0.65 (-0.69 to -0.61) | 43.616 |
| Netherlands | Rheumatic heart disease | Both | 40 ( 23 to 66 ) | 139 ( 77 to 227 ) | 0.219 ( 0.127 to 0.35 ) | 0.422 ( 0.251 to 0.664 ) | 2.22 (1.65 to 2.79) | 247.5 |
| New Zealand | Rheumatic heart disease | Both | 47 ( 29 to 70 ) | 71 ( 45 to 106 ) | 1.268 ( 0.788 to 1.87 ) | 1.096 ( 0.695 to 1.594 ) | -0.4 (-0.54 to -0.26) | 51.064 |
| Nicaragua | Rheumatic heart disease | Both | 32 ( 21 to 50 ) | 19 ( 12 to 28 ) | 0.91 ( 0.579 to 1.323 ) | 0.303 ( 0.191 to 0.444 ) | -4.02 (-4.19 to -3.86) | -40.625 |
| Niger | Rheumatic heart disease | Both | 131 ( 71 to 218 ) | 304 ( 161 to 509 ) | 1.376 ( 0.782 to 2.217 ) | 1.062 ( 0.608 to 1.702 ) | -0.85 (-0.95 to -0.76) | 132.061 |
| Nigeria | Rheumatic heart disease | Both | 950 ( 556 to 1481 ) | 1486 ( 866 to 2309 ) | 0.926 ( 0.554 to 1.441 ) | 0.587 ( 0.352 to 0.912 ) | -1.79 (-1.91 to -1.67) | 56.421 |
| Niue | Rheumatic heart disease | Both | 0 ( 0 to 0 ) | 0 ( 0 to 0 ) | 1.809 ( 1.083 to 2.737 ) | 1.499 ( 0.913 to 2.373 ) | -0.8 (-0.84 to -0.75) | #NUM! |
| North Macedonia | Rheumatic heart disease | Both | 12 ( 7 to 19 ) | 8 ( 5 to 13 ) | 0.628 ( 0.376 to 0.983 ) | 0.325 ( 0.202 to 0.47 ) | -2.35 (-2.5 to -2.19) | -33.333 |
| Northern Mariana Islands | Rheumatic heart disease | Both | 1 ( 0 to 1 ) | 1 ( 0 to 1 ) | 1.494 ( 0.906 to 2.306 ) | 1.488 ( 0.897 to 2.322 ) | 0.12 (0.07 to 0.17) | 0 |
| Norway | Rheumatic heart disease | Both | 20 ( 10 to 34 ) | 35 ( 20 to 58 ) | 0.291 ( 0.161 to 0.484 ) | 0.337 ( 0.193 to 0.553 ) | -0.38 (-1.07 to 0.32) | 75 |
| Oman | Rheumatic heart disease | Both | 13 ( 8 to 19 ) | 20 ( 13 to 29 ) | 0.692 ( 0.45 to 1 ) | 0.482 ( 0.308 to 0.706 ) | -1.24 (-1.37 to -1.11) | 53.846 |
| Pakistan | Rheumatic heart disease | Both | 4287 ( 2716 to 6197 ) | 8300 ( 5101 to 12492 ) | 4.146 ( 2.655 to 5.853 ) | 3.792 ( 2.348 to 5.578 ) | -0.44 (-0.48 to -0.39) | 93.609 |
| Palau | Rheumatic heart disease | Both | 0 ( 0 to 1 ) | 0 ( 0 to 0 ) | 2.266 ( 1.392 to 3.359 ) | 1.865 ( 1.178 to 2.917 ) | -0.71 (-0.76 to -0.67) | #NUM! |
| Palestine | Rheumatic heart disease | Both | 35 ( 19 to 57 ) | 57 ( 29 to 94 ) | 1.398 ( 0.797 to 2.196 ) | 0.997 ( 0.558 to 1.599 ) | -1.09 (-1.21 to -0.96) | 62.857 |
| Panama | Rheumatic heart disease | Both | 26 ( 17 to 37 ) | 16 ( 10 to 23 ) | 1.218 ( 0.786 to 1.773 ) | 0.365 ( 0.229 to 0.533 ) | -4.37 (-4.64 to -4.09) | -38.462 |
| Papua New Guinea | Rheumatic heart disease | Both | 112 ( 67 to 171 ) | 248 ( 143 to 392 ) | 2.913 ( 1.818 to 4.327 ) | 2.52 ( 1.496 to 3.846 ) | -0.56 (-0.59 to -0.52) | 121.429 |
| Paraguay | Rheumatic heart disease | Both | 49 ( 27 to 82 ) | 51 ( 32 to 77 ) | 1.088 ( 0.644 to 1.716 ) | 0.709 ( 0.444 to 1.059 ) | -1.49 (-1.55 to -1.43) | 4.082 |
| Peru | Rheumatic heart disease | Both | 154 ( 98 to 228 ) | 186 ( 117 to 270 ) | 0.835 ( 0.531 to 1.213 ) | 0.53 ( 0.332 to 0.766 ) | -1.62 (-1.73 to -1.51) | 20.779 |
| Philippines | Rheumatic heart disease | Both | 806 ( 506 to 1224 ) | 1048 ( 626 to 1586 ) | 1.072 ( 0.675 to 1.6 ) | 0.879 ( 0.53 to 1.33 ) | -0.73 (-0.79 to -0.67) | 30.025 |
| Poland | Rheumatic heart disease | Both | 752 ( 462 to 1132 ) | 463 ( 276 to 718 ) | 1.788 ( 1.112 to 2.688 ) | 0.744 ( 0.453 to 1.115 ) | -2.89 (-2.99 to -2.78) | -38.431 |
| Portugal | Rheumatic heart disease | Both | 93 ( 57 to 143 ) | 132 ( 75 to 202 ) | 0.73 ( 0.457 to 1.081 ) | 0.633 ( 0.364 to 0.972 ) | -0.45 (-0.61 to -0.3) | 41.935 |
| Puerto Rico | Rheumatic heart disease | Both | 21 ( 13 to 31 ) | 16 ( 10 to 23 ) | 0.567 ( 0.36 to 0.848 ) | 0.473 ( 0.306 to 0.677 ) | -1.11 (-1.27 to -0.94) | -23.81 |
| Qatar | Rheumatic heart disease | Both | 4 ( 2 to 5 ) | 19 ( 12 to 28 ) | 0.94 ( 0.608 to 1.369 ) | 0.773 ( 0.487 to 1.116 ) | -0.78 (-0.97 to -0.58) | 375 |
| Republic of Korea | Rheumatic heart disease | Both | 137 ( 89 to 200 ) | 260 ( 154 to 408 ) | 0.372 ( 0.235 to 0.547 ) | 0.34 ( 0.21 to 0.521 ) | 0.04 (-0.22 to 0.3) | 89.781 |
| Republic of Moldova | Rheumatic heart disease | Both | 57 ( 35 to 86 ) | 13 ( 8 to 19 ) | 1.283 ( 0.791 to 1.926 ) | 0.35 ( 0.211 to 0.511 ) | -4.77 (-4.95 to -4.59) | -77.193 |
| Romania | Rheumatic heart disease | Both | 180 ( 112 to 268 ) | 75 ( 44 to 118 ) | 0.726 ( 0.455 to 1.079 ) | 0.318 ( 0.192 to 0.479 ) | -3.31 (-3.76 to -2.86) | -58.333 |
| Russian Federation | Rheumatic heart disease | Both | 1386 ( 872 to 2050 ) | 590 ( 326 to 975 ) | 0.86 ( 0.551 to 1.264 ) | 0.284 ( 0.165 to 0.45 ) | -4.11 (-4.5 to -3.71) | -57.431 |
| Rwanda | Rheumatic heart disease | Both | 68 ( 39 to 107 ) | 72 ( 40 to 115 ) | 0.795 ( 0.472 to 1.237 ) | 0.521 ( 0.303 to 0.811 ) | -1.64 (-1.74 to -1.54) | 5.882 |
| Saint Kitts and Nevis | Rheumatic heart disease | Both | 0 ( 0 to 0 ) | 0 ( 0 to 0 ) | 0.607 ( 0.386 to 0.88 ) | 0.306 ( 0.198 to 0.45 ) | -2.37 (-2.47 to -2.26) | #NUM! |
| Saint Lucia | Rheumatic heart disease | Both | 2 ( 1 to 3 ) | 1 ( 1 to 2 ) | 1.355 ( 0.822 to 2.089 ) | 0.699 ( 0.441 to 1.047 ) | -2.49 (-2.65 to -2.34) | -50 |
| Saint Vincent and the Grenadines | Rheumatic heart disease | Both | 2 ( 1 to 3 ) | 1 ( 1 to 1 ) | 1.533 ( 0.894 to 2.478 ) | 0.813 ( 0.504 to 1.213 ) | -2.26 (-2.4 to -2.12) | -50 |
| Samoa | Rheumatic heart disease | Both | 4 ( 2 to 6 ) | 4 ( 2 to 6 ) | 1.958 ( 1.158 to 3.028 ) | 1.708 ( 0.983 to 2.77 ) | -0.57 (-0.61 to -0.52) | 0 |
| San Marino | Rheumatic heart disease | Both | 0 ( 0 to 0 ) | 0 ( 0 to 1 ) | 0.814 ( 0.49 to 1.267 ) | 0.747 ( 0.462 to 1.118 ) | -0.09 (-0.15 to -0.04) | #NUM! |
| Sao Tome and Principe | Rheumatic heart disease | Both | 2 ( 1 to 4 ) | 3 ( 2 to 5 ) | 1.64 ( 0.908 to 2.581 ) | 1.3 ( 0.724 to 2.168 ) | -0.79 (-0.86 to -0.72) | 50 |
| Saudi Arabia | Rheumatic heart disease | Both | 113 ( 72 to 169 ) | 102 ( 65 to 151 ) | 0.664 ( 0.426 to 0.975 ) | 0.32 ( 0.203 to 0.474 ) | -2.61 (-2.71 to -2.52) | -9.735 |
| Senegal | Rheumatic heart disease | Both | 149 ( 84 to 252 ) | 202 ( 108 to 328 ) | 1.684 ( 0.977 to 2.704 ) | 1.176 ( 0.669 to 1.846 ) | -1.2 (-1.25 to -1.15) | 35.57 |
| Serbia | Rheumatic heart disease | Both | 36 ( 22 to 54 ) | 25 ( 14 to 37 ) | 0.353 ( 0.215 to 0.522 ) | 0.199 ( 0.121 to 0.292 ) | -2.71 (-3.11 to -2.32) | -30.556 |
| Seychelles | Rheumatic heart disease | Both | 0 ( 0 to 0 ) | 0 ( 0 to 0 ) | 0.381 ( 0.239 to 0.584 ) | 0.228 ( 0.139 to 0.341 ) | -1.25 (-1.56 to -0.94) | #NUM! |
| Sierra Leone | Rheumatic heart disease | Both | 62 ( 35 to 105 ) | 100 ( 56 to 165 ) | 1.31 ( 0.776 to 2.135 ) | 1.017 ( 0.585 to 1.59 ) | -0.8 (-0.84 to -0.76) | 61.29 |
| Singapore | Rheumatic heart disease | Both | 10 ( 6 to 14 ) | 20 ( 13 to 30 ) | 0.379 ( 0.242 to 0.573 ) | 0.321 ( 0.205 to 0.468 ) | -0.64 (-0.74 to -0.55) | 100 |
| Slovakia | Rheumatic heart disease | Both | 32 ( 20 to 48 ) | 28 ( 17 to 43 ) | 0.584 ( 0.356 to 0.868 ) | 0.412 ( 0.251 to 0.611 ) | -0.97 (-1.25 to -0.69) | -12.5 |
| Slovenia | Rheumatic heart disease | Both | 22 ( 12 to 34 ) | 31 ( 17 to 53 ) | 0.936 ( 0.536 to 1.445 ) | 0.772 ( 0.447 to 1.231 ) | -0.67 (-1.12 to -0.22) | 40.909 |
| Solomon Islands | Rheumatic heart disease | Both | 7 ( 4 to 12 ) | 13 ( 8 to 20 ) | 2.183 ( 1.332 to 3.307 ) | 1.854 ( 1.129 to 2.842 ) | -0.63 (-0.66 to -0.6) | 85.714 |
| Somalia | Rheumatic heart disease | Both | 88 ( 47 to 148 ) | 164 ( 94 to 257 ) | 0.897 ( 0.499 to 1.42 ) | 0.654 ( 0.388 to 1.02 ) | -1.23 (-1.31 to -1.15) | 86.364 |
| South Africa | Rheumatic heart disease | Both | 673 ( 395 to 1055 ) | 640 ( 384 to 976 ) | 1.556 ( 0.926 to 2.398 ) | 1.149 ( 0.701 to 1.746 ) | -1.01 (-1.09 to -0.93) | -4.903 |
| South Sudan | Rheumatic heart disease | Both | 62 ( 30 to 105 ) | 80 ( 46 to 127 ) | 0.877 ( 0.472 to 1.429 ) | 0.734 ( 0.427 to 1.152 ) | -0.68 (-0.79 to -0.57) | 29.032 |
| Spain | Rheumatic heart disease | Both | 701 ( 412 to 1090 ) | 1025 ( 624 to 1518 ) | 1.332 ( 0.795 to 2.04 ) | 1.118 ( 0.694 to 1.621 ) | -0.75 (-0.86 to -0.63) | 46.22 |
| Sri Lanka | Rheumatic heart disease | Both | 107 ( 69 to 157 ) | 86 ( 54 to 126 ) | 0.596 ( 0.389 to 0.873 ) | 0.402 ( 0.254 to 0.593 ) | -1.21 (-1.29 to -1.13) | -19.626 |
| Sudan | Rheumatic heart disease | Both | 372 ( 208 to 612 ) | 524 ( 305 to 848 ) | 1.568 ( 0.911 to 2.467 ) | 1.064 ( 0.631 to 1.676 ) | -1.34 (-1.39 to -1.29) | 40.86 |
| Suriname | Rheumatic heart disease | Both | 5 ( 3 to 8 ) | 3 ( 2 to 5 ) | 1.14 ( 0.689 to 1.886 ) | 0.613 ( 0.381 to 0.899 ) | -2.21 (-2.29 to -2.13) | -40 |
| Sweden | Rheumatic heart disease | Both | 95 ( 52 to 157 ) | 107 ( 58 to 170 ) | 0.646 ( 0.376 to 1.037 ) | 0.483 ( 0.269 to 0.76 ) | -1.33 (-1.67 to -0.99) | 12.632 |
| Switzerland | Rheumatic heart disease | Both | 40 ( 23 to 63 ) | 58 ( 32 to 89 ) | 0.443 ( 0.269 to 0.677 ) | 0.414 ( 0.251 to 0.613 ) | 0.09 (-0.34 to 0.53) | 45 |
| Syrian Arab Republic | Rheumatic heart disease | Both | 275 ( 152 to 442 ) | 121 ( 70 to 191 ) | 1.708 ( 1.001 to 2.638 ) | 0.892 ( 0.506 to 1.432 ) | -2.68 (-2.9 to -2.45) | -56 |
| Taiwan (Province of China) | Rheumatic heart disease | Both | 177 ( 107 to 266 ) | 153 ( 87 to 253 ) | 1.113 ( 0.668 to 1.707 ) | 0.392 ( 0.227 to 0.636 ) | -4.05 (-4.31 to -3.78) | -13.559 |
| Tajikistan | Rheumatic heart disease | Both | 89 ( 50 to 145 ) | 155 ( 85 to 258 ) | 1.349 ( 0.777 to 2.147 ) | 1.375 ( 0.765 to 2.271 ) | -0.12 (-0.19 to -0.04) | 74.157 |
| Thailand | Rheumatic heart disease | Both | 459 ( 271 to 718 ) | 287 ( 182 to 425 ) | 0.771 ( 0.468 to 1.203 ) | 0.482 ( 0.304 to 0.72 ) | -1.64 (-1.73 to -1.55) | -37.473 |
| Timor-Leste | Rheumatic heart disease | Both | 13 ( 7 to 21 ) | 18 ( 9 to 29 ) | 1.454 ( 0.824 to 2.339 ) | 1.119 ( 0.602 to 1.765 ) | -0.99 (-1.04 to -0.93) | 38.462 |
| Togo | Rheumatic heart disease | Both | 58 ( 31 to 98 ) | 85 ( 45 to 143 ) | 1.326 ( 0.762 to 2.166 ) | 0.942 ( 0.527 to 1.541 ) | -1.18 (-1.23 to -1.12) | 46.552 |
| Tokelau | Rheumatic heart disease | Both | 0 ( 0 to 0 ) | 0 ( 0 to 0 ) | 2.119 ( 1.292 to 3.235 ) | 1.545 ( 0.967 to 2.385 ) | -1.24 (-1.3 to -1.18) | #NUM! |
| Tonga | Rheumatic heart disease | Both | 2 ( 1 to 3 ) | 2 ( 1 to 3 ) | 1.998 ( 1.223 to 3.074 ) | 1.524 ( 0.918 to 2.396 ) | -0.94 (-0.98 to -0.9) | 0 |
| Trinidad and Tobago | Rheumatic heart disease | Both | 16 ( 9 to 26 ) | 8 ( 5 to 12 ) | 1.213 ( 0.684 to 1.898 ) | 0.666 ( 0.419 to 0.99 ) | -2.21 (-2.38 to -2.05) | -50 |
| Tunisia | Rheumatic heart disease | Both | 77 ( 50 to 115 ) | 71 ( 45 to 102 ) | 0.893 ( 0.579 to 1.308 ) | 0.616 ( 0.395 to 0.902 ) | -1.25 (-1.35 to -1.16) | -7.792 |
| Turkey | Rheumatic heart disease | Both | 452 ( 296 to 658 ) | 542 ( 348 to 777 ) | 0.788 ( 0.524 to 1.147 ) | 0.654 ( 0.422 to 0.946 ) | -0.75 (-0.95 to -0.55) | 19.912 |
| Turkmenistan | Rheumatic heart disease | Both | 54 ( 31 to 87 ) | 23 ( 14 to 34 ) | 1.306 ( 0.783 to 2.054 ) | 0.465 ( 0.289 to 0.688 ) | -4.16 (-4.61 to -3.71) | -57.407 |
| Tuvalu | Rheumatic heart disease | Both | 0 ( 0 to 0 ) | 0 ( 0 to 0 ) | 2.192 ( 1.36 to 3.347 ) | 1.634 ( 0.951 to 2.512 ) | -1.15 (-1.22 to -1.07) | #NUM! |
| Uganda | Rheumatic heart disease | Both | 225 ( 114 to 394 ) | 325 ( 191 to 515 ) | 1.077 ( 0.605 to 1.793 ) | 0.697 ( 0.415 to 1.085 ) | -1.56 (-1.62 to -1.51) | 44.444 |
| Ukraine | Rheumatic heart disease | Both | 204 ( 110 to 332 ) | 107 ( 59 to 168 ) | 0.322 ( 0.177 to 0.511 ) | 0.167 ( 0.096 to 0.253 ) | -2.42 (-2.66 to -2.19) | -47.549 |
| United Arab Emirates | Rheumatic heart disease | Both | 28 ( 16 to 45 ) | 80 ( 49 to 121 ) | 1.588 ( 0.971 to 2.541 ) | 1.172 ( 0.707 to 1.801 ) | -0.9 (-0.97 to -0.82) | 185.714 |
| United Kingdom | Rheumatic heart disease | Both | 384 ( 226 to 605 ) | 305 ( 187 to 483 ) | 0.458 ( 0.285 to 0.705 ) | 0.259 ( 0.163 to 0.393 ) | -2.38 (-2.53 to -2.23) | -20.573 |
| United Republic of Tanzania | Rheumatic heart disease | Both | 207 ( 120 to 328 ) | 345 ( 197 to 546 ) | 0.7 ( 0.412 to 1.094 ) | 0.551 ( 0.327 to 0.86 ) | -0.62 (-0.72 to -0.52) | 66.667 |
| United States of America | Rheumatic heart disease | Both | 2056 ( 1240 to 3136 ) | 2081 ( 1273 to 3175 ) | 0.693 ( 0.432 to 1.035 ) | 0.43 ( 0.262 to 0.638 ) | -1.69 (-2.15 to -1.24) | 1.216 |
| United States Virgin Islands | Rheumatic heart disease | Both | 1 ( 0 to 1 ) | 0 ( 0 to 0 ) | 0.596 ( 0.384 to 0.88 ) | 0.416 ( 0.266 to 0.615 ) | -1.45 (-1.54 to -1.35) | -100 |
| Uruguay | Rheumatic heart disease | Both | 13 ( 8 to 20 ) | 9 ( 5 to 13 ) | 0.386 ( 0.244 to 0.58 ) | 0.184 ( 0.115 to 0.273 ) | -2.37 (-2.49 to -2.25) | -30.769 |
| Uzbekistan | Rheumatic heart disease | Both | 459 ( 264 to 722 ) | 449 ( 268 to 692 ) | 1.889 ( 1.113 to 2.919 ) | 1.381 ( 0.814 to 2.103 ) | -0.94 (-1.21 to -0.67) | -2.179 |
| Vanuatu | Rheumatic heart disease | Both | 4 ( 2 to 7 ) | 7 ( 4 to 11 ) | 2.672 ( 1.574 to 4.141 ) | 2.227 ( 1.35 to 3.439 ) | -0.65 (-0.69 to -0.62) | 75 |
| Venezuela (Bolivarian Republic of) | Rheumatic heart disease | Both | 110 ( 70 to 161 ) | 47 ( 30 to 68 ) | 0.586 ( 0.379 to 0.848 ) | 0.174 ( 0.111 to 0.251 ) | -4.66 (-4.94 to -4.37) | -57.273 |
| Viet Nam | Rheumatic heart disease | Both | 753 ( 489 to 1127 ) | 718 ( 463 to 1062 ) | 1.041 ( 0.678 to 1.539 ) | 0.763 ( 0.489 to 1.127 ) | -1.21 (-1.27 to -1.14) | -4.648 |
| Yemen | Rheumatic heart disease | Both | 282 ( 157 to 446 ) | 442 ( 243 to 683 ) | 1.649 ( 0.959 to 2.526 ) | 1.147 ( 0.68 to 1.75 ) | -1.43 (-1.5 to -1.36) | 56.738 |
| Zambia | Rheumatic heart disease | Both | 58 ( 33 to 92 ) | 99 ( 55 to 158 ) | 0.633 ( 0.37 to 0.989 ) | 0.487 ( 0.285 to 0.77 ) | -0.97 (-1.04 to -0.9) | 70.69 |
| Zimbabwe | Rheumatic heart disease | Both | 274 ( 153 to 457 ) | 364 ( 189 to 614 ) | 2.014 ( 1.153 to 3.241 ) | 1.943 ( 1.037 to 3.236 ) | -0.06 (-0.1 to -0.03) | 32.847 |
